# A Scoping Review of the Current Knowledge of the Social Determinants of Health and Infectious Diseases (Specifically COVID-19, Tuberculosis, and H1N1 Influenza) in Canadian Arctic Indigenous Communities

**DOI:** 10.3390/ijerph22010001

**Published:** 2024-12-24

**Authors:** Fariba Kolahdooz, Se Lim Jang, Sarah Deck, David Ilkiw, Gertrude Omoro, Arja Rautio, Sami Pirkola, Helle Møller, Gary Ferguson, Birgitta Evengård, Lianne Mantla-Look, Debbie DeLancey, André Corriveau, Stephanie Irlbacher-Fox, Adrian Wagg, Cindy Roache, Katherine Rittenbach, Henry J. Conter, Ryan Falk, Sangita Sharma

**Affiliations:** 1 Indigenous and Global Health Research Group, Department of Medicine, Faculty of Medicine & Dentistry, College of Health Sciences, University of Alberta, 1-126 8602 112 Street, Edmonton, AB T6G 2E1, Canada; fariba.kolahdooz@ualberta.ca (F.K.); selim@ualberta.ca (S.L.J.); sarahjdeck@gmail.com (S.D.); d.ilkiw@lse.ac.uk (D.I.); gertrudeomoro@gmail.com (G.O.); cindyroache@hotmail.com (C.R.); 2 Arctic Health Research Group, Faculty of Medicine, University of Oulu, Pentti Kaiteran Katu 1, 90570 Oulu, Finland; arja.rautio@oulu.fi; 3 Faculty of Social Sciences, Tampere University, Kalevantie 4, 33100 Tampere, Finland; sami.pirkola@tuni.fi; 4 Department of Health Sciences, Lakehead University, 955 Oliver Rd, Thunder Bay, ON P7B 5E1, Canada; hmoeller@lakeheadu.ca; 5 Institute for Research and Education to Advance Community Health, Washington State University, 1100 Olive Wy #1200, Seattle, WA 98101, USA; gary.ferguson@wsu.edu; 6 Section of Infection and Immunology, Department of Clinical Microbiology, Umeå University, Universitetstorget 4, 901 87 Umeå, Sweden; birgitta.evengard@umu.se; 7 Hotıì ts’eeda Northwest Territories SPOR SUPPORT Unit, 1000, 4920-52nd Street, Yellowknife, NT X1A 3T1, Canadafcl@northwestel.net (S.I.-F.); 8 Aurora College, 5004 54 St, Yellowknife, NT X1A 2R6, Canada; ddelancey33@gmail.com; 9 Independent Public Health Consultant for Northwest Territories and Nunavut, Yellowknife, NT X1A 1L7, Canada; andrecorriveau@icloud.com; 10 Division of Geriatric Medicine, Department of Medicine, Faculty of Medicine & Dentistry, College of Health Sciences, University of Alberta, 1-198 11350 83 Avenue, Edmonton, AB T6G 2P4, Canada; wagg@ualberta.ca; 11 Department of Psychiatry, Cumming School of Medicine, University of Calgary, 3330 Hospital Dr NW, Calgary, AB T2N 4N1, Canada; katherine.rittenbach@ucalgary.ca; 12 Hoffmann-La Roche Limited, 7070 Mississauga Rd, Mississauga, ON L5N 5M8, Canada; henry.conter@roche.com; 13 Beaufort-Delta Region, Northwest Territories Health and Social Services Authority, Government of the Northwest Territories, Inuvik, NT XOE 0T0, Canada

**Keywords:** Canadian Arctic, Indigenous, social determinants of health, infectious diseases, pandemic, COVID-19

## Abstract

Social determinants of health (SDHs) and the impact of colonization can make Canadian Arctic Indigenous communities susceptible to infectious diseases, including the coronavirus disease 2019 (COVID-19). This scoping review followed the PRISMA guidelines for scoping reviews and studied what is known about selected pandemics (COVID-19, tuberculosis, and H1N1 influenza) and SDHs (healthcare accessibility, food insecurity, mental health, cultural continuity, housing, community infrastructure, and socioeconomic status (SES)) for Canadian Arctic Indigenous communities. Original studies published in English and French up to October 2024 were located in databases (PubMed, Medline, and CINAHL), *AlterNative: An International Journal of Indigenous Peoples*, and through reference tracking. We included 118 studies: 6 relating to COVID-19, 5 to influenza, 5 to TB, 27 to food insecurity, 26 to healthcare access, 22 to mental health, 9 to SES, 8 to housing, 7 to cultural continuity, and 3 to community infrastructure. SDHs affecting Indigenous individuals include food insecurity, limited healthcare access, mental health challenges, low SES, suboptimal housing, and limited cultural continuity. These findings are relevant to other Arctic regions. It is crucial to understand how SDHs impact the health of Arctic communities and to utilize this information to inform policy and practice decisions for pandemic prevention, management, and treatment. Many SDHs pose challenges for preventing and managing infectious diseases.

## 1. Introduction

Social determinants of health (SDHs) are strong predictors of how population groups can mitigate the impact of and prevent transmission of infectious diseases, including COVID-19. In the United States, COVID-19 led to the deaths of 1 in 2300 Indigenous people compared with 1 in 3600 non-Hispanic white people [1], a disparity attributed to structural and socioeconomic inequalities [2,3]. Arctic Indigenous communities are particularly susceptible to the pandemic’s effects due to unique SDH challenges, including the long-lasting and ongoing trauma resulting from colonization [2,4].

The Arctic extends into eight nations: Denmark (Greenland), United States (Alaska), Sweden, Norway, Finland, Russia, Iceland, and Canada. Of the estimated 7 million inhabitants, almost 10% are Indigenous [5]. In Canada, Indigenous peoples account for 5% of the total population and over 75% of the Arctic population [6]. The Arctic accounts for approximately 40% of Canada’s landmass and has the lowest population density (3.3 persons per km^2^) of all Arctic Nations after Iceland [7]. Although no clear definition of the Canadian Arctic exists, it politically includes the northern territories of Nunavut, Northwest Territories (NWT), and Yukon, and can anthropologically include parts of Northern Quebec and Newfoundland and Labrador [7]. Climatically, the Arctic expands into the Arctic tundra of Northern Ontario [8,9].

When discussing the relationship between SDHs, health outcomes, and infectious diseases in the Canadian Arctic, a historical perspective is essential. Cross-continental travel from Europe to America began in the late 1400s [10], introducing previously non-existent diseases into Indigenous communities [10]. Historical accounts report that the Labrador Inuit’s contact with John Cabot in 1498 and subsequent contact with whalers, explorers, missionaries, and traders, led to numerous outbreaks of diseases, including smallpox, syphilis, influenza, and measles [11]. Inuit in the Canadian Arctic experienced substantial environmental and lifestyle changes as a result of European settlement. In the 20th century, the “High Arctic Relocation” involved the Canadian government relocating nomadic Inuit from Northern Quebec to permanent government-built settlements in the High Arctic; this caused an outbreak of measles among the Inuit as well as the dogs (quimmiit), which were relied upon for survival [12,13]. Many of the new settlements were poorly located and constructed, lacked proper sanitation, and were distant from food sources. Diseases such as tuberculosis (TB) were rampant, and individuals were forcibly transferred to southern hospitals for treatment, separating patients from family, language, culture, and communities—sometimes forever. Despite the declining trend of TB in the Arctic, TB rates are still approximately 300 times higher among Inuit than non-Indigenous Canadians [14]. During the 1918–1919 influenza pandemic, mortality was as high as 90% in some Indigenous communities in Labrador [15]. During the 2009–2010 H1N1 pandemic, Indigenous communities across Canada had a disproportionately high risk of disease acquisition and severe manifestation of the infection compared with non-Indigenous communities [16]. Such increased risks for infectious diseases and poor health outcomes during pandemics in Indigenous communities are related to risk factors such as overcrowded housing, limited healthcare access [17], delays in hospitalization, and higher comorbidity rates [16].

The all-too-brief history above explicates an entangled relationship between infectious disease and colonialism that continues to affect Indigenous communities through socially mediated health inequities and adverse health experiences and outcomes [18,19]. For more than 100 years, Indigenous children were forcibly relocated to residential schools and disconnected from family, community, language, and culture; many experienced physical, sexual, and psychological abuse [20]. Many residential schools provided poor-quality diets and unsanitary and overcrowded living conditions [19,20,21]. Residential schools ran from the 1870s until 1996, and the adverse effects continue to be felt through psychological mechanisms, intergenerational trauma, and biologically embedded mechanisms such as malnutrition [19,20,21]. Average life expectancy is approximately 10 years shorter for Inuit, at 72.4 years compared with 82.9 years for non-Indigenous populations in Canada [22]. Intergenerational trauma in Indigenous communities has also resulted in disadvantageous socioeconomic status (SES) and a higher burden of illness resulting from poor general health and increased risk of chronic diseases, many of which were foreign to Indigenous communities, such as depression, substance abuse, and suicide [19,20]. For Indigenous health, ongoing colonialism and assimilation practices not only result in health inequity as SDHs themselves, but they also lead to other SDHs, such as disparities in healthcare access [18,23]. The literature suggests that SDH indicators are associated with morbidity and mortality from many infectious diseases, including COVID-19, and are a critical part of pandemic public health priorities, goals, and policies [24,25,26]. Given the historical accounts regarding colonization, SDHs specific to Arctic Indigenous communities have been identified, including food insecurity, healthcare access, mental health, SES, housing, cultural continuity, and community infrastructure [27,28]. These SDHs have made Indigenous communities in the Arctic vulnerable to numerous infectious diseases, including respiratory diseases such as TB and influenza [29]. Studies on the association between SDHs and infectious disease in the Canadian Arctic are scarce. For instance, only two studies have been identified that explore the role of a limited number of SDHs in managing COVID-19 in Canadian Arctic communities [30,31]. Reviewing the current literature regarding SDHs in the Canadian Arctic is crucial to improving COVID-19 outcomes and for future pandemic planning and preparedness.

This study’s aim was to undertake a scoping review of what is known about SDHs (food insecurity; healthcare access; mental health; cultural continuity; housing; community infrastructure; and socioeconomic status (SES), which encompasses livelihoods, employment, income, and education) and infectious diseases (specifically COVID-19, tuberculosis, and H1N1 influenza) for Canadian Arctic Indigenous communities. This scoping review was intended to identify and summarize the available evidence regarding this broad aim [29], which may shed light on the importance of considering SDHs in the prevention and management of infectious disease outbreaks and pandemic preparedness policies.

## 2. Materials and Methods

The methodological framework described by Levac, Colquhoun, and O’Brien [32], as well as the Preferred Reporting Items for Systematic Reviews and Meta-Analyses Extension for Scoping Reviews Statement [33], were used for this scoping review (Table S1). This review did not require ethics approval, as no human subjects were involved in the research. We merely examined the already-published literature.

Arctic Canada was defined as the three northern territories (Yukon, NWT, and Nunavut), the northern regions of Quebec and Newfoundland and Labrador, and remote regions in northern Ontario close to the northern coastline. In addition to the COVID-19 pandemic, a preliminary search of the literature identified eight infectious diseases that have substantially affected Canadian Arctic Indigenous communities: TB, H1N1 influenza, sexually transmitted infections (STIs) including human immunodeficiency virus, diphtheria, smallpox, Helicobacter pylori (H. pylori) infection, and hepatitis. This review focussed on TB, H1N1 influenza, and COVID-19 because of the similarities in their methods of transmission.

### 2.1. Search Strategies 

After consulting seminal works related to SDHs in Indigenous communities [27,28] and selecting the three infectious diseases, the following search terms were selected and searched for with command and operator terms such as AND, OR, asterisks, and quotation marks: Arctic Canada, COVID-19, tuberculosis (TB), influenza (H1N1), food insecurity (traditional/country food), healthcare access (utilization, accessibility, and medical travel), mental health (depression, suicide, and substance/alcohol use), SES (livelihoods, employment, income, and education), housing, cultural continuity (language), community infrastructure, environment (climate), remoteness, and discrimination (colonialism). Mental health was considered to include suicide prevention campaigns, violence reduction, and addiction and substance abuse treatment. Our review considered mental health as the environment, individual, and social elements that affect one’s mental health. Three scientific databases (PubMed, Medline, and CINAHL) [34] and *AlterNative: An International Journal of Indigenous Peoples* (which included references not found in the other databases) were searched, and all articles published at the time of the search in October 2024 were retrieved. The search was limited to original studies published in English and French (Table S2). The references for other types of publications, including reviews and commentaries, were reviewed for additional articles.

### 2.2. Study Selection

Articles were deemed eligible if they investigated at least one of the predefined areas of interest, they pertained to Arctic Canada as defined above, and the full text was available through the University of Alberta library. Two authors screened the titles and abstracts, reviewed the full texts, and discussed the eligibility of the studies, identifying and presenting to each other the study settings and findings related to the search terms. A third author verified the screening criteria and assisted in resolving any disagreement between the two authors through extensive discussions. Multinational studies were included if findings were presented by country and results related solely to Canada were available. Following discussion, the search terms “environment” and “remoteness” were removed, as they yielded too many search results and were deemed out of this review’s scope. The term “discrimination” was removed, as it was discussed in the context of other SDHs and was impossible to separate. Following a full-text review, articles that were deemed out of scope or not about the Canadian Arctic were excluded.

### 2.3. Data Extraction

The following information was extracted from the eligible papers: year of publication, location, population, methods, and the summary of findings. The authors extensively discussed how to present the results, selecting article excerpts that best reflected significant study findings (Table S3).

### 2.4. Summarizing the Results and Quality Appraisal

Included articles were categorized into one of the three infectious diseases (COVID-19, H1N1 influenza, and TB) or one of the seven areas of Arctic Indigenous SDHs (healthcare access, food insecurity, mental health, SES, housing, cultural continuity, and community infrastructure). Three authors individually utilized NVivo 12 to thematically analyse and code the findings of eligible articles and verified the codes through discussion. Another author mediated any discrepancies in coding and analysis to avoid bias and improve the trustworthiness of the results presented. Subthemes emerged from the thematic analysis. Relevant quotes were extracted for each subtheme (Table S4). To evaluate the quality of each study, three authors individually utilized STROBE [35] for quantitative studies, SRQR [36] for qualitative studies, and MMAT for mixed-method studies [37]. Another author mediated any discrepancies (Table S5).

## 3. Results

**Figure 1:**
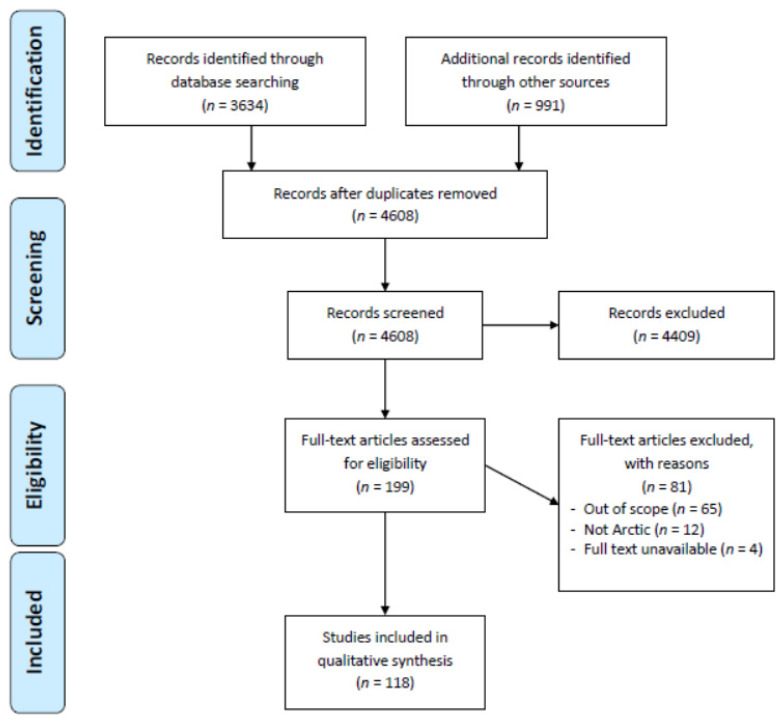
PRISMA flow diagram: selection of sources of evidence.

**Figure 2:**
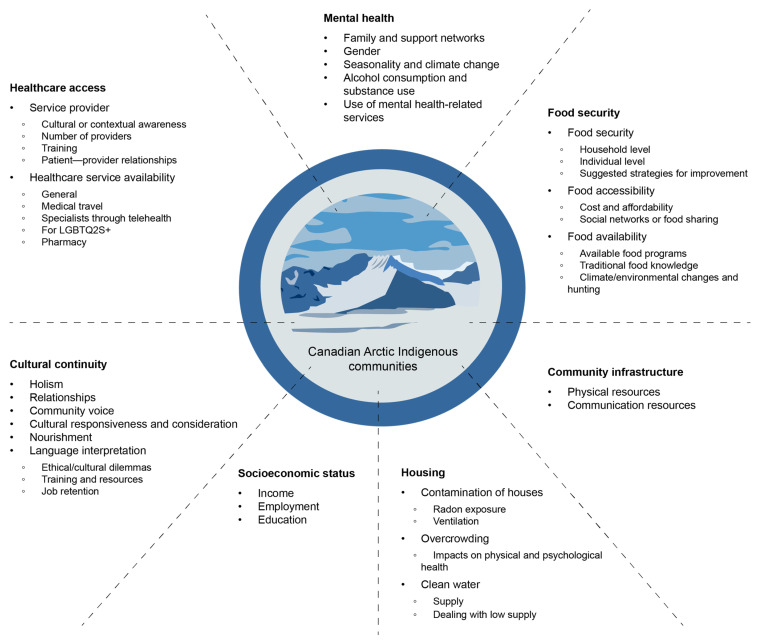
Summary of thematic analysis of selected studies about SDHs in Canadian Arctic Indigenous communities.

The database search yielded 3634 results, and an additional 991 articles were identified by manually reviewing the reference lists of secondary studies. After removing duplicates, 4608 articles remained. After screening for titles and abstracts, 199 articles remained and received a full-text assessment. Finally, 118 articles remained eligible and were included (Figure 1, PRISMA flow diagram: selection of sources of evidence). Of the articles, 6 focussed on COVID-19, 5 on influenza, and 5 on TB; 27 pertained to food insecurity, 26 to healthcare accessibility, 22 to mental health, 9 to SES, 8 to housing, 7 to cultural continuity, and 3 to community infrastructure. The themes that emerged are visually summarized in Figure 2, Summary of thematic analysis of selected studies about SDHs in Canadian Arctic Indigenous communities. Sixty articles were quantitative, fifty-two were qualitative, and six were mixed-method.

### 3.1. Infectious Diseases

COVID-19: Six papers examined COVID-19 in Northern Canada [4,30,31,38,39,40]. Between 21 February 2020, when the first COVID-19 case was documented in the Arctic, and 1 July 2020, Arctic Canada had isolated cases and no significant outbreaks [4,38]. The remoteness of the communities, strong public health guidelines, strict quarantine requirements, and precautions informed by an understanding of existing SDHs including limited healthcare and SES [4], as well as strict territory-wide lockdown measures [40], were integral to preventing the spread of COVID-19. Arctic Canada endured the second wave in November and December 2020 with limited fatalities due to strict public health measures and because many Indigenous community members were on the land (i.e., in minimally populated areas where hunting, fishing, and foraging occur) [38]. When the Delta variant became the dominant variant in 2021, Northern Canada showed a tsunami-like pattern, with daily cases and fatalities abruptly increasing [39]. In Iqaluit, Nunavut, community members, frontline workers, and decision makers identified challenges to addressing COVID-19, such as overcrowded housing, restricted connectivity to family due to social distancing, and mental health issues including alcohol and substance misuse [30]. However, the pandemic revealed resilience through partnerships among the government and community agencies, compassionate frontline workers, and community supports such as food hampers [30]. COVID-19 impacted healthcare access for residents in Nunavut, who utilized advanced care in Manitoba [31]. Mitigation strategies such as expanding virtual care and cross-jurisdictional healthcare coordination through electronic medical records were limited due to infrastructure barriers [31].

H1N1 Influenza: The five papers regarding HINI influenza yielded five major themes: one paper addressed hospital admissions [41], two addressed pandemic experiences [42,43], one addressed community responses [44], three addressed public health responses [42,43,44], and four addressed future pandemic plans or recommendations [17,42,43,44].

One study examined infant hospitalization for respiratory tract infections in Inuit regions during 2009 and found that H1N1 infection accounted for 12.1% of admissions [41]. In northern Ontario, the pandemic experience included prevention strategies and measures such as screening at healthcare facilities, closing down public/community centres, and postponing large gatherings [43]. Low rates of adherence to such measures by community members due to limited public campaigning and education; unsuccessful distribution of vaccines [43]; and limited resources, particularly personnel (i.e., nurses) were identified as barriers to effective prevention of the H1N1 influenza pandemic [42,43]. Recommendations for future pandemics emphasized the need for adaptability and effective communication at all levels (from governments to communities); clarification regarding roles, responsibilities, and accountability across all levels, from governments to communities, while also highlighting the importance of clearly defining roles, responsibilities, and accountability; and responsiveness to the evolving needs of communities [17,42,43,44]. Community responses encompassed specific plans implemented in phases by the communities, which tended to be dynamic from the initial implementation to the end of the pandemic in northern Ontario [44]. Authors noted the need for more community- or region-specific responses [44]. Critical public health responses during the pandemic included disease surveillance, the distribution of antiviral medications, and the provision of health services [42].

Tuberculosis: Five papers pertained to TB [45,46,47,48,49]. Nunavik, an Inuit region in Northern Quebec, had a TB incidence rate of 199.5/100,000 [47]. In 2021, TB was 300 times more prevalent in Inuit communities (170/100,000) than non-Indigenous populations (0.5/100,000) [45]. A study in Iqaluit, Nunavut noted more than half (53.0%) of patients with latent TB were Inuit, while 28.6% were non-Inuit (the ethnicity of the remaining 18.5% of patients was unknown) [46]. TB status was significantly associated with personal factors (age and ethnicity) [46] and an SDHs (educational attainment) [45]. One study concluded that socioeconomic factors play important roles in the transmission of TB [49]. Another study in Nunavut implemented interventions to prevent TB among youth utilizing training videos and suggested future studies are needed to address specific age groups while remaining sensitive to Indigenous cultures and traditions [48].

### 3.2. SDHs

Food insecurity: A total of 27 articles were included: 15 papers presented on the prevalence of food insecurity [50,51,52,53,54,55,56,57,58,59,60,61,62,63,64], 12 discussed food accessibility [50,54,55,56,58,59,65,66,67,68,69,70], and 15 discussed food availability [50,54,55,58,59,65,66,67,68,71,72,73,74,75,76].

Over 40% of individuals in an Inuit community in Northern Quebec experienced high or very high food insecurity [56]. A survey found 62.6% of adults in NWT, Nunavut, and Northern Quebec were food insecure [62]. Of 50 Inuit in a Nunavut community, 64% were food insecure [64]. Forty-five percent of the survey participants aged 13–18 years in NWT reported experiencing food insecurity [60]. At the individual level, food insecurity was associated with an annual income of less than CAD 20,000, an at-risk adiposity level (Nunavut) [53], limited formal education, living with three or more family members, unemployment, having few hunters or fishers in the family (Northern Quebec) [57], being women, and relying on store-bought foods [64]. The majority of community members in Nunavut and NWT utilizing community food programmes (e.g., food banks) did not have enough food in the past year and coped by eating less preferred food, reducing food consumption, and selling belongings for money to purchase food [58,59]. At the household level, food insecurity was more common in Inuit households (45%) than non-Inuit (4%) in Nunavut [51]. The prevalence of severe food insecurity was higher in Inuit households (27%) compared with First Nations households (17%), although overall food insecurity was more common in First Nations households (70% compared with 63%) [53,55]. Household food insecurity was associated with the family member in charge of food preparation being 40 years old or younger, unemployment [51], suboptimal housing conditions, the absence of an active hunter in the family in Nunavut [53], and the presence of children in Nunavut and northeastern Ontario [52,55]. In Nunavut, 69.6% of households with children aged 3–5 years experienced moderate or severe food insecurity [61]. Food insecurity remained concerning in Arctic Canada despite national efforts. In 2010, before the launch of the Nutrition North Canada programme, the rate of food insecurity in Nunavut was 33.1%; it increased to 46.6% in 2014 after the full implementation of the programme [63].

Food accessibility was subcategorized into purchase/cost/affordability and social network/food sharing. Although an individual’s ability to address food insecurity varied by region [67], high food costs and limited food options were common challenges [68,70]. With traditional food supplies decreasing, market foods were considered alternative sources, but they were often unaffordable or of low quality and nutritional value for people living in Yukon, northeastern Ontario, and NWT [50,55,59]. Remoteness resulted in long transportation times, high food costs, limited variety [68], and compromised quality in Nunavut [68], particularly with perishable foods in Yukon and NWT [50,59]. Market foods were also two to three times more expensive in the northern than in the southern regions of Canada [65]. Traditional/country foods were reported to be affordable and easily accessible by Dene/Métis women, while Inuit women reported otherwise [66]. Difficulties accessing markets and traditional/country foods increased among low-income individuals or families [58,65,67]. School snack/breakfast programmes were reported to increase students’ access to healthy food, although they may be improved by increasing food variety [68].

Food sharing within networks of families or community members was identified as a way to secure traditional/country foods in communities in northwestern Ontario [54] and Nunavut [75]. Having extended family, older or hunter household members, and someone to supply traditional/country food in the family network was advantageous for accessing traditional/country foods in Northern Quebec, Nunavut, and NWT [56,58,59,69,76]. Households headed by single young women were marginalized in family networks in Northern Quebec and NWT [56,69], although households headed by single older (over the age of 60 years) women were not, as the children were often old enough to provide traditional/country foods for household consumption [69]. Individuals or households with the ability to provide compensation or hunting supplies for food were more likely to receive traditional/country foods from others in Nunavut and Northern Quebec communities [50,56,65].

Traditional/country foods were less available in larger communities in Nunavut [65]. Reasons for the limited availability of traditional/country foods were categorized into the hunting ability [54,58,59,65,67,71,72] and environmental factors [50,54,66,71,73,74].

Hunting became difficult for several reasons: knowledge related to harvesting and preparing traditional/country foods was being lost, particularly in younger generations in Nunavut [65]; formalized employment reduced the time available to hunt [65]; and costs of hunting supplies were perceived to be high [54,59,65,71,72,75]. Hunting was also not always successful, making hunting less economically efficient and enjoyable in northwestern Ontario [54]. Available supports, such as funding for hunting equipment, were inadequate in Nunavut [65]. Community food programmes, such as freezers to share hunted or harvested traditional food, can address economic barriers; still, there are issues around dependency and social exclusion, more so in northern Labrador [71].

Climate changes (e.g., increased rainfall, warmer temperatures) were observed across Arctic communities [71,73,74,75]. These provided access to lands that were historically inaccessible, increasing hunting grounds and vegetation growth (fattening caribou) [73], but also resulted in unstable and unsafe hunting conditions and changes in the health, abundance, behaviours, and migration patterns of animals [50,54,66,71,73,74,75]. Some Inuit women felt climate changes had less influence on food availability compared with other social and personal factors such as the high cost of living, unemployment, and substance use [76]. Environmental contamination was reported to affect animal health and the taste/texture of harvested food in NWT and Nunavut [54,73].

Healthcare access: A total of 26 papers addressed healthcare access through 3 areas of discussion: 14 studies discussed service providers [77,78,79,80,81,82,83,84,85,86,87,88,89,90], and 21 discussed healthcare service availability [77,79,80,81,83,84,85,87,88,91,92,93,94,95,96,97,98,99,100,101,102].

Strong patient–provider relationships were associated with positive health outcomes [77,83,88]. Factors that enhanced the relationships included the healthcare providers’ level of community involvement [78,87]; their ability to communicate clearly and respectfully, both verbally [83,87] and through body language [81]; and their recognition of the impact of SDHs on health [92]. However, service providers, particularly nurses from Southern Canada, reported feeling overworked and burnt out due to limited support [77], and they often felt unprepared for the cultural context and institutional procedures in the Arctic. High demand for providers, coupled with staffing shortages [79,80,81,82], has been shown to lead to provider burnout, high turnover rates [77,78,79,80,81,92], and barriers to effective training [77,79], all of which hinder patients’ access to culturally safe [77] and continuous care [92]. Understaffing forced healthcare providers to focus more on acute care than managing chronic diseases [79]. Physicians practicing in the northern territories of Yukon, NWT, and Nunavut identified several contributors to their burnout, including limited involvement in constructing healthcare policy, limited cultural safety, discontinuity of care, tasks outside their scope of practice, high turnover, and limited support [86]. However, building supportive relationships with colleagues and the community and spending time on the land have been shown to mitigate physician burnout [86]. In the Beaufort–Delta regions of NWT, family physicians with enhanced surgical skills performed 47.7% of all surgeries and endoscopic procedures, with caesarean sections, tubal ligations, dilation and curettage, herniorrhaphies, and appendectomies being the most common procedures [89], and this showed that surgeries can be accessed closer to home when physicians with enhanced surgical skills and specialists surgeons are working in networks.

The availability and accessibility of healthcare services were discussed in 13 articles [77,79,80,81,83,84,85,88,91,92,93,94,95], which addressed issues such as general accessibility, medical travel, virtual consultations, and services inclusive of Two-Spirit people, Lesbian, Gay, Bisexual, Transgender, Queer, Intersex, and other gender expressions and sexual orientations (2SLGBTQI+). Primary care nurses were generally more accessible than physicians due to the limited number of physicians available [91]. Physician services were primarily accessible through visiting physicians or by medical travel to regional centres [91]. Additionally, northern communities often faced limited access to services within the communities [87,88], with some services requiring travel [83]. For members of the 2SLGBTQI+ communities, the limited availability of inclusive care poses a significant barrier to accessing healthcare. Individuals reported that prevailing norms often devalued non-binary identities, and it was a challenge to receive 2SLGBTQI+-specific health information and services [85,93], particularly in small communities in NWT, where residents are familiar with one another and maintaining confidentiality is difficult [94].

Medical travel is a key part of healthcare in the Arctic. A 2010 study in Nunavut, NWT, northern Labrador, and Northern Quebec reported that while 94% of non-Indigenous adults lived within a linear distance of 50 km from a hospital, 50% of Inuit adults lived more than 400 km from a hospital and were required to travel to access critical healthcare services [91]. Time spent at each facility differed according to the treatment required, from an average of nine days in Edmonton [96] to over a month in Ontario among Inuit cancer patients and escorts from Nunavut [100]. The major reasons for medical travel and hospitalization in Manitoba for Inuit in the Kivalliq region of Nunavut were pregnancy and birth and respiratory diseases [102]. From 2011 to 2016, NWT, and Nunavut averaged 23,012 and 21,578 annual medical trips, respectively, and spent 49% (CAD 9.5 million) and 35% (CAD 24.8 million) of total medical travel costs on emergency medevacs (6% and 9% of all medical travel, respectively), respectively [97]. Patients in Yukon and the central Canadian Arctic faced costs associated with childcare, telephone calls, and travel for partners and the lost wages associated with taking time off work [83,98]. Medical travel presents numerous challenges to patients in Nunavut, NWT, and northern Ontario, including making arrangements for family [83,96,98,100], communication [96,98,100], limited availability of medical equipment [77,99], logistical complexities [96,100], impersonal policies [96,98,100], the effects of geographical remoteness and adverse weather conditions [77,100], and the experience of navigating unfamiliar environments during care [96,98,100]. Patients felt overwhelmed by strangers in shared residences, unfamiliar weather in southern Canada, and unfamiliar food [96,100]. One study examined virtual consultations as an alternative to medical travel and estimated that virtual consultations saved 58 face-to-face referrals between August 2014 and April 2016, saving a total cost of CAD 180,553, or CAD 1101 per session [95]. The utilization of technology, including robots, to provide more physician care in northern Newfoundland and Labrador communities was suggested as a feasible and cost-effective option to improve healthcare access [101].

Mental health: Twenty-two articles focussed on mental health, addressing five major factors of mental health. Five discussed alcohol and substance use [103,104,105,106,107], three discussed the use of mental health-related services [108,109,110], and several discussed factors affecting mental health: family and support networks [108,109,111,112,113,114,115], gender [105,108,116,117,118,119,120,121], and seasonality and climate change [108,122,123,124].

High levels of suicide in Nunavut could create a “social logic” to suicide, which could be internalized by the community and repeated [113]. A history of substance use and having a parent with an alcohol or drug addiction were reported as factors related to suicide attempts in Northern Quebec [103]. Alcohol use was reported among 60% of pregnant Inuit respondents in Northern Quebec [104]. Binge drinking was identified as a major public health concern in some communities in Nunavut and Northern Quebec [105,106].

Physicians in Nunavut were reported as the primary source of psychiatric consultations [108], although about two-thirds of psychiatric care clients in Iqaluit reported no previous professional psychiatric support [108], which aligns with the aforementioned limited number of physicians. Mental health consultations were more frequently used among people who attempted suicide compared with people who died by suicide or people who never attempted suicide in Nunavut [109]. Of all mental health consultations provided in Manitoba, 27–35% accounted for Inuit who had relocated to Manitoba, and 18–30% accounted for Inuit who resided in Nunavut but accessed consultations in Manitoba, suggesting underserved needs for mental health consultations for Inuit in Nunavut [110].

Research on family and support networks produced mixed findings. Some studies found that families and friends positively influenced mental health [109,111,112,115], and support networks helped individuals feel engaged with and connected to the community [112,114]. Family and social support could also adversely affect mental health; of Inuit receiving psychiatric consultation, 35% reported family conflict/stress, 26% marital and relationship stress, and 26% family member abuse [108].

Gender was described as a male–female dichotomy, and reporting on 2SLGBTQI+ persons was scarce [105,108,116,117,118,119,121]. Although rates varied by community and time, suicidality was more common among men [117,118]. Male Inuit youth were 17% more likely than female youth to have previously attempted suicide [117]. Women (*n* = 1674) were more likely than men (*n* = 1184) to call a crisis line [119]. A study among adolescents in NWT found the rates of severe types of depression were twice as high among cisgender adolescents than 2SLGBTQI+ adolescents [120].

Studies found seasonal variations in mental health. Winter months (October–March) accounted for around three-quarters of the total psychiatric consultation referrals for Inuit clients living in Nunavut [108]. Seasonal mood changes, including seasonal affective disorder, appeared to be more frequent among Inuit in the Arctic than in other settings [122]. In the context of climate change, warmer temperatures affected the quantity and quality of snow and sea ice in the winter, preventing Inuit in northern Newfoundland and Labrador from spending time on the land, potentially contributing to mental health-related clinic visits [123] and feeling depressed [115,124].

Socioeconomic status (SES): Nine studies pertained to SES [125,126,127,128,129,130,131,132,133]. Income, employment [125,126,127,128,129,130,131], and educational attainment [125,127,128,132,133] were coded under SES. Within Arctic communities, these indicators were associated with health behaviours [125,128,131], including eating patterns and safe sex practices [125,128,131], and health outcomes, including chronic conditions [126,127,130,132,133]. Individuals with low SES disproportionately experienced challenges related to other SDHs, such as food and housing insecurity [129,133].

Housing: In the eight studies that examined housing environments, three main themes were apparent: two studies investigated contamination of houses [134,135], three investigated overcrowding [136,137,138], and four examined water [138,139,140,141]. In Whitehorse, Yukon, 34.9% of surveyed houses had a concentration of radon, which can cause lung cancer, higher than the Canadian guideline, and the estimated average annual radon dose from inhalation among adults in some subdivisions was as high as 39 times the world average [135]. Thirteen percent of houses surveyed in Nunavut had low ventilation, increased CO_2_ levels, and poor air quality, which were associated with illnesses including respiratory tract infections [134]. Poor air quality was in part due to overcrowding [134]. Studies found that a high prevalence of overcrowding also led to food insecurity, increased fires, and increased health centre visits [136,137,138]. Studies also showed many distribution challenges and delays with household water supplies [138,139,140] and concerns over the quantity and quality of water [141]. One study reported over half of the households in one Nunavut community were without water at least once every two to four weeks [139]. Community members coped with delays by personally retrieving water, sharing water among families and neighbours, and changing activities [138,139]. In an Inuit community in Northern Quebec, 33% reported having experienced a water shortage during the past week, with coping strategies including reducing the frequency of laundry and length of showers and re-using used water to wash hands and take baths [141].

Cultural continuity: Cultural continuity refers to maintaining one’s traditional language and culture and cultivating cultural identity [27]. Seven studies addressed six aspects of cultural continuity: one discussed holism [142], four discussed relationships [142,143,144,145], four discussed community [142,144,145,146] and cultural responsiveness and knowledge [142,143,145,147], three discussed nourishment [142,144,145], and three discussed language [144,147,148].

Holism involves viewing a person in relation to lands, traditions, homes, values, roles, and responsibilities in the world and recognizes the interconnections between the physical, mental, emotional, and spiritual aspects of a person’s health [142]. For example, a land-based mental health programme could use a camp setting to honour the importance of one’s connections to land and family in the healing journey [142].

Relationships refer to connections between individuals and communities and related aspects such as respect [142,145] and communication [143]. Close relationships with family were associated with well-being, health, and healing [143]. Changes in childbearing/parenting practices and segregation of generations contributed to cultural discontinuity and confusion in people’s cultural identity [143]. Building relationships with other community members through community events was related to good health [144]. Although modern technologies such as telephone and radio allow for different forms of communication, face-to-face communication was preferred and resulted in positive emotions, well-being, and healing [143].

Community members maintain a sense of community by sharing histories, cultures, perceptions of health, experiences and interactions within healthcare systems, and insights regarding community health needs [142]. Members of northern remote communities highlighted a need for northern-based resources reflecting local cultures, experiences, and stories in risk management messaging [146]. In Inuit self-governed regions in NWT, Nunavut, Northern Quebec, and northern Labrador, satisfaction with local Inuit governments was significantly associated with positively self-rated health [144].

Cultural responsiveness and knowledge were identified as important to health [143,145], but they are, at times, overlooked by non-Inuit care providers [147]. Inuit nurses and nursing students could help alleviate cultural tensions in the healthcare system through “double culturedness”, which enables Inuit nurses and nursing students to learn, teach, communicate, and interact in both Inuit and Western ways [147]. The exchange of knowledge between healthcare providers and communities “that incorporates a holistic view of the interconnectedness of traditional spiritual and environmental laws and an understanding of the natural order” (p. 10) could promote cultural sensitivity in healthcare [142].

Achieving nourishment through traditional foods, respecting resources, and sharing with others are important cultural aspects of Canadian Arctic Indigenous communities [142]. Harvesting, consuming, and feeling satisfied with traditional foods were positively associated with good health [144]. Spiritual and emotional nourishment through sewing was found to promote health among Inuit women [145].

Having access to services in local Indigenous languages has been associated with positively reported self-rated health [144]. Inuit nursing students recognized a disconnect between healthcare education and Inuit languages and reported the need for an interpreter to properly care for Inuit patients, as the students had not learned medical vocabulary in Inuktitut [147]. Healthcare professionals practicing end-of-life care reported heavily relying on interpreters when discussing options or providing support to patients and families in Northern Quebec [148]. Despite the necessity of and high demand for interpreters, low job retention and absenteeism were common among interpreters due to emotional stress, limited formal training/resources, lack of recognition, and ethical and cultural dilemmas [148].

Community infrastructure: Three papers explored the broader infrastructure within remote communities [149,150,151], discussed as both physical (e.g., steep stairways to homes and buildings) and communication resources [150,151]. Elders in a Nunavut community relied on a community radio station for news about the community and services; however, the station had been down for several months [150]. Physical community infrastructure in Nunavut, including roads and lighting, could improve walkability and help reduce body mass index (BMI) [149].

## 4. Discussion

### 4.1. Summary of Main Results

This scoping review summarized the current scientific literature regarding selected pandemics (COVID-19, H1N1 influenza, and TB) and SDHs (healthcare, food insecurity, mental health, SES, cultural continuity, housing, and community infrastructure) in Canadian Arctic Indigenous communities. SDHs in Arctic communities create unique health environments that may be more susceptible to the impact of pandemics and must be considered in future public health approaches [152]. Only three studies explored the interface of the selected infectious diseases and SDHs in the Canadian Arctic [30,31,45], guaranteeing evidence to be deduced from other settings and further research.

### 4.2. Interpretation and Implications of Results

Indigenous communities across North America have historically been disproportionately affected by infectious diseases like TB [153], the 1918–1919 influenza pandemic [15], and the 2009–2010 H1N1 pandemic [17,154,155]. The literature indicates that SDHs play a role in the spread and severity of infectious diseases [156]. Restrictions put in place to prevent COVID-19 may present greater challenges to Indigenous communities than non-Indigenous communities in the Arctic due to the SDHs identified in this scoping review, including socioeconomic and geographic challenges, food insecurity, water insecurity, housing barriers, and a culture of holism and interconnectedness that includes traditional gatherings [157]. Indigenous sovereignty, traditional knowledge, and community resilience may protect Indigenous communities from the negative impacts of a pandemic and are critical to include in pandemic prevention and management [158,159].

For the SDH of healthcare access, medical travel was identified as an important mode of accessing healthcare for Arctic communities. Pandemics and related public health measures (e.g., travel bans, reduction in non-urgent care) can complicate medical travels [160,161]. In other settings, delayed care resulted in reduced cancer survival due to delayed diagnoses [162] and permanent vision impairment or blindness due to postponed eye care [163]. In southern Canada, COVID-19 evoked fear, uncertainty, frustration, anger, helplessness, and sadness among nurses [164]. This review highlighted that prior to the COVID-19 pandemic, healthcare providers in the Arctic experienced high levels of stress and burnout, which has serious implications for the current accessibility of care in Arctic communities. Research is needed to understand medical travel experiences during the COVID-19 pandemic and the short-, medium-, and long-term effects of probable delayed access to care as well as to expand the understanding of heavy workload and stress on healthcare providers in Arctic communities.

Mental health challenges among Indigenous communities are generally understood to be rooted in a history of and ongoing colonialism, forced assimilation, and the resulting intergenerational trauma [165,166]. Pandemics and public health measures increased anxiety, depression, stress, insomnia, compulsive behaviours, phobias, and life dissatisfaction [167]. This scoping review identified that mental health among residents of Canadian Arctic communities may be influenced by seasonality, which also may contribute to seasonal variation in the transmission of viral infections, particularly at higher latitudes [168]. During the COVID-19 pandemic, it was suggested that Indigenous community members go on the land and engage in traditional activities to cope [169,170]. This recommendation may help Indigenous community members cope with mental health challenges and strengthen feelings of cultural continuity while reducing the chance of transmission of infections.

Contemporary health perspectives require that we understand how centuries of colonialism and assimilation situated the ongoing health experiences, outcomes, and inequities of Indigenous communities [18,171]. This scoping review found some literature on cultural continuity. Many Indigenous community members experience barriers to traditional foods, traditional knowledge, languages, lands, holistic views of health, and self-determination [172,173,174,175]. Public health measures during pandemics may impact Indigenous cultural practices such as gatherings, which critically strengthen community and family relationships and allow for holistic traditional nourishment through the sharing of foods. This review also highlighted how culturally safe care can improve the general health and healthcare utilization for chronic condition management [96,144,176,177,178,179]. There is a need for acknowledging the importance of and minimizing impacts on cultural continuity for effective public health measure implementation and acceptance within communities during a pandemic.

In Canada, compared with the national rate (8.8% in 2017–2018), food insecurity rates have historically been higher in the northern territories (49.4% in Nunavut, 15.9% in NWT, and 12.6% in Yukon in 2017–2018) [180]. The findings of this review support this trend, describing higher food costs and limited food availability in the Arctic. During the COVID-19 pandemic, 14.6% of Canadian households in southern provinces experienced food insecurity [181]. Although evidence is lacking, one can anticipate that communities in Arctic Canada needed to survive a higher rate of food insecurity considering the impact of the pandemic on food production, distribution, and transportation to remote regions [182,183,184,185]. Moderate or severe food insecurity may have significant biological implications, including malnutrition, suboptimal immunity, and increased susceptibility to infections. Inuit traditionally had diets rich in vitamins A and D [186,187], but have recently undergone a dietary transition, reducing Inuit consumption of traditional/country foods and thus the intake of nutrients important for immunity [186,187,188,189,190]. Individuals experiencing food insecurity may prioritize accessing food programmes over following public health guidelines such as avoiding crowds [191].

Housing and infrastructure play an important role in containing and managing infectious disease outbreaks at the individual, household/family, and community levels. Overcrowding and poor housing quality could increase the spread and impact of infectious diseases [192]. This review identified these housing issues in Arctic communities in addition to limited water supplies, which can impact adherence to certain public health measures such as frequent handwashing. Other aspects of the living environment and infrastructure, such as roads, public lighting, and phone and internet access, could affect access to information and services during the pandemic, and they merit investigation.

Despite the deficit in exploring the interrelation with SDHs, studies regarding previous TB and H1N1 outbreaks have made some suggestions that may be relevant to COVID-19 prevention strategies in the Canadian Arctic. Communication of public health information was made to the public directly from the territorial governments [193]. It was necessary to deliver clear public health messages, implement measures that were relevant to the local context, and improve public adherence to public health measures. While the distribution of H1N1 vaccines was challenging in the Canadian Arctic [43], Indigenous communities, particularly in remote and isolated regions, were prioritized in the COVID-19 vaccine rollout [194]. Further studies about the impacts of limited resources and the effectiveness of culturally sensitive interventions in the COVID-19 context are required.

### 4.3. Shortcomings and Strengths

Although the authors believe this review sets the stage for future action and research regarding infectious diseases and SDHs in Canadian Arctic Indigenous contexts, there are several limitations. Firstly, the literature search may not have identified all the existing literature, as it was limited to the selected search terms, the databases available through the University of Alberta, and studies published in English and French. However, we consulted two publications regarding SDHs and carefully deliberated the search terms. As well, English and French are Canada’s official languages, and the authors believe searching for articles in other languages would not have yielded different results. Secondly, some SDHs that may have implications for COVID-19 were excluded from this review. For instance, the SDH of the environment and remoteness, unique in the Arctic setting, were presented in relation to other SDHs; however, the authors believe this review provides context to these excluded SDHs. The SDH of discrimination was also omitted. Ongoing systematic racism rooted in the history of colonialism shapes many other SDHs among Indigenous peoples in Canada, and the authors found that discrimination was tightly interwoven with other SDHs in the literature. It is critical to acknowledge that the legacy of colonialism and systematic and systemic discrimination continue to negatively shape SDHs, and subsequently, adverse infectious disease experiences and outcomes in Indigenous communities. A review regarding the impacts of discrimination on the prevention and management of infectious diseases among Indigenous communities in Canada remains necessary. Lastly, this review focussed on the Canadian Arctic. Prior to the COVID-19 pandemic, the common concern was that Arctic Indigenous communities worldwide experience more adverse health outcomes than non-Indigenous populations [195]. Health inequalities in Arctic Indigenous community members, including higher rates of binge drinking, smoking, overweight/obesity, suicide mortality, and a shorter life expectancy in Greenland, the USA, and Canada; lower social and ethnic health indicators and lower dental health index in Arctic Russia; and lower confidence in and satisfaction with primary healthcare services in Norway [195] are likely to have worsened during the pandemic. However, Arctic nations are heterogeneous and have unique health settings and experiences of SDHs. Given the global impact of COVID-19, further review and discussion concerning the implications of SDHs on the COVID-19 pandemic in other Arctic countries is needed. This review discusses the implications of the findings and the need for further studies regarding SDHs on infectious diseases in Canadian Arctic Indigenous communities (Table 1).

## 5. Conclusions

Previous pandemics inform strategies mitigating the impact of current and future public health emergencies such as the COVID-19 pandemic. SDHs present many unique challenges for health and the control and management of infectious disease outbreaks in Canadian Arctic Indigenous communities. Considering the COVID-19 pandemic, there is an urgent need to understand the experiences and consequences of SDHs in Arctic Indigenous communities and to document the response strategies that have been successful or need improvement to inform future pandemic response policies.

## Figures and Tables

**Table 1:** Recommendations for future directions for research and public health in Arctic Canada.

Further studies
Explore the impact of COVID-19 on medical travel and the short-, medium-, and long-term effects of delayed access to care. Understand the impact of pandemics on healthcare providers serving Indigenous communities in Arctic Canada. Examine the spread of respiratory viral infections and mental health status throughout the seasons. Explore community members’ experiences with and perceptions of public health messages and overall health system responses during the pandemic. Understand the living environment and infrastructure, such as roads, public lighting, communication (phone, internet access) and impact on access to information and services during pandemics.
Public health policies and programmes
Design, implement, and evaluate targeted, evidence-based, community-informed, and community-led responses to pandemics, including culturally safe public health messaging and information sharing. Support healthcare providers’ mental health and enhance the ability to provide quality care while preventing burnout.

## Supplementary Materials

The following supporting information can be downloaded at: https://www.mdpi.com/article/10.3390/ijerph22010001/s1, Table S1: PRISMA-ScR checklist Table S2: Search strategy, Table S3: Characteristics and major findings of selected articles, Table S4: Themes and subthemes under each category and selected quotes from the studies, Table S5: Critical appraisal.

## References

[1] Hansen T. How COVID-19 Could Destroy Indigenous Communities. https://www.bbc.com/future/article/20200727-how-covid-19-could-destroy-indigenous-communities.

[2] Power T., Wilson D., Best O., Brockie T., Bourque Bearskin L., Millender E., Lowe J. (2020). COVID-19 and Indigenous Peoples: An imperative for action. J. Clin. Nurs..

[3] Economic Commission for Latin America and the Caribbean (ECLAC) and others (2021). The Impact of COVID-19 on Indigenous Peoples in Latin America (Abya Yala): Between Invisibility and Collective Resistance”, Project Documents.

[4] Petrov A.N., Welford M., Golosov N., DeGroote J., Degai T., Savelyev A. (2020). Spatiotemporal dynamics of the COVID-19 pandemic in the arctic: Early data and emerging trends. Int. J. Circumpolar Health.

[5] Leneisja Jungsberg E.T., Heleniak T., Wang S., Ramage J., Roto J. (2019). Atlas of Population, Society and Economy in the Arctic.

[6] Statistics Canada Indigenous Population Continues to Grow and Is Much Younger than the Non-Indigenous Population, Although the Pace of Growth Has Slowed. https://www150.statcan.gc.ca/n1/en/daily-quotidien/220921/dq220921a-eng.pdf?st=HxiuO8Q7.

[7] Young T.K., Bjerregaard P. (2008). Health Transitions in Arctic Populations.

[8] Bjerregaard P., Young T.K., Dewailly E., Ebbesson S.O. (2004). Indigenous health in the Arctic: An overview of the circumpolar Inuit population. Scand. J. Public Health.

[9] Langlois A. Canada’s Arctic Tundra. https://www.hww.ca/assets/pdfs/factsheets/tundra-en.pdf.

[10] McNeill W.H. (1998). Plagues and Peoples.

[11] Bonesteel S., Anderson E. (2008). Canada’s Relationship with Inuit: A History of Policy and Program Development.

[12] Grant S. (2016). Errors Exposed: Inuit Relocation to the High Arctic, 1953–1960. Documents on Canada’s Artic Sovereignty and Security.

[13] Qikiqtani Inuit Association (2013). QTC Final Report: Achieving Saimaqatigiingniq.

[14] Vachon J., Gallant V., Siu W. (2018). Tuberculosis in Canada, 2016. Can. Commun. Dis. Rep..

[15] Mamelund S.-E., Sattenspiel L., Dimka J. (2013). Influenza-Associated Mortality during the 1918–1919 Influenza Pandemic in Alaska and Labrador: A Comparison. Soc. Sci. Hist..

[16] Morrison K.T., Buckeridge D.L., Yanyu X., Moghadas S.M. (2014). The impact of geographical location of residence on disease outcomes among Canadian First Nations populations during the 2009 influenza A(H1N1) pandemic. Health Place.

[17] Charania N.A., Tsuji L.J. (2011). The 2009 H1N1 pandemic response in remote First Nation communities of Subarctic Ontario: Barriers and improvements from a health care services perspective. Int. J. Circumpolar Health.

[18] Richardson L., Crawford A. (2020). COVID-19 and the decolonization of Indigenous public health. CMAJ.

[19] Kaspar V. (2014). The lifetime effect of residential school attendance on indigenous health status. Am. J. Public Health.

[20] Wilk P., Maltby A., Cooke M. (2017). Residential schools and the effects on Indigenous health and well-being in Canada-a scoping review. Public Health Rev..

[21] Chief Moon-Riley K., Copeland J.L., Metz G.A.S., Currie C.L. (2019). The biological impacts of Indigenous residential school attendance on the next generation. SSM-Popul. Health.

[22] Inuit Tapiriit Kanatami (2018). Inuit Statistical Profile 2018.

[23] Kim P.J. (2019). Social Determinants of Health Inequities in Indigenous Canadians Through a Life Course Approach to Colonialism and the Residential School System. Health Equity.

[24] Brakefield W.S., Olusanya O.A., White B., Shaban-Nejad A. (2022). Social Determinants and Indicators of COVID-19 Among Marginalized Communities: A Scientific Review and Call to Action for Pandemic Response and Recovery. Disaster Med. Public.

[25] Abrams E.M., Szefler S.J. (2020). COVID-19 and the impact of social determinants of health. Lancet Respir. Med..

[26] Czyzewski K. (2011). Colonialism as a Broader Social Determinant of Health. Int. Indig. Policy J..

[27] Inuit Tapiirit Kanatami (2014). Social Determinants of Inuit Health in Canada.

[28] Reading C., Wien F. (2009). Health Inequalities and Social Determinants of Aboriginal Peoples’ Health.

[29] Munn Z., Peters M.D.J., Stern C., Tufanaru C., McArthur A., Aromataris E. (2018). Systematic review or scoping review? Guidance for authors when choosing between a systematic or scoping review approach. BMC Med. Res. Methodol..

[30] Healey Akearok G.K., Rana Z. (2024). Community perspectives on COVID-19 outbreak and public health: Inuit positive protective pathways and lessons for Indigenous public health theory. Can. J. Public Health.

[31] Lavoie J.G., Clark W., McDonnell L., Nickel N., Dutton R., Kanayok J., Fowler-Woods M., Anawak J., Brown N., Voisey Clark G. (2023). Cross-jurisdictional pandemic management: Providers speaking on the experience of Nunavut Inuit accessing services in Manitoba during the COVID-19 pandemic. Int. J. Circumpolar Health.

[32] Levac D., Colquhoun H., O’Brien K.K. (2010). Scoping studies: Advancing the methodology. Implement. Sci..

[33] Tricco A.C., Lillie E., Zarin W., O’Brien K.K., Colquhoun H., Levac D., Moher D., Peters M.D.J., Horsley T., Weeks L. (2018). PRISMA Extension for Scoping Reviews (PRISMA-ScR): Checklist and Explanation. Ann. Intern. Med..

[34] Aromataris E., Pearson A. (2014). The systematic review: An overview. Am. J. Nurs..

[35] von Elm E., Altman D.G., Egger M., Pocock S.J., Gøtzsche P.C., Vandenbroucke J.P. (2007). The Strengthening the Reporting of Observational Studies in Epidemiology (STROBE) statement: Guidelines for reporting observational studies. Lancet.

[36] O’Brien B.C., Harris I.B., Beckman T.J., Reed D.A., Cook D.A. (2014). Standards for reporting qualitative research: A synthesis of recommendations. Acad. Med..

[37] Hong Q.N., Pluye P., Fàbregues S., Bartlett G., Boardman F., Cargo M., Dagenais P., Gagnon M.P., Griffiths F., Nicolau B. (2019). Improving the content validity of the mixed methods appraisal tool: A modified e-Delphi study. J. Clin. Epidemiol..

[38] Petrov A.N., Welford M., Golosov N., DeGroote J., Devlin M., Degai T., Savelyev A. (2021). The “second wave” of the COVID-19 pandemic in the Arctic: Regional and temporal dynamics. Int. J. Circumpolar Health.

[39] Tiwari S., Petrov A.N., Devlin M., Welford M., Golosov N., DeGroote J., Degai T., Ksenofontov S. (2022). The second year of pandemic in the Arctic: Examining spatiotemporal dynamics of the COVID-19 “Delta wave” in Arctic regions in 2021. Int. J. Circumpolar Health.

[40] Vilches T.N., Abdollahi E., Cipriano L.E., Haworth-Brockman M., Keynan Y., Sheffield H., Langley J.M., Moghadas S.M. (2022). Impact of non-pharmaceutical interventions and vaccination on COVID-19 outbreaks in Nunavut, Canada: A Canadian Immunization Research Network (CIRN) study. BMC Public Health.

[41] Banerji A., Panzov V., Young M., Robinson J., Lee B., Moraes T., Mamdani M., Giles B.L., Jiang D., Bisson D. (2016). Hospital admissions for lower respiratory tract infections among infants in the Canadian Arctic: A cohort study. CMAJ Open.

[42] Charania N.A., Tsuji L.J. (2011). Government bodies and their influence on the 2009 H1N1 health sector pandemic response in remote and isolated First Nation communities of sub-Arctic Ontario, Canada. Rural Remote Health.

[43] Charania N.A., Tsuji L.J. (2013). Assessing the effectiveness and feasibility of implementing mitigation measures for an influenza pandemic in remote and isolated First Nations communities: A qualitative community-based participatory research approach. Rural Remote Health.

[44] Charania N.A., Tsuji L.J. (2012). A community-based participatory approach and engagement process creates culturally appropriate and community informed pandemic plans after the 2009 H1N1 influenza pandemic: Remote and isolated First Nations communities of sub-arctic Ontario, Canada. BMC Public Health.

[45] Kilabuk E., Momoli F., Mallick R., Van Dyk D., Pease C., Zwerling A., Potvin S.E., Alvarez G.G. (2019). Social determinants of health among residential areas with a high tuberculosis incidence in a remote Inuit community. J. Epidemiol. Community Health.

[46] Pease C., Zwerling A., Mallick R., Patterson M., Demaio P., Finn S., Allen J., Van Dyk D., Alvarez G.G. (2019). The latent tuberculosis infection cascade of care in Iqaluit, Nunavut, 2012–2016. BMC Infect. Dis..

[47] Bourgeois A.C., Zulz T., Bruce M.G., Stenz F., Koch A., Parkinson A., Hennessy T., Cooper M., Newberry C., Randell E. (2018). Tuberculosis in the Circumpolar Region, 2006–2012. Int. J. Tuberc. Lung Dis..

[48] Alvarez G.G., Van Dyk D.D., Colquhoun H., Moreau K.A., Mulpuru S., Graham I.D. (2016). Developing and Field Testing a Community Based Youth Initiative to Increase Tuberculosis Awareness in Remote Arctic Inuit Communities. PLoS ONE.

[49] Alvarez G.G., Zwerling A.A., Duncan C., Pease C., Van Dyk D., Behr M.A., Lee R.S., Mulpuru S., Pakhale S., Cameron D.W. (2021). Molecular Epidemiology of Mycobacterium tuberculosis To Describe the Transmission Dynamics Among Inuit Residing in Iqaluit Nunavut Using Whole-Genome Sequencing. Clin. Infect. Dis..

[50] Douglas V., Chan H.M., Wesche S., Dickson C., Kassi N., Netro L., Williams M. (2014). Reconciling traditional knowledge, food security, and climate change: Experience from Old Crow, YT, Canada. Prog. Community Health Partnersh..

[51] Guo Y., Berrang-Ford L., Ford J., Lardeau M.P., Edge V., Patterson K., Team I.R., Harper S.L. (2015). Seasonal prevalence and determinants of food insecurity in Iqaluit, Nunavut. Int. J. Circumpolar Health.

[52] Huet C., Ford J.D., Edge V.L., Shirley J., King N., Team I.R., Harper S.L. (2017). Food insecurity and food consumption by season in households with children in an Arctic city: A cross-sectional study. BMC Public Health.

[53] Huet C., Rosol R., Egeland G.M. (2012). The prevalence of food insecurity is high and the diet quality poor in Inuit communities. J. Nutr..

[54] Skinner K., Hanning R.M., Desjardins E., Tsuji L.J. (2013). Giving voice to food insecurity in a remote indigenous community in subarctic Ontario, Canada: Traditional ways, ways to cope, ways forward. BMC Public Health.

[55] Skinner K., Hanning R.M., Tsuji L.J. (2014). Prevalence and severity of household food insecurity of First Nations people living in an on-reserve, sub-Arctic community within the Mushkegowuk Territory. Public Health Nutr..

[56] Ready E. (2018). Sharing-based social capital associated with harvest production and wealth in the Canadian Arctic. PLoS ONE.

[57] Teh L., Pirkle C., Furgal C., Fillion M., Lucas M. (2017). Psychometric validation of the household food insecurity access scale among Inuit pregnant women from Northern Quebec. PLoS ONE.

[58] Ford J., Lardeau M.P., Vanderbilt W. (2012). The characteristics and experience of community food program users in arctic Canada: A case study from Iqaluit, Nunavut. BMC Public Health.

[59] Ford J.D., Lardeau M.P., Blackett H., Chatwood S., Kurszewski D. (2013). Community food program use in Inuvik, Northwest Territories. BMC Public Health.

[60] Logie C.H., Lys C., Sokolovic N., Malama K., Mackay K.I., McNamee C., Lad A., Kanbari A. (2024). Examining Pathways from Food Insecurity to Safer Sex Efficacy Among Northern and Indigenous Adolescents in the Northwest Territories, Canada. Int. J. Behav. Med..

[61] Egeland G.M., Pacey A., Cao Z., Sobol I. (2010). Food insecurity among Inuit preschoolers: Nunavut Inuit Child Health Survey, 2007–2008. CMAJ.

[62] Egeland G.M., Johnson-Down L., Cao Z.R., Sheikh N., Weiler H. (2011). Food insecurity and nutrition transition combine to affect nutrient intakes in Canadian arctic communities. J. Nutr..

[63] St-Germain A.F., Galloway T., Tarasuk V. (2019). Food insecurity in Nunavut following the introduction of Nutrition North Canada. CMAJ.

[64] Ford J., Berrang-Ford L. (2009). Food security in Igloolik, Nunavut: An exploratory study. Polar Rec..

[65] Chan H.M., Fediuk K., Hamilton S., Rostas L., Caughey A., Kuhnlein H., Egeland G., Loring E. (2006). Food security in Nunavut, Canada: Barriers and recommendations. Int. J. Circumpolar Health.

[66] Lambden J., Receveur O., Kuhnlein H.V. (2007). Traditional food attributes must be included in studies of food security in the Canadian Arctic. Int. J. Circumpolar Health.

[67] Lambden J., Receveur O., Marshall J., Kuhnlein H.V. (2006). Traditional and market food access in Arctic Canada is affected by economic factors. Int. J. Circumpolar Health.

[68] Skinner K., Hanning R.M., Tsuji L.J. (2006). Barriers and supports for healthy eating and physical activity for First Nation youths in northern Canada. Int. J. Circumpolar Health.

[69] Collings P., Marten M.G., Pearce T., Young A.G. (2016). Country food sharing networks, household structure, and implications for understanding food insecurity in Arctic Canada. Ecol. Food Nutr..

[70] Blom C.D.B., Steegeman P., Voss C., Sonneveld B. (2022). Food in the cold: Exploring food security and sovereignty in Whitehorse, Yukon. Int. J. Circumpolar Health.

[71] Organ J., Castleden H., Furgal C., Sheldon T., Hart C. (2014). Contemporary programs in support of traditional ways: Inuit perspectives on community freezers as a mechanism to alleviate pressures of wild food access in Nain, Nunatsiavut. Health Place.

[72] Spiegelaar N.F., Tsuji L.J. (2013). Impact of Euro-Canadian agrarian practices: In search of sustainable import-substitution strategies to enhance food security in subarctic Ontario, Canada. Rural Remote Health.

[73] Nancarrow T.L., Chan H.M. (2010). Observations of environmental changes and potential dietary impacts in two communities in Nunavut, Canada. Rural Remote Health.

[74] Wesche S.D., Chan H.M. (2010). Adapting to the impacts of climate change on food security among Inuit in the Western Canadian Arctic. Ecohealth.

[75] Gilbert S.Z., Walsh D.E., Levy S.N., Maksagak B., Milton M.I., Ford J.D., Hawley N.L., Dubrow R. (2021). Determinants, effects, and coping strategies for low-yield periods of harvest: A qualitative study in two communities in Nunavut, Canada. Food Secur..

[76] Beaumier M.C., Ford J.D., Tagalik S. (2015). The food security of Inuit women in Arviat, Nunavut: The role of socio-economic factors and climate change. Polar Rec..

[77] McDonnell L., Lavoie J.G., Healy G., Wong S., Goulet S., Clark W. (2019). Non-clinical determinants of Medevacs in Nunavut: Perspectives from northern health service providers and decision-makers. Int. J. Circumpolar Health.

[78] Tarlier D.S., Browne A.J., Johnson J. (2007). The influence of geographical and social distance on nursing practice and continuity of care in a remote First Nations community. Can. J. Nurs. Res..

[79] Bhattacharyya O.K., Estey E.A., Rasooly I.R., Harris S., Zwarenstein M., Barnsley J. (2011). Providers’ perceptions of barriers to the management of type 2 diabetes in remote Aboriginal settings. Int. J. Circumpolar Health.

[80] Oosterveer T.M., Young T.K. (2015). Primary health care accessibility challenges in remote indigenous communities in Canada’s North. Int. J. Circumpolar Health.

[81] Romain S.J., Kohler J.C., Young K. (2015). Policy versus practice: A community-based qualitative study of the realities of pharmacy services in Nunavut, Canada. J. Pharm. Policy Pract..

[82] Young T.K., Fedkina N., Chatwood S., Bjerregaard P. (2018). Comparing health care workforce in circumpolar regions: Patterns, trends and challenges. Int. J. Circumpolar Health.

[83] Galloway T., Horlick S., Cherba M., Cole M., Woodgate R.L., Healey Akearok G. (2020). Perspectives of Nunavut patients and families on their cancer and end of life care experiences. Int. J. Circumpolar Health.

[84] Bird S.M., Wiles J.L., Okalik L., Kilabuk J., Egeland G.M. (2008). Living with diabetes on Baffin Island: Inuit storytellers share their experiences. Can. J. Public Health.

[85] Logie C.H., Lys C.L., Dias L., Schott N., Zouboules M.R., MacNeill N., Mackay K. (2019). “Automatic assumption of your gender, sexuality and sexual practices is also discrimination”: Exploring sexual healthcare experiences and recommendations among sexually and gender diverse persons in Arctic Canada. Health Soc. Care Community.

[86] Hansen N., Jensen K., MacNiven I., Pollock N., D’Hont T., Chatwood S. (2021). Exploring the impact of rural health system factors on physician burnout: A mixed-methods study in Northern Canada. BMC Health Serv. Res..

[87] Fraser S.L., Nadeau L. (2015). Experience and representations of health and social services in a community of Nunavik. Contemp. Nurse.

[88] Cooper R., Pollock N.J., Affleck Z., Bain L., Hansen N.L., Robertson K., Chatwood S. (2021). Patient healthcare experiences in the Northwest Territories, Canada: An analysis of news media articles. Int. J. Circumpolar Health.

[89] Falk R., Topstad D. (2023). Surgery in the western Canadian Arctic: The relative impact of family physicians with enhanced surgical skills working collaboratively with specialist surgeons. Can. J. Rural Med..

[90] Falk R., Topstad D., Lee L. (2022). Surgical Task-Sharing in the Western Canadian Arctic: A Networked Model Between Family Physicians with Enhanced Surgical Skills and Specialist Surgeons. World J. Surg..

[91] McDonald J.T., Trenholm R. (2010). Cancer-related health behaviours and health service use among Inuit and other residents of Canada’s north. Soc. Sci. Med..

[92] Bhattacharyya O.K., Rasooly I.R., Naqshbandi M., Estey E.A., Esler J., Toth E., Macaulay A.C., Harris S.B. (2011). Challenges to the provision of diabetes care in first nations communities: Results from a national survey of healthcare providers in Canada. BMC Health Serv. Res..

[93] Logie C.H., Lys C. (2015). The process of developing a community-based research agenda with lesbian, gay, bisexual, transgender and queer youth in the Northwest Territories, Canada. Int. J. Circumpolar Health.

[94] Logie C.H., Lys C.L., Schott N., Dias L., Zouboules M.R., Mackay K. (2018). ‘In the North you can’t be openly gay’: Contextualising sexual practices among sexually and gender diverse persons in Northern Canada. Glob. Public Health.

[95] Liddy C., McKellips F., Armstrong C.D., Afkham A., Fraser-Roberts L., Keely E. (2017). Improving access to specialists in remote communities: A cross-sectional study and cost analysis of the use of eConsult in Nunavut. Int. J. Circumpolar Health.

[96] Kerber K., Kolahdooz F., Otway M., Laboucan M., Jang S.L., Lawrence S., Aronyk S., Quinn M., Irlbacher-Fox S., Milligan C. (2019). Opportunities for improving patient experiences among medical travellers from Canada’s far north: A mixed-methods study. BMJ Open.

[97] Young T.K., Tabish T., Young S.K., Healey G. (2019). Patient transportation in Canada’s northern territories: Patterns, costs and providers’ perspectives. Rural Remote Health.

[98] Chamberlain M., Barclay K. (2000). Psychosocial costs of transferring indigenous women from their community for birth. Midwifery.

[99] Sheffield H.A., Sheffield C.A. (2019). Nasal CPAP on paediatric air transport in the Canadian Arctic: A case series. Paediatr. Child. Health.

[100] Jull J., Sheppard A.J., Hizaka A., Inuit Medical Interpreter T., Barton G., Doering P., Dorschner D., Edgecombe N., Ellis M., Graham I.D. (2021). Experiences of Inuit in Canada who travel from remote settings for cancer care and impacts on decision making. BMC Health Serv. Res..

[101] Mendez I., Jong M., Keays-White D., Turner G. (2013). The use of remote presence for health care delivery in a northern Inuit community: A feasibility study. Int. J. Circumpolar Health.

[102] Clark W., Lavoie J.G., McDonnell L., Nickel N., Anawak J., Brown L., Clark G., Evaluardjuk-Palmer M., Ford F., Dutton R. (2022). Trends in Inuit health services utilisation in Manitoba: Findings from the Qanuinngitsiarutiksait study. Int. J. Circumpolar Health.

[103] Kirmayer L.J., Boothroyd L.J., Hodgins S. (1998). Attempted suicide among Inuit youth: Psychosocial correlates and implications for prevention. Can. J. Psychiatry.

[104] Fortin M., Muckle G., Anassour-Laouan-Sidi E., Jacobson S.W., Jacobson J.L., Belanger R.E. (2016). Trajectories of Alcohol Use and Binge Drinking Among Pregnant Inuit Women. Alcohol Alcohol..

[105] Decaluwe B., Fortin M., Moisan C., Muckle G., Belanger R.E. (2019). Drinking motives supporting binge drinking of Inuit adolescents. Can. J. Public Health.

[106] Haggarty J.M., Cernovsky Z., Bedard M., Merskey H. (2008). Suicidality in a sample of Arctic households. Suicide Life Threat. Behav..

[107] Wood D.S. (2011). Alcohol controls and violence in Nunavut: A comparison of wet and dry communities. Int. J. Circumpolar Health.

[108] Law S.F., Hutton E.M. (2007). Community Psychiatry in the Canadian Arctic—Reflections From A 1-year Continuous Consultation Series in Iqaluit, Nunavut. Can. J. Community Ment. Health.

[109] Beaudoin V., Seguin M., Chawky N., Affleck W., Chachamovich E., Turecki G. (2018). Protective Factors in the Inuit Population of Nunavut: A Comparative Study of People Who Died by Suicide, People Who Attempted Suicide, and People Who Never Attempted Suicide. Int. J. Environ. Res. Public Health.

[110] Lavoie J.G., Clark W., McDonnell L., Toor J., Nickel N., Anang P., Kusugak M.A., Evaluardjuk-Palmer T., Brown N., Voisey Clark G. (2024). Inuit mental health service utilisation in Manitoba: Results from the qanuinngitsiarutiksait study. Int. J. Circumpolar Health.

[111] Collins P.Y., Delgado R.A., Apok C., Baez L., Bjerregaard P., Chatwood S., Chipp C., Crawford A., Crosby A., Dillard D. (2019). RISING SUN: Prioritized Outcomes for Suicide Prevention in the Arctic. Psychiatr. Serv..

[112] Petrasek MacDonald J., Cunsolo Willox A., Ford J.D., Shiwak I., Wood M., Team I., Rigolet Inuit Community G. (2015). Protective factors for mental health and well-being in a changing climate: Perspectives from Inuit youth in Nunatsiavut, Labrador. Soc. Sci. Med..

[113] Kral M.J. (2013). “The weight on our shoulders is too much, and we are falling”: Suicide among Inuit male youth in Nunavut, Canada. Med. Anthropol. Q..

[114] O’Neill L., George S., Koehn C., Shepard B. (2013). Informal and formal mental health: Preliminary qualitative findings. Int. J. Circumpolar Health.

[115] Poliakova N., Riva M., Fletcher C., Desrochers-Couture M., Courtemanche Y., Moisan C., Fraser S., Pepin C., Belanger R.E., Muckle G. (2024). Sociocultural factors in relation to mental health within the Inuit population of Nunavik. Can. J. Public Health.

[116] Fortin M., Belanger R.E., Boucher O., Muckle G. (2015). Temporal trends of alcohol and drug use among Inuit of Northern Quebec, Canada. Int. J. Circumpolar Health.

[117] Kirmayer L.J., Malus M., Boothroyd L.J. (1996). Suicide attempts among Inuit youth: A community survey of prevalence and risk factors. Acta Psychiatr. Scand..

[118] Pollock N.J., Mulay S., Valcour J., Jong M. (2016). Suicide Rates in Aboriginal Communities in Labrador, Canada. Am. J. Public Health.

[119] Tan J.C., Maranzan K.A., Boone M., Vander Velde J., Levy S. (2012). Caller characteristics, call contents, and types of assistance provided by caller sex and age group in a Canadian Inuit crisis line in Nunavut, 1991–2001. Suicide Life Threat. Behav..

[120] Logie C.H., Lys C.L., Sokolovic N., Mackay K.I., Donkers H., Kanbari A., Pooyak S., Loppie C. (2021). Contextual factors associated with depression among Northern and Indigenous adolescents in the Northwest Territories, Canada. Glob. Ment. Health (Camb.).

[121] Affleck W., Oliffe J.L., Inukpuk M.M., Tempier R., Darroch F., Crawford A., Séguin M.S. (2022). Suicide amongst young Inuit males: The perspectives of Inuit health and wellness workers in Nunavik. SSM-Qual. Res. Health.

[122] Haggarty J.M., Cernovsky Z., Husni M., Minor K., Kermeen P., Merskey H. (2002). Seasonal affective disorder in an Arctic community. Acta Psychiatr. Scand..

[123] Middleton J., Cunsolo A., Pollock N., Jones-Bitton A., Wood M., Shiwak I., Flowers C., Harper S.L. (2021). Temperature and place associations with Inuit mental health in the context of climate change. Environ. Res..

[124] Willox A.C., Harper S.L., Edge V.L., Landman K., Houle K., Ford J.D., Govt R.I.C. (2013). The land enriches the soul: On climatic and environmental change, affect, and emotional health and well-being in Rigolet, Nunatsiavut, Canada. Emot. Space Soc..

[125] Galloway T., Johnson-Down L., Egeland G.M. (2015). Socioeconomic and Cultural Correlates of Diet Quality in the Canadian Arctic: Results from the 2007–2008 Inuit Health Survey. Can. J. Diet. Pract. Res..

[126] Zienczuk N., Egeland G.M. (2012). Association between socioeconomic status and overweight and obesity among Inuit adults: International Polar Year Inuit Health Survey, 2007–2008. Int. J. Circumpolar Health.

[127] Basham C.A., Karim M.E. (2019). Multimorbidity prevalence in Canada: A comparison of Northern Territories with Provinces, 2013/14. Int. J. Circumpolar Health.

[128] Hopping B.N., Erber E., Mead E., Sheehy T., Roache C., Sharma S. (2010). Socioeconomic indicators and frequency of traditional food, junk food, and fruit and vegetable consumption amongst Inuit adults in the Canadian Arctic. J. Hum. Nutr. Diet..

[129] Schmidt R., Hrenchuk C., Bopp J., Poole N. (2015). Trajectories of women’s homelessness in Canada’s 3 northern territories. Int. J. Circumpolar Health.

[130] Baron M., Riva M., Fletcher C. (2019). The social determinants of healthy ageing in the Canadian Arctic. Int. J. Circumpolar Health.

[131] Logie C.H., Lys C.L., Mackay K., MacNeill N., Pauchulo A., Yasseen A.S. (2019). Syndemic Factors Associated with Safer Sex Efficacy Among Northern and Indigenous Adolescents in Arctic Canada. Int. J. Behav. Med..

[132] Young T.K. (1996). Sociocultural and behavioural determinants of obesity among Inuit in the central Canadian Arctic. Soc. Sci. Med..

[133] Young M.G., Manion K. (2017). Harm reduction through housing first: An assessment of the Emergency Warming Centre in Inuvik, Canada. Harm Reduct. J..

[134] Kovesi T., Gilbert N.L., Stocco C., Fugler D., Dales R.E., Guay M., Miller J.D. (2007). Indoor air quality and the risk of lower respiratory tract infections in young Canadian Inuit children. CMAJ.

[135] Hansen V., Sabo A., Korn J., MacLean D., Riget F.F., Clausen D.S., Cubley J. (2023). Indoor radon survey in Whitehorse, Canada, and dose assessment. J. Radiol. Prot..

[136] Pepin C., Muckle G., Moisan C., Forget-Dubois N., Riva M. (2018). Household overcrowding and psychological distress among Nunavik Inuit adolescents: A longitudinal study. Int. J. Circumpolar Health.

[137] Young T.K., Mollins C.J. (1996). The impact of housing on health: An ecologic study from the Canadian Arctic. Arctic Med. Res..

[138] Ruiz-Castell M., Muckle G., Dewailly E., Jacobson J.L., Jacobson S.W., Ayotte P., Riva M. (2015). Household crowding and food insecurity among Inuit families with school-aged children in the Canadian Arctic. Am. J. Public Health.

[139] Daley K., Castleden H., Jamieson R., Furgal C., Ell L. (2014). Municipal water quantities and health in Nunavut households: An exploratory case study in Coral Harbour, Nunavut, Canada. Int. J. Circumpolar Health.

[140] Daley K., Castleden H., Jamieson R., Furgal C., Ell L. (2015). Water systems, sanitation, and public health risks in remote communities: Inuit resident perspectives from the Canadian Arctic. Soc. Sci. Med..

[141] Cassivi A., Carabin A., Dorea C., Rodriguez M.J., Guilherme S. (2024). Domestic access to water in a decentralized truck-to-cistern system: A case study in the Northern Village of Kangiqsualujjuaq, Nunavik (Canada). J. Water Health.

[142] Chatwood S., Paulette F., Baker G.R., Eriksen A.M.A., Hansen K.L., Eriksen H., Hiratsuka V., Lavoie J., Lou W., Mauro I. (2017). Indigenous Values and Health Systems Stewardship in Circumpolar Countries. Int. J. Environ. Res. Public Health.

[143] Kral M.J., Idlout L., Minore J.B., Dyck R.J., Kirmayer L.J. (2011). Unikkaartuit: Meanings of well-being, unhappiness, health, and community change among Inuit in Nunavut, Canada. Am. J. Community Psychol..

[144] Newell S.L., Dion M.L., Doubleday N.C. (2020). Cultural continuity and Inuit health in Arctic Canada. J. Epidemiol. Community Health.

[145] Emanuelsen K., Pearce T., Oakes J., Harper S.L., Ford J.D. (2020). Sewing and Inuit women’s health in the Canadian Arctic. Soc. Sci. Med..

[146] Glass C.T.R., Giles A.R. (2020). Community-based risk messaging in Inuvik, Northwest Territories, Canada. Health Promot. Int..

[147] Moller H. (2013). “Double culturedness”: The “capital” of Inuit nurses. Int. J. Circumpolar Health.

[148] Hordyk S.R., Macdonald M.E., Brassard P. (2017). Inuit interpreters engaged in end-of-life care in Nunavik, Northern Quebec. Int. J. Circumpolar Health.

[149] Akande V.O., Ruiter R.A.C., Kremers S.P.J. (2019). Environmental and Motivational Determinants of Physical Activity among Canadian Inuit in the Arctic. Int. J. Environ. Res. Public Health.

[150] Baron M., Fletcher C., Riva M. (2020). Aging, Health and Place from the Perspective of Elders in an Inuit Community. J. Cross Cult. Gerontol..

[151] Akande V.O., Fawehinmi T.O., Ruiter R.A.C., Kremers S.P.J. (2021). Healthy Dietary Choices and Physical Activity Participation in the Canadian Arctic: Understanding Nunavut Inuit Perspectives on the Barriers and Enablers. Int. J. Environ. Res. Public Health.

[152] Cascini F., Hoxhaj I., Zaçe D., Ferranti M., Di Pietro M.L., Boccia S., Ricciardi W. (2020). How health systems approached respiratory viral pandemics over time: A systematic review. BMJ Glob. Health.

[153] Springer Y.P., Kammerer J.S., Silk B.J., Langer A.J. (2022). Tuberculosis in Indigenous Persons—United States, 2009–2019. J. Racial Ethn. Health Disparities.

[154] Hennessy T., Bruden D., Castrodale L., Komatsu K., Erhart L., Thompson D., Bradley K., O’LEARY D., McLaughlin J., Landen M. (2016). A case-control study of risk factors for death from 2009 pandemic influenza A (H1N1): Is American Indian racial status an independent risk factor?. Epidemiol. Infect..

[155] SteelFisher G.K., Blendon R.J., Kang M., Ward J.R., Kahn E.B., Maddox K.E., Lubell K.M., Tucker M., Ben-Porath E.N. (2015). Adoption of preventive behaviors in response to the 2009 H1N1 influenza pandemic: A multiethnic perspective. Influenza Other Respir. Viruses.

[156] Duarte R., Aguiar A., Pinto M., Furtado I., Tiberi S., Lönnroth K., Migliori G.B. (2021). Different disease, same challenges: Social determinants of tuberculosis and COVID-19. Pulmonology.

[157] United Nations, Department of Economic and Social Affairs Indigenous Peoples COVID-19 and Indigenous Peoples. https://www.un.org/development/desa/indigenouspeoples/covid-19.html.

[158] Petrov A.N., Dorough D.S., Tiwari S., Welford M., Golosov N., Devlin M., Degai T., Ksenofontov S., DeGroote J. (2023). Indigenous health-care sovereignty defines resilience to the COVID-19 pandemic. Lancet.

[159] Zavaleta-Cortijo C., Ford J.D., Galappaththi E.K., Namanya D.B., Nkwinti N., George B., Togarepi C., Akugre F.A., Arotoma-Rojas I., Pickering K. (2023). Indigenous knowledge, community resilience, and health emergency preparedness. Lancet Planet. Health.

[160] Northwest Territories Health and Social Services Authority PUBLIC NOTICE: Service Update – Stanton Territorial Hospital and Medical Travel. https://www.nthssa.ca/en/newsroom/public-notice-service-update-stanton-territorial-hospital-and-medical-travel.

[161] Government of Nunavut COVID-19 GN Update—19 March 2020. https://gov.nu.ca/executive-and-intergovernmental-affairs/news/covid-19-gn-update-march-19-2020.

[162] Vose J.M. (2020). Delay in Cancer Screening and Diagnosis During the COVID-19 Pandemic: What Is the Cost?. Oncology (Williston Park).

[163] Toro M.D., Brezin A.P., Burdon M., Cummings A.B., Evren Kemer O., Malyugin B.E., Prieto I., Teus M.A., Tognetto D., Tornblom R. (2021). Early impact of COVID-19 outbreak on eye care: Insights from EUROCOVCAT group. Eur. J. Ophthalmol..

[164] Lapum J., Nguyen M., Fredericks S., Lai S., McShane J. (2021). “Goodbye … Through a Glass Door”: Emotional Experiences of Working in COVID-19 Acute Care Hospital Environments. Can. J. Nurs. Res..

[165] Menzies P. (2010). Intergenerational Trauma from a Mental Health Perspective. Nativ. Soc. Work. J..

[166] Bombay A., Matheson K., Anisman H. (2014). The intergenerational effects of Indian Residential Schools: Implications for the concept of historical trauma. Transcult. Psychiatry.

[167] Talevi D., Socci V., Carai M., Carnaghi G., Faleri S., Trebbi E., di Bernardo A., Capelli F., Pacitti F. (2020). Mental health outcomes of the COVID-19 pandemic. Riv. Psichiatr..

[168] Lagacé-Wiens P., Bullard J., Cole R., Van Caeseele P. (2021). Seasonality of coronaviruses and other respiratory viruses in Canada: Implications for COVID-19. Can. Commun. Dis. Rep..

[169] Banning J. (2020). How Indigenous people are coping with COVID-19. Cmaj.

[170] Hotıì ts’eeda Northwest Territories SPOR Support Unit COVID-19 Resources for the NWT. https://nwtspor.ca/supported-projects/covid-19-resources-nwt.

[171] Hayward A., Cidro J., Dutton R., Passey K. (2020). A review of health and wellness studies involving Inuit of Manitoba and Nunavut. Int. J. Circumpolar Health.

[172] Peiris D., Brown A., Cass A. (2008). Addressing inequities in access to quality health care for indigenous people. CMAJ.

[173] Marrone S. (2007). Understanding barriers to health care: A review of disparities in health care services among indigenous populations. Int. J. Circumpolar Health.

[174] Shahid S., Thompson S.C. (2009). An overview of cancer and beliefs about the disease in Indigenous people of Australia, Canada, New Zealand and the US. Aust. N. Z. J. Public Health.

[175] Blanchet R., Batal M., Johnson-Down L., Johnson S., Louie C., Terbasket E., Terbasket P., Wright H., Willows N., Okanagan Nation Salmon Reintroduction I. (2021). An Indigenous food sovereignty initiative is positively associated with well-being and cultural connectedness in a survey of Syilx Okanagan adults in British Columbia, Canada. BMC Public Health.

[176] Kolahdooz F., Jang S.L., Corriveau A., Gotay C., Johnston N., Sharma S. (2014). Knowledge, attitudes, and behaviours towards cancer screening in indigenous populations: A systematic review. Lancet. Oncol..

[177] Yi K.J., Landais E., Kolahdooz F., Sharma S. (2015). Factors influencing the health and wellness of urban aboriginal youths in Canada: Insights of in-service professionals, care providers, and stakeholders. Am. J. Public Health.

[178] Nader F., Kolahdooz F., Sharma S. (2017). Assessing Health Care Access and Use among Indigenous Peoples in Alberta: A Systematic Review. J. Health Care Poor Underserved.

[179] Kolahdooz F., Launier K., Nader F., Yi K.J., Baker P., McHugh T., Vallianatos H., Sharma S. (2016). Canadian Indigenous Women’s Perspectives of Maternal Health and Health Care Services: A Systematic Review. Divers. Equal. Health Care.

[180] Statistics Canada Household Food Insecurity, 2017/2018. https://www150.statcan.gc.ca/n1/pub/82-625-x/2020001/article/00001-eng.htm.

[181] Polsky J.Y., Gilmour H. (2020). Food insecurity and mental health during the COVID-19 pandemic. Health Rep..

[182] Rahman S., Hossain I., Mullick A.R., Khan M.H. (2020). Food Security and the Coronavirus Disease 2019 (COVID-19): A Systematic Review. J. Med. Sci. Clin. Res..

[183] Elsahoryi N., Al-Sayyed H., Odeh M., McGrattan A., Hammad F. (2020). Effect of COVID-19 on food security: A cross-sectional survey. Clin. Nutr. ESPEN.

[184] Kent K., Murray S., Penrose B., Auckland S., Visentin D., Godrich S., Lester E. (2020). Prevalence and Socio-Demographic Predictors of Food Insecurity in Australia during the COVID-19 Pandemic. Nutrients.

[185] Mayasari N.R., Ho D.K.N., Lundy D.J., Skalny A.V., Tinkov A.A., Teng I.C., Wu M.C., Faradina A., Mohammed A.Z.M., Park J.M. (2020). Impacts of the COVID-19 Pandemic on Food Security and Diet-Related Lifestyle Behaviors: An Analytical Study of Google Trends-Based Query Volumes. Nutrients.

[186] Sharma S., Hopping B.N., Roache C., Sheehy T. (2013). Nutrient intakes, major food sources and dietary inadequacies of Inuit adults living in three remote communities in Nunavut, Canada. J. Hum. Nutr. Diet..

[187] Sheehy T., Kolahdooz F., Schaefer S.E., Douglas D.N., Corriveau A., Sharma S. (2015). Traditional food patterns are associated with better diet quality and improved dietary adequacy in Aboriginal peoples in the Northwest Territories, Canada. J. Hum. Nutr. Diet..

[188] Rosol R., Powell-Hellyer S., Chan H.M. (2016). Impacts of decline harvest of country food on nutrient intake among Inuit in Arctic Canada: Impact of climate change and possible adaptation plan. Int. J. Circumpolar Health.

[189] Pakseresht M., Lang R., Rittmueller S., Roache C., Sheehy T., Batal M., Corriveau A., Sharma S. (2014). Food expenditure patterns in the Canadian Arctic show cause for concern for obesity and chronic disease. Int. J. Behav. Nutr. Phys. Act..

[190] Erber E., Beck L., Hopping B.N., Sheehy T., De Roose E., Sharma S. (2010). Food patterns and socioeconomic indicators of food consumption amongst Inuvialuit in the Canadian Arctic. J. Hum. Nutr. Diet..

[191] Pereira M., Oliveira A.M. (2020). Poverty and food insecurity may increase as the threat of COVID-19 spreads. Public Health Nutr..

[192] McLeod M., Gurney J., Harris R., Cormack D., King P. (2023). COVID-19: We must not forget about Indigenous health and equity. Aust. N. Z. J. Public Health.

[193] Peterson M., Akearok G.H., Cueva K., Lavoie J.G., Larsen C.V., Johannsdottir L., Cook D., Nilsson L.M., Rautio A., Timlin U. (2023). Public health restrictions, directives, and measures in Arctic countries in the first year of the COVID-19 pandemic. Int. J. Circumpolar Health.

[194] National Advisory Committee on Immunization Archived: Guidance on the Prioritization of Initial Doses of COVID-19 Vaccine(s). https://www.canada.ca/en/public-health/services/immunization/national-advisory-committee-on-immunization-naci/guidance-prioritization-initial-doses-covid-19-vaccines.html.

[195] Young T.K., Broderstad A.R., Sumarokov Y.A., Bjerregaard P. (2020). Disparities amidst plenty: A health portrait of Indigenous peoples in circumpolar regions. Int. J. Circumpolar Health.

