# Supplementary material for: A Scoping Review of the Current Knowledge of the Social Determinants of Health and Infectious Diseases (Specifically COVID-19, Tuberculosis, and H1N1 Influenza) in Canadian Arctic Indigenous Communities
Source: Int J Environ Res Public Health. 2024 Dec 24;22(1):1. doi: 10.3390/ijerph22010001 (PMC11765080; doi:10.3390/ijerph22010001)
Supplement: Supplementary file 1 [file ijerph-22-00001-s001.zip › Table S2. Search strategy.pdf]

Table S2. Search strategy

	Filter: English or French		
	Pubmed and CINAHL		Medline
1	Arctic Canada	COVID-19	1. exp Arctic Regions/ or arctic.mp.<br>2. canada.mp. or exp Canada/<br>3. 1 and 2<br>4. COVID-19.mp. or exp COVID-19/<br>5. 3 and 4<br>6. limit 5 to English language<br>7. limit 5 to French language
2	Arctic Canada	TB OR tuberculosis	1. exp Arctic Regions/ or arctic.mp.<br>2. canada.mp. or exp Canada/<br>3. 1 and 2<br>4. Tuberculosis. mp. Or exp Tuberculosis/<br>5. 3 and 4<br>6 limit 5 to English language<br>7. limit 5 to French language
3	Arctic Canada	influenza OR H1N1	1. exp Arctic Regions/ or arctic.mp.<br>2. canada.mp. or exp Canada/<br>3. 1 and 2<br>4. influenza. mp. Or exp Influenza, Human/<br>5. exp Influenza A Virus, H1N1 Subtype/or H1N1. mp<br>6. 4 and 5<br>7. limit 6 to English language<br>8. limit 6 to French language
4	Arctic Canada	cultur*<br>language*	1. exp Arctic Regions/ or arctic.mp.<br>2. canada.mp. or exp Canada/<br>3. 1 and 2<br>4. culture.mp. or exp Culture/<br>5. exp Language/ or language.mp.<br>6. 4 or 5<br>7. 3 and 6<br>8. limit 7 to English language

			9. limit 7 to French language
5	Arctic Canada	livelihood*<br>employment<br>income	1. exp Arctic Regions/ or arctic.mp.<br>2. canada.mp. or exp Canada/<br>3. 1 and 2<br>4. livelihood.mp.<br>5. employment.mp. or exp Employment/<br>6. income.mp. or exp Income/<br>7. 4 or 5 or 6<br>8. 3 and 7<br>9. limit 8 to English language<br>10. limit 8 to French language
6	Arctic Canada	hous*<br>famil*<br>habitat*	1. exp Arctic Regions/ or arctic.mp.<br>2. canada.mp. or exp Canada/<br>3. 1 and 2<br>4. housing.mp. or exp Housing/<br>5. exp Family Characteristics/ or exp Family/ or exp Family Relations/ or family.mp.<br>6. habitat.mp.<br>7. 4 or 5 or 6<br>8. 3 and 7<br>9. limit 8 to English language<br>10. limit 8 to French language
7	Arctic Canada	education	1. exp Arctic Regions/ or arctic.mp.<br>2. canada.mp. or exp Canada/<br>3. 1 and 2<br>4. education.mp. or exp Education/<br>5. 3 and 4<br>6. limit 5 to English language<br>7. limit 5 to French language
8	Arctic Canada	food AND (security OR insecurity)<br>cost of living<br>country OR traditional AND food<br>subsistence	1. exp Arctic Regions/ or arctic.mp.<br>2. canada.mp. or exp Canada/<br>3. 1 and 2<br>4. food security.mp. or exp Food Supply/

			5. cost of living.mp. or exp Economics/<br>6. traditional food.mp.<br>7. country food.mp.<br>8. subsistence.mp.<br>9. 4 or 5 or 6 or 7 or 8<br>10. 3 and 9<br>11. limit 10 to English language<br>12. limit 10 to French language
9	Arctic Canada	healthcare OR "health care" AND (utilization OR access OR availability OR quality OR service OR system* OR person* OR provider OR staff)<br>"medical travel"	1. exp Arctic Regions/ or arctic.mp.<br>2. canada.mp. or exp Canada/<br>3. 1 and 2<br>4. healthcare.mp.<br>5. utilization.mp.<br>6. access.mp.<br>7. availability.mp. or exp Health Services Accessibility/<br>8. exp "Quality of Health Care"/ or quality.mp.<br>9. service.mp.<br>10. system.mp.<br>11. healthcare provider.mp. or exp Health Personnel/<br>12. medical travel.mp.<br>13. 5 or 6 or 7 or 8 or 9 or 10<br>14. 4 and 13<br>15. 11 or 12 or 14<br>16. 3 and 15<br>17. limit 16 to English language<br>18. limit 16 to French language
10	Arctic Canada	mental AND health OR wellness<br>depression<br>suicide<br>substance OR alcohol AND use OR misuse OR abuse OR dependen*	1. exp Arctic Regions/ or arctic.mp.<br>2. canada.mp. or exp Canada/<br>3. 1 and 2<br>4. mental health.mp. or exp Mental Health/<br>5. depression.mp. or exp Depression/<br>6. exp Suicide/ or suicide.mp.<br>7. substance.mp. or exp Substance-Related Disorders/<br>8. exp Alcohol Drinking/ or alcoholism.mp. or exp Alcoholism/

			9. 4 or 5 or 6 or 7 or 8<br>10. 3 and 9<br>11. limit 10 to English language<br>12. limit 10 to French language
11	Arctic Canada	community AND infrastructure OR resource* OR capacit*	1. exp Arctic Regions/ or arctic.mp.<br>2. canada.mp. or exp Canada/<br>3. 1 and 2<br>4. community.mp. or exp Residence Characteristics/<br>5. infrastructure.mp.<br>6. resource.mp.<br>7. capacity.mp.<br>8. 5 or 6 or 7<br>9. 4 and 8<br>10. 3 and 9<br>11. limit 10 to English language<br>12. limit 10 to French language
