# Supplementary material for: A Scoping Review of the Current Knowledge of the Social Determinants of Health and Infectious Diseases (Specifically COVID-19, Tuberculosis, and H1N1 Influenza) in Canadian Arctic Indigenous Communities
Source: Int J Environ Res Public Health. 2024 Dec 24;22(1):1. doi: 10.3390/ijerph22010001 (PMC11765080; doi:10.3390/ijerph22010001)
Supplement: Supplementary file 1 [file ijerph-22-00001-s001.zip › Table S3. Characteristics and major findings of selected articles.pdf]

Table S3. Characteristics and major findings of selected articles

First Author, Reference, Publication Year	Study Design			Findings
	Location	Population	Methods	
**Infectious Disease 1: COVID-19**				
Akearok GKH, et al.,[30] 2024	Iqaluit, Nunavut	22 community members, 17 frontline workers, and 5 decision makers	Interviews (Qualitative)	- Challenges to addressing the COVID-19 outbreak in Iqaluit included overcrowded housing, social disconnection from family, and mental health issues including alcohol and cannabis misuse<br>- Strengths highlighted during the outbreak were partnership among the government and community organizations, dedicated frontline workers, community supports, and travel restrictions.
Lavoie JG, et al.,[31] 2023	Manitoba	14 healthcare service providers	Interviews (Qualitative)	- The cross-jurisdictional healthcare systems between Manitoba and Nunavut incorporated strategies to mitigate the impact of COVID-19 on challenges to providing healthcare services to Nunavut residents accessing services in Manitoba: expanding virtual care and cross-jurisdictional healthcare coordination.<br>- Intrastructure limitations existed to expand virtual care and access electronic medical records.
Vilches TN, et al.,[40] 2022	Nunavut	N/A	Theoretical model (Quantitative)	- Without non-pharmaceutical interventions (i.e. strict lock down measures), the peak incidence would have been 4.7 times higher, even with accelerated vaccination.
Tiwari S, et al.,[39] 2022	Canada, Denmark, Finland, Iceland, Norway, Sweden, Russia, and the USA	N/A	Analyses of regional data (Fall 2021-January 2022) (Quantitative)	- Many Arctic regions, including Northern Canada, showed a tsunami-like COVID-19 pattern and had abrupt spikes in daily cases and corresponding increase in fatalities starting fall 2021.<br>- Some Arctic regions, including Northern Canada, had lower cumulative cases compared to the respective nation.
Petrov AN, et al.,[38] 2021	Canada, Denmark, Finland, Iceland, Norway, Sweden, Russia, and the USA	N/A	Analyses of regional data (February 2020-	- Northern Canada had the spread under control, with very few deaths.

			January 2021) (Quantitative)	- Northern Canada was at a "pre-pandemic" state until later in 2020 and had had a smaller second wave in November and December 2020.
Petrov AN, et al.,[4] 2020	Canada, Denmark, Finland, Iceland, Norway, Sweden, Russia and the USA	N/A	Analyses of regional data (February-July 2020) (Quantitative)	- Northern Canada and Greenland did not have significant COVID-19 outbreaks between February and July 2020.
**Infectious Disease 2: H1N1 Influenza**				
Banerji A, et al.,[41] 2016	- NWT, Nunavut, and Northern Quebec	- 348 admissions of infants (<1 year) with lower respiratory tract infection	- Prospective multicentre surveillance study (Quantitative)	- Influenza A (H1N1) was found in 12% of the cases.
Charania NA, et al.[43] 2013	- Northern Ontario	- 9 adult key informants	- Interviews (Qualitative)	- Lack of resources (supplies, monies, trained personnel) and poor community awareness were barriers to implementing measures.
Charania NA, et al.,[44] 2012	- Northern Ontario	- Community stakeholders involved in the community's pandemic response	- Interviews and meetings (Qualitative)	- Participants suggested adding the following in the pandemic response: community-specific detail about supplies and resources needed due to the geographical remoteness of study communities, detail on how, when, where, and who was responsible for implementing the pandemic plans.
Charania NA, et al.,[42] 2011	- Northern Ontario	- 13 adult key informants in health care services	- Interviews (Qualitative)	- Barriers included receiving contradicting governmental guidelines and direction from many sources, and a lack of human resources, information sharing, and specific details in community-level pandemic plans.<br>- Developing a communication plan, increasing human resources, and updating community-level pandemic plans were suggested.
Charania NA et al.,[17] 2011	- Northern Ontario	- 13 adult key informants in health care services	- Interviews (Qualitative)	- Participants experienced challenges related to overcrowded housing, limited human resources, and limited community awareness when delivering healthcare services during the pandemic.
**Infectious Disease 3: Tuberculosis**				

Alvarez GG, et al.,[49] 2021	- Nunavut	- 185 TB cases among 178 patients	- Social network analysis and genome sequencing (Quantitative)	- The largest super-spreading events were associated with a homeless shelter and a gambling house, suggesting a socioeconomic nature of TB spread.
Kilabuk E, et al.,[45] 2019	- Nunavut	- 261 participants, mostly Inuit	- In-person surveys (Quantitative)	- Unadjusted risk ratios for latent TB infection were associated with age, education, smoking tobacco, crowded housing, and Inuit ethnicity.<br>- After adjusting for other SDH, latent TB infection was associated with increasing age, crowded housing, and ethnicity.
Pease C, et al.,[46] 2019	- Nunavut	- 2,303 patients with a tuberculin skin test implanted	- Retrospective review of medical records (Quantitative)	- Undergoing a tuberculin skin test due to employment screening was associated with increased non-initiation of treatment.
Bourgeois AC, et al.,[47] 2018	- Canada, Finland, Greenland, Norway, Russia, Sweden, and the US	- 7,213 cases of tuberculosis reported in the International Circumpolar Surveillance Tuberculosis system.	- Descriptive analysis of all active TB cases (Quantitative)	- The annual TB incidence ranged from 4.3/100,000 (Northern Sweden) to 199.5/100,000 (Northern Quebec, Canada).<br>- Jurisdictions with the highest proportion of cases that were classified as cured or as having completed treatment were Yukon (96.6%), the NWT (85.7%), Northern Norway (84.6%), and Alaska (83.6%).<br>- The highest proportions of death among TB cases were observed in Arkhangelsk (13.6%) and the NWT (13.0%).
Alvarez GG, et al.,[48] 2016	- Nunavut	- 41 Inuit youth (12-20 years)	- Field test case study using a knowledge-to-action process (Quantitative)	- The youth-led interventions were acceptable to the Inuit communities and resulted in uptake of TB knowledge among community participants.
**SDH 1: Healthcare Access**				
Falk R, et al.,[89] 2023	- NWT	- N/A	- Discharge Abstract Database and Natioinal Ambulatory Care Reporting	- Family physicians with enhanced surgical skills performed 47.7% of all surgical and endoscopic procedures (21.9% of surgeries, and 78.7% of endoscopic procedures, and surgical specialists, either itinerant or out-of-territory (medical travel) performed 52.3%.

			Service data (Quantitative)	
Falk R, et al.,[90] 2022	- NWT	- 22 stakeholders in the surgical system	- Semi-structured interviews (Qualitative)	- Family physicians with enhanced surgical skills can be primary surgical providers in remote regions.<br>- Referrals for a surgery outside of the scope of family physicians with enhanced surgical skills can be supported by specilist surgeons.
Clark W, et al.,[102] 2022	- Manitoba	- N/A	- Administrative data (Quantitative)	- The use of out-patient services in Manitoba has declined among Inuit from Nunavut.<br>- Main reasons for hospitalization were pregnancy and birth, and respiratory diseases for Inuit from Nunavut, and mental health for Inuit living in Manitoba.
Jull J, et al.[100], 2021	- Nunavut and Ontario	- 8 Inuit cancer patients and 6 medical escorts of Inuit cancer patients on medical travel in Ontario	- Semi-structured interviews (Qualitative)	- Participants described the experience in relation to 4 main events through the cancer care journey: initiating cancer care, getting cancer care, getting supports from family and community, and engaging in decision making.<br>- Most participants were away from home for over one month.<br>- Participants experienced challenges such as delay in the initial diagnosis, overwhelming travel logistics that often involved more than one flight, limited information and support, geography, weather, and commitments to job and family care.
Hansen N, et al.,[86] 2021	- Yukon, NWT, and Nunavut	- 57 physicians	- Survey and semi-structured interview (Mixed-method)	- Positive experiences among the participants were directly associated with love of work and coworkers' support, and inversely associated with using electronic medical record, insufficient remuneration, and cultural issues.<br>- Factors contributing to physician burnout included "lack of influence on policy and administration", "systemic failures in cultural safety", "discontinuity of care", "upshifting of tasks", "physician turnover", and "lack of systemic supports".

				- Factors mitigating physician burnout included "relationships" with colleagues and communities and "time on the land and outdoor activities"<br>- Factors that could either contribute to or mitigate physician burnout included "scope of practice", "blurring boundaries", and "time away".
Cooper R, et al.,[88] 2021	- NWT	- N/A	- Review of 128 eligible news media articles covering 71 patient experience cases (Mixed-method)	- Most articles reported negative patient experiences related to "structural barriers to care", "perpetuation of inequalities and colonial legacy", and challenges with "health system communication".<br>- Positive patient experiences related to quick follow-ups and referrals, improved access to treatments, and telehealth.
Galloway T, et al.,[83] 2020	- Nunavut	- 10 patients and family members who experienced cancer or end of life care	- Interviews (Qualitative)	- The following themes were identified: difficulties associated with extensive medical travel, preference for care within the community and for family involvement in care, challenges with communication, challenges with culturally appropriate care, and the value of service providers with strong ties to the community.
Logie CH, et al.,[85] 2019	- NWT	- 16 LGBTQ2S+ youth, 21 LGBTQ2S+ adults, and 14 key informants	- Interviews (Qualitative)	- Lack of healthcare facilities in small communities resulted in LGBTQ2S+ persons having concerns about confidentiality, anonymity, and privacy.<br>- Participants also had difficulties with access to seeing healthcare professionals due to long wait times.
Sheffield HA, et al.,[99] 2019	- Nunavut	- 10 paediatric or neonatal patients with respiratory distress or infections	- Case studies (Quantitative)	- The cases showed effective and safe use of non-invasive ventilation in air transport of patients in respiratory distress improved patient transport in rural and remote regions.
McDonnell L, et al.,[77] 2019	- Nunavut and Manitoba	- 90 frontline care providers and decision makers	- Interviews (Qualitative)	- Medevacs can be the result of several factors: the referring and receiving provider's experience, insufficient staffing in health centres, lack of access to diagnostic or treatment-related, and challenges related to recruitment and retention.

Kerber K, et al.,[96] 2019	- NWT	- 52 patients and patient escorts, 14 key informants	- One-on-one interviews, focus group discussions and key informant interviews (Qualitative)	- Respondents were satisfied with the care received overall with some unnecessary burdens and bureaucratic challenges throughout the travel process.<br>- To improve access to healthcare: improve information and logistic plans prior to travel, increase effective communication between services, reduce jurisdiction and bureaucratic barriers to care, reduce indirect costs of travel and direct costs of uninsured services, and have a patient escort or advocate available to assist with appointments and navigation of the system.
Young TK, et al.,[97] 2019	- Nunavut, NWT, Yukon	- 287 nurses and 61 physicians	- Analysis of medical travel data from the health departments, online surveys (Quantiative)	- The proportion of the population living within 100 km of a hospital was 83% in Yukon, 63% in NWT, and 21% in Nunavut.<br>- The relative absence of roads is a major reason why the patient transportation costs are high in Nunavut and NWT.<br>- Medical travel accounted for 5% of the health expenditure in NWT and 20% in Nunavut.<br>- A medevac on average costs $218 per person per year in NWT and $700 in Nunavut.
Young TK, et al.,[82] 2018	- 8 Arctic countries	- N/A	- Review and comparison of publicly available databases (Quantitative)	- Nordic countries exceeded North America in the density of all three categories of health professionals.<br>- The largest disparities between "north" and "south" are observed in the NWT and Nunavut, Canada..
Logie CH, et al.,[94] 2018	- NWT	- 16 LGBTQ2S+ youth (15-24 years), 21 LGBTQ2S+ adults (≥25 years), and 14 key informants	- Interviews (Qualitative)	- Participants discussed how structural contexts such as heteronormativity in sexual health education and a lack of access to safer sex tools constrained one’s ability to practice safer sex.<br>- Social contexts of intersectional stigma resulted in shame, concealing identities, and fear of accessing safer sex materials.
Liddy C, et al.,[95] 2017	- Nunavut	- 165 eConsult cases	- Cost analysis of all eConsult cases submitted	- Of the submitted 165 eConsult cases, the most popular specialties were dermatology (16%), cardiology (8%),

			between August 2014 and April 2016 (Quantitative)	endocrinology (7%), otolaryngology (7%), and obstetrics/gynaecology (7%).<br>- Specialists provided a response in a median of 0.9 days. Total savings associated with eConsult in Nunavut are estimated at $180,552.73 or $1,100.93 per eConsult.
Romain SJ, et al.,[81] 2015	- Nunavut	- 35 key informants	- Interviews (Qualitative)	- Significant human resources were utilized in community health centres to distribute duties associated with retail pharmacy medications.<br>- Large quantities of unclaimed prescription medications suggested suboptimal patient care and low adherence rates<br>- The absence of a clear policy and oversight for some controlled substances, such as narcotics, may increase the risk for potential illegal procurement or abuse.
Oosterveer TM, et al.,[80] 2015	- NWT	- 14 primary healthcare service providers and service users	- Interviews (Qualitative)	- Both primary care service providers and service users understood the constraints in providing equitable access to primary health care services in remote communities.<br>- Emergency care was found to be particularly challenging, because of limited qualified staff in the community and the dependence on aeromedical evacuations.<br>- For non-emergency care, the need to travel outside the community was generally not favoured.<br>- All recognized the need for more preventive services which were often postponed or delayed because of the overwhelming demand for acute care.
Logie CH, et al.,[93] 2015	- NWT	- 12 LGBTQ2S+ persons and 15 stakeholders	- A meeting with youth, a meeting with stakeholders (Qualitative)	- LGBTQ2S+ youth identified community norms that devalued same sex identities and stigma surrounding LGBTQ2S+-specific services and agencies.<br>- Stigma was exacerbated for youth in secondary schools.
Fraser SL, et al.,[87] 2015	- Northern Québec	- 14 community members including 3 elders	- Individual interviews (Qualitative)	- Themes that are inter-related with healthcare and social service experiences were identified, including needs for services, access to services, and satisfaction with care

Mendez I, et al.,[101] 2013	- Northern Newfoundland and Labrador	- Physicians, nurses and patients who utilized a total of 252 activation of remote presence	- Surveys (Quantitative)	- The use of the RP-7 robot that provided remote physician presence was estimated to have reduced air transport of patients by 60%.<br>- Physicians, nurses, and patients expressed a high degree of satisfaction, suggesting the use of robot technology for remote physician presence may be a feasible and cost-effective way to improve healthcare access in northern remote communities.
Bhattacharyya OK, et al.,[79] 2011	- Northwestern Ontario	- 24 healthcare providers	- Interviews and focus group (Qualitative)	- Clinic-related barriers such as short staffing, staff turnover and system fragmentation were discussed.
Bhattacharyya OK, et al.,[92] 2011	- Northwestern Ontario	- 24 healthcare providers	- Semi-structured interviews (Qualitative)	- Although healthcare providers ranked patient factors as having a large impact on care than CHRs, physicians were less likely to rank patient-provider communication as having a large impact.
McDonald TJ, et al.,[91] 2010	- Nunavut, NWT, Northern Labrador, Northern Quebec	- 14,282 Inuit, Métis, First Nation and non-Indigenous adults (21-65 years)	- Analysis of the Canadian Community Health Surveys and Aboriginal People's Survey by Statistics Canada (Quantitative)	- Lower utilization of cancer screening appeared to be due to unobserved factors specific to Inuit and the unique social-cultural context.
Bird SM, et al.,[84] 2008	- Nunavut	- 4 Inuit with type 2 diabetes mellitus for a minimum of 5 years and had been prescribed oral anti-hyperglycemic medication	- Interviews, field observations, and informal interviews (Qualitative)	- Accessibility was a concern with respect to foods, health knowledge, language interpretation, and health service.<br>- There was strong desire for diabetes education and support, coupled with skepticism towards outsiders.<br>- Talking, listening, and food sharing were described most often as examples of social support.
Tarlier DS, et al.,[78] 2007	- Northern regions of Western Canadian provinces	- 15 patients and health care providers	- Patient observations and interviews	- Remote geographical location, and nurses' level of preparedness to practise in the North formed patterns of social distancing in nurse-patient relationships and

			with care providers (Qualitative)	constrained nurses' ability to engage in practice that promotes continuity of care.
Chamberlain M, et al.,[98] 2000	- Central Canadian Arctic	- 23 postnatal women and partners and 5 community members.	- Interviews (Qualitative)	- Women faced many stressors as a result of being transferred from the community for the birth of the baby, including the absence of a partner and family support.<br>- Stressors were categorised as emotional, physical and economic.
**SDH 2: Food Insecurity**				
Logie CH, et al.,[60] 2024	- NWT	- 410 adolescents aged 13-18 years	- Surveys (Quantitative)	- 45% reported experiencing any food insecurity.
Blom CDB, et al.,[70] 2022	- Yukon	- 45 community members and 4 food system experts	- Online surveys with community members and semi-structured interviews with food system experts (Mixed-method)	- 45% of community members felt fresh, local food was expensive.<br>- 31% of community members faced financial barriers to purchase foods.<br>- 50% of food experts noticed a loss of knowledge about harvesting food from the land.
Gilbert SZ, et al.,[75] 2021	- Nunavut	- 30 Elders, hunters and trappers	- Semi-structured interviews (Qualitative)	- Participants noticed that environmenal impacts, reduced wildline populations, changing animal migration patterns, and financial limitations impacted the availability of country food.<br>- Community members coped through food sharing networks.
St-Germain AAF, et al.,[63] 2019	- Nunavut	- 3250 households	- Canadian Community Health survey data (Quantitative)	- In 2010, the year before Nutrition North Canada launched, 33.1% of households were food insecure.<br>- In 2011, the year of the launch, 39.4% of households were food insecure.<br>- In 2014, the year after the full implementation, 46.6% of households were food insecure.<br>- After the full implementation, the rate of food insecurity raised by 13.2%.

Ready E,[56] 2018	- Northern Quebec	- 109 households	- Household survey (Quantitative)	- Food sharing is more common in households with higher food availability.<br>- Poor, low-harvest households may be more vulnerable to disruptions in the availability of country food.
Huet C, et al.,[52] 2017	- Nunavut	- 431 households with children, 468 households without children	- In person interviews, telephone questionnaires (Quantitative)	- Food insecurity was significantly higher in households with children (32.9%) than in households without children (23.2%).<br>- Low formal education attainment of the person responsible for food preparation was associated with increased odds of food insecurity in households with children.
Teh L, et al.,[57] 2017	- Northern Quebec	- 130 pregnant women (18-48 years)	- Psychometric evaluation of a modified Household Food Insecurity Access Scale (Quantitative)	- Women were more likely to be food insecure at both ends of the sample's age distribution, particularly women with low formal educational attainment, who had three or more adults and/or children in the household, who were unemployed, and who had few household hunters/fishers.
Collings P, et al.,[69] 2016	- NWT	- 22 households	- A social network analysis of country food exchange (Quantitative)	- Households of single men and women experienced limited access to country food.
Beaumier MC, et al.,[76] 2015	- Nunavut	- 70 Inuit women, and 8 key informants	- Semi-structured interviews, focus groups, key informant interviews (Qualitative)	- Factors, such as high cost of living, limited income, unemployment, and substance use, were perceived to have a greater impact on food security than climate change.<br>- Full-time hunter in the family or extended family affected the availability of traditional food.
Guo Y, et al.,[51] 2015	- Nunavut	- 532 randomly selected households	- Modified United States Department of Agriculture Food Security	- 28.7% of surveyed households in Iqaluit were food insecure, a rate 3 times higher than the national average.<br>- Food insecurity was associated with poor quality housing and reliance on income support.

			Survey (Quantitative)	
Organ J, et al.,[71] 2014	- Northern Labrador	- 13 community freezer users, 2 volunteer managers, 2 elders, 9 active harvesters, and 6 freezer managers.	- Interview, focus groups, participant observation, and document analysis (Qualitative)	- The community freezer supported socio-cultural, economic, and local access to wild foods.<br>- There were issues associated with supply, dependency, social exclusion, and tension between feasibility and traditional values and practices.
Douglas V, et al.,[50] 2014	- Yukon	- 2 Elders, 2 youth, and 16+ key informants	- Interviews, focus groups, nutrient analysis of caribou muscles and organs. (Qualitative)	- The challenges to food security had 4 major themes: traditional harvest (cost and difficulty of harvesting caribou), market foods (poor nutritional quality, lack of nutrition education), decline in TF culture (younger generation losing connection to traditional culture), and adaptation strategies (improved food storage, gardening/food production and conservation through increased traditional education).
Skinner K, et al.,[54] 2014	- Northwestern Ontario	- One adult from 51 households	- Interviews (Qualitative)	- Food sharing, especially with family, was an important way to adapt to food shortages.<br>- Participants felt hunting, preserving and storing TF was important.
Skinner K, et al.,[55] 2013	- Northeastern Ontario	- 64 households	- Household Food Security Survey Module questionnaire (Quantitative)	- 70% of households were food insecure, 17% severely and 53% moderately.<br>- The prevalence of food insecurity in households with children was 76%.
Ford JD, et al.,[59] 2013	- NWT	- 61 CFP users.	- Photovoice workshops, a modified USDA food security survey, and open-ended interviews (Qualitative)	- CFP users were more likely to be housing insecure, female, middle aged (35-64), unemployed, Indigenous, and lack a high school education.

Spiegelaar NF, et al.,[72] 2013	- Northwestern Ontario	- 8 community members	- Interviews and analysis of soil samples	- Considering climate change, fruits and vegetables, historically stunted-in-growth or outside the distributional range of Subarctic Canada, could grow in the North.
Ford J, et al.,[58] 2012	- Nunavut	- 94 clients of CFPs	- Open ended interviews and a fixed-choice survey on a census (Qualitative)	- CFP users are more likely to be Inuit, be unemployed, have not completed high school, be more dependent on social assistance, have low household income, and be without hunters in the household compared to the general Iqaluit population.
Huet C, et al.,[53] 2012	- Nunavut	- 1901 Inuit households	- Survey (Quantitative)	- Food insecurity was associated with household crowding, income support, public housing, single adult households, having a home in need of major repairs, having an active hunter in the home.
Egeland GM, et al.,[62] 2011	- NWT, Nunavut, and Northern Quebec	- 1901 Inuit households accounting for 2595 adults.	- Survey (Quantitative)	- 62.6% of adults were food insecure.<br>- Food insecurity was associated with dietary inadequacies.
Nancarrow TL, et al.,[73] 2010	- Nunavut	- 17 adults	- Two-day bilingual focus groups (Qualitative)	- Three themes emerged from the observations: ice/snow/water, weather, and changes in species.<br>- Climate change can affect the accessibility and availability of the key species of country foods.<br>- Participants found that climate change was affecting the country food harvest in both positive and negative ways.
Wesche SD, et al.,[74] 2010	- NWT	- N/A	- Analysis or reports of climate observations or food use (Quantitative)	- Changes in access to, availability of, quality of, and ability to use TF resources has implications for quality of diet.
Egeland GM, et al.[61] 2010	- Nunavut	- 388 Inuit children aged 3-5 years, and the caregivers	- Survey (Quantitative)	- 69.6% of the households were food insecure (34.4% severely).<br>- 56.1% of children were food insecure (25.1% severely)<br>- Caregivers of severely food insecure children experienced the children going hungry (90.4%), skipping meals (75.8%), or not eating the whole day (60.1%).

				- 85.1% of caregivers of moderately food insecure children worried about food running out.
Ford JD, et al., [64] 2009	- Nunavut	- 50 Inuit community members	- Survey (Quantitative)	- 64% of the participants experienced food insecurity in the past year.<br>- Women and participants who consumed store-bought food were at an increased risk of food insecurity.<br>- Consuming traditional foods was associated with food security.
Lambden J, et al.,[66] 2007	- Arctic Canada	- Dene/Métis and Inuit women (≥20 years)	- Survey (Quantitative)	- 10%-38% of participants noticed recent changes in the quality and health of TF species.
Skinner K, et al.,[68] 2006	- Northwestern Ontario	- 22 adults and 60 youth in grades 6-8, 7 adult key informants	- Focus groups, one-on-one key informant interviews, and a community environment scan (Qualitative)	- Themes were empowerment, trust, resources, barriers and opportunities.<br>- Major sub-themes were food security, cost, accessibility/availability, capacity building, community support, programs/training and the school snack/breakfast program.
Chan HM, et al.,[65] 2006	- Nunavut	- 46 community members (17-60 years)	- Focus groups (Qualitative)	- Barriers to increased TF consumption included high costs of hunting and changes in lifestyle and cultural practices.
Lambden J, et al.,[67] 2006	- Arctic Canada	- 1771 Yukon First Nations, Dene/Métis and Inuit women	- Survey (Quantitative)	- A considerable regional variation in the ability to afford adequate food existed (40-70%) and it was reflected in the percentage of community members who could afford, or had access to, hunting or fishing equipment.<br>- Up to 50% of the responses indicated inadequate access to fishing and hunting equipment, and up to 46% of participants said not being able to afford to go hunting or fishing.
**SDH 3: Mental Health**				
Lavoie JG, et al.,[110] 2024	- Manitoba	- N/A	- Administrative data (Quantitative)	- Of all mental health consultations in Manitoba, Kivalliq Inuit accounted for 18-30%, and Inuit living in Manitoba accounted for 27-35%.<br>- Given >10 times the population of Kivalliq Inuit compared to Inuit living in Manitoba, there is

				underserved needs for mental health related consultations.
Poliakova N, et al.,[115] 2024	- Northern Quebec	- 1,326 Inuit	- 2017 Nunavik Inuit Health Survey (Quantitative)	- Lower depression scores were associated with higher family cohesion and participation in hunting/fishing activities weekly.<br>- Lower probability of suicide ideation was associated with higher community cohesion.
Affleck W, et al.,[121] 2022	- Northern Quebec	- 18 Inuit health workers	- Interviews (Qualitative)	- Factors impacting young Inuit men's suicidality includes: intergenerational trauma rooted from the Dog Slaughter (a historic event when dogs in Inuit communities were killed; transition from traditional life to modern life; and structural discrimination.<br>- There is a need to incorporate traditional masculine ideals in suicide prevention among young Inuit men.
Logie CH, et al.,[120] 2021	- NWT	- 339 adolescents (13-18 years)	- Questionnaire (Quantitative)	- Female cisgender, LGBQ2S+ identity, and food insecurity were independently and significantly associated with higher severity depression compared to the counterparts.
Middleton J, et al.,[123] 2021	- Northern Newfoundland and Labrador	- N/A	- Review of electronic patient records and temperature data from Environment and Climate Change Canada weather stations (Quantitativ)	- Warmer temperature was significantly associated with the incidence rate of daily community clinic visits related to mental health. Reduced land use and land-based resources due to warm temperature were suggested to affect mental health.
Decaluwe B, et al.,[105] 2019	- Nunavik, Quebec	- 174 adolescents	- Interview data from the Nunavik Child Development Study (Quantitative)	- A higher number of binge drinking episodes were reported among adolescents who frequently endorse enhancement motives, while social and coping motives have been exclusively related to binge drinking episodes among males.
Collins PY, et al.,[111] 2019	- Arctic countries (USA, Canada,	- 140 service providers,	- Delphi methodology	- The cross-national RISING SUN initiative prioritized outcomes for suicide prevention interventions.

	Scandinavian countries, Greenland, and Russia)	researchers, Indigenous community members, policy makers	and face-to-face discussions (Qualitative)	
Beaudoin V, et al.,[109] 2018	- Nunavut	- 30 suicide cases, 30 suicide attempt cases, 30 controls (never attempted suicide)	- Semi-structured interview (Qualitative)	- Environmental: control individuals had more stable family environment, financial security, and stable workplace than the other two groups.<br>- Use of service: People who attempted suicide had a higher use of mental health consultation, and people who dies of suicide were more likely to be hospitalized than the other two groups.<br>- Social: control individuals had a more stable intimate relationship, and pride in the parents than the other two groups.<br>- Individual: control individuals were more likely to get along with others, manage emotions, and have good school performance than the other two groups.<br>- Cultural: no significant differences between the three groups.
Fortin M, et al.,[104] 2016	- Arctic Quebec	- 248 Inuit women	- Interviews at mid-pregnancy, and at 1 and 11 months postpartum (Quantitative)	- The proportions of drinkers and bingers were 73% and 54% during the year prior to pregnancy and 62% and 33% after delivery.<br>- 60% of women continued to drink alcohol during pregnancy.
Pollock NJ, et al.,[118] 2016	- Newfoundland and Labrador	- 745 suicide cases	- Secondary analysis of Vital Statistics Death Database (Quantitative)	- Suicide rates were elevated among females in the Inuit communities.<br>- Suicide disproportionately affects Innu and Inuit communities in Labrador.
Petrasek MacDonald J, et al.,[112] 2015	- Northern Labrador	- 17 youth (15-25 years)	- In-depth conversational interviews (Qualitative)	- Five key protective factors for mental health were: being on the land, connecting to Inuit culture, strong communities, relationships with family and friends, and staying busy.

Fortin M, et al.,[116] 2015	- Northern Quebec	- Participants of two surveys combined	- Analysis of the Santé Québec Health Survey (1992) and the Nunavik Inuit Health Survey Qanuippitaa (2004) (Quantitative)	- Alcohol and drug use among Inuit increased significantly, particularly among young adults.<br>- 60% of drug users consumed alcohol on a regular basis.
Willox AC, et al.,[124] 2013	- Northern Newfoundland and Labrador	- 57 Inuit	- In-depth interviews (Qualitative)	- Many participants felt depressed or angry due to climate change, which had negative impact on the spiritual and cultural connection to the land.
O'Neill L, et al.,[114] 2013	- Yukon, NWT, and Northern British Columbia	- 20 Northern health practitioners	- Narrative interviews (Qualitative)	- Many practitioners coped with challenges of Northern practice, yet still experienced the limitations of small communities and lack of resources in small communities to adequately address mental health support.
Kral MJ,[113] 2013	- Nunavut	- 27 Inuit (17-61 years)	- Interviews (Qualitative)	- Relationships with girlfriends and parents were related to anger among Inuit male youth and embedded with suicides.
Tan JC, et al.,[119] 2012	- Nunavut, NWT, Labrador, and Northern Quebec	- 3974 received calls	- Quantitative and qualitative analysis of call records (Mixed-method)	- The majority of users were adult females who called to discuss problems primarily related to relationships and loneliness/boredom.
Wood DS,[107] 2011	- Nunavut	- 23 communities	- Analysis of community-level records of violent crimes known to the police (Quantitative)	- Wet communities had higher rates of violent crime compared to dry communities.
Haggarty JM, et al.,[106] 2008	- Nunavut	- 111 Inuit (≥14 years)	- Household survey (Quantitative)	- 43.6% of the participants had suicidal ideation within the past week and 30% attempted suicide within last 6 months.

				- Higher overall suicidality scores were associated with higher anxiety, and alcohol abuse, but not with depression or gender.
Law SF, et al.,[108] 2007	- Nunavut	- 110 clients	- Retrospective chart review (Quantitative)	- Interpersonal and socio-environmental stressors were precipitators of psychiatric crises such as suicide attempts.<br>- Psychiatric issues appeared deeply interwoven with interpersonal, socioeconomic, and societal changes.
Haggarty JM, et al.,[122] 2002	- An Inuit community above 70° N	- 88 residents	- Survey (Quantitative)	- 22.6% of the community sample was found to be depressed.<br>- 6.3% had seasonal affective disorder.
Kirmayer LJ, et al.,[103] 1998	- Nunavik, Quebec	- 203 Inuit (15-25 years)	- Secondary analysis of a community survey (Quantitative)	- A psychiatric problem, recent alcohol abuse, and cocaine or crack use were the strongest correlates of attempted suicide for females, while solvent use and number of recent life events were the strongest correlates for males.
Kirmayer LJ, et al.,[117] 1996	- Northern Quebec	- 14 interviews with Inuit (14-25 years)	- Interviews (Qualitative)	- Risk factors for suicide attempts included male gender, having a friend who had attempted or committed suicide, a history of being physically abused, a history of solvent abuse, and having a parent with an alcohol or drug problem.<br>- Protective factors included a family history of having received treatment for a psychiatric problem, more frequent church attendance, and a high level of academic achievement.
**SDH 4: Socioeconomic Status**				
Logie CH, et al.,[131] 2019	- NWT	- 610 adolescents (13-17 years)	- Cross-sectional survey (Quantitative)	- Addressing poverty and violence was suggested to advance adolescent sexual health in the NWT.
Baron M, et al.,[130] 2019	- NWT, Nunavut, Northern Quebec, Northern Labrador	- 850 adults (>50 years)	- Analysis of Statistics Canada's 2006 Aboriginal People Survey (Quantitative)	- In comparison to people reporting poor health, people reporting good health were more likely to have a higher individual income.

Basham CA, et al.,[127] 2019	- Canadian provinces and territories	- Canadians (2,849 in the Northern territories and 108,075) in the Southern provinces (>12 years)	- Analysis of the Canadian Community Health Survey data (Quantitative)	- Northern territories tended to have lower prevalence estimates than provinces for multimorbidity.<br>- Lower multimorbidity among households without a post-secondary graduate was found.
Young MG, et al.,[133] 2017	- NWT	- 9 respondents and 7 stakeholders	- Interviews (Qualitative)	- Several themes and subthemes related to participants' experiences at the emergency warming centre and success of the centre emerged including feeling safe.
Galloway T, et al.,[125] 2015	- NWT, Nunavut, Northern Quebec	- 2,595 Inuit adults (>18 years)<br>-	- Food frequency questionnaire and 24-h dietary recall (Quantitative)	- Associations between SES and diet quality differed considerably between men and women.
Schmidt R, et al.,[129] 2015	- Yukon, NWT, and Nunavut	- 61 homeless women (≥18 years)	- Interviews (Qualitative)	- Factors including poverty and unstable housing were interconnected and multifaceted challenges related to women's service engagement.<br>- Women called for integrated, trauma-informed, and women-centred services; and addressing of the social and economic factors that affect homelessness and mental health.
Zienczuk N, et al.,[126] 2012	- Nunavut, Northern Labrador, and NWT	- 2,592 Inuit adults (≥18 years)	- A cross-sectional health survey (Quantitative)	- Higher education, employment, personal income, and private housing were significantly positively correlated with an at-risk BMI.
Hopping BN, et al.,[128] 2010	- Nunavut	- 211 Inuit adults	- Food frequency questionnaires (Quantitative)	- Education was positively associated with fruit and vegetables consumption and negatively associated with TF consumption.<br>- Households on income support were more likely to consume TF and non-nutrient-dense foods.
Young TK, et al.,[132] 1996	- NWT	- 434 Inuit adults (>18 years)	- Community health survey (Quantitative)	- Obesity indices were associated with higher income, fluency in the Inuit language, and less time spent on the land.<br>- Obesity was associated with higher SES among Inuit men, and with lower SES among Inuit women.
**SDH 5: Cultural Continuity**				

Newell SL, et al.,[144] 2020	- NWT, Nunavut, Northern Quebec, Northern Labrador	- A total of 14,955 participants	- Analysis of the Arctic Supplements of the Aboriginal Peoples Surveys (Quantitative)	- Cultural continuity has a significantly positive association with health.<br>- Cultural continuity at a community level can potentially serve as a protective factor when faced with negative influences on health.
Glass CTR, et al.,[146] 2020	- NWT	- Participants of Inuvialuit or Gwich'in descent boating experience (18–85 years)	- Interviews and focus group discussions (Qualitative)	- There was a need for collaboration on boating safety resources that reflect Northerners' experiences and cultures, while promoting healthy behaviours.
Emanuelsen K, et al.[145] 2020	- NWT	- 30 Inuit women	- Semi-structured interviews (Qualitative)	- Sewing was found to contribute to Inuit women's personal health and the community's collective health by promoting "pride and sense of accomplishment", "cultural identity", "relaxation, decompression, and socialization", and "spirituality and healing".
Chatwood S, et al.,[142] 2017	- Arctic countries: United States, Canada, Norway and Finland	- 10 experts including healthcare professionals, informal caregivers, Indigenous leaders, and Elders	- Consensus-building (Qualitative)	- Nine Indigenous values in the healthcare setting were: humanity, cultural responsiveness, teaching, nourishment, community voice, kinship, respect, holism, and empowerment.
Hordyk SR, et al.,[148] 2017	- Northern Quebec	- 24 current and former interpreters from local health centres and tertiary care contexts	- Informal and formal interviews (Qualitative)	- Many interpreters have no formal training and have few resources (e.g., visual aids, dictionaries) to draw upon during medical consultations.<br>- Many interpreters personally knew the clients and often felt overwhelmed by moral dilemmas when translating end of life information for patients and families.
Moller H,[147] 2013	- Greenland and Nunavut	- 5 Greenlandic and 2 Nunavut communities	- Participant observations, interviews, questionnaires, and reviews (Qualitative)	- Student success depended on nurses and students possessing or having acquired "double culturedness."<br>- Double culturedness refers to the ability to communicate, understand, negotiate and interact, using 2 ways of being in the world and 2 ways of learning and teaching.

Kral MJ, et al.,[143] 2011	- Nunavut	- 50 community members and 66 high school and college students	- Interviews (Qualitative)	- Three themes of well-being: the family, talking/communication, and traditional Inuit cultural values and practices.<br>- Narratives about community and personal change were primarily about family, intergenerational segregation, an increasing population, more trouble in romantic relationships among youth, drug use, and poverty.
**SDH 6: Housing**				
Cassivi A, et al.[141] 2024	- Northern Quebec	- 65 households	- Questionnaire (Quantitative)	- 37% had no individuals consuing tap water; factors included taste of chlorine, fear of contamination, plumbing issues, unacceptable appearance, undesirable temperature.<br>- 50% of households received trucked water 5-6 times per week.<br>- Over 10% found quantity of trucked water insufficient.<br>- 33% experienced water shortage at least once in the previous week.<br>- To cope with water shortage, participants reported reducing laundry frequency, reducing shower length, securing additional sources, and re-using water.
Hansen V, et al.,[135] 2023	- Whitehorse, Yukon	- 232 residential homes	- Radon measurement using Radon Matters (Quantitative)	- 34.0% of surveyed houses had radon concentrations >100 Bq m^{-3}, 20.5% had >200 Bq m^{-3} and 14.4% had >300 Bq m^{-3}.<br>- The estimated annual indoor inhalation dose was 3.0 – 51.0 mSv, compared to the world average of 1.3 mSv.
Pepin C, et al.,[136] 2018	- Northern Quebec	- 220 children (8-15 years) born after at least 35 weeks of pregnancy, weighting a minimum of 2.5 kg, and without major birth defects	- People-per-room ratio, the Center for Epidemiologic Studies Depression Rating Scale for psychological distress measure (Quantitative)	- The results did not show that childhood household crowding had a long-term effect on psychological distress.<br>- Household crowding could be a risk factor only when in interaction with other elements related with poverty or housing or could be experienced as a difficulty for adolescents on other aspects than depressive symptoms and suicidal thoughts.

Ruiz-Castell M, et al.,[138] 2015	- Northern Quebec	- 292 primary caregiver-child dyads	- Interviews (Quantitative)	- Nearly 62% of Inuit families resided in crowded households and were placed at risk for food insecurity.
Daley K, et al.,[140] 2015	- Nunavut	- 28 residents, 9 key informants	- Semi-structured interviews (Qualitative)	- The transition from traditional semi-nomadic living to permanent settlement has influenced current water usage patterns and health and safety perceptions.<br>- Residents are concerned about increased exposure to and risks for waterborne health issues rooted from environmental, social, and cultural factors.
Daley K, et al.,[139] 2014	- Nunavut	- 28 residents, 9 key informants	- Interviews and a review of government water documents (Qualitative)	- Water shortages and services interruptions limit the ability of some households to adhere to public health advice.
Kovesi T, et al.,[134] 2007	- Nunavut	- 49 homes of Inuit children (<5 years)	- Indoor ventilation measure, record of respiratory infection (Quantitative)	- Reported respiratory infection was significantly associated with occupancy (OR 1.81 for each additional occupant, 95% CI 1.14–2.86).<br>- Reduced ventilation and crowding may contribute to the observed excess of lower respiratory tract infection among young Inuit children.
Young TK, et al.,[137] 1996	- NWT and Nunavut	- Unknown	- Data from community-wide housing survey and data relating to physical and social health routinely reported to various service delivery agencies (Qualitative)	- Communities with worse SES are more likely to have a higher rate of health centre visits.<br>- Compared with housing, SES was the stronger factor in predicting the rate of health centre visits.
**SDH 7: Community Infrastructure**				
Akande VO, et al.,[151] 2021	- Nunavut	- 16 Inuit adults	- Photo elicitation interviews and one-on-one-	- Barriers to healthy dietary choices and physical activity participation included limited infrastructure and

			semi-structured interviews (Qualitative)	community resources, affordability and availability of healthy and TF, weather conditions, and social networks.
Baron M, et al.,[150] 2020	- Nunavut	- 20 Inuit (50-86 years)	- Interviews (Qualitative)	- The main resources supporting health included living in houses adapted to aging health conditions, having access to community activities, spending time with children and on the land, and having social support.
Akande VO, et al.,[149] 2019	- Nunavut	- 272 Inuit and non-Inuit adults (18-64 years)	- Seven-day pedometer, survey (Quantitative)	- Physical activity levels represented by steps taken were low.<br>- Improving the external physical environment and internal motivational regulation may improve physical activity levels.

BMI: Body Mass Index; CFP: Community Food Program; CHR: Community health representative; CI: Confidential interval; N/A: Not applicable; NWT: Northwest Territories; OR: Odds ratio; SDH: Social determinants of health; SES: Socioeconomic status; TB; tuberculosis; TF: Traditional foods
