# Supplementary material for: A Scoping Review of the Current Knowledge of the Social Determinants of Health and Infectious Diseases (Specifically COVID-19, Tuberculosis, and H1N1 Influenza) in Canadian Arctic Indigenous Communities
Source: Int J Environ Res Public Health. 2024 Dec 24;22(1):1. doi: 10.3390/ijerph22010001 (PMC11765080; doi:10.3390/ijerph22010001)
Supplement: Supplementary file 1 [file ijerph-22-00001-s001.zip › Table S4. Themes and subthemes under each category and selected quotes from the studies.pdf]

**Table S4. Themes and subthemes under each category and selected quotes from the studies**

| |
|---|
| **Infectious Disease 1: COVID-19** |
| **Theme 1: Regional Differences** |
| "[Northern Canada and Greenland] had no significant proliferation of the pandemic, registered only isolated cases, and posted few or no deaths. These remote territories implemented strict quarantines that included outright travel bans, self-isolation and closures. … However, the areas belonging to this group are highly vulnerable to the COVID-19 pandemic given the remoteness, lack of healthcare infrastructure, and underlying socioeconomic and health issues prevalent in local communities, so these remain of particular concern as the pandemic continues." [4] |
| **Infectious Disease 2: H1N1 Influenza** |
| **Theme 1: Community Response** |
| **Subtheme 1: Community Response** |
| "In general, the communities' pandemic plans were divided into three phases" [44]<br>"By comparing the communities' 1st and 4th generation pandemic plans, our results suggest that there was a vast difference between what was initially outlined in the plans and what their response actually comprised of" [44] |
| **Subtheme 2: Community or Region Specific** |
| "In Community C's communication plan, it was added that, if necessary, practitioners of traditional First Nations medicine provide health teachings" [44]<br>"While adding community specific information to pandemic plans is important, it is also of great value to generally address the unique conditions of a region" [44] |
| **Theme 2: Future Pandemic Plans or Recommendations** |
| "It is recommended that all levels of government collaborate to create a consistent and complementary communication effort, especially when providing guidelines" [42]<br>"Another beneficial modification suggested was to partially close the community's borders, so that a mode of receiving needed supplies and human resources could still function" [43]<br>"Although incorporating expert knowledge is imperative for pandemic planning at the national level, flexibility is required at the community level to allow for plans to be adapted to address communities' realities" [44] |
| **Theme 3: Public Health Response** |
| **Subtheme 1: Bureaucratic Confusion** |
| "In contrast, federal and provincial participants from one community were in disagreement. Although the provincial representative stated that they followed the provided guideline, federal participants believed that the antivirals were not efficiently distributed" [42]<br>"Some participants reported that there was confusion about which health care facility was responsible for receiving and distributing antivirals" [43] |
| **Theme 4: Hospital Admission** |
| **Subtheme 1: Admission Rates** |
| "During 2009 there were 348 admissions for lower respiratory tract infection among 293 infants" [41]<br>"The rate of admission for respiratory syncytial virus infection per 1000 live births in the Northwest Territories was 19.7 compared to 75.3 in Nunavut and 176 in Nunavik, with the highest of 195.1 in the Kitikmeot Region" [41] |
| **Subtheme 2: Contributing Factors** |
| "Longer length of stay was associated with isolation of more than 1 virus versus 1 virus and with the presence of underlying risk factors. Length of stay decreased by 32.2% for every 30-day increase in patient age" [41]<br>"Contributing factors may include lower unemployment rates and higher per-capita income in the Northwest Territories, which influence food security and nutrition and, consequently, rates of lower respiratory tract infection. The proportion of Inuit in Nunavut and Nunavik is 80%-90% compared to 11.1% in the Northwest Territories, which suggests that an important risk factor may be ethnicity" [41] |
| **Theme 5: Pandemic Experience** |
| **Subtheme 1: Effective Strategies** |
| "Participants reported that screening the general public for influenza-like illness using health questionnaires and declarations at public places was successful during [the 2009 H1N1 influenza pandemic]." [43]<br>"The majority of participants said that closing down schools and childcare centres in the community were effective mitigation measures that were employed during their [2009 H1N1 influenza pandemic] response" [43]<br>"Participants generally reported that canceling or postponing events was more effective than simply restricting attendance since no human or other resources were required to screen people attending the event." [43] |
| **Subtheme 2: Ineffective Measures** |

| |
|---|
| "Anecdotal commentary indicated that these measures had low rates of compliance, as some community members doubted the worthiness of these measures, and were difficult to enforce, especially if other travel methods were available (e.g., Winter road, boat)." [43]<br>"For instance, it was noted that one community was only distributed half of their allotted vaccines in a timely manner." [43] |
| **Subtheme 3: Key Informant Perspective** |
| "Participants reported that 30 of the 41 mitigation measures were used in some form or the other during their response to the [2009 H1N1 influenza] pandemic" [43]<br>"Participants reported that mitigation measures were considered to be effective particularly if the measures aided in decreasing virus transmission" [43]<br>"Risk population and increasing community awareness about influenza pandemics. However, participants reported that some of the measures that they considered to be effective were not necessarily feasible to implement given the unique conditions experienced in their communities" [43] |
| **Subtheme 4: Limited Services or Resources** |
| "Representatives from each government body agreed that there was a lack of human resources, especially nurses, during the pandemic response in each study community." [42]<br>"Although lack of required supplies and trained personnel were reported issues" [43]<br>"Shortages of qualified personnel to immunize and lack of adequate education hindered the distribution of vaccines in the communities" [43] |
| **Infectious Disease 3: Tuberculosis (TB)** |
| **Theme 1: Associated Factors** |
| "Unadjusted analyses demonstrated that age, education, smoking tobacco, crowded housing conditions and Inuit ethnicity and statistically significant associations with LTBI status" [45]<br>"After adjustment for all measured SDH, age, crowding, and Inuit ethnicity remained statistically significant determinants of LTBI status" [45]<br>"Older age and receiving a TST during employment screening were associated with non-initiation of treatment while only older age was associated with noncompletion of treatment." [46]<br>"A reduced risk of having a positive result was associated with female sex, non-Inuit ethnicity, and obtaining a TST due to employment screening, school screening or physician or self-referral." [46] |
| **Theme 2: Extend of the problem** |
| **Subtheme 1: Number of Cases** |
| "For 2006-2012, 7213 cases of active TB were reported across the 10 ICS-TB participating jurisdictions. Three ICS-TB jurisdictions had an average annual crude TB IR of >100 cases per 100 000 population: Nunavut, Nunavik, and Greenland" [47]<br>"The highest proportion of laboratory-confirmed cases, including all smear and/or culture-positive cases, was reported in Alaska followed by Yukon, and Nunavut." [47]<br>"19.1% of referred patients were diagnosed with LTBI during a 51-month period in a routine screening program in a remote Canadian artic region with predominantly Inuit population." [46] |
| **Subtheme 2: Treatment** |
| "The proportion of case on an RMP-INH-PZA-EMB (RIPE) treatment regimen varied considerably across jurisdictions. The highest proportion of cases on the RIPE treatment regimens was observed in Greenland, followed by Alaska and the Northwest Territories. The jurisdiction with the lowest proportion of cases on a RIPE regimen was Yukon and Arkhangelsk. Unless drug resistance was detected, the other jurisdictions mainly used different variations of drug combinations of RMP, ING, PZA, EMB and streptomycin" [47]<br>"The most common reason for not offering treatment was discordance between TST and IGRA (46 of 59 patients were not offered treatment. Treatment was started by 246 patients which is 75% of the 328 patients offered treatment and 56% of the 439 patients with LTBI. Treatment was completed by 186 (75.6%) of the 246 patients who initiated treatment. The most common reasons provided for not completing treatment were irregular attendance (16 of 60 non-completers), moving (15 of 60 non-completers) and adverse effects of treatment (14 of 60 non-completers)." [46]<br>"Older age and receiving a TST during employment screening were associated with non-initiation of treatment while only older age was associated with non-completion of treatment" [46] |
| **Theme 3: Intervention** |
| **Subtheme 1: Intervention Type** |
| "In four communities in Nunavut with populations ranging from 500 to 1900, over 40 youth participated in the Taima TB Youth Education Initiative" [48]~~(42)~~ |
| **Subtheme 2: Barriers** |
| "The identified barriers to implementing the research interventions with Inuit youth in remote communities fell into two categories: barriers to youth learning and barriers to local implementation" [48]<br>"Diverse literacy skills within this group made reliable written and video footage evaluation difficult" [48] |

| |
|---|
| "Data for people presenting to health centre for TB testing (passive screening) proved to be impossible to obtain in three of the four communities. Many of the health centres in the project communities do not collect this data or do not distinguish in their data collection between passive and active testing" [48] |
| **Subtheme 3: Future Recommendations** |
| "After the first community, the evaluation process was adapted to include the video interviews used in the other three communities" [48]~~(42)~~<br>"This suggested the videos were a useful way of learning about TB, were relevant to this milieu, offered new information, and could be used for future TB teaching" [48]<br>"Age did seem to affect the results in that the older youth seemed to be fair better suggesting that future studies should likely adapt the activities to a more age specific target group" [48]<br>"Being sensitive to Inuit culture and tradition is important for ensuring that interventions are acceptable to culturally distinct communities." [48] |
| **Subtheme 4: Outcome** |
| "Knowledge uptake by the youth participants was moderate across the communities" [48]<br>"In communities where the initiative was implemented by local health educators, knowledge uptake scores were lower, but still satisfactory. Scores were better in older youth groups compared to the younger youth." [48] |
| **SDH 1: Healthcare Access** |
| **Theme 1: Service Providers** |
| **Subtheme 1: Cultural or Contextual Awareness** |
| "Cultural safety and relationship development between patients and nurses is a key in facilitating patients using primary healthcare services as a preventative measure." [77]<br>"For the most part, they had expected that, because of its remote location and its First Nations population, the community would be "different" in some ways from the communities in which they customarily lived and worked. Indeed, several nurses noted that it was the difference, the opportunity to experience First Nations "cultures" and the sense of "adventure," that had attracted them in the first place. Often, however, the nurses had not been prepared for the contextual contrasts they encountered on arriving in the community. [78]<br>"… the nurses were faced with an exceptionally challenging patient population in terms of complex disease management and co-existing social and health issues. Community residents lived with everyday risk factors for poor health that would be considered unacceptable in mainstream communities, such as overcrowded housing conditions without running water. Nurses were expected to provide primary care in a setting that was defined not only by health and social inequities but also by an overwhelming burden of illness and injury." [78] |
| **Subtheme 2: Limited Number of Providers** |
| "Staff shortages were discussed as a concern in relation to the ability of health care staff to focus on chronic disease care over acute care. As one nurse explained: Right now there's nobody here to run the diabetes program, there's only 2 full-time nurses here right now, so things like immunizations, prenatal, you know acute stuff, gets done before chronic stuff." [79]<br>*Burnout*<br>"The high visibility of CHNs in small communities can lead to staff feeling like they are constantly working, with no separation between personal life and work. Without support from other staff members and clearly defined roles, nurses in remote areas can often feel as though they work 24 hours a day, with no respite. That amount of work for any profession is unadvisable and, in the case of northern medical providers, can lead to poor mental well-being and burnout." [77]<br>"One nurse described her initiation to the work as follows: "When I came here, everything seemed to be stressing me out. It was the isolation, being in a different culture…but the work aspect of it had me so stressed out that it was affecting everything else." Contextual and practice issues such as these contributed to a high turnover rate among nursing staff at this site. A review of relevant documents revealed that over 70 nurses had been employed in the nursing station in the 2-year period immediately preceding data collection." [78]<br>*Turnover*<br>"Understaffing, low retention and a high turnover rate of [primary healthcare service providers] is a chronic persistent problem throughout the [Northwest Territories]." [80]<br>"Staff turnover for remote nursing is recognized as a serious challenge, with vacancy rates between 37 and 57 % across the regions in Nunavut." [81] |
| **Subtheme 3: Limited Training** |
| "Nursing stations are often staffed with "people [who] have less and less skills because there are [no] formal settings to get skills for this type of setting." Participants explained that northern nurses used to graduate from specialized training programs, which prepared them for the demands of work in remote communities. Nursing shortages have forced the communities in [the Sioux Lookout Zone] to rely more on short term replacement nurses (from agencies) who do not necessarily receive such training." [79]<br>"Hiring practices do not always allow for overlap between the previous staff member and the incumbent, "If a position number is filled they can't put another person into that position while that position number is filled so I have to wait until an experienced nurse has left this building to bring an inexperienced nurse in and that's not the way you learn this job" |

| |
|---|
| (Interview 0301). Without overlap time to do on-the-job training, new employees may be left feeling overwhelmed and under-supported.” [77] |
| **Subtheme 4: Patient-Provider Relationships** |
| “Some respondents related instances when they felt that health service providers failed to listen or respond appropriately to patients’ descriptions of symptoms, resulting in negative outcomes for patients.” [83]<br>“According to patients and family members, the most satisfactory interactions with service providers were with individuals who have served for many years in the community and were therefore highly knowledgeable about the language, customs, families and community ways.” [83]~~(50)~~<br>“In the absence of relational engagement, the process of care was characterized by a sense of disengagement within nurse-patient encounters, which was one factor creating gaps in continuity of care.” [78]<br>*Communications*<br>“One participant described examples of Inuit forms of non-verbal communication that might not be properly interpreted by caregivers who lack awareness of local dialect or gestures.” [83]<br>“As the vast majority of Nunavummiut speak Inuktitut as their first language and few health care providers are fluent, language challenges may also contribute to misunderstandings.” [81] |
| **Theme 2: Healthcare Service Availability** |
| **Subtheme 1: General** |
| “The need for public health is highlighted by the rapid changes in lifestyles and the accompanying modern lifestyle-related diseases.” [80] |
| **Subtheme 2: Medical Travel** |
| *Arrangements for family*<br>“Medical travel imposed significant burdens on families and communities in the forms of childcare, missing paid work, time away from family, and the financial costs of travel, only some of which was recouped through health insurance benefits.” [83]<br>“The single biggest challenge reported was making arrangements for children, pets and other household responsibilities while away” [96]<br>*Communication*<br>“Respondents noticed limited information provided before travel, and unclear process of determining itineraries.” [96]<br>*Cost*<br>“During the weeks away from home mothers spent a great deal of money on baby sitters and on telephone calls to their children to reassure themselves. There was the additional cost of airfare if the partner came out too and there was the cost of the partner's time off work or away from hunting to look after children.” [98]<br>*Limited equipment*<br>“In addition to decreasing length of stay, many other undesired side effects of intubation and invasive ventilation are avoided with noninvasive ventilation. These include risks of failed intubation in rural and remote settings with minimal back up and limited equipment, upper airway trauma, barotrauma and volutrauma on the lungs, exposure to sedative medications and paralytics, increased risk of ventilator-associated pneumonias, and increased long-term rates of poor compliance and chronic lung disease.” [99]<br>*Logistics*<br>“Respondents described the impact of delays between obtaining approval and receiving care: ‘By the time [the patient] found out that he had cancer, it had metastasised already. Medical information took forever to reach us.’” [96]<br>“Jurisdiction and responsibility for care Reciprocal billing and aligning policies and procedures in the different health jurisdictions were described as recurrent obstacles. Medical travellers and non- clinical programme staff were unclear about what services were covered, and what to do when patients were turned away.” [96]<br>*Remoteness or weather*<br>“Because of the remote geographic location of most communities, weather can impact medevac decision-making.” [77]<br>*Unfamiliar environment*<br>“These feelings were aggravated by the difficulties of living in a residence with strangers, an unfamiliarly hot environment (summer in the south) with no air conditioning and unfamiliar food.” [98]<br>“Of respondents who reported difficulties, 5.0% identified that travel to an unfamiliar city was the most challenging. The city’s size and range of services was described as overwhelming, especially in comparison to Northern communities. The ability to navigate complicated logistics had an impact on access to care.” [96]<br>*Communication between levels of care*<br>“Limited communication between various levels of care and from decision makers could impact care. Respondents noted that healthcare providers in Edmonton are not always aware of the care that is available in the patients’ home communities. Continuity of care is a challenge given that remote and isolated communities rely on visiting healthcare providers from Southern centres, and there is a high turnover among providers.” [96]<br>*Patient-centred care during travel*<br>“When parents in Community B discussed the events around their baby's birth their perception was that they were allowed to make few choices themselves. They felt the majority of decisions were made by health professionals without their input. Lack of choice was particularly evident for the place of delivery and its conduct.” [98] |

| |
|---|
| “The first had problems when she was unable to get assistance with breast feeding, despite repeated requests for assistance, resulting in engorged breasts and cracked nipples on discharge. Before leaving the city she was unable to find anyone to help her because she was unaware of whom to contact.” [98]<br>“Respondents described the benefit of welcoming and safe healthcare environments that provide high-quality, trauma-informed and culturally appropriate care. Healthcare providers and staff that exhibited cultural competence were described as providing better support to patients.” [96] |
| **Subtheme 3: Specialists through eConsult** |
| “The estimated total societal savings resulting from eConsult in Nunavut were $180,552.73, or $1,100.93 per eConsult. Excluding the costs of added referrals from the cost analysis increased the estimated societal savings to $195,373.71 or $1,191.30 per eConsult.” [95] |
| **Subtheme 4: LGBTQ2S+** |
| “Stakeholders identified the need to better understand the needs of Indigenous LGBTQ youth in the NWT who may not be accessing mainstream LGBTQ-specific community groups or events to the same degree as non-Indigenous LGBTQ youth.” [93]<br>*Stigma, shame, feeling judged*<br>“In the meeting, LGBTQ youth discussed community norms that devalued same sex identities and stigma surrounding LGBTQ-specific services.” [93] |
| **Subtheme 5: Pharmacy** |
| “In remote communities without retail pharmacies, weather delays and retail pharmacy dispensing times can delay pharmacy deliveries by anywhere from two to ten days. These delays in many cases would significantly impact patient care.” [81]<br>“Even in situations when a prescriber is within the community, it has been noted that understaffing (a common issue in the north) and/or high patient loads can cause [community health nurses] to dispense medications from ward stock rather than seek out providers for prescription authorizations.” [81] |
| **SDH 2: Food Insecurity** |
| **Theme 1: Food Insecurity Rates** |
| **Subtheme 1: Household Level** |
| “Food insecurity in general and more severe food insecurity in particular were more prevalent among Inuit households completing the survey than non-Inuit households. Approximately 45% of Inuit households surveyed in both September and May were considered food insecure compared to only 5% of non-Inuit households in September and 4% in May.” [51] |
| **Subtheme 2: Individual Level** |
| “The prevalence of food insecurity and severe food insecurity was higher ($P < 0.001$) among those not completing a secondary education and among those with an income <$20,000 CAD compared with those of greater education and income.” [53]<br>*Community food program users*<br>“More than two thirds of the [community food program user] participants (70%) had experienced times in the last year when there was not enough food at home and it was not possible to access more. Coping strategies at these times included: switching to less preferred and lower quality foods (70%), reducing portions for oneself (64%), reducing portions for others in the household (45%), selling things to access money for food (42%) and sending household members to eat elsewhere (28%).” [59] |
| **Subtheme 3: Suggested strategies to improve food security** |
| “Community members expressed eagerness to find alternatives to both traditional food species and market foods. Long-term food storage and gardening were both suggested as possible solutions to food security in Old Crow.” [50]<br>“Many participants discussed the importance of increasing independence and self-sufficiency so that they didn’t have to rely on food transported from the south. They wanted to advocate for food security initiatives and put some of the responsibility for improving food security towards community leaders.” [54]<br>“Managers, harvesters, and users alike suggested that financially supporting and/or employing harvesters through the community freezer initiative would be an option available to help people get back to the land, while at the same time promoting wild food consumption and helping to maintain a flow of wild foods in their community.” [54] |
| **Theme 2: Food Accessibility** |
| **Subtheme 1: Purchase, Cost, and Affordability** |
| “With the decline in the traditional food supply, the community is eager to consider alternatives. Market foods are available and already form a significant part of the local diet, but share problems with quality and cost with other northern communities. Participants expressed concerns with the nutritional quality of market foods, especially the poor quality of fresh fruit and vegetables. Food costs were another major concern, with this being blamed on Air North freight charges.” [50]<br>“Cost associated with the purchase of fruits, vegetables and dairy products made them inaccessible.” [59] |

| |
|---|
| "Since the community is isolated and primarily accessible only by plane, the cost of purchasing marketed food is high. As a result of the length of time required for transport, variety is limited, the quality of fresh produce is poor, and perishable foods quickly deteriorate in quality." [68]<br>*Limited income*<br>"Household income [was] perceived by many as insufficient to support living/hunting costs." [65]<br>"When asked about the main challenge to achieving a sense of food security at the household level, 35% of participants answered unemployment. Closely following unemployment was income support being too low or not having enough money (26%) and the need to support other members of the family or household crowding (14%). The high cost of food was the main difficulty for 12% of respondents." [58] |
| **Subtheme 2: Social Network or Food Sharing** |
| "When asked how they adapt when there wasn't enough food, the majority of participants mentioned food sharing. Food sharing with family was the most common, followed by food shared between community members and then food shared with friends. Food sharing with family included immediate family as well as relatives, even if the relatives lived in another community. Food sharing was seen as a normal part of daily life and occurred more often during hunting seasons when game meat was made available by hunters. Most of the participants described that the food shared was traditional game meats." [54] |
| **Theme 3: Food Availability** |
| **Subtheme 1: Food Programs** |
| "[Community food programs] were highly valued among users, with 82% reporting that they regularly help alleviate hunger, while the absence of other options for accessing food during times of need was widely noted." [58]<br>"For users, the [community] freezer temporarily connects them to their traditional, land-based lifestyle by making all that is beneficial to them from their traditional territory more accessible." [71]<br>"[Community] gardens were regarded by community members as a means to acquire produce at a cheaper price, especially potatoes, which are favoured but very expensive in Fort Albany." [72]<br>"The school snack/breakfast program emerged as a valued resource that currently supports healthy eating. However, it could be expanded and enhanced with increased resources (e.g. personnel, time, money and food variety)." [68] |
| **Subtheme 2: Hunting or traditional food knowledge** |
| "There was generally concern expressed that the younger generations, along with Vuntut Gwitchin living in Whitehorse, are losing their connection to traditional culture. It was suggested that the school and parents should be encouraged to provide more education in traditional ways—both sharing the harvest and traditional food handling and preparation methods." [50]<br>"The loss of knowledge on how to prepare [traditional food] was identified as a challenge for some; one Photovoice participant explained not knowing how to prepare geese which had been given to her by family members." [59]<br>"Participants from both communities reported that the health of the animals has declined, and the taste and texture of caribou and fish had changed. Participants from Kugaaruk cited the reason as being pollution from exploration, or using wooden storage containers for the fish instead of using ice." [73]<br>"Residents spoke about travelling during unstable/unpredictable ice conditions, which were occurring with greater frequency; this created anxiety and unsafe travel conditions regardless of the financial costs incurred, which led some users to opt out of harvesting activities." [71]<br>"Barriers mentioned included the loss of culture as a reason for no more hunting; less [personal] time for hunting, fishing and cooking traditional food due to employment, and concern about environmental contaminants in hunted food." [54]<br>"The general trend is that harvest levels for some of the most important traditional foods, including caribou, geese, fish, and whales, are declining due to changes in migration patterns and population numbers (availability), changes in the ability to travel to harvesting areas (access), or declines in species health (quality)" [74] |
| **Subtheme 3: Limited food availability** |
| "'Living in the north … we don't always get the right foods, meaning vegetables, fruits, and dairy products within our store. … The meats are not very selective here because we don't know [when] the shipments arrive … by the time we know most of the meat is outdated. So, I think that we are limited here." [55] |
| **SDH 3: Mental Health** |
| **Theme 1: Alcohol and Substance Use** |
| "Although both solvent and alcohol abuse were associated with suicide attempt in the sample as a whole, the link to solvent abuse was much stronger in males and increased the odds of attempt by 4.4. times." [103]<br>"Over the entire 21-year cross section that was considered, communities in Nunavut that prohibited alcohol reported fewer violent crimes compared to those where the importation of alcohol was legal." [107]<br>"Some negative feelings like boredom were associated with negative behaviours, such as alcohol and substance abuse. As one young woman commented, "that's what everyone who's drinking says- they drink 'cause there's nothing else to do… One of my friends always says that he smokes dope so that the day don't seem so long." [112] |
| **Theme 2: Family and Support Networks** |

| |
|---|
| "Suicide attempters were significantly more likely to report parents with a drinking or drug problems, having friends who had attempted or committed suicide, use of solvents in their life time, and having had personal or mental health problems during the previous year." [117]<br>"Inuit more generally spoke about suicide in terms of aloneness, romantic relationship problems, and family relationship problems." [113]<br>"The most common stressor was family conflict/stress (35%, n=38), closely followed by marital relationship stress (e.g. breakup-ups; 26%, n=29)" [108] |
| **Theme 3: Gender** |
| "Results differed by sex. Among males, differences were observed within all three categories of drinking motives (enchantment, social, coping), while only one category adolescent females (enchantment) was revealed to be significant." [105]<br>"The gender divide in suicide mortality was similar in Labrador and newfoundland because males accounted for the majority of deaths (n=110; 85.9% and n=5222; 84.6%) and had a higher ASMR [age standard mortality rates] than did females in both regions." [118]<br>"Distress call were made more frequently by females (n=1,674) than by males (n=1,184)." [119] |
| **Theme 4: Seasonality and climate change** |
| "The arctic months from October to March accounted for (73%) (n=81) of the total referrals, while referrals in the summer months from July to September accounted for only 15% (n=17)." [108] |
| **Theme 5: Use of mental health-related services** |
| "Given that Nunavut has the highest rate of suicide in the world, our results suggest underserved needs rather than lower needs." [110] |
| **SDH 4: Socioeconomic Status (SES)** |
| **Theme 1: Income** |
| "Women described how lack of resources, instability and discrimination affected their ability to make an adequate income. Most women relied on Income Support at levels that do not match the high cost of living in the Arctic." [129]<br>"Using the lowest income category as the reference category (<\$20,000), odds of an at-risk BMI increased with increasing categories of income: OR=1.82 (95% CI 1.47, 2.27) and 3.66 (95% CI 2.57, 5.21) for those \$20,000-60,000, and >\$60,000, respectively." [126]<br>"In the 'poor health' profile… less than one-third had an individual income superior or equal to \$20,000, and lived in a community with a high socio-economic level." [130] |
| **Theme 2: Employment** |
| "For both males and females, employment was associated with significantly greater total energy intake, fibre intake, and lower intake of [traditional foods]." [125]<br>"Employment and income support were both associated with increased traditional food consumption… employment may increase financial ability to purchase traditional foods from hunters or through local networks, and also supports the cost of motor vehicles and fire arms which require fuel, ammunition, and maintenance, hunting has become a costly endeavor." [128]<br>"Higher education, employment, personal income, and private housing were all significantly associated with an at-risk BMI." [126] |
| **Theme 3: Education** |
| "A few noted that they had been encouraged to go back to school, but none suggested that they were currently attending classes. On respondent said: …it's not easy being homeless and trying to go back to school… and not having anything to eat or anywhere to wash your clothes or have a shower." (111)<br>"Many women interviewed had not completed high school… Although a few described completing high school as adults, for many, their current unstable living conditions made it challenging to undertake further education." [129]<br>"For women, acculturation appears to be attended by higher educational attainment which results in lower, rather than higher, risk of obesity and metabolic disease." [125] |
| **SDH 5: Cultural Continuity** |
| **Theme 1: Holism** |
| " A holistic view of a person's ties to land, home, traditions, values, distinctive roles and responsibilities and boundaries/possibilities." [142]<br>"… emphasizes interconnections between the quality of our mental, physical, emotional and spiritual lives." [142] |
| **Theme 2: Relationship** |
| "By far the most prominent theme across all interview questions/questionnaire items, and interrelated with most other themes, was the central importance of family and kinship. Being with family, speaking with family, visiting, going on the land together, sharing food together, and many other family-related activities were closely associated with wellness, happiness, health and healing." [143] |

| |
|---|
| “Increasing levels of community involvement through either sporting events, community events or attending public meetings were statistically associated with a 17% increase in the odds of having good health ($p<0.01$).” [144] |
| **Theme 3: Community voice** |
| “Community members’ shared histories, experiences, language(s) and economy/trades shape how we conceive health, experience health care, develop trust in health care systems and interact with Western medical systems. Access to quality health care for all members of the community is crucial.” [142]<br>“As soon as they see a [familiar] face, it hits close to home.’ Richard, a local hunter and recreational boater, also felt that engaging in safe boating practices was important as a community. He explained, ‘[Safety] comes back to the responsibility of the community as a whole. We can all work together to make this work.” [146] |
| **Theme 4: Cultural responsiveness and consideration** |
| “Cultural sensitivity by promoting a knowledge exchange among health-care workers, researchers, and communities that incorporates a holistic view of the interconnectedness of traditional spiritual and environmental laws and an understanding of the natural order.” [142]<br>“However, because Inuit nurses and students are double-cultured, they are able to negotiate the cultural gaps between Inuit and Southerners, including language, body language and differences in pedagogical approaches.” [147] |
| **Theme 5: Nourishment** |
| “This value recognizes the importance of water and food as nourishment to achieve balanced health, emphasizes local/traditional food and the sharing of food and recognizes the need to use resources wisely and to ensure equitable access.” [142]<br>“Household harvesting activities and satisfaction with access to country food were both positively associated with the odds of better health by 11% and 54%-57%, respectively, with consumption being significantly associated ($p<0.01$).” [144] |
| **Theme 6: Language interpretation** |
| **Subtheme 1: Ethical/cultural dilemmas** |
| “There were significant moral conflicts, especially when the values of the health care providers clashed with those of the communities, families, and interpreters themselves. Interpreters described feeling caught between the expectations of the patient/family on one side and the health care providers/institution on the other. These encounters could produce moments during which no apparent “right” solution would emerge, leaving interpreters feeling isolated in the middle, an experience one described as “a very lonely experience.” Interpreters were left with deep emotional distress and without guidance in how to resolve such conflicts.” [148]<br>“For example, interpreters are often expected by the health care team to transmit news of death and terminal diagnoses. Such tasks are traditionally assigned to community leaders and elders, known locally as Tutsalukkaijiit. These leaders and elders are recognised as having attained the life experience that equips them for the emotional weight of responding to family grief, and the wisdom to guide this process. Several interpreters described feeling overwhelmed when the health care providers assigned them such a task, feeling that they would be contravening local custom and social norms.” [148] |
| **Subtheme 2: Limited training and resources** |
| “Inuit interpreters described lack of linguistic training and resources as a major challenge in their work. While the anatomical knowledge they had attained through hunting, schooling and various media (e.g., television, radio and internet) had served them well, many described their own knowledge as inadequate.” [148]<br>“They also spoke of the need for professional recognition of their services and wished for the opportunity to receive this recognition based on the training and experience they have had while working in the field.” [148] |
| **Subtheme 3: Low job retention** |
| “A management problem common across these diverse health contexts is low job retention and absenteeism of the northern attendants. … [It] is emotionally stressful to interpret difficult news and regulate conflict between patients, families and health care providers; there is little to no on-the-job linguistic training and supplementary interpreting resources; and the wages and benefits are not competitive compared to other local employment (e.g., with schools or the municipality.” [148]<br>“Those who provided interpretation services in these contexts are employed as “préposé en établissement nordique”– or “northern attendants”. These northern attendants speak English and/or French as well as Inuktitut. Their job description includes tasks such as cleaning, sterilising equipment, feeding and washing patients, as well as interpretation.” [148] |
| **SDH 6: Housing** |
| **Theme 1: Contaminatin of houses** |
| “The estimated annual average dose due to inhalation of indoor radon to adults living in the sub-Arctic subdivisions of Whitehorse is higher than the world’s average annual effective dose of 1.3 mSv.”[135]<br>“Six houses (13% of 46) had ventilation below the recommended minimum of 0.35 changes of air per hour” [134]<br>“Houses in Qikiqtaaluk Region were small and crowded relative to most Canadian homes, and they had reduced ventilation both in absolute terms and relative to the number of occupants. Ventilation was reduced in about 80% of the houses evaluated, with mean and peak $CO_2$ levels commonly exceeding recommended values” [134] |

| |
|---|
| “Twenty-one of the children (43%) were reported to have been admitted to hospital in Nunavut and 11 (22%) to have required transfer to a tertiary centre” [134]<br>“Reported lower respiratory tract infection was significantly associated with mean and maximum indoor CO2 concentrations” [134]<br>“Reported asthma was associated with increased maximum indoor CO2 but not with other measures of ventilation” [134] |
| **Theme 2: Overcrowding** |
| **Subtheme 1: Prevalence** |
| “The prevalence of household crowding was high at T1 [between 2005 and 2010], with a little over six out of 10 children living in households characterized by a PPR above 1. This prevalence decreased to four out of 10 at T2 [between 2013 and 2016].” [136]<br>“On average, there were 6.6 individuals per house, with a median of 6 individuals. The prevalence of overcrowding was high, with 6 out of 10 households with more than 1 person per room” [138]<br>“It can be seen that the housing factor is poorly correlated with any of the three outcome variables [morbidity, breach of safety, social problems]” [137] |
| **Subtheme 2: Other Impacts** |
| “[A] paradoxical relationship between housing and number of fires and admission to correctional institutions were observed: the better the housing, the more fired and incarcerations for offences” [137] |
| **Subtheme 3: Physical Health** |
| “The use of health centre visits turned out to serve quite well as an overall indicator of morbidity, being correlated with most housing and SES indicators.” [137] |
| **Subtheme 4: Psychological Health** |
| “At T2, nearly one-third of the adolescents reached a clinical level of depressive symptoms. Also, nearly one out of five adolescents had suicidal thoughts during the year preceding the interview” [136]<br>“Our results did not show any long-term effects of household crowding on adolescents’ psychological distress” [136]<br>“In this study, household overcrowding was not found as a risk factor of psychological distress by itself, but could be part of a wider and complex combination of health determinants of Nunavik Inuit adolescents.” [136] |
| **Theme 3: Water** |
| **Subtheme 1: Water Supply** |
| “Our data collected at the household level, revealed that, while these relatively low domestic water quantities are adequate for some families, those living in overcrowded households are accessing water at levels more typically seen in developing countries such as India, Bangladesh and some East African nations. Although some people are coping by retrieving water independently or sharing between households – as is customary for Inuit – for some subsections of the population, water shortages are limiting their ability to follow public health standards and negatively impacting their overall well-being.” [139] |
| **Subtheme 2: Availability** |
| “That 15 of the 28 households reported being without domestic water at least once per 2-4 weeks.” [139]<br>“Participants did in fact report that households with young children often require more water than they receive” [139]<br>“Our research revealed, however, that many distributions system challenges still exist and that some households in the case study community are not receiving adequate quantities of municipally delivered water.” [139] |
| **Subtheme 3: Dealing with low supply** |
| “Participants identified three coping techniques used to deal with municipal water delivery delays” 1)retrieving their own water, or ice, from local rivers and lakes, 2)relying on neighbours and extended family to share available water and 3)altering their daily activities based on water availability” [139]<br>“Many participants conveyed their preference for independently retrieving water as per traditional practices, regardless of a delay situation, which alleviated their dependence on municipal services” [139]<br>“Despite noting their frustration, some participants accepted that periodically not having water at your home was part of everyday life.” [139] |
| **Subtheme 4: Impact of low supply** |
| “Domestic water delays and shortages exceeded the notion of inconvenience for some participants, and began limiting their ability to adhere to hygiene related public health practices and routines.” [139]<br>“As a result, their health and well-being may be negatively affected if they are not capable of supplementing their domestic water supply by other means. In particular, the consequences of shortages and interruptions are more severe for participants in overcrowded housing, families, with young children households dealing with existing communicable infections.” [139] |
| **SDH 7: Community infrastructure** |
| **Theme 1: Physical resources** |
| “[In] the winter the sidewalks are not as maintained. In the summer you could sort of go anywhere, and everywhere quicker as compared to walking in deep snow, or snow banks to go around” [151] |

| Theme 2: Communication resources |
|---|
| “These types of information are usually relayed through the local FM radio, which used to be the main mode of public communication in Arctic communities …” [150] |

CHN: Community health nurse; ICS-TB: International Circumpolar Surveillance Tuberculosis Working Group; IGRA: Interferon gamma release assay; IR: Incidence rate; LTB: Latent tuberculosis; LTBI: LTB infection; NWT: Northwest Territories; OR: Odds ratio; SDH: Social determinants of health; SES: Socioeconomic status; TB: Tuberculosis; TST: Tuberculin skin test
