# Supplementary material for: A Scoping Review of the Current Knowledge of the Social Determinants of Health and Infectious Diseases (Specifically COVID-19, Tuberculosis, and H1N1 Influenza) in Canadian Arctic Indigenous Communities
Source: Int J Environ Res Public Health. 2024 Dec 24;22(1):1. doi: 10.3390/ijerph22010001 (PMC11765080; doi:10.3390/ijerph22010001)
Supplement: Supplementary file 1 [file ijerph-22-00001-s001.zip › Table S5. Critical appraisal.pdf]

Table S5. Critical appraisal

Appraisal tools used:

1. For quantitative studies: von Elm E, Altman DG, Egger M, Pocock SJ, Gøtzsche PC, Vandenbroucke JP. The Strengthening the Reporting of Observational Studies in Epidemiology (STROBE) statement: guidelines for reporting observational studies. Lancet. 2007;370(9596):1453-7.
2. For qualitative studies: O'Brien BC, Harris IB, Beckman TJ, Reed DA, Cook DA. Standards for reporting qualitative research: a synthesis of recommendations. Acad Med. 2014;89(9):1245-51.
3. For mixed-method studies: Hong QN, Pluye P, Fàbregues S, Bartlett G, Boardman F, Cargo M, Dagenais P, Gagnon M-P, Griffiths F, Nicolau B, O'Cathain A, Rousseau M-C, Vedel I. Mixed Methods Appraisal Tool (MMAT), version 2018. Registration of Copyright (#1148552), Canadian Intellectual Property Office, Industry Canada.

Petrov, A.N.; Welford, M.; Golosov, N.; DeGroote, J.; Degai, T.; Savelyev, A. Spatiotemporal dynamics of the COVID-19 pandemic in the arctic: early data and emerging trends. Int J Circumpolar Health 2020, 79, 1835251, doi:10.1080/22423982.2020.1835251.

STROBE Statement—checklist of items that should be included in reports of observational studies

	Item No	Recommendation	Recommendation met? (Y/N)
**Title and abstract**	1	(*a*) Indicate the study's design with a commonly used term in the title or the abstract	Y
		(*b*) Provide in the abstract an informative and balanced summary of what was done and what was found	Y
**Introduction**			
Background/rationale	2	Explain the scientific background and rationale for the investigation being reported	Y
Objectives	3	State specific objectives, including any prespecified hypotheses	Y
**Methods**			
Study design	4	Present key elements of study design early in the paper	Y
Setting	5	Describe the setting, locations, and relevant dates, including periods of recruitment, exposure, follow-up, and data collection	Y
Participants	6	(*a*) *Cohort study*—Give the eligibility criteria, and the sources and methods of selection of participants. Describe methods of follow-up<br>*Case-control study*—Give the eligibility criteria, and the sources and methods of case ascertainment and control selection. Give the rationale for the choice of cases and controls<br>*Cross-sectional study*—Give the eligibility criteria, and the sources and methods of selection of participants	Y
		(*b*) *Cohort study*—For matched studies, give matching criteria and number of exposed and unexposed<br>*Case-control study*—For matched studies, give matching criteria and the number of controls per case	N/A
Variables	7	Clearly define all outcomes, exposures, predictors, potential confounders, and effect modifiers. Give diagnostic criteria, if applicable	Y
Data sources/ measurement	8*	For each variable of interest, give sources of data and details of methods of assessment (measurement). Describe comparability of assessment methods if there is more than one group	Y
Bias	9	Describe any efforts to address potential sources of bias	N
Study size	10	Explain how the study size was arrived at	N/A
Quantitative variables	11	Explain how quantitative variables were handled in the analyses. If applicable, describe which groupings were chosen and why	N/A
Statistical methods	12	(*a*) Describe all statistical methods, including	N/A

		those used to control for confounding	
		(*b*) Describe any methods used to examine subgroups and interactions	N/A
		(*c*) Explain how missing data were addressed	Y
		(*d*) *Cohort study*—If applicable, explain how loss to follow-up was addressed<br>*Case-control study*—If applicable, explain how matching of cases and controls was addressed<br>*Cross-sectional study*—If applicable, describe analytical methods taking account of sampling strategy	N/A
		(*e*) Describe any sensitivity analyses	N/A
**Results**			
Participants	13*	(a) Report numbers of individuals at each stage of study—eg numbers potentially eligible, examined for eligibility, confirmed eligible, included in the study, completing follow-up, and analysed	Y
		(b) Give reasons for non-participation at each stage	N/A
		(c) Consider use of a flow diagram	N/A
Descriptive data	14*	(a) Give characteristics of study participants (eg demographic, clinical, social) and information on exposures and potential confounders	Y
		(b) Indicate number of participants with missing data for each variable of interest	N/A
		(c) *Cohort study*—Summarise follow-up time (eg, average and total amount)	N/A
Outcome data	15*	*Cohort study*—Report numbers of outcome events or summary measures over time	N/A
		Case-control study—Report numbers in each exposure category, or summary measures of exposure	N/A
		Cross-sectional study—Report numbers of outcome events or summary measures	*Y*
Main results	16	(*a*) Give unadjusted estimates and, if applicable, confounder-adjusted estimates and their precision (eg, 95% confidence interval). Make clear which confounders were adjusted for and why they were included	N/A
		(*b*) Report category boundaries when continuous variables were categorized	N/A
		(*c*) If relevant, consider translating estimates of relative risk into absolute risk for a meaningful time period	N/A
Other analyses	17	Report other analyses done—eg analyses of subgroups and interactions, and sensitivity analyses	N/A
**Discussion**			
Key results	18	Summarise key results with reference to study objectives	Y
Limitations	19	Discuss limitations of the study, taking into account sources of potential bias or imprecision. Discuss both direction and magnitude of any potential bias	Y
Interpretation	20	Give a cautious overall interpretation of results	Y

		considering objectives, limitations, multiplicity of analyses, results from similar studies, and other relevant evidence	
Generalisability	21	Discuss the generalisability (external validity) of the study results	N/A
**Other information**			
Funding	22	Give the source of funding and the role of the funders for the present study and, if applicable, for the original study on which the present article is based	Y

Charania, N.A.; Tsuji, L.J. The 2009 H1N1 pandemic response in remote First Nation communities of Subarctic Ontario: barriers and improvements from a health care services perspective. Int J Circumpolar Health 2011, 70, 564-575, doi:10.3402/ijch.v70i5.17849.

Standards for Reporting Qualitative Research (SRQR)

**No. Topic**	**Item**	**0 – no<br>1 – maybe<br>2-Yes**
**Title and abstract**		
S1 Title	Concise description of the nature and topic of the study identifying the study as qualitative or indicating the approach (e.g., ethnography, grounded theory) or data collection methods (e.g., interview, focus group) is recommended	2
S2 Abstract	Summary of key elements of the study using the abstract format of the intended publication; typically includes objective, methods, results, and conclusions	2
**Introduction**		
S3 Problem formulation	Description and significance of the problem/phenomenon studied; review of relevant theory and empirical work; problem statement	2
S4 Purpose or research question	Purpose of the study and specific objectives or questions	2
**Methods**		
S5 Qualitative approach and research paradigm	Qualitative approach (e.g., ethnography, grounded theory, case study, phenomenology, narrative research) and guiding theory if appropriate; identifying the research paradigm (e.g., positivist, constructivist/interpretivist) is also recommended	0
S6 Researcher characteristics and reflexivity	Researchers' characteristics that may influence the research, including personal attributes, qualifications/experience, relationship with participants, assumptions, or presuppositions; potential or actual interaction between researchers' characteristics and the research questions, approach, methods, results, or transferability	0
S7 Context	Setting/site and salient contextual factors; rationale[a]	2
S8 Sampling strategy	How and why research participants, documents, or events were selected; criteria for deciding when no further sampling was necessary (e.g., sampling saturation); rationale[a]	2
S9 Ethical issues pertaining to human subjects	Documentation of approval by an appropriate ethics review board and participant consent, or explanation for lack thereof; other confidentiality and data security issues	2
S10 Data collection methods	Types of data collected; details of data collection procedures including (as appropriate) start and stop dates of data collection	2

	and analysis, iterative process, triangulation of sources/methods, and modification of procedures in response to evolving study findings; rationale[a]	
S11 Data collection instruments and technologies	Description of instruments (e.g., interview guides, questionnaires) and devices (e.g., audio recorders) used for data collection; if/how the instrument(s) changed over the course of the study	2
S12 Units of study	Number and relevant characteristics of participants, documents, or events included in the study; level of participation (could be reported in results)	2
S13 Data processing	Methods for processing data prior to and during analysis, including transcription, data entry, data management and security, verification of data integrity, data coding, and anonymization/deidentification of excerpts	2
S14 Data analysis	Process by which inferences, themes, etc., were identified and developed, including researchers involved in data analysis; usually references a specific paradigm or approach; rationale[a]	2
S15 Techniques to enhance trustworthiness	Techniques to enhance trustworthiness and credibility of data analysis (e.g., member checking, audit trail, triangulation); rationale[a]	2
**Results/Findings**		
S16 Synthesis and interpretation	Main findings (e.g., interpretations, inferences, and themes); might include development of a theory or model, or integration with prior research or theory	2
S17 Links to empirical data	Evidence (e.g., quotes, field notes, text excerpts, photographs) to substantiate analytic findings	2
**Discussion**		
S18 Integration with prior work, implications, transferability, and contribution(s) to the field	Short summary of main findings; explanation of how findings and conclusions connect to, support, elaborate on, or challenge conclusions of earlier scholarship; discussion of scope of application/generalizability; identification of unique contribution(s) to scholarship in a discipline or field	2
S19 Limitations	Trustworthiness and limitations of findings	0
**Other**		
S20 Conflicts of interest	Potential sources of influence or perceived influence on study conduct and conclusions; how these were managed	0
S21 Funding	Sources of funding and other support; role of funders in data collection, interpretation, and reporting	2

Petrov, A.N.; Welford, M.; Golosov, N.; DeGroote, J.; Devlin, M.; Degai, T.; Savelyev, A. The "second wave" of the COVID-19 pandemic in the Arctic: regional and temporal dynamics. Int J Circumpolar Health 2021, 80, 1925446, doi:10.1080/22423982.2021.1925446.

STROBE Statement—checklist of items that should be included in reports of observational studies

	Item No	Recommendation	Recommendation met? (Y/N)
**Title and abstract**	1	(*a*) Indicate the study's design with a commonly used term in the title or the abstract	Y
		(*b*) Provide in the abstract an informative and balanced summary of what was done and what was found	Y
**Introduction**			
Background/rationale	2	Explain the scientific background and rationale for the investigation being reported	Y
Objectives	3	State specific objectives, including any prespecified hypotheses	Y
**Methods**			
Study design	4	Present key elements of study design early in the paper	Y
Setting	5	Describe the setting, locations, and relevant dates, including periods of recruitment, exposure, follow-up, and data collection	Y
Participants	6	(*a*) *Cohort study*—Give the eligibility criteria, and the sources and methods of selection of participants. Describe methods of follow-up<br>*Case-control study*—Give the eligibility criteria, and the sources and methods of case ascertainment and control selection. Give the rationale for the choice of cases and controls<br>*Cross-sectional study*—Give the eligibility criteria, and the sources and methods of selection of participants	Y
		(*b*) *Cohort study*—For matched studies, give matching criteria and number of exposed and unexposed<br>*Case-control study*—For matched studies, give matching criteria and the number of controls per case	N/A
Variables	7	Clearly define all outcomes, exposures, predictors, potential confounders, and effect modifiers. Give diagnostic criteria, if applicable	Y
Data sources/ measurement	8*	For each variable of interest, give sources of data and details of methods of assessment (measurement). Describe comparability of assessment methods if there is more than one group	Y
Bias	9	Describe any efforts to address potential sources of bias	N
Study size	10	Explain how the study size was arrived at	N/A
Quantitative variables	11	Explain how quantitative variables were handled in the analyses. If applicable, describe which groupings were chosen and why	N/A
Statistical methods	12	(*a*) Describe all statistical methods, including	N/A

		those used to control for confounding	
		(*b*) Describe any methods used to examine subgroups and interactions	N/A
		(*c*) Explain how missing data were addressed	Y
		(*d*) *Cohort study*—If applicable, explain how loss to follow-up was addressed<br>*Case-control study*—If applicable, explain how matching of cases and controls was addressed<br>*Cross-sectional study*—If applicable, describe analytical methods taking account of sampling strategy	N/A
		(*e*) Describe any sensitivity analyses	N/A
**Results**			
Participants	13*	(a) Report numbers of individuals at each stage of study—eg numbers potentially eligible, examined for eligibility, confirmed eligible, included in the study, completing follow-up, and analysed	Y
		(b) Give reasons for non-participation at each stage	N/A
		(c) Consider use of a flow diagram	N/A
Descriptive data	14*	(a) Give characteristics of study participants (eg demographic, clinical, social) and information on exposures and potential confounders	Y
		(b) Indicate number of participants with missing data for each variable of interest	N/A
		(c) *Cohort study*—Summarise follow-up time (eg, average and total amount)	N/A
Outcome data	15*	*Cohort study*—Report numbers of outcome events or summary measures over time	N/A
		Case-control study—Report numbers in each exposure category, or summary measures of exposure	N/A
		Cross-sectional study—Report numbers of outcome events or summary measures	*Y*
Main results	16	(*a*) Give unadjusted estimates and, if applicable, confounder-adjusted estimates and their precision (eg, 95% confidence interval). Make clear which confounders were adjusted for and why they were included	N/A
		(*b*) Report category boundaries when continuous variables were categorized	N/A
		(*c*) If relevant, consider translating estimates of relative risk into absolute risk for a meaningful time period	N/A
Other analyses	17	Report other analyses done—eg analyses of subgroups and interactions, and sensitivity analyses	N/A
**Discussion**			
Key results	18	Summarise key results with reference to study objectives	Y
Limitations	19	Discuss limitations of the study, taking into account sources of potential bias or imprecision. Discuss both direction and magnitude of any potential bias	Y
Interpretation	20	Give a cautious overall interpretation of results	Y

		considering objectives, limitations, multiplicity of analyses, results from similar studies, and other relevant evidence	
Generalisability	21	Discuss the generalisability (external validity) of the study results	N/A
**Other information**			
Funding	22	Give the source of funding and the role of the funders for the present study and, if applicable, for the original study on which the present article is based	Y

Banerji, A.; Panzov, V.; Young, M.; Robinson, J.; Lee, B.; Moraes, T.; Mamdani, M.; Giles, B.L.; Jiang, D.; Bisson, D.; et al. Hospital admissions for lower respiratory tract infections among infants in the Canadian Arctic: a cohort study. CMAJ Open 2016, 4, E615-E622, doi:10.9778/cmajo.20150051.

STROBE Statement—checklist of items that should be included in reports of observational studies

	Item No	Recommendation	Recommendation met? (Y/N)
**Title and abstract**	1	(*a*) Indicate the study's design with a commonly used term in the title or the abstract	Y
		(*b*) Provide in the abstract an informative and balanced summary of what was done and what was found	Y
**Introduction**			
Background/rationale	2	Explain the scientific background and rationale for the investigation being reported	Y
Objectives	3	State specific objectives, including any prespecified hypotheses	Y
**Methods**			
Study design	4	Present key elements of study design early in the paper	Y
Setting	5	Describe the setting, locations, and relevant dates, including periods of recruitment, exposure, follow-up, and data collection	Y
Participants	6	(*a*) *Cohort study*—Give the eligibility criteria, and the sources and methods of selection of participants. Describe methods of follow-up *Case-control study*—Give the eligibility criteria, and the sources and methods of case ascertainment and control selection. Give the rationale for the choice of cases and controls *Cross-sectional study*—Give the eligibility criteria, and the sources and methods of selection of participants	Y
		(*b*) *Cohort study*—For matched studies, give matching criteria and number of exposed and unexposed *Case-control study*—For matched studies, give matching criteria and the number of controls per case	N/A
Variables	7	Clearly define all outcomes, exposures, predictors, potential confounders, and effect modifiers. Give diagnostic criteria, if applicable	Y
Data sources/ measurement	8*	For each variable of interest, give sources of data and details of methods of assessment (measurement). Describe comparability of assessment methods if there is more than one group	Y
Bias	9	Describe any efforts to address potential sources of bias	Y
Study size	10	Explain how the study size was arrived at	Y
Quantitative variables	11	Explain how quantitative variables were handled in the analyses. If applicable, describe which groupings were chosen and why	Y
Statistical methods	12	(*a*) Describe all statistical methods, including	Y

		those used to control for confounding	
		(*b*) Describe any methods used to examine subgroups and interactions	Y
		(*c*) Explain how missing data were addressed	
		(*d*) *Cohort study*—If applicable, explain how loss to follow-up was addressed<br>*Case-control study*—If applicable, explain how matching of cases and controls was addressed<br>*Cross-sectional study*—If applicable, describe analytical methods taking account of sampling strategy	N/A
		(<u>*e*</u>) Describe any sensitivity analyses	Y
**Results**			
Participants	13*	(a) Report numbers of individuals at each stage of study—eg numbers potentially eligible, examined for eligibility, confirmed eligible, included in the study, completing follow-up, and analysed	Y
		(b) Give reasons for non-participation at each stage	N/A
		(c) Consider use of a flow diagram	N/A
Descriptive data	14*	(a) Give characteristics of study participants (eg demographic, clinical, social) and information on exposures and potential confounders	Y
		(b) Indicate number of participants with missing data for each variable of interest	N/A
		(c) *Cohort study*—Summarise follow-up time (eg, average and total amount)	N/A
Outcome data	15*	*Cohort study*—Report numbers of outcome events or summary measures over time	N/A
		Case-control study—Report numbers in each exposure category, or summary measures of exposure	N/A
		Cross-sectional study—Report numbers of outcome events or summary measures	Y
Main results	16	(*a*) Give unadjusted estimates and, if applicable, confounder-adjusted estimates and their precision (eg, 95% confidence interval). Make clear which confounders were adjusted for and why they were included	Y
		(*b*) Report category boundaries when continuous variables were categorized	N/A
		(*c*) If relevant, consider translating estimates of relative risk into absolute risk for a meaningful time period	N/A
Other analyses	17	Report other analyses done—eg analyses of subgroups and interactions, and sensitivity analyses	Y
**Discussion**			
Key results	18	Summarise key results with reference to study objectives	Y
Limitations	19	Discuss limitations of the study, taking into account sources of potential bias or imprecision. Discuss both direction and magnitude of any potential bias	Y
Interpretation	20	Give a cautious overall interpretation of results	Y

		considering objectives, limitations, multiplicity of analyses, results from similar studies, and other relevant evidence	
Generalisability	21	Discuss the generalisability (external validity) of the study results	N/A
**Other information**			
Funding	22	Give the source of funding and the role of the funders for the present study and, if applicable, for the original study on which the present article is based	Y

Charania, N.A.; Tsuji, L.J. Government bodies and their influence on the 2009 H1N1 health sector pandemic response in remote and isolated First Nation communities of sub-Arctic Ontario, Canada. Rural Remote Health 2011, 11, 1781.

Standards for Reporting Qualitative Research (SRQR)

**No. Topic**	**Item**	**0 – no 1 – maybe 2-Yes**
**Title and abstract**		
S1 Title	Concise description of the nature and topic of the study identifying the study as qualitative or indicating the approach (e.g., ethnography, grounded theory) or data collection methods (e.g., interview, focus group) is recommended	2
S2 Abstract	Summary of key elements of the study using the abstract format of the intended publication; typically includes objective, methods, results, and conclusions	2
**Introduction**		
S3 Problem formulation	Description and significance of the problem/phenomenon studied; review of relevant theory and empirical work; problem statement	2
S4 Purpose or research question	Purpose of the study and specific objectives or questions	2
**Methods**		
S5 Qualitative approach and research paradigm	Qualitative approach (e.g., ethnography, grounded theory, case study, phenomenology, narrative research) and guiding theory if appropriate; identifying the research paradigm (e.g., positivist, constructivist/interpretivist) is also recommended	0
S6 Researcher characteristics and reflexivity	Researchers' characteristics that may influence the research, including personal attributes, qualifications/experience, relationship with participants, assumptions, or presuppositions; potential or actual interaction between researchers' characteristics and the research questions, approach, methods, results, or transferability	0
S7 Context	Setting/site and salient contextual factors; rationale[a]	2
S8 Sampling strategy	How and why research participants, documents, or events were selected; criteria for deciding when no further sampling was necessary (e.g., sampling saturation); rationale[a]	2
S9 Ethical issues pertaining to human subjects	Documentation of approval by an appropriate ethics review board and participant consent, or explanation for lack thereof; other confidentiality and data security issues	2
S10 Data collection methods	Types of data collected; details of data collection procedures including (as appropriate) start and stop dates of data collection	2

	and analysis, iterative process, triangulation of sources/methods, and modification of procedures in response to evolving study findings; rationale[a]	
S11 Data collection instruments and technologies	Description of instruments (e.g., interview guides, questionnaires) and devices (e.g., audio recorders) used for data collection; if/how the instrument(s) changed over the course of the study	2
S12 Units of study	Number and relevant characteristics of participants, documents, or events included in the study; level of participation (could be reported in results)	2
S13 Data processing	Methods for processing data prior to and during analysis, including transcription, data entry, data management and security, verification of data integrity, data coding, and anonymization/deidentification of excerpts	2
S14 Data analysis	Process by which inferences, themes, etc., were identified and developed, including researchers involved in data analysis; usually references a specific paradigm or approach; rationale[a]	2
S15 Techniques to enhance trustworthiness	Techniques to enhance trustworthiness and credibility of data analysis (e.g., member checking, audit trail, triangulation); rationale[a]	2
**Results/Findings**		
S16 Synthesis and interpretation	Main findings (e.g., interpretations, inferences, and themes); might include development of a theory or model, or integration with prior research or theory	2
S17 Links to empirical data	Evidence (e.g., quotes, field notes, text excerpts, photographs) to substantiate analytic findings	2
**Discussion**		
S18 Integration with prior work, implications, transferability, and contribution(s) to the field	Short summary of main findings; explanation of how findings and conclusions connect to, support, elaborate on, or challenge conclusions of earlier scholarship; discussion of scope of application/generalizability; identification of unique contribution(s) to scholarship in a discipline or field	2
S19 Limitations	Trustworthiness and limitations of findings	2
**Other**		
S20 Conflicts of interest	Potential sources of influence or perceived influence on study conduct and conclusions; how these were managed	0
S21 Funding	Sources of funding and other support; role of funders in data collection, interpretation, and reporting	2

Charania, N.A.; Tsuji, L.J. Assessing the effectiveness and feasibility of implementing mitigation measures for an influenza pandemic in remote and isolated First Nations communities: a qualitative community-based participatory research approach. Rural Remote Health 2013, 13, 2566.

Standards for Reporting Qualitative Research (SRQR)

**No. Topic**	**Item**	**0 – no 1 – maybe 2-Yes**
**Title and abstract**		
S1 Title	Concise description of the nature and topic of the study identifying the study as qualitative or indicating the approach (e.g., ethnography, grounded theory) or data collection methods (e.g., interview, focus group) is recommended	2
S2 Abstract	Summary of key elements of the study using the abstract format of the intended publication; typically includes objective, methods, results, and conclusions	2
**Introduction**		
S3 Problem formulation	Description and significance of the problem/phenomenon studied; review of relevant theory and empirical work; problem statement	2
S4 Purpose or research question	Purpose of the study and specific objectives or questions	2
**Methods**		
S5 Qualitative approach and research paradigm	Qualitative approach (e.g., ethnography, grounded theory, case study, phenomenology, narrative research) and guiding theory if appropriate; identifying the research paradigm (e.g., positivist, constructivist/interpretivist) is also recommended	0
S6 Researcher characteristics and reflexivity	Researchers' characteristics that may influence the research, including personal attributes, qualifications/experience, relationship with participants, assumptions, or presuppositions; potential or actual interaction between researchers' characteristics and the research questions, approach, methods, results, or transferability	0
S7 Context	Setting/site and salient contextual factors; rationale[a]	2
S8 Sampling strategy	How and why research participants, documents, or events were selected; criteria for deciding when no further sampling was necessary (e.g., sampling saturation); rationale[a]	2
S9 Ethical issues pertaining to human subjects	Documentation of approval by an appropriate ethics review board and participant consent, or explanation for lack thereof; other confidentiality and data security issues	2
S10 Data collection methods	Types of data collected; details of data collection procedures including (as appropriate) start and stop dates of data collection	2

	and analysis, iterative process, triangulation of sources/methods, and modification of procedures in response to evolving study findings; rationale[a]	
S11 Data collection instruments and technologies	Description of instruments (e.g., interview guides, questionnaires) and devices (e.g., audio recorders) used for data collection; if/how the instrument(s) changed over the course of the study	2
S12 Units of study	Number and relevant characteristics of participants, documents, or events included in the study; level of participation (could be reported in results)	2
S13 Data processing	Methods for processing data prior to and during analysis, including transcription, data entry, data management and security, verification of data integrity, data coding, and anonymization/deidentification of excerpts	2
S14 Data analysis	Process by which inferences, themes, etc., were identified and developed, including researchers involved in data analysis; usually references a specific paradigm or approach; rationale[a]	2
S15 Techniques to enhance trustworthiness	Techniques to enhance trustworthiness and credibility of data analysis (e.g., member checking, audit trail, triangulation); rationale[a]	2
**Results/Findings**		
S16 Synthesis and interpretation	Main findings (e.g., interpretations, inferences, and themes); might include development of a theory or model, or integration with prior research or theory	2
S17 Links to empirical data	Evidence (e.g., quotes, field notes, text excerpts, photographs) to substantiate analytic findings	2
**Discussion**		
S18 Integration with prior work, implications, transferability, and contribution(s) to the field	Short summary of main findings; explanation of how findings and conclusions connect to, support, elaborate on, or challenge conclusions of earlier scholarship; discussion of scope of application/generalizability; identification of unique contribution(s) to scholarship in a discipline or field	2
S19 Limitations	Trustworthiness and limitations of findings	2
**Other**		
S20 Conflicts of interest	Potential sources of influence or perceived influence on study conduct and conclusions; how these were managed	0
S21 Funding	Sources of funding and other support; role of funders in data collection, interpretation, and reporting	2

.

Charania, N.A.; Tsuji, L.J. A community-based participatory approach and engagement process creates culturally appropriate and community informed pandemic plans after the 2009 H1N1 influenza pandemic: remote and isolated First Nations communities of sub-arctic Ontario, Canada. BMC Public Health 2012, 12, 268, doi:10.1186/1471-2458-12-268.

Standards for Reporting Qualitative Research (SRQR)

**No. Topic**	**Item**	**0 – no 1 – maybe 2-Yes**
**Title and abstract**		
S1 Title	Concise description of the nature and topic of the study identifying the study as qualitative or indicating the approach (e.g., ethnography, grounded theory) or data collection methods (e.g., interview, focus group) is recommended	2
S2 Abstract	Summary of key elements of the study using the abstract format of the intended publication; typically includes objective, methods, results, and conclusions	2
**Introduction**		
S3 Problem formulation	Description and significance of the problem/phenomenon studied; review of relevant theory and empirical work; problem statement	2
S4 Purpose or research question	Purpose of the study and specific objectives or questions	2
**Methods**		
S5 Qualitative approach and research paradigm	Qualitative approach (e.g., ethnography, grounded theory, case study, phenomenology, narrative research) and guiding theory if appropriate; identifying the research paradigm (e.g., positivist, constructivist/interpretivist) is also recommended	0
S6 Researcher characteristics and reflexivity	Researchers' characteristics that may influence the research, including personal attributes, qualifications/experience, relationship with participants, assumptions, or presuppositions; potential or actual interaction between researchers' characteristics and the research questions, approach, methods, results, or transferability	0
S7 Context	Setting/site and salient contextual factors; rationale[a]	2
S8 Sampling strategy	How and why research participants, documents, or events were selected; criteria for deciding when no further sampling was necessary (e.g., sampling saturation); rationale[a]	2
S9 Ethical issues pertaining to human subjects	Documentation of approval by an appropriate ethics review board and participant consent, or explanation for lack thereof; other confidentiality and data security issues	2
S10 Data collection	Types of data collected; details of data collection procedures	2

methods	including (as appropriate) start and stop dates of data collection and analysis, iterative process, triangulation of sources/methods, and modification of procedures in response to evolving study findings; rationale[a]	
S11 Data collection instruments and technologies	Description of instruments (e.g., interview guides, questionnaires) and devices (e.g., audio recorders) used for data collection; if/how the instrument(s) changed over the course of the study	2
S12 Units of study	Number and relevant characteristics of participants, documents, or events included in the study; level of participation (could be reported in results)	2
S13 Data processing	Methods for processing data prior to and during analysis, including transcription, data entry, data management and security, verification of data integrity, data coding, and anonymization/deidentification of excerpts	2
S14 Data analysis	Process by which inferences, themes, etc., were identified and developed, including researchers involved in data analysis; usually references a specific paradigm or approach; rationale[a]	2
S15 Techniques to enhance trustworthiness	Techniques to enhance trustworthiness and credibility of data analysis (e.g., member checking, audit trail, triangulation); rationale[a]	2
**Results/Findings**		
S16 Synthesis and interpretation	Main findings (e.g., interpretations, inferences, and themes); might include development of a theory or model, or integration with prior research or theory	2
S17 Links to empirical data	Evidence (e.g., quotes, field notes, text excerpts, photographs) to substantiate analytic findings	2
**Discussion**		
S18 Integration with prior work, implications, transferability, and contribution(s) to the field	Short summary of main findings; explanation of how findings and conclusions connect to, support, elaborate on, or challenge conclusions of earlier scholarship; discussion of scope of application/generalizability; identification of unique contribution(s) to scholarship in a discipline or field	2
S19 Limitations	Trustworthiness and limitations of findings	2
**Other**		
S20 Conflicts of interest	Potential sources of influence or perceived influence on study conduct and conclusions; how these were managed	2
S21 Funding	Sources of funding and other support; role of funders in data collection, interpretation, and reporting	2

.

Kilabuk, E.; Momoli, F.; Mallick, R.; Van Dyk, D.; Pease, C.; Zwerling, A.; Potvin, S.E.; Alvarez, G.G. Social determinants of health among residential areas with a high tuberculosis incidence in a remote Inuit community. J Epidemiol Community Health 2019, 73, 401-406, doi:10.1136/jech-2018-211261.

STROBE Statement—checklist of items that should be included in reports of observational studies

	Item No	Recommendation	Recommendation met? (Y/N)
**Title and abstract**	1	(*a*) Indicate the study's design with a commonly used term in the title or the abstract	Y
		(*b*) Provide in the abstract an informative and balanced summary of what was done and what was found	Y
**Introduction**			
Background/rationale	2	Explain the scientific background and rationale for the investigation being reported	Y
Objectives	3	State specific objectives, including any prespecified hypotheses	Y
**Methods**			
Study design	4	Present key elements of study design early in the paper	Y
Setting	5	Describe the setting, locations, and relevant dates, including periods of recruitment, exposure, follow-up, and data collection	Y
Participants	6	(*a*) *Cohort study*—Give the eligibility criteria, and the sources and methods of selection of participants. Describe methods of follow-up *Case-control study*—Give the eligibility criteria, and the sources and methods of case ascertainment and control selection. Give the rationale for the choice of cases and controls *Cross-sectional study*—Give the eligibility criteria, and the sources and methods of selection of participants	Y
		(*b*) *Cohort study*—For matched studies, give matching criteria and number of exposed and unexposed *Case-control study*—For matched studies, give matching criteria and the number of controls per case	N/A
Variables	7	Clearly define all outcomes, exposures, predictors, potential confounders, and effect modifiers. Give diagnostic criteria, if applicable	Y
Data sources/ measurement	8*	For each variable of interest, give sources of data and details of methods of assessment (measurement). Describe comparability of assessment methods if there is more than one group	Y

Bias	9	Describe any efforts to address potential sources of bias	N
Study size	10	Explain how the study size was arrived at	Y
Quantitative variables	11	Explain how quantitative variables were handled in the analyses. If applicable, describe which groupings were chosen and why	Y
Statistical methods	12	(*a*) Describe all statistical methods, including those used to control for confounding	Y
		(*b*) Describe any methods used to examine subgroups and interactions	Y
		(*c*) Explain how missing data were addressed	Y
		(*d*) *Cohort study*—If applicable, explain how loss to follow-up was addressed<br>*Case-control study*—If applicable, explain how matching of cases and controls was addressed<br>*Cross-sectional study*—If applicable, describe analytical methods taking account of sampling strategy	N/A
		(*e*) Describe any sensitivity analyses	Y
**Results**			
Participants	13*	(a) Report numbers of individuals at each stage of study—eg numbers potentially eligible, examined for eligibility, confirmed eligible, included in the study, completing follow-up, and analysed	Y
		(b) Give reasons for non-participation at each stage	Y
		(c) Consider use of a flow diagram	Y
Descriptive data	14*	(a) Give characteristics of study participants (eg demographic, clinical, social) and information on exposures and potential confounders	Y
		(b) Indicate number of participants with missing data for each variable of interest	Y
		(c) *Cohort study*—Summarise follow-up time (eg, average and total amount)	N
Outcome data	15*	*Cohort study*—Report numbers of outcome events or summary measures over time	N/A
		Case-control study—Report numbers in each exposure category, or summary measures of exposure	N/A
		Cross-sectional study—Report numbers of outcome events or summary measures	*Y*
Main results	16	(*a*) Give unadjusted estimates and, if applicable, confounder-adjusted estimates and their precision (eg, 95% confidence interval). Make clear which confounders were adjusted for and why they were included	Y
		(*b*) Report category boundaries when	N/A

		continuous variables were categorized	
		(*c*) If relevant, consider translating estimates of relative risk into absolute risk for a meaningful time period	N/A
Other analyses	17	Report other analyses done—eg analyses of subgroups and interactions, and sensitivity analyses	Y
**Discussion**			
Key results	18	Summarise key results with reference to study objectives	Y
Limitations	19	Discuss limitations of the study, taking into account sources of potential bias or imprecision. Discuss both direction and magnitude of any potential bias	Y
Interpretation	20	Give a cautious overall interpretation of results considering objectives, limitations, multiplicity of analyses, results from similar studies, and other relevant evidence	Y
Generalisability	21	Discuss the generalisability (external validity) of the study results	N
**Other information**			
Funding	22	Give the source of funding and the role of the funders for the present study and, if applicable, for the original study on which the present article is based	Y

Pease, C.; Zwerling, A.; Mallick, R.; Patterson, M.; Demaio, P.; Finn, S.; Allen, J.; Van Dyk, D.; Alvarez, G.G. The latent tuberculosis infection cascade of care in Iqaluit, Nunavut, 2012-2016. BMC Infect Dis 2019, 19, 890, doi:10.1186/s12879-019-4557-3.

STROBE Statement—checklist of items that should be included in reports of observational studies

	Item No	Recommendation	Recommendation met? (Y/N)
**Title and abstract**	1	(*a*) Indicate the study's design with a commonly used term in the title or the abstract	Y
		(*b*) Provide in the abstract an informative and balanced summary of what was done and what was found	Y
**Introduction**			
Background/rationale	2	Explain the scientific background and rationale for the investigation being reported	Y
Objectives	3	State specific objectives, including any prespecified hypotheses	Y
**Methods**			
Study design	4	Present key elements of study design early in the paper	Y
Setting	5	Describe the setting, locations, and relevant dates, including periods of recruitment, exposure, follow-up, and data collection	Y
Participants	6	(*a*) *Cohort study*—Give the eligibility criteria, and the sources and methods of selection of participants. Describe methods of follow-up<br>*Case-control study*—Give the eligibility criteria, and the sources and methods of case ascertainment and control selection. Give the rationale for the choice of cases and controls<br>*Cross-sectional study*—Give the eligibility criteria, and the sources and methods of selection of participants	Y
		(*b*) *Cohort study*—For matched studies, give matching criteria and number of exposed and unexposed<br>*Case-control study*—For matched studies, give matching criteria and the number of controls per case	N/A
Variables	7	Clearly define all outcomes, exposures, predictors, potential confounders, and effect modifiers. Give diagnostic criteria, if applicable	Y
Data sources/ measurement	8*	For each variable of interest, give sources of data and details of methods of assessment (measurement). Describe comparability of assessment methods if there is more than one group	Y

Bias	9	Describe any efforts to address potential sources of bias	N
Study size	10	Explain how the study size was arrived at	Y
Quantitative variables	11	Explain how quantitative variables were handled in the analyses. If applicable, describe which groupings were chosen and why	Y
Statistical methods	12	(*a*) Describe all statistical methods, including those used to control for confounding	Y
		(*b*) Describe any methods used to examine subgroups and interactions	Y
		(*c*) Explain how missing data were addressed	
		(*d*) *Cohort study*—If applicable, explain how loss to follow-up was addressed<br>*Case-control study*—If applicable, explain how matching of cases and controls was addressed<br>*Cross-sectional study*—If applicable, describe analytical methods taking account of sampling strategy	N/A
		(*e*) Describe any sensitivity analyses	Y
**Results**			
Participants	13*	(a) Report numbers of individuals at each stage of study—eg numbers potentially eligible, examined for eligibility, confirmed eligible, included in the study, completing follow-up, and analysed	Y
		(b) Give reasons for non-participation at each stage	N/A
		(c) Consider use of a flow diagram	Y
Descriptive data	14*	(a) Give characteristics of study participants (eg demographic, clinical, social) and information on exposures and potential confounders	Y
		(b) Indicate number of participants with missing data for each variable of interest	Y
		(c) *Cohort study*—Summarise follow-up time (eg, average and total amount)	N/A
Outcome data	15*	*Cohort study*—Report numbers of outcome events or summary measures over time	N/A
		Case-control study—Report numbers in each exposure category, or summary measures of exposure	N/A
		Cross-sectional study—Report numbers of outcome events or summary measures	*Y*
Main results	16	(*a*) Give unadjusted estimates and, if applicable, confounder-adjusted estimates and their precision (eg, 95% confidence interval). Make clear which confounders were adjusted for and why they were included	Y
		(*b*) Report category boundaries when	N/A

		continuous variables were categorized	
		(*c*) If relevant, consider translating estimates of relative risk into absolute risk for a meaningful time period	N/A
Other analyses	17	Report other analyses done—eg analyses of subgroups and interactions, and sensitivity analyses	Y
**Discussion**			
Key results	18	Summarise key results with reference to study objectives	Y
Limitations	19	Discuss limitations of the study, taking into account sources of potential bias or imprecision. Discuss both direction and magnitude of any potential bias	Y
Interpretation	20	Give a cautious overall interpretation of results considering objectives, limitations, multiplicity of analyses, results from similar studies, and other relevant evidence	Y
Generalisability	21	Discuss the generalisability (external validity) of the study results	N
**Other information**			
Funding	22	Give the source of funding and the role of the funders for the present study and, if applicable, for the original study on which the present article is based	Y

Bourgeois, A.C.; Zulz, T.; Bruce, M.G.; Stenz, F.; Koch, A.; Parkinson, A.; Hennessy, T.; Cooper, M.; Newberry, C.; Randell, E.; et al. Tuberculosis in the Circumpolar Region, 2006-2012. Int J Tuberc Lung Dis 2018, 22, 641-648, doi:10.5588/ijtld.17.0525.

STROBE Statement—checklist of items that should be included in reports of observational studies

	Item No	Recommendation	Recommendation met? (Y/N)
**Title and abstract**	1	(*a*) Indicate the study's design with a commonly used term in the title or the abstract	Y
		(*b*) Provide in the abstract an informative and balanced summary of what was done and what was found	Y
**Introduction**			
Background/rationale	2	Explain the scientific background and rationale for the investigation being reported	Y
Objectives	3	State specific objectives, including any prespecified hypotheses	Y
**Methods**			
Study design	4	Present key elements of study design early in the paper	Y
Setting	5	Describe the setting, locations, and relevant dates, including periods of recruitment, exposure, follow-up, and data collection	Y
Participants	6	(*a*) *Cohort study*—Give the eligibility criteria, and the sources and methods of selection of participants. Describe methods of follow-up *Case-control study*—Give the eligibility criteria, and the sources and methods of case ascertainment and control selection. Give the rationale for the choice of cases and controls *Cross-sectional study*—Give the eligibility criteria, and the sources and methods of selection of participants	Y
		(*b*) *Cohort study*—For matched studies, give matching criteria and number of exposed and unexposed *Case-control study*—For matched studies, give matching criteria and the number of controls per case	N/A
Variables	7	Clearly define all outcomes, exposures, predictors, potential confounders, and effect modifiers. Give diagnostic criteria, if applicable	Y
Data sources/ measurement	8*	For each variable of interest, give sources of data and details of methods of assessment (measurement). Describe comparability of assessment methods if there is more than one group	Y

Bias	9	Describe any efforts to address potential sources of bias	Y
Study size	10	Explain how the study size was arrived at	Y
Quantitative variables	11	Explain how quantitative variables were handled in the analyses. If applicable, describe which groupings were chosen and why	Y
Statistical methods	12	(*a*) Describe all statistical methods, including those used to control for confounding	Y
		(*b*) Describe any methods used to examine subgroups and interactions	N/A
		(*c*) Explain how missing data were addressed	
		(*d*) *Cohort study*—If applicable, explain how loss to follow-up was addressed<br>*Case-control study*—If applicable, explain how matching of cases and controls was addressed<br>*Cross-sectional study*—If applicable, describe analytical methods taking account of sampling strategy	N/A
		(*e*) Describe any sensitivity analyses	N/A
**Results**			
Participants	13*	(a) Report numbers of individuals at each stage of study—eg numbers potentially eligible, examined for eligibility, confirmed eligible, included in the study, completing follow-up, and analysed	Y
		(b) Give reasons for non-participation at each stage	N/A
		(c) Consider use of a flow diagram	N/A
Descriptive data	14*	(a) Give characteristics of study participants (eg demographic, clinical, social) and information on exposures and potential confounders	N/A
		(b) Indicate number of participants with missing data for each variable of interest	N/A
		(c) *Cohort study*—Summarise follow-up time (eg, average and total amount)	N/A
Outcome data	15*	*Cohort study*—Report numbers of outcome events or summary measures over time	N/A
		Case-control study—Report numbers in each exposure category, or summary measures of exposure	N/A
		Cross-sectional study—Report numbers of outcome events or summary measures	*Y*
Main results	16	(*a*) Give unadjusted estimates and, if applicable, confounder-adjusted estimates and their precision (eg, 95% confidence interval). Make clear which confounders were adjusted for and why they were included	Y
		(*b*) Report category boundaries when	N/A

		continuous variables were categorized	
		(*c*) If relevant, consider translating estimates of relative risk into absolute risk for a meaningful time period	N/A
Other analyses	17	Report other analyses done—eg analyses of subgroups and interactions, and sensitivity analyses	N/A
**Discussion**			
Key results	18	Summarise key results with reference to study objectives	Y
Limitations	19	Discuss limitations of the study, taking into account sources of potential bias or imprecision. Discuss both direction and magnitude of any potential bias	Y
Interpretation	20	Give a cautious overall interpretation of results considering objectives, limitations, multiplicity of analyses, results from similar studies, and other relevant evidence	Y
Generalisability	21	Discuss the generalisability (external validity) of the study results	N
**Other information**			
Funding	22	Give the source of funding and the role of the funders for the present study and, if applicable, for the original study on which the present article is based	N

Alvarez, G.G.; Van Dyk, D.D.; Colquhoun, H.; Moreau, K.A.; Mulpuru, S.; Graham, I.D. Developing and Field Testing a Community Based Youth Initiative to Increase Tuberculosis Awareness in Remote Arctic Inuit Communities. PLoS One 2016, 11, e0159241, doi:10.1371/journal.pone.0159241.

STROBE Statement—checklist of items that should be included in reports of observational studies

	**Item No**	**Recommendation**	**Recommendation met? (Y/N)**
**Title and abstract**	1	(*a*) Indicate the study's design with a commonly used term in the title or the abstract	Y
		(*b*) Provide in the abstract an informative and balanced summary of what was done and what was found	Y
**Introduction**			
Background/rationale	2	Explain the scientific background and rationale for the investigation being reported	Y
Objectives	3	State specific objectives, including any prespecified hypotheses	Y
**Methods**			
Study design	4	Present key elements of study design early in the paper	Y
Setting	5	Describe the setting, locations, and relevant dates, including periods of recruitment, exposure, follow-up, and data collection	Y
Participants	6	(*a*) *Cohort study*—Give the eligibility criteria, and the sources and methods of selection of participants. Describe methods of follow-up<br>*Case-control study*—Give the eligibility criteria, and the sources and methods of case ascertainment and control selection. Give the rationale for the choice of cases and controls<br>*Cross-sectional study*—Give the eligibility criteria, and the sources and methods of selection of participants	Y
		(*b*) *Cohort study*—For matched studies, give matching criteria and number of exposed and unexposed<br>*Case-control study*—For matched studies, give matching criteria and the number of controls per case	N/A
Variables	7	Clearly define all outcomes, exposures, predictors, potential confounders, and effect modifiers. Give diagnostic criteria, if applicable	Y
Data sources/ measurement	8*	For each variable of interest, give sources of data and details of methods of assessment (measurement). Describe comparability of assessment methods if there is more than one group	Y

Bias	9	Describe any efforts to address potential sources of bias	N/A
Study size	10	Explain how the study size was arrived at	Y
Quantitative variables	11	Explain how quantitative variables were handled in the analyses. If applicable, describe which groupings were chosen and why	Y
Statistical methods	12	(*a*) Describe all statistical methods, including those used to control for confounding	Y
		(*b*) Describe any methods used to examine subgroups and interactions	Y
		(*c*) Explain how missing data were addressed	Y
		(*d*) *Cohort study*—If applicable, explain how loss to follow-up was addressed<br>*Case-control study*—If applicable, explain how matching of cases and controls was addressed<br>*Cross-sectional study*—If applicable, describe analytical methods taking account of sampling strategy	N/A
		(*e*) Describe any sensitivity analyses	N/A
**Results**			
Participants	13*	(a) Report numbers of individuals at each stage of study—eg numbers potentially eligible, examined for eligibility, confirmed eligible, included in the study, completing follow-up, and analysed	Y
		(b) Give reasons for non-participation at each stage	N/A
		(c) Consider use of a flow diagram	N/A
Descriptive data	14*	(a) Give characteristics of study participants (eg demographic, clinical, social) and information on exposures and potential confounders	Y
		(b) Indicate number of participants with missing data for each variable of interest	Y
		(c) *Cohort study*—Summarise follow-up time (eg, average and total amount)	N/A
Outcome data	15*	*Cohort study*—Report numbers of outcome events or summary measures over time	N/A
		Case-control study—Report numbers in each exposure category, or summary measures of exposure	N/A
		Cross-sectional study—Report numbers of outcome events or summary measures	*Y*
Main results	16	(*a*) Give unadjusted estimates and, if applicable, confounder-adjusted estimates and their precision (eg, 95% confidence interval). Make clear which confounders were adjusted for and why they were included	Y
		(*b*) Report category boundaries when	N/A

		continuous variables were categorized	
		(*c*) If relevant, consider translating estimates of relative risk into absolute risk for a meaningful time period	N/A
Other analyses	17	Report other analyses done—eg analyses of subgroups and interactions, and sensitivity analyses	Y
**Discussion**			
Key results	18	Summarise key results with reference to study objectives	Y
Limitations	19	Discuss limitations of the study, taking into account sources of potential bias or imprecision. Discuss both direction and magnitude of any potential bias	Y
Interpretation	20	Give a cautious overall interpretation of results considering objectives, limitations, multiplicity of analyses, results from similar studies, and other relevant evidence	Y
Generalisability	21	Discuss the generalisability (external validity) of the study results	N
**Other information**			
Funding	22	Give the source of funding and the role of the funders for the present study and, if applicable, for the original study on which the present article is based	Y

.

Alvarez, G.G.; Zwerling, A.A.; Duncan, C.; Pease, C.; Van Dyk, D.; Behr, M.A.; Lee, R.S.; Mulpuru, S.; Pakhale, S.; Cameron, D.W.; et al. Molecular Epidemiology of Mycobacterium tuberculosis To Describe the Transmission Dynamics Among Inuit Residing in Iqaluit Nunavut Using Whole-Genome Sequencing. Clin Infect Dis 2021, 72, 2187-2195, doi:10.1093/cid/ciaa420.

STROBE Statement—checklist of items that should be included in reports of observational studies

	Item No	Recommendation	Recommendation met? (Y/N)
**Title and abstract**	1	(*a*) Indicate the study's design with a commonly used term in the title or the abstract	Y
		(*b*) Provide in the abstract an informative and balanced summary of what was done and what was found	Y
**Introduction**			
Background/rationale	2	Explain the scientific background and rationale for the investigation being reported	Y
Objectives	3	State specific objectives, including any prespecified hypotheses	Y
**Methods**			
Study design	4	Present key elements of study design early in the paper	Y
Setting	5	Describe the setting, locations, and relevant dates, including periods of recruitment, exposure, follow-up, and data collection	Y
Participants	6	(*a*) *Cohort study*—Give the eligibility criteria, and the sources and methods of selection of participants. Describe methods of follow-up<br>*Case-control study*—Give the eligibility criteria, and the sources and methods of case ascertainment and control selection. Give the rationale for the choice of cases and controls<br>*Cross-sectional study*—Give the eligibility criteria, and the sources and methods of selection of participants	Y
		(*b*) *Cohort study*—For matched studies, give matching criteria and number of exposed and unexposed<br>*Case-control study*—For matched studies, give matching criteria and the number of controls per case	N/A
Variables	7	Clearly define all outcomes, exposures, predictors, potential confounders, and effect modifiers. Give diagnostic criteria, if applicable	Y
Data sources/ measurement	8*	For each variable of interest, give sources of data and details of methods of assessment (measurement). Describe comparability of assessment methods if there is more than one	Y

		group	
Bias	9	Describe any efforts to address potential sources of bias	N
Study size	10	Explain how the study size was arrived at	Y
Quantitative variables	11	Explain how quantitative variables were handled in the analyses. If applicable, describe which groupings were chosen and why	Y
Statistical methods	12	(*a*) Describe all statistical methods, including those used to control for confounding	Y
		(*b*) Describe any methods used to examine subgroups and interactions	Y
		(*c*) Explain how missing data were addressed	
		(*d*) *Cohort study*—If applicable, explain how loss to follow-up was addressed<br>*Case-control study*—If applicable, explain how matching of cases and controls was addressed<br>*Cross-sectional study*—If applicable, describe analytical methods taking account of sampling strategy	N/A
		(*e*) Describe any sensitivity analyses	N
**Results**			
Participants	13*	(a) Report numbers of individuals at each stage of study—eg numbers potentially eligible, examined for eligibility, confirmed eligible, included in the study, completing follow-up, and analysed	Y
		(b) Give reasons for non-participation at each stage	N/A
		(c) Consider use of a flow diagram	N/A
Descriptive data	14*	(a) Give characteristics of study participants (eg demographic, clinical, social) and information on exposures and potential confounders	Y
		(b) Indicate number of participants with missing data for each variable of interest	Y
		(c) *Cohort study*—Summarise follow-up time (eg, average and total amount)	N/A
Outcome data	15*	*Cohort study*—Report numbers of outcome events or summary measures over time	N/A
		Case-control study—Report numbers in each exposure category, or summary measures of exposure	N/A
		Cross-sectional study—Report numbers of outcome events or summary measures	*Y*
Main results	16	(*a*) Give unadjusted estimates and, if applicable, confounder-adjusted estimates and their precision (eg, 95% confidence interval). Make clear which confounders were adjusted for and why they were included	Y

		(*b*) Report category boundaries when continuous variables were categorized	N/A
		(*c*) If relevant, consider translating estimates of relative risk into absolute risk for a meaningful time period	N/A
Other analyses	17	Report other analyses done—eg analyses of subgroups and interactions, and sensitivity analyses	N/A
**Discussion**			
Key results	18	Summarise key results with reference to study objectives	Y
Limitations	19	Discuss limitations of the study, taking into account sources of potential bias or imprecision. Discuss both direction and magnitude of any potential bias	Y
Interpretation	20	Give a cautious overall interpretation of results considering objectives, limitations, multiplicity of analyses, results from similar studies, and other relevant evidence	Y
Generalisability	21	Discuss the generalisability (external validity) of the study results	N
**Other information**			
Funding	22	Give the source of funding and the role of the funders for the present study and, if applicable, for the original study on which the present article is based	Y

McDonnell, L.; Lavoie, J.G.; Healy, G.; Wong, S.; Goulet, S.; Clark, W. Non-clinical determinants of Medevacs in Nunavut: perspectives from northern health service providers and decision-makers. Int J Circumpolar Health 2019, 78, 1571384, doi:10.1080/22423982.2019.1571384.

Standards for Reporting Qualitative Research (SRQR)

No. Topic	Item	0 – no<br>1 – maybe<br>2-Yes
**Title and abstract**		
S1 Title	Concise description of the nature and topic of the study identifying the study as qualitative or indicating the approach (e.g., ethnography, grounded theory) or data collection methods (e.g., interview, focus group) is recommended	2
S2 Abstract	Summary of key elements of the study using the abstract format of the intended publication; typically includes objective, methods, results, and conclusions	2
**Introduction**		
S3 Problem formulation	Description and significance of the problem/phenomenon studied; review of relevant theory and empirical work; problem statement	2
S4 Purpose or research question	Purpose of the study and specific objectives or questions	2
**Methods**		
S5 Qualitative approach and research paradigm	Qualitative approach (e.g., ethnography, grounded theory, case study, phenomenology, narrative research) and guiding theory if appropriate; identifying the research paradigm (e.g., positivist, constructivist/interpretivist) is also recommended	0
S6 Researcher characteristics and reflexivity	Researchers' characteristics that may influence the research, including personal attributes, qualifications/experience, relationship with participants, assumptions, or presuppositions; potential or actual interaction between researchers' characteristics and the research questions, approach, methods, results, or transferability	0
S7 Context	Setting/site and salient contextual factors; rationale[a]	2
S8 Sampling strategy	How and why research participants, documents, or events were selected; criteria for deciding when no further sampling was necessary (e.g., sampling saturation); rationale[a]	2
S9 Ethical issues pertaining to human subjects	Documentation of approval by an appropriate ethics review board and participant consent, or explanation for lack thereof; other confidentiality and data security issues	2
S10 Data collection methods	Types of data collected; details of data collection procedures including (as appropriate) start and stop dates of data collection	2

	and analysis, iterative process, triangulation of sources/methods, and modification of procedures in response to evolving study findings; rationale[a]	
S11 Data collection instruments and technologies	Description of instruments (e.g., interview guides, questionnaires) and devices (e.g., audio recorders) used for data collection; if/how the instrument(s) changed over the course of the study	2
S12 Units of study	Number and relevant characteristics of participants, documents, or events included in the study; level of participation (could be reported in results)	2
S13 Data processing	Methods for processing data prior to and during analysis, including transcription, data entry, data management and security, verification of data integrity, data coding, and anonymization/deidentification of excerpts	2
S14 Data analysis	Process by which inferences, themes, etc., were identified and developed, including researchers involved in data analysis; usually references a specific paradigm or approach; rationale[a]	2
S15 Techniques to enhance trustworthiness	Techniques to enhance trustworthiness and credibility of data analysis (e.g., member checking, audit trail, triangulation); rationale[a]	2
**Results/Findings**		
S16 Synthesis and interpretation	Main findings (e.g., interpretations, inferences, and themes); might include development of a theory or model, or integration with prior research or theory	2
S17 Links to empirical data	Evidence (e.g., quotes, field notes, text excerpts, photographs) to substantiate analytic findings	2
**Discussion**		
S18 Integration with prior work, implications, transferability, and contribution(s) to the field	Short summary of main findings; explanation of how findings and conclusions connect to, support, elaborate on, or challenge conclusions of earlier scholarship; discussion of scope of application/generalizability; identification of unique contribution(s) to scholarship in a discipline or field	2
S19 Limitations	Trustworthiness and limitations of findings	2
**Other**		
S20 Conflicts of interest	Potential sources of influence or perceived influence on study conduct and conclusions; how these were managed	2
S21 Funding	Sources of funding and other support; role of funders in data collection, interpretation, and reporting	2

Tarlier, D.S.; Browne, A.J.; Johnson, J. The influence of geographical and social distance on nursing practice and continuity of care in a remote First Nations community. Can J Nurs Res 2007, 39, 126-148.

Standards for Reporting Qualitative Research (SRQR)

**No. Topic**	**Item**	**0 – no 1 – maybe 2-Yes**
**Title and abstract**		
S1 Title	Concise description of the nature and topic of the study identifying the study as qualitative or indicating the approach (e.g., ethnography, grounded theory) or data collection methods (e.g., interview, focus group) is recommended	1
S2 Abstract	Summary of key elements of the study using the abstract format of the intended publication; typically includes objective, methods, results, and conclusions	2
**Introduction**		
S3 Problem formulation	Description and significance of the problem/phenomenon studied; review of relevant theory and empirical work; problem statement	2
S4 Purpose or research question	Purpose of the study and specific objectives or questions	2
**Methods**		
S5 Qualitative approach and research paradigm	Qualitative approach (e.g., ethnography, grounded theory, case study, phenomenology, narrative research) and guiding theory if appropriate; identifying the research paradigm (e.g., positivist, constructivist/interpretivist) is also recommended	2
S6 Researcher characteristics and reflexivity	Researchers' characteristics that may influence the research, including personal attributes, qualifications/experience, relationship with participants, assumptions, or presuppositions; potential or actual interaction between researchers' characteristics and the research questions, approach, methods, results, or transferability	2
S7 Context	Setting/site and salient contextual factors; rationale[a]	2
S8 Sampling strategy	How and why research participants, documents, or events were selected; criteria for deciding when no further sampling was necessary (e.g., sampling saturation); rationale[a]	2
S9 Ethical issues pertaining to human subjects	Documentation of approval by an appropriate ethics review board and participant consent, or explanation for lack thereof; other confidentiality and data security issues	2
S10 Data collection methods	Types of data collected; details of data collection procedures including (as appropriate) start and stop dates of data collection and analysis, iterative process, triangulation of sources/methods,	2

	and modification of procedures in response to evolving study findings; rationale[a]	
S11 Data collection instruments and technologies	Description of instruments (e.g., interview guides, questionnaires) and devices (e.g., audio recorders) used for data collection; if/how the instrument(s) changed over the course of the study	2
S12 Units of study	Number and relevant characteristics of participants, documents, or events included in the study; level of participation (could be reported in results)	2
S13 Data processing	Methods for processing data prior to and during analysis, including transcription, data entry, data management and security, verification of data integrity, data coding, and anonymization/deidentification of excerpts	2
S14 Data analysis	Process by which inferences, themes, etc., were identified and developed, including researchers involved in data analysis; usually references a specific paradigm or approach; rationale[a]	2
S15 Techniques to enhance trustworthiness	Techniques to enhance trustworthiness and credibility of data analysis (e.g., member checking, audit trail, triangulation); rationale[a]	2
**Results/Findings**		
S16 Synthesis and interpretation	Main findings (e.g., interpretations, inferences, and themes); might include development of a theory or model, or integration with prior research or theory	2
S17 Links to empirical data	Evidence (e.g., quotes, field notes, text excerpts, photographs) to substantiate analytic findings	2
**Discussion**		
S18 Integration with prior work, implications, transferability, and contribution(s) to the field	Short summary of main findings; explanation of how findings and conclusions connect to, support, elaborate on, or challenge conclusions of earlier scholarship; discussion of scope of application/generalizability; identification of unique contribution(s) to scholarship in a discipline or field	2
S19 Limitations	Trustworthiness and limitations of findings	0
**Other**		
S20 Conflicts of interest	Potential sources of influence or perceived influence on study conduct and conclusions; how these were managed	1
S21 Funding	Sources of funding and other support; role of funders in data collection, interpretation, and reporting	2

Bhattacharyya, O.K.; Estey, E.A.; Rasooly, I.R.; Harris, S.; Zwarenstein, M.; Barnsley, J. Providers' perceptions of barriers to the management of type 2 diabetes in remote Aboriginal settings. Int J Circumpolar Health 2011, 70, 552-563, doi:10.3402/ijch.v70i5.17848.

Standards for Reporting Qualitative Research (SRQR)

**No. Topic**	**Item**	**0 – no 1 – maybe 2-Yes**
**Title and abstract**		
S1 Title	Concise description of the nature and topic of the study identifying the study as qualitative or indicating the approach (e.g., ethnography, grounded theory) or data collection methods (e.g., interview, focus group) is recommended	1
S2 Abstract	Summary of key elements of the study using the abstract format of the intended publication; typically includes objective, methods, results, and conclusions	2
**Introduction**		
S3 Problem formulation	Description and significance of the problem/phenomenon studied; review of relevant theory and empirical work; problem statement	2
S4 Purpose or research question	Purpose of the study and specific objectives or questions	2
**Methods**		
S5 Qualitative approach and research paradigm	Qualitative approach (e.g., ethnography, grounded theory, case study, phenomenology, narrative research) and guiding theory if appropriate; identifying the research paradigm (e.g., positivist, constructivist/interpretivist) is also recommended	2
S6 Researcher characteristics and reflexivity	Researchers' characteristics that may influence the research, including personal attributes, qualifications/experience, relationship with participants, assumptions, or presuppositions; potential or actual interaction between researchers' characteristics and the research questions, approach, methods, results, or transferability	1
S7 Context	Setting/site and salient contextual factors; rationale[a]	2
S8 Sampling strategy	How and why research participants, documents, or events were selected; criteria for deciding when no further sampling was necessary (e.g., sampling saturation); rationale[a]	2
S9 Ethical issues pertaining to human subjects	Documentation of approval by an appropriate ethics review board and participant consent, or explanation for lack thereof; other confidentiality and data security issues	2
S10 Data collection methods	Types of data collected; details of data collection procedures including (as appropriate) start and stop dates of data collection	2

	and analysis, iterative process, triangulation of sources/methods, and modification of procedures in response to evolving study findings; rationale[a]	
S11 Data collection instruments and technologies	Description of instruments (e.g., interview guides, questionnaires) and devices (e.g., audio recorders) used for data collection; if/how the instrument(s) changed over the course of the study	2
S12 Units of study	Number and relevant characteristics of participants, documents, or events included in the study; level of participation (could be reported in results)	2
S13 Data processing	Methods for processing data prior to and during analysis, including transcription, data entry, data management and security, verification of data integrity, data coding, and anonymization/deidentification of excerpts	2
S14 Data analysis	Process by which inferences, themes, etc., were identified and developed, including researchers involved in data analysis; usually references a specific paradigm or approach; rationale[a]	2
S15 Techniques to enhance trustworthiness	Techniques to enhance trustworthiness and credibility of data analysis (e.g., member checking, audit trail, triangulation); rationale[a]	2
**Results/Findings**		
S16 Synthesis and interpretation	Main findings (e.g., interpretations, inferences, and themes); might include development of a theory or model, or integration with prior research or theory	2
S17 Links to empirical data	Evidence (e.g., quotes, field notes, text excerpts, photographs) to substantiate analytic findings	2
**Discussion**		
S18 Integration with prior work, implications, transferability, and contribution(s) to the field	Short summary of main findings; explanation of how findings and conclusions connect to, support, elaborate on, or challenge conclusions of earlier scholarship; discussion of scope of application/generalizability; identification of unique contribution(s) to scholarship in a discipline or field	2
S19 Limitations	Trustworthiness and limitations of findings	2
**Other**		
S20 Conflicts of interest	Potential sources of influence or perceived influence on study conduct and conclusions; how these were managed	2
S21 Funding	Sources of funding and other support; role of funders in data collection, interpretation, and reporting	2

Oosterveer, T.M.; Young, T.K. Primary health care accessibility challenges in remote indigenous communities in Canada's North. Int J Circumpolar Health 2015, 74, 29576, doi:10.3402/ijch.v74.29576.

Standards for Reporting Qualitative Research (SRQR)

No. Topic	Item	0 – no<br>1 – maybe<br>2-Yes
**Title and abstract**		
S1 Title	Concise description of the nature and topic of the study identifying the study as qualitative or indicating the approach (e.g., ethnography, grounded theory) or data collection methods (e.g., interview, focus group) is recommended	2
S2 Abstract	Summary of key elements of the study using the abstract format of the intended publication; typically includes objective, methods, results, and conclusions	2
**Introduction**		
S3 Problem formulation	Description and significance of the problem/phenomenon studied; review of relevant theory and empirical work; problem statement	2
S4 Purpose or research question	Purpose of the study and specific objectives or questions	2
**Methods**		
S5 Qualitative approach and research paradigm	Qualitative approach (e.g., ethnography, grounded theory, case study, phenomenology, narrative research) and guiding theory if appropriate; identifying the research paradigm (e.g., positivist, constructivist/interpretivist) is also recommended	2
S6 Researcher characteristics and reflexivity	Researchers' characteristics that may influence the research, including personal attributes, qualifications/experience, relationship with participants, assumptions, or presuppositions; potential or actual interaction between researchers' characteristics and the research questions, approach, methods, results, or transferability	0
S7 Context	Setting/site and salient contextual factors; rationale[a]	2
S8 Sampling strategy	How and why research participants, documents, or events were selected; criteria for deciding when no further sampling was necessary (e.g., sampling saturation); rationale[a]	2
S9 Ethical issues pertaining to human subjects	Documentation of approval by an appropriate ethics review board and participant consent, or explanation for lack thereof; other confidentiality and data security issues	2
S10 Data collection methods	Types of data collected; details of data collection procedures including (as appropriate) start and stop dates of data collection and analysis, iterative process, triangulation of sources/methods,	2

	and modification of procedures in response to evolving study findings; rationale[a]	
S11 Data collection instruments and technologies	Description of instruments (e.g., interview guides, questionnaires) and devices (e.g., audio recorders) used for data collection; if/how the instrument(s) changed over the course of the study	2
S12 Units of study	Number and relevant characteristics of participants, documents, or events included in the study; level of participation (could be reported in results)	2
S13 Data processing	Methods for processing data prior to and during analysis, including transcription, data entry, data management and security, verification of data integrity, data coding, and anonymization/deidentification of excerpts	2
S14 Data analysis	Process by which inferences, themes, etc., were identified and developed, including researchers involved in data analysis; usually references a specific paradigm or approach; rationale[a]	2
S15 Techniques to enhance trustworthiness	Techniques to enhance trustworthiness and credibility of data analysis (e.g., member checking, audit trail, triangulation); rationale[a]	2
**Results/Findings**		
S16 Synthesis and interpretation	Main findings (e.g., interpretations, inferences, and themes); might include development of a theory or model, or integration with prior research or theory	2
S17 Links to empirical data	Evidence (e.g., quotes, field notes, text excerpts, photographs) to substantiate analytic findings	2
**Discussion**		
S18 Integration with prior work, implications, transferability, and contribution(s) to the field	Short summary of main findings; explanation of how findings and conclusions connect to, support, elaborate on, or challenge conclusions of earlier scholarship; discussion of scope of application/generalizability; identification of unique contribution(s) to scholarship in a discipline or field	2
S19 Limitations	Trustworthiness and limitations of findings	0
**Other**		
S20 Conflicts of interest	Potential sources of influence or perceived influence on study conduct and conclusions; how these were managed	2
S21 Funding	Sources of funding and other support; role of funders in data collection, interpretation, and reporting	2

Romain, S.J.; Kohler, J.C.; Young, K. Policy versus practice: a community-based qualitative study of the realities of pharmacy services in Nunavut, Canada. J Pharm Policy Pract 2015, 8, 22,

Standards for Reporting Qualitative Research (SRQR)

No. Topic	Item	0 – no 1 – maybe 2-Yes
**Title and abstract**		
S1 Title	Concise description of the nature and topic of the study identifying the study as qualitative or indicating the approach (e.g., ethnography, grounded theory) or data collection methods (e.g., interview, focus group) is recommended	2
S2 Abstract	Summary of key elements of the study using the abstract format of the intended publication; typically includes objective, methods, results, and conclusions	2
**Introduction**		
S3 Problem formulation	Description and significance of the problem/phenomenon studied; review of relevant theory and empirical work; problem statement	2
S4 Purpose or research question	Purpose of the study and specific objectives or questions	2
**Methods**		
S5 Qualitative approach and research paradigm	Qualitative approach (e.g., ethnography, grounded theory, case study, phenomenology, narrative research) and guiding theory if appropriate; identifying the research paradigm (e.g., positivist, constructivist/interpretivist) is also recommended	2
S6 Researcher characteristics and reflexivity	Researchers' characteristics that may influence the research, including personal attributes, qualifications/experience, relationship with participants, assumptions, or presuppositions; potential or actual interaction between researchers' characteristics and the research questions, approach, methods, results, or transferability	0
S7 Context	Setting/site and salient contextual factors; rationale[a]	2
S8 Sampling strategy	How and why research participants, documents, or events were selected; criteria for deciding when no further sampling was necessary (e.g., sampling saturation); rationale[a]	2
S9 Ethical issues pertaining to human subjects	Documentation of approval by an appropriate ethics review board and participant consent, or explanation for lack thereof; other confidentiality and data security issues	2
S10 Data collection methods	Types of data collected; details of data collection procedures including (as appropriate) start and stop dates of data collection and analysis, iterative process, triangulation of sources/methods,	2

	and modification of procedures in response to evolving study findings; rationale[a]	
S11 Data collection instruments and technologies	Description of instruments (e.g., interview guides, questionnaires) and devices (e.g., audio recorders) used for data collection; if/how the instrument(s) changed over the course of the study	2
S12 Units of study	Number and relevant characteristics of participants, documents, or events included in the study; level of participation (could be reported in results)	2
S13 Data processing	Methods for processing data prior to and during analysis, including transcription, data entry, data management and security, verification of data integrity, data coding, and anonymization/deidentification of excerpts	2
S14 Data analysis	Process by which inferences, themes, etc., were identified and developed, including researchers involved in data analysis; usually references a specific paradigm or approach; rationale[a]	2
S15 Techniques to enhance trustworthiness	Techniques to enhance trustworthiness and credibility of data analysis (e.g., member checking, audit trail, triangulation); rationale[a]	2
**Results/Findings**		
S16 Synthesis and interpretation	Main findings (e.g., interpretations, inferences, and themes); might include development of a theory or model, or integration with prior research or theory	2
S17 Links to empirical data	Evidence (e.g., quotes, field notes, text excerpts, photographs) to substantiate analytic findings	2
**Discussion**		
S18 Integration with prior work, implications, transferability, and contribution(s) to the field	Short summary of main findings; explanation of how findings and conclusions connect to, support, elaborate on, or challenge conclusions of earlier scholarship; discussion of scope of application/generalizability; identification of unique contribution(s) to scholarship in a discipline or field	2
S19 Limitations	Trustworthiness and limitations of findings	0
**Other**		
S20 Conflicts of interest	Potential sources of influence or perceived influence on study conduct and conclusions; how these were managed	2
S21 Funding	Sources of funding and other support; role of funders in data collection, interpretation, and reporting	2

Young, T.K.; Fedkina, N.; Chatwood, S.; Bjerregaard, P. Comparing health care workforce in circumpolar regions: patterns, trends and challenges. Int J Circumpolar Health 2018, 77, 1492825, doi:10.1080/22423982.2018.1492825.

STROBE Statement—checklist of items that should be included in reports of observational studies

	Item No	Recommendation	Recommendation met? (Y/N)
**Title and abstract**	1	(*a*) Indicate the study's design with a commonly used term in the title or the abstract	Y
		(*b*) Provide in the abstract an informative and balanced summary of what was done and what was found	Y
**Introduction**			
Background/rationale	2	Explain the scientific background and rationale for the investigation being reported	Y
Objectives	3	State specific objectives, including any prespecified hypotheses	Y
**Methods**			
Study design	4	Present key elements of study design early in the paper	Y
Setting	5	Describe the setting, locations, and relevant dates, including periods of recruitment, exposure, follow-up, and data collection	Y
Participants	6	(*a*) *Cohort study*—Give the eligibility criteria, and the sources and methods of selection of participants. Describe methods of follow-up<br>*Case-control study*—Give the eligibility criteria, and the sources and methods of case ascertainment and control selection. Give the rationale for the choice of cases and controls<br>*Cross-sectional study*—Give the eligibility criteria, and the sources and methods of selection of participants	Y
		(*b*) *Cohort study*—For matched studies, give matching criteria and number of exposed and unexposed<br>*Case-control study*—For matched studies, give matching criteria and the number of controls per case	N/A
Variables	7	Clearly define all outcomes, exposures, predictors, potential confounders, and effect modifiers. Give diagnostic criteria, if applicable	Y
Data sources/ measurement	8*	For each variable of interest, give sources of data and details of methods of assessment (measurement). Describe comparability of assessment methods if there is more than one	Y

		group	
Bias	9	Describe any efforts to address potential sources of bias	N
Study size	10	Explain how the study size was arrived at	N/A
Quantitative variables	11	Explain how quantitative variables were handled in the analyses. If applicable, describe which groupings were chosen and why	N
Statistical methods	12	(*a*) Describe all statistical methods, including those used to control for confounding	N/A
		(*b*) Describe any methods used to examine subgroups and interactions	N/A
		(*c*) Explain how missing data were addressed	N/A
		(*d*) *Cohort study*—If applicable, explain how loss to follow-up was addressed<br>*Case-control study*—If applicable, explain how matching of cases and controls was addressed<br>*Cross-sectional study*—If applicable, describe analytical methods taking account of sampling strategy	N/A
		(*e*) Describe any sensitivity analyses	N/A
**Results**			
Participants	13*	(a) Report numbers of individuals at each stage of study—eg numbers potentially eligible, examined for eligibility, confirmed eligible, included in the study, completing follow-up, and analysed	N/A
		(b) Give reasons for non-participation at each stage	N/A
		(c) Consider use of a flow diagram	N/A
Descriptive data	14*	(a) Give characteristics of study participants (eg demographic, clinical, social) and information on exposures and potential confounders	Y
		(b) Indicate number of participants with missing data for each variable of interest	N/A
		(c) *Cohort study*—Summarise follow-up time (eg, average and total amount)	N/A
Outcome data	15*	*Cohort study*—Report numbers of outcome events or summary measures over time	N/A
		Case-control study—Report numbers in each exposure category, or summary measures of exposure	N/A
		Cross-sectional study—Report numbers of outcome events or summary measures	*Y*
Main results	16	(*a*) Give unadjusted estimates and, if applicable, confounder-adjusted estimates and their precision (eg, 95% confidence interval). Make clear which confounders were adjusted for and why they were included	N/A

		(*b*) Report category boundaries when continuous variables were categorized	N/A
		(*c*) If relevant, consider translating estimates of relative risk into absolute risk for a meaningful time period	N/A
Other analyses	17	Report other analyses done—eg analyses of subgroups and interactions, and sensitivity analyses	N/A
**Discussion**			
Key results	18	Summarise key results with reference to study objectives	Y
Limitations	19	Discuss limitations of the study, taking into account sources of potential bias or imprecision. Discuss both direction and magnitude of any potential bias	Y
Interpretation	20	Give a cautious overall interpretation of results considering objectives, limitations, multiplicity of analyses, results from similar studies, and other relevant evidence	Y
Generalisability	21	Discuss the generalisability (external validity) of the study results	N/A
**Other information**			
Funding	22	Give the source of funding and the role of the funders for the present study and, if applicable, for the original study on which the present article is based	Y

Galloway, T.; Horlick, S.; Cherba, M.; Cole, M.; Woodgate, R.L.; Healey Akearok, G. Perspectives of Nunavut patients and families on their cancer and end of life care experiences. Int J Circumpolar Health 2020, 79, 1766319, doi:10.1080/22423982.2020.1766319.

Standards for Reporting Qualitative Research (SRQR)

**No. Topic**	**Item**	**0 – no 1 – maybe 2-Yes**
**Title and abstract**		
S1 Title	Concise description of the nature and topic of the study identifying the study as qualitative or indicating the approach (e.g., ethnography, grounded theory) or data collection methods (e.g., interview, focus group) is recommended	2
S2 Abstract	Summary of key elements of the study using the abstract format of the intended publication; typically includes objective, methods, results, and conclusions	2
**Introduction**		
S3 Problem formulation	Description and significance of the problem/phenomenon studied; review of relevant theory and empirical work; problem statement	2
S4 Purpose or research question	Purpose of the study and specific objectives or questions	2
**Methods**		
S5 Qualitative approach and research paradigm	Qualitative approach (e.g., ethnography, grounded theory, case study, phenomenology, narrative research) and guiding theory if appropriate; identifying the research paradigm (e.g., positivist, constructivist/interpretivist) is also recommended	2
S6 Researcher characteristics and reflexivity	Researchers' characteristics that may influence the research, including personal attributes, qualifications/experience, relationship with participants, assumptions, or presuppositions; potential or actual interaction between researchers' characteristics and the research questions, approach, methods, results, or transferability	1
S7 Context	Setting/site and salient contextual factors; rationale[a]	2
S8 Sampling strategy	How and why research participants, documents, or events were selected; criteria for deciding when no further sampling was necessary (e.g., sampling saturation); rationale[a]	2
S9 Ethical issues pertaining to human subjects	Documentation of approval by an appropriate ethics review board and participant consent, or explanation for lack thereof; other confidentiality and data security issues	2
S10 Data collection methods	Types of data collected; details of data collection procedures including (as appropriate) start and stop dates of data collection	2

	and analysis, iterative process, triangulation of sources/methods, and modification of procedures in response to evolving study findings; rationale[a]	
S11 Data collection instruments and technologies	Description of instruments (e.g., interview guides, questionnaires) and devices (e.g., audio recorders) used for data collection; if/how the instrument(s) changed over the course of the study	2
S12 Units of study	Number and relevant characteristics of participants, documents, or events included in the study; level of participation (could be reported in results)	2
S13 Data processing	Methods for processing data prior to and during analysis, including transcription, data entry, data management and security, verification of data integrity, data coding, and anonymization/deidentification of excerpts	2
S14 Data analysis	Process by which inferences, themes, etc., were identified and developed, including researchers involved in data analysis; usually references a specific paradigm or approach; rationale[a]	2
S15 Techniques to enhance trustworthiness	Techniques to enhance trustworthiness and credibility of data analysis (e.g., member checking, audit trail, triangulation); rationale[a]	2
**Results/Findings**		
S16 Synthesis and interpretation	Main findings (e.g., interpretations, inferences, and themes); might include development of a theory or model, or integration with prior research or theory	2
S17 Links to empirical data	Evidence (e.g., quotes, field notes, text excerpts, photographs) to substantiate analytic findings	2
**Discussion**		
S18 Integration with prior work, implications, transferability, and contribution(s) to the field	Short summary of main findings; explanation of how findings and conclusions connect to, support, elaborate on, or challenge conclusions of earlier scholarship; discussion of scope of application/generalizability; identification of unique contribution(s) to scholarship in a discipline or field	2
S19 Limitations	Trustworthiness and limitations of findings	2
**Other**		
S20 Conflicts of interest	Potential sources of influence or perceived influence on study conduct and conclusions; how these were managed	2
S21 Funding	Sources of funding and other support; role of funders in data collection, interpretation, and reporting	2

Bird, S.M.; Wiles, J.L.; Okalik, L.; Kilabuk, J.; Egeland, G.M. Living with diabetes on Baffin Island: Inuit storytellers share their experiences. Can J Public Health 2008, 99, 17-21, doi:10.1007/bf03403734.

Standards for Reporting Qualitative Research (SRQR)

No. Topic	Item	0 – no 1 – maybe 2-Yes
**Title and abstract**		
S1 Title	Concise description of the nature and topic of the study identifying the study as qualitative or indicating the approach (e.g., ethnography, grounded theory) or data collection methods (e.g., interview, focus group) is recommended	2
S2 Abstract	Summary of key elements of the study using the abstract format of the intended publication; typically includes objective, methods, results, and conclusions	2
**Introduction**		
S3 Problem formulation	Description and significance of the problem/phenomenon studied; review of relevant theory and empirical work; problem statement	2
S4 Purpose or research question	Purpose of the study and specific objectives or questions	2
**Methods**		
S5 Qualitative approach and research paradigm	Qualitative approach (e.g., ethnography, grounded theory, case study, phenomenology, narrative research) and guiding theory if appropriate; identifying the research paradigm (e.g., positivist, constructivist/interpretivist) is also recommended	0
S6 Researcher characteristics and reflexivity	Researchers' characteristics that may influence the research, including personal attributes, qualifications/experience, relationship with participants, assumptions, or presuppositions; potential or actual interaction between researchers' characteristics and the research questions, approach, methods, results, or transferability	2
S7 Context	Setting/site and salient contextual factors; rationale[a]	2
S8 Sampling strategy	How and why research participants, documents, or events were selected; criteria for deciding when no further sampling was necessary (e.g., sampling saturation); rationale[a]	2
S9 Ethical issues pertaining to human subjects	Documentation of approval by an appropriate ethics review board and participant consent, or explanation for lack thereof; other confidentiality and data security issues	2
S10 Data collection methods	Types of data collected; details of data collection procedures including (as appropriate) start and stop dates of data collection and analysis, iterative process, triangulation of sources/methods, and modification of procedures in response to evolving study	1

	findings; rationale[a]	
S11 Data collection instruments and technologies	Description of instruments (e.g., interview guides, questionnaires) and devices (e.g., audio recorders) used for data collection; if/how the instrument(s) changed over the course of the study	2
S12 Units of study	Number and relevant characteristics of participants, documents, or events included in the study; level of participation (could be reported in results)	2
S13 Data processing	Methods for processing data prior to and during analysis, including transcription, data entry, data management and security, verification of data integrity, data coding, and anonymization/deidentification of excerpts	2
S14 Data analysis	Process by which inferences, themes, etc., were identified and developed, including researchers involved in data analysis; usually references a specific paradigm or approach; rationale[a]	2
S15 Techniques to enhance trustworthiness	Techniques to enhance trustworthiness and credibility of data analysis (e.g., member checking, audit trail, triangulation); rationale[a]	2
**Results/Findings**		
S16 Synthesis and interpretation	Main findings (e.g., interpretations, inferences, and themes); might include development of a theory or model, or integration with prior research or theory	2
S17 Links to empirical data	Evidence (e.g., quotes, field notes, text excerpts, photographs) to substantiate analytic findings	2
**Discussion**		
S18 Integration with prior work, implications, transferability, and contribution(s) to the field	Short summary of main findings; explanation of how findings and conclusions connect to, support, elaborate on, or challenge conclusions of earlier scholarship; discussion of scope of application/generalizability; identification of unique contribution(s) to scholarship in a discipline or field	2
S19 Limitations	Trustworthiness and limitations of findings	2
**Other**		
S20 Conflicts of interest	Potential sources of influence or perceived influence on study conduct and conclusions; how these were managed	2
S21 Funding	Sources of funding and other support; role of funders in data collection, interpretation, and reporting	2

Logie, C.H.; Lys, C.L.; Dias, L.; Schott, N.; Zouboules, M.R.; MacNeill, N.; Mackay, K. "Automatic assumption of your gender, sexuality and sexual practices is also discrimination": Exploring sexual healthcare experiences and recommendations among sexually and gender diverse persons in Arctic Canada. Health Soc Care Community 2019, 27, 1204-1213, doi:10.1111/hsc.12757.

Standards for Reporting Qualitative Research (SRQR)

**No. Topic**	**Item**	**0 – no<br>1 – maybe<br>2-Yes**
**Title and abstract**		
S1 Title	Concise description of the nature and topic of the study identifying the study as qualitative or indicating the approach (e.g., ethnography, grounded theory) or data collection methods (e.g., interview, focus group) is recommended	2
S2 Abstract	Summary of key elements of the study using the abstract format of the intended publication; typically includes objective, methods, results, and conclusions	2
**Introduction**		
S3 Problem formulation	Description and significance of the problem/phenomenon studied; review of relevant theory and empirical work; problem statement	2
S4 Purpose or research question	Purpose of the study and specific objectives or questions	2
**Methods**		
S5 Qualitative approach and research paradigm	Qualitative approach (e.g., ethnography, grounded theory, case study, phenomenology, narrative research) and guiding theory if appropriate; identifying the research paradigm (e.g., positivist, constructivist/interpretivist) is also recommended	0
S6 Researcher characteristics and reflexivity	Researchers' characteristics that may influence the research, including personal attributes, qualifications/experience, relationship with participants, assumptions, or presuppositions; potential or actual interaction between researchers' characteristics and the research questions, approach, methods, results, or transferability	0
S7 Context	Setting/site and salient contextual factors; rationale[a]	2
S8 Sampling strategy	How and why research participants, documents, or events were selected; criteria for deciding when no further sampling was necessary (e.g., sampling saturation); rationale[a]	2
S9 Ethical issues pertaining to human subjects	Documentation of approval by an appropriate ethics review board and participant consent, or explanation for lack thereof; other confidentiality and data security issues	2
S10 Data collection	Types of data collected; details of data collection procedures	2

methods	including (as appropriate) start and stop dates of data collection and analysis, iterative process, triangulation of sources/methods, and modification of procedures in response to evolving study findings; rationale[a]	
S11 Data collection instruments and technologies	Description of instruments (e.g., interview guides, questionnaires) and devices (e.g., audio recorders) used for data collection; if/how the instrument(s) changed over the course of the study	2
S12 Units of study	Number and relevant characteristics of participants, documents, or events included in the study; level of participation (could be reported in results)	2
S13 Data processing	Methods for processing data prior to and during analysis, including transcription, data entry, data management and security, verification of data integrity, data coding, and anonymization/deidentification of excerpts	2
S14 Data analysis	Process by which inferences, themes, etc., were identified and developed, including researchers involved in data analysis; usually references a specific paradigm or approach; rationale[a]	2
S15 Techniques to enhance trustworthiness	Techniques to enhance trustworthiness and credibility of data analysis (e.g., member checking, audit trail, triangulation); rationale[a]	2
**Results/Findings**		
S16 Synthesis and interpretation	Main findings (e.g., interpretations, inferences, and themes); might include development of a theory or model, or integration with prior research or theory	2
S17 Links to empirical data	Evidence (e.g., quotes, field notes, text excerpts, photographs) to substantiate analytic findings	2
**Discussion**		
S18 Integration with prior work, implications, transferability, and contribution(s) to the field	Short summary of main findings; explanation of how findings and conclusions connect to, support, elaborate on, or challenge conclusions of earlier scholarship; discussion of scope of application/generalizability; identification of unique contribution(s) to scholarship in a discipline or field	2
S19 Limitations	Trustworthiness and limitations of findings	2
**Other**		
S20 Conflicts of interest	Potential sources of influence or perceived influence on study conduct and conclusions; how these were managed	0
S21 Funding	Sources of funding and other support; role of funders in data collection, interpretation, and reporting	2

Hansen, N.; Jensen, K.; MacNiven, I.; Pollock, N.; D'Hont, T.; Chatwood, S. Exploring the impact of rural health system factors on physician burnout: a mixed-methods study in Northern Canada. BMC Health Serv Res 2021, 21, 869, doi:10.1186/s12913-021-06899-y.

Mixed Methods Appraisal Tool (MMAT), version 2018

**Category of study designs**	**Methodological quality criteria**	**Responses**			
		Yes	No	Can't tell	Comments
Screening questions (for all types)	S1. Are there clear research questions?	X			
	S2. Do the collected data allow to address the research questions?	X			
	Further appraisal may not be feasible or appropriate when the answer is 'No' or 'Can't tell' to one or both screening questions.				
1. Qualitative	1.1. Is the qualitative approach appropriate to answer the research question?	X			
	1.2. Are the qualitative data collection methods adequate to address the research question?	X			
	1.3. Are the findings adequately derived from the data?	X			
	1.4. Is the interpretation of results sufficiently substantiated by data?	X			
	1.5. Is there coherence between qualitative data sources, collection, analysis and interpretation?	X			
2. Quantitative randomized controlled trials	2.1. Is randomization appropriately performed?	N/A			
	2.2. Are the groups comparable at baseline?	N/A			
	2.3. Are there complete outcome data?	N/A			
	2.4. Are outcome assessors blinded to the intervention provided?	N/A			
	2.5 Did the participants adhere to the assigned intervention?	N/A			
3. Quantitative non-randomized	3.1. Are the participants representative of the target population?	N/A			
	3.2. Are measurements appropriate regarding both the outcome and intervention (or exposure)?	N/A			
	3.3. Are there complete outcome data?	N/A			
	3.4. Are the confounders accounted for in the design and analysis?	N/A			
	3.5. During the study period, is the intervention administered (or exposure occurred) as intended?	N/A			
4. Quantitative descriptive	4.1. Is the sampling strategy relevant to address the research question?	X			
	4.2. Is the sample representative of the target population?		X		*Participants were those who responded*

					to the invitation letter and completed the online survey.
	4.3. Are the measurements appropriate?	X			
	4.4. Is the risk of nonresponse bias low?		X		
	4.5. Is the statistical analysis appropriate to answer the research question?	X			
5. Mixed methods	5.1. Is there an adequate rationale for using a mixed methods design to address the research question?	X			
	5.2. Are the different components of the study effectively integrated to answer the research question?	X			
	5.3. Are the outputs of the integration of qualitative and quantitative components adequately interpreted?	X			
	5.4. Are divergences and inconsistencies between quantitative and qualitative results adequately addressed?	X			
	5.5. Do the different components of the study adhere to the quality criteria of each tradition of the methods involved?	X			

Fraser, S.L.; Nadeau, L. Experience and representations of health and social services in a community of Nunavik. Contemp Nurse 2015, 51, 286-300, doi:10.1080/10376178.2016.1171728.

Standards for Reporting Qualitative Research (SRQR)

**No. Topic**	**Item**	**0 – no<br>1 – maybe<br>2-Yes**
**Title and abstract**		
S1 Title	Concise description of the nature and topic of the study identifying the study as qualitative or indicating the approach (e.g., ethnography, grounded theory) or data collection methods (e.g., interview, focus group) is recommended	0
S2 Abstract	Summary of key elements of the study using the abstract format of the intended publication; typically includes objective, methods, results, and conclusions	2
**Introduction**		
S3 Problem formulation	Description and significance of the problem/phenomenon studied; review of relevant theory and empirical work; problem statement	2
S4 Purpose or research question	Purpose of the study and specific objectives or questions	2
**Methods**		
S5 Qualitative approach and research paradigm	Qualitative approach (e.g., ethnography, grounded theory, case study, phenomenology, narrative research) and guiding theory if appropriate; identifying the research paradigm (e.g., positivist, constructivist/interpretivist) is also recommended	2
S6 Researcher characteristics and reflexivity	Researchers' characteristics that may influence the research, including personal attributes, qualifications/experience, relationship with participants, assumptions, or presuppositions; potential or actual interaction between researchers' characteristics and the research questions, approach, methods, results, or transferability	2
S7 Context	Setting/site and salient contextual factors; rationale[a]	2
S8 Sampling strategy	How and why research participants, documents, or events were selected; criteria for deciding when no further sampling was necessary (e.g., sampling saturation); rationale[a]	2
S9 Ethical issues pertaining to human subjects	Documentation of approval by an appropriate ethics review board and participant consent, or explanation for lack thereof; other confidentiality and data security issues	2
S10 Data collection methods	Types of data collected; details of data collection procedures including (as appropriate) start and stop dates of data collection and analysis, iterative process, triangulation of sources/methods,	2

	and modification of procedures in response to evolving study findings; rationale[a]	
S11 Data collection instruments and technologies	Description of instruments (e.g., interview guides, questionnaires) and devices (e.g., audio recorders) used for data collection; if/how the instrument(s) changed over the course of the study	2
S12 Units of study	Number and relevant characteristics of participants, documents, or events included in the study; level of participation (could be reported in results)	2
S13 Data processing	Methods for processing data prior to and during analysis, including transcription, data entry, data management and security, verification of data integrity, data coding, and anonymization/deidentification of excerpts	2
S14 Data analysis	Process by which inferences, themes, etc., were identified and developed, including researchers involved in data analysis; usually references a specific paradigm or approach; rationale[a]	2
S15 Techniques to enhance trustworthiness	Techniques to enhance trustworthiness and credibility of data analysis (e.g., member checking, audit trail, triangulation); rationale[a]	2
**Results/Findings**		
S16 Synthesis and interpretation	Main findings (e.g., interpretations, inferences, and themes); might include development of a theory or model, or integration with prior research or theory	2
S17 Links to empirical data	Evidence (e.g., quotes, field notes, text excerpts, photographs) to substantiate analytic findings	2
**Discussion**		
S18 Integration with prior work, implications, transferability, and contribution(s) to the field	Short summary of main findings; explanation of how findings and conclusions connect to, support, elaborate on, or challenge conclusions of earlier scholarship; discussion of scope of application/generalizability; identification of unique contribution(s) to scholarship in a discipline or field	2
S19 Limitations	Trustworthiness and limitations of findings	0
**Other**		
S20 Conflicts of interest	Potential sources of influence or perceived influence on study conduct and conclusions; how these were managed	0
S21 Funding	Sources of funding and other support; role of funders in data collection, interpretation, and reporting	2

Cooper, R.; Pollock, N.J.; Affleck, Z.; Bain, L.; Hansen, N.L.; Robertson, K.; Chatwood, S. Patient healthcare experiences in the Northwest Territories, Canada: an analysis of news media articles. Int J Circumpolar Health 2021, 80, 1886798, doi:10.1080/22423982.2021.1886798.

Mixed Methods Appraisal Tool (MMAT), version 2018

**Category of study designs**	**Methodological quality criteria**	**Responses**			
		Yes	No	Can't tell	Comments
Screening questions (for all types)	S1. Are there clear research questions?	X			
	S2. Do the collected data allow to address the research questions?	X			
	Further appraisal may not be feasible or appropriate when the answer is 'No' or 'Can't tell' to one or both screening questions.				
1. Qualitative	1.1. Is the qualitative approach appropriate to answer the research question?	X			
	1.2. Are the qualitative data collection methods adequate to address the research question?	X			
	1.3. Are the findings adequately derived from the data?	X			
	1.4. Is the interpretation of results sufficiently substantiated by data?	X			
	1.5. Is there coherence between qualitative data sources, collection, analysis and interpretation?	X			
2. Quantitative randomized controlled trials	2.1. Is randomization appropriately performed?	N/A			
	2.2. Are the groups comparable at baseline?	N/A			
	2.3. Are there complete outcome data?	N/A			
	2.4. Are outcome assessors blinded to the intervention provided?	N/A			
	2.5 Did the participants adhere to the assigned intervention?	N/A			
3. Quantitative non-randomized	3.1. Are the participants representative of the target population?	N/A			
	3.2. Are measurements appropriate regarding both the outcome and intervention (or exposure)?	N/A			
	3.3. Are there complete outcome data?	N/A			
	3.4. Are the confounders accounted for in the design and analysis?	N/A			
	3.5. During the study period, is the intervention administered (or exposure occurred) as intended?	N/A			
4. Quantitative descriptive	4.1. Is the sampling strategy relevant to address the research question?	X			
	4.2. Is the sample representative of the target population?		X		*N/A*
	4.3. Are the measurements appropriate?		X		
	4.4. Is the risk of nonresponse bias low?	X			*Three*

					authors were independently involved in the search.
	4.5. Is the statistical analysis appropriate to answer the research question?	X			
5. Mixed methods	5.1. Is there an adequate rationale for using a mixed methods design to address the research question?	X			
	5.2. Are the different components of the study effectively integrated to answer the research question?	X			
	5.3. Are the outputs of the integration of qualitative and quantitative components adequately interpreted?	X			
	5.4. Are divergences and inconsistencies between quantitative and qualitative results adequately addressed?			X	
	5.5. Do the different components of the study adhere to the quality criteria of each tradition of the methods involved?	X			

McDonald, J.T.; Trenholm, R. Cancer-related health behaviours and health service use among Inuit and other residents of Canada's north. Soc Sci Med 2010, 70, 1396-1403, doi:10.1016/j.socscimed.2010.01.008.

STROBE Statement—checklist of items that should be included in reports of observational studies

	Item No	Recommendation	Recommendation met? (Y/N)
**Title and abstract**	1	(*a*) Indicate the study's design with a commonly used term in the title or the abstract	Y
		(*b*) Provide in the abstract an informative and balanced summary of what was done and what was found	Y
**Introduction**			
Background/rationale	2	Explain the scientific background and rationale for the investigation being reported	Y
Objectives	3	State specific objectives, including any prespecified hypotheses	Y
**Methods**			
Study design	4	Present key elements of study design early in the paper	Y
Setting	5	Describe the setting, locations, and relevant dates, including periods of recruitment, exposure, follow-up, and data collection	Y
Participants	6	(*a*) *Cohort study*—Give the eligibility criteria, and the sources and methods of selection of participants. Describe methods of follow-up<br>*Case-control study*—Give the eligibility criteria, and the sources and methods of case ascertainment and control selection. Give the rationale for the choice of cases and controls<br>*Cross-sectional study*—Give the eligibility criteria, and the sources and methods of selection of participants	N/A
		(*b*) *Cohort study*—For matched studies, give matching criteria and number of exposed and unexposed<br>*Case-control study*—For matched studies, give matching criteria and the number of controls per case	N/A
Variables	7	Clearly define all outcomes, exposures, predictors, potential confounders, and effect modifiers. Give diagnostic criteria, if applicable	Y
Data sources/ measurement	8*	For each variable of interest, give sources of data and details of methods of assessment (measurement). Describe comparability of assessment methods if there is more than one group	Y
Bias	9	Describe any efforts to address potential sources of bias	Y

Study size	10	Explain how the study size was arrived at	N/A
Quantitative variables	11	Explain how quantitative variables were handled in the analyses. If applicable, describe which groupings were chosen and why	Y
Statistical methods	12	(*a*) Describe all statistical methods, including those used to control for confounding	Y
		(*b*) Describe any methods used to examine subgroups and interactions	N/A
		(*c*) Explain how missing data were addressed	N/A
		(*d*) *Cohort study*—If applicable, explain how loss to follow-up was addressed<br>*Case-control study*—If applicable, explain how matching of cases and controls was addressed<br>*Cross-sectional study*—If applicable, describe analytical methods taking account of sampling strategy	N/A
		(*e*) Describe any sensitivity analyses	N/A
**Results**			
Participants	13*	(a) Report numbers of individuals at each stage of study—eg numbers potentially eligible, examined for eligibility, confirmed eligible, included in the study, completing follow-up, and analysed	N/A
		(b) Give reasons for non-participation at each stage	N/A
		(c) Consider use of a flow diagram	N/A
Descriptive data	14*	(a) Give characteristics of study participants (eg demographic, clinical, social) and information on exposures and potential confounders	Y
		(b) Indicate number of participants with missing data for each variable of interest	N/A
		(c) *Cohort study*—Summarise follow-up time (eg, average and total amount)	N/A
Outcome data	15*	*Cohort study*—Report numbers of outcome events or summary measures over time	N/A
		Case-control study—Report numbers in each exposure category, or summary measures of exposure	N/A
		Cross-sectional study—Report numbers of outcome events or summary measures	N/A
Main results	16	(*a*) Give unadjusted estimates and, if applicable, confounder-adjusted estimates and their precision (eg, 95% confidence interval). Make clear which confounders were adjusted for and why they were included	Y
		(*b*) Report category boundaries when continuous variables were categorized	N/A
		(*c*) If relevant, consider translating estimates of relative risk into absolute risk for a meaningful time period	N/A
Other analyses	17	Report other analyses done—eg analyses of subgroups and interactions, and sensitivity analyses	Y

**Discussion**			
Key results	18	Summarise key results with reference to study objectives	Y
Limitations	19	Discuss limitations of the study, taking into account sources of potential bias or imprecision. Discuss both direction and magnitude of any potential bias	Y (in Methods)
Interpretation	20	Give a cautious overall interpretation of results considering objectives, limitations, multiplicity of analyses, results from similar studies, and other relevant evidence	Y
Generalisability	21	Discuss the generalisability (external validity) of the study results	N/A
**Other information**			
Funding	22	Give the source of funding and the role of the funders for the present study and, if applicable, for the original study on which the present article is based	Y

Bhattacharyya, O.K.; Rasooly, I.R.; Naqshbandi, M.; Estey, E.A.; Esler, J.; Toth, E.; Macaulay, A.C.; Harris, S.B. Challenges to the provision of diabetes care in first nations communities: results from a national survey of healthcare providers in Canada. BMC Health Serv Res 2011, 11, 283, doi:10.1186/1472-6963-11-283.

Standards for Reporting Qualitative Research (SRQR)

**No. Topic**	**Item**	**0 – no<br>1 – maybe<br>2-Yes**
**Title and abstract**		
S1 Title	Concise description of the nature and topic of the study identifying the study as qualitative or indicating the approach (e.g., ethnography, grounded theory) or data collection methods (e.g., interview, focus group) is recommended	1
S2 Abstract	Summary of key elements of the study using the abstract format of the intended publication; typically includes objective, methods, results, and conclusions	2
**Introduction**		
S3 Problem formulation	Description and significance of the problem/phenomenon studied; review of relevant theory and empirical work; problem statement	2
S4 Purpose or research question	Purpose of the study and specific objectives or questions	2
**Methods**		
S5 Qualitative approach and research paradigm	Qualitative approach (e.g., ethnography, grounded theory, case study, phenomenology, narrative research) and guiding theory if appropriate; identifying the research paradigm (e.g., positivist, constructivist/interpretivist) is also recommended	2
S6 Researcher characteristics and reflexivity	Researchers' characteristics that may influence the research, including personal attributes, qualifications/experience, relationship with participants, assumptions, or presuppositions; potential or actual interaction between researchers' characteristics and the research questions, approach, methods, results, or transferability	1
S7 Context	Setting/site and salient contextual factors; rationale[a]	2
S8 Sampling strategy	How and why research participants, documents, or events were selected; criteria for deciding when no further sampling was necessary (e.g., sampling saturation); rationale[a]	2
S9 Ethical issues pertaining to human subjects	Documentation of approval by an appropriate ethics review board and participant consent, or explanation for lack thereof; other confidentiality and data security issues	2
S10 Data collection	Types of data collected; details of data collection procedures	2

methods	including (as appropriate) start and stop dates of data collection and analysis, iterative process, triangulation of sources/methods, and modification of procedures in response to evolving study findings; rationale[a]	
S11 Data collection instruments and technologies	Description of instruments (e.g., interview guides, questionnaires) and devices (e.g., audio recorders) used for data collection; if/how the instrument(s) changed over the course of the study	2
S12 Units of study	Number and relevant characteristics of participants, documents, or events included in the study; level of participation (could be reported in results)	2
S13 Data processing	Methods for processing data prior to and during analysis, including transcription, data entry, data management and security, verification of data integrity, data coding, and anonymization/deidentification of excerpts	2
S14 Data analysis	Process by which inferences, themes, etc., were identified and developed, including researchers involved in data analysis; usually references a specific paradigm or approach; rationale[a]	2
S15 Techniques to enhance trustworthiness	Techniques to enhance trustworthiness and credibility of data analysis (e.g., member checking, audit trail, triangulation); rationale[a]	2
**Results/Findings**		
S16 Synthesis and interpretation	Main findings (e.g., interpretations, inferences, and themes); might include development of a theory or model, or integration with prior research or theory	2
S17 Links to empirical data	Evidence (e.g., quotes, field notes, text excerpts, photographs) to substantiate analytic findings	2
**Discussion**		
S18 Integration with prior work, implications, transferability, and contribution(s) to the field	Short summary of main findings; explanation of how findings and conclusions connect to, support, elaborate on, or challenge conclusions of earlier scholarship; discussion of scope of application/generalizability; identification of unique contribution(s) to scholarship in a discipline or field	2
S19 Limitations	Trustworthiness and limitations of findings	2
**Other**		
S20 Conflicts of interest	Potential sources of influence or perceived influence on study conduct and conclusions; how these were managed	2
S21 Funding	Sources of funding and other support; role of funders in data collection, interpretation, and reporting	2

Logie, C.H.; Lys, C. The process of developing a community-based research agenda with lesbian, gay, bisexual, transgender and queer youth in the Northwest Territories, Canada. Int J Circumpolar Health 2015, 74, 28188, doi:10.3402/ijch.v74.28188.

Standards for Reporting Qualitative Research (SRQR)

No. Topic	Item	0 – no 1 – maybe 2-Yes
**Title and abstract**		
S1 Title	Concise description of the nature and topic of the study identifying the study as qualitative or indicating the approach (e.g., ethnography, grounded theory) or data collection methods (e.g., interview, focus group) is recommended	1
S2 Abstract	Summary of key elements of the study using the abstract format of the intended publication; typically includes objective, methods, results, and conclusions	2
**Introduction**		
S3 Problem formulation	Description and significance of the problem/phenomenon studied; review of relevant theory and empirical work; problem statement	2
S4 Purpose or research question	Purpose of the study and specific objectives or questions	2
**Methods**		
S5 Qualitative approach and research paradigm	Qualitative approach (e.g., ethnography, grounded theory, case study, phenomenology, narrative research) and guiding theory if appropriate; identifying the research paradigm (e.g., positivist, constructivist/interpretivist) is also recommended	0
S6 Researcher characteristics and reflexivity	Researchers' characteristics that may influence the research, including personal attributes, qualifications/experience, relationship with participants, assumptions, or presuppositions; potential or actual interaction between researchers' characteristics and the research questions, approach, methods, results, or transferability	0
S7 Context	Setting/site and salient contextual factors; rationale[a]	2
S8 Sampling strategy	How and why research participants, documents, or events were selected; criteria for deciding when no further sampling was necessary (e.g., sampling saturation); rationale[a]	2
S9 Ethical issues pertaining to human subjects	Documentation of approval by an appropriate ethics review board and participant consent, or explanation for lack thereof; other confidentiality and data security issues	0
S10 Data collection methods	Types of data collected; details of data collection procedures including (as appropriate) start and stop dates of data collection	2

	and analysis, iterative process, triangulation of sources/methods, and modification of procedures in response to evolving study findings; rationale[a]	
S11 Data collection instruments and technologies	Description of instruments (e.g., interview guides, questionnaires) and devices (e.g., audio recorders) used for data collection; if/how the instrument(s) changed over the course of the study	0
S12 Units of study	Number and relevant characteristics of participants, documents, or events included in the study; level of participation (could be reported in results)	2
S13 Data processing	Methods for processing data prior to and during analysis, including transcription, data entry, data management and security, verification of data integrity, data coding, and anonymization/deidentification of excerpts	0
S14 Data analysis	Process by which inferences, themes, etc., were identified and developed, including researchers involved in data analysis; usually references a specific paradigm or approach; rationale[a]	0
S15 Techniques to enhance trustworthiness	Techniques to enhance trustworthiness and credibility of data analysis (e.g., member checking, audit trail, triangulation); rationale[a]	0
**Results/Findings**		
S16 Synthesis and interpretation	Main findings (e.g., interpretations, inferences, and themes); might include development of a theory or model, or integration with prior research or theory	2
S17 Links to empirical data	Evidence (e.g., quotes, field notes, text excerpts, photographs) to substantiate analytic findings	0
**Discussion**		
S18 Integration with prior work, implications, transferability, and contribution(s) to the field	Short summary of main findings; explanation of how findings and conclusions connect to, support, elaborate on, or challenge conclusions of earlier scholarship; discussion of scope of application/generalizability; identification of unique contribution(s) to scholarship in a discipline or field	1
S19 Limitations	Trustworthiness and limitations of findings	2
**Other**		
S20 Conflicts of interest	Potential sources of influence or perceived influence on study conduct and conclusions; how these were managed	2
S21 Funding	Sources of funding and other support; role of funders in data collection, interpretation, and reporting	2

Logie, C.H.; Lys, C.L.; Schott, N.; Dias, L.; Zouboules, M.R.; Mackay, K. 'In the North you can't be openly gay': Contextualising sexual practices among sexually and gender diverse persons in Northern Canada. Glob Public Health 2018, 13, 1865-1877, doi:10.1080/17441692.2018.1449881.

Standards for Reporting Qualitative Research (SRQR)

**No. Topic**	**Item**	**0 – no 1 – maybe 2-Yes**
**Title and abstract**		
S1 Title	Concise description of the nature and topic of the study identifying the study as qualitative or indicating the approach (e.g., ethnography, grounded theory) or data collection methods (e.g., interview, focus group) is recommended	2
S2 Abstract	Summary of key elements of the study using the abstract format of the intended publication; typically includes objective, methods, results, and conclusions	2
**Introduction**		
S3 Problem formulation	Description and significance of the problem/phenomenon studied; review of relevant theory and empirical work; problem statement	2
S4 Purpose or research question	Purpose of the study and specific objectives or questions	2
**Methods**		
S5 Qualitative approach and research paradigm	Qualitative approach (e.g., ethnography, grounded theory, case study, phenomenology, narrative research) and guiding theory if appropriate; identifying the research paradigm (e.g., positivist, constructivist/interpretivist) is also recommended	1
S6 Researcher characteristics and reflexivity	Researchers' characteristics that may influence the research, including personal attributes, qualifications/experience, relationship with participants, assumptions, or presuppositions; potential or actual interaction between researchers' characteristics and the research questions, approach, methods, results, or transferability	2
S7 Context	Setting/site and salient contextual factors; rationale[a]	2
S8 Sampling strategy	How and why research participants, documents, or events were selected; criteria for deciding when no further sampling was necessary (e.g., sampling saturation); rationale[a]	2
S9 Ethical issues pertaining to human subjects	Documentation of approval by an appropriate ethics review board and participant consent, or explanation for lack thereof; other confidentiality and data security issues	2
S10 Data collection methods	Types of data collected; details of data collection procedures including (as appropriate) start and stop dates of data collection	2

	and analysis, iterative process, triangulation of sources/methods, and modification of procedures in response to evolving study findings; rationale[a]	
S11 Data collection instruments and technologies	Description of instruments (e.g., interview guides, questionnaires) and devices (e.g., audio recorders) used for data collection; if/how the instrument(s) changed over the course of the study	2
S12 Units of study	Number and relevant characteristics of participants, documents, or events included in the study; level of participation (could be reported in results)	2
S13 Data processing	Methods for processing data prior to and during analysis, including transcription, data entry, data management and security, verification of data integrity, data coding, and anonymization/deidentification of excerpts	2
S14 Data analysis	Process by which inferences, themes, etc., were identified and developed, including researchers involved in data analysis; usually references a specific paradigm or approach; rationale[a]	2
S15 Techniques to enhance trustworthiness	Techniques to enhance trustworthiness and credibility of data analysis (e.g., member checking, audit trail, triangulation); rationale[a]	2
**Results/Findings**		
S16 Synthesis and interpretation	Main findings (e.g., interpretations, inferences, and themes); might include development of a theory or model, or integration with prior research or theory	2
S17 Links to empirical data	Evidence (e.g., quotes, field notes, text excerpts, photographs) to substantiate analytic findings	2
**Discussion**		
S18 Integration with prior work, implications, transferability, and contribution(s) to the field	Short summary of main findings; explanation of how findings and conclusions connect to, support, elaborate on, or challenge conclusions of earlier scholarship; discussion of scope of application/generalizability; identification of unique contribution(s) to scholarship in a discipline or field	2
S19 Limitations	Trustworthiness and limitations of findings	2
**Other**		
S20 Conflicts of interest	Potential sources of influence or perceived influence on study conduct and conclusions; how these were managed	2
S21 Funding	Sources of funding and other support; role of funders in data collection, interpretation, and reporting	2

Liddy, C.; McKellips, F.; Armstrong, C.D.; Afkham, A.; Fraser-Roberts, L.; Keely, E. Improving access to specialists in remote communities: a cross-sectional study and cost analysis of the use of eConsult in Nunavut. Int J Circumpolar Health 2017, 76, 1323493, doi:10.1080/22423982.2017.1323493.

STROBE Statement—checklist of items that should be included in reports of observational studies

	Item No	Recommendation	Recommendation met? (Y/N)
**Title and abstract**	1	(*a*) Indicate the study's design with a commonly used term in the title or the abstract	Y
		(*b*) Provide in the abstract an informative and balanced summary of what was done and what was found	Y
**Introduction**			
Background/rationale	2	Explain the scientific background and rationale for the investigation being reported	Y
Objectives	3	State specific objectives, including any prespecified hypotheses	Y
**Methods**			
Study design	4	Present key elements of study design early in the paper	Y
Setting	5	Describe the setting, locations, and relevant dates, including periods of recruitment, exposure, follow-up, and data collection	Y
Participants	6	(*a*) *Cohort study*—Give the eligibility criteria, and the sources and methods of selection of participants. Describe methods of follow-up *Case-control study*—Give the eligibility criteria, and the sources and methods of case ascertainment and control selection. Give the rationale for the choice of cases and controls *Cross-sectional study*—Give the eligibility criteria, and the sources and methods of selection of participants	Y
		(*b*) *Cohort study*—For matched studies, give matching criteria and number of exposed and unexposed *Case-control study*—For matched studies, give matching criteria and the number of controls per case	N/A
Variables	7	Clearly define all outcomes, exposures, predictors, potential confounders, and effect modifiers. Give diagnostic criteria, if applicable	Y
Data sources/ measurement	8*	For each variable of interest, give sources of data and details of methods of assessment (measurement). Describe comparability of assessment methods if there is more than one group	Y

Bias	9	Describe any efforts to address potential sources of bias	N
Study size	10	Explain how the study size was arrived at	Y
Quantitative variables	11	Explain how quantitative variables were handled in the analyses. If applicable, describe which groupings were chosen and why	Y
Statistical methods	12	(*a*) Describe all statistical methods, including those used to control for confounding	Y
		(*b*) Describe any methods used to examine subgroups and interactions	N/A
		(*c*) Explain how missing data were addressed	N/A
		(*d*) *Cohort study*—If applicable, explain how loss to follow-up was addressed<br>*Case-control study*—If applicable, explain how matching of cases and controls was addressed<br>*Cross-sectional study*—If applicable, describe analytical methods taking account of sampling strategy	N/A
		(*e*) Describe any sensitivity analyses	N/A
**Results**			
Participants	13*	(a) Report numbers of individuals at each stage of study—eg numbers potentially eligible, examined for eligibility, confirmed eligible, included in the study, completing follow-up, and analysed	Y
		(b) Give reasons for non-participation at each stage	N/A
		(c) Consider use of a flow diagram	N/A
Descriptive data	14*	(a) Give characteristics of study participants (eg demographic, clinical, social) and information on exposures and potential confounders	Y
		(b) Indicate number of participants with missing data for each variable of interest	N/A
		(c) *Cohort study*—Summarise follow-up time (eg, average and total amount)	N/A
Outcome data	15*	*Cohort study*—Report numbers of outcome events or summary measures over time	N/A
		Case-control study—Report numbers in each exposure category, or summary measures of exposure	N/A
		Cross-sectional study—Report numbers of outcome events or summary measures	*Y*
Main results	16	(*a*) Give unadjusted estimates and, if applicable, confounder-adjusted estimates and their precision (eg, 95% confidence interval). Make clear which confounders were adjusted for and why they were included	N/A
		(*b*) Report category boundaries when	N/A

		continuous variables were categorized	
		(*c*) If relevant, consider translating estimates of relative risk into absolute risk for a meaningful time period	N/A
Other analyses	17	Report other analyses done—eg analyses of subgroups and interactions, and sensitivity analyses	N/A
**Discussion**			
Key results	18	Summarise key results with reference to study objectives	Y
Limitations	19	Discuss limitations of the study, taking into account sources of potential bias or imprecision. Discuss both direction and magnitude of any potential bias	Y
Interpretation	20	Give a cautious overall interpretation of results considering objectives, limitations, multiplicity of analyses, results from similar studies, and other relevant evidence	Y
Generalisability	21	Discuss the generalisability (external validity) of the study results	N/A
**Other information**			
Funding	22	Give the source of funding and the role of the funders for the present study and, if applicable, for the original study on which the present article is based	Y

Kerber, K.; Kolahdooz, F.; Otway, M.; Laboucan, M.; Jang, S.L.; Lawrence, S.; Aronyk, S.; Quinn, M.; Irlbacher-Fox, S.; Milligan, C.; et al. Opportunities for improving patient experiences among medical travellers from Canada's far north: a mixed-methods study. BMJ Open 2019, 9, e030885, doi:10.1136/bmjopen-2019-030885.

Standards for Reporting Qualitative Research (SRQR)

**No. Topic**	**Item**	**0 – no 1 – maybe 2-Yes**
**Title and abstract**		
S1 Title	Concise description of the nature and topic of the study identifying the study as qualitative or indicating the approach (e.g., ethnography, grounded theory) or data collection methods (e.g., interview, focus group) is recommended	2
S2 Abstract	Summary of key elements of the study using the abstract format of the intended publication; typically includes objective, methods, results, and conclusions	2
**Introduction**		
S3 Problem formulation	Description and significance of the problem/phenomenon studied; review of relevant theory and empirical work; problem statement	2
S4 Purpose or research question	Purpose of the study and specific objectives or questions	2
**Methods**		
S5 Qualitative approach and research paradigm	Qualitative approach (e.g., ethnography, grounded theory, case study, phenomenology, narrative research) and guiding theory if appropriate; identifying the research paradigm (e.g., positivist, constructivist/interpretivist) is also recommended	1
S6 Researcher characteristics and reflexivity	Researchers' characteristics that may influence the research, including personal attributes, qualifications/experience, relationship with participants, assumptions, or presuppositions; potential or actual interaction between researchers' characteristics and the research questions, approach, methods, results, or transferability	2
S7 Context	Setting/site and salient contextual factors; rationale[a]	2
S8 Sampling strategy	How and why research participants, documents, or events were selected; criteria for deciding when no further sampling was necessary (e.g., sampling saturation); rationale[a]	2
S9 Ethical issues pertaining to human subjects	Documentation of approval by an appropriate ethics review board and participant consent, or explanation for lack thereof; other confidentiality and data security issues	2
S10 Data collection	Types of data collected; details of data collection procedures	2

methods	including (as appropriate) start and stop dates of data collection and analysis, iterative process, triangulation of sources/methods, and modification of procedures in response to evolving study findings; rationale[a]	
S11 Data collection instruments and technologies	Description of instruments (e.g., interview guides, questionnaires) and devices (e.g., audio recorders) used for data collection; if/how the instrument(s) changed over the course of the study	2
S12 Units of study	Number and relevant characteristics of participants, documents, or events included in the study; level of participation (could be reported in results)	2
S13 Data processing	Methods for processing data prior to and during analysis, including transcription, data entry, data management and security, verification of data integrity, data coding, and anonymization/deidentification of excerpts	2
S14 Data analysis	Process by which inferences, themes, etc., were identified and developed, including researchers involved in data analysis; usually references a specific paradigm or approach; rationale[a]	1
S15 Techniques to enhance trustworthiness	Techniques to enhance trustworthiness and credibility of data analysis (e.g., member checking, audit trail, triangulation); rationale[a]	1
**Results/Findings**		
S16 Synthesis and interpretation	Main findings (e.g., interpretations, inferences, and themes); might include development of a theory or model, or integration with prior research or theory	2
S17 Links to empirical data	Evidence (e.g., quotes, field notes, text excerpts, photographs) to substantiate analytic findings	2
**Discussion**		
S18 Integration with prior work, implications, transferability, and contribution(s) to the field	Short summary of main findings; explanation of how findings and conclusions connect to, support, elaborate on, or challenge conclusions of earlier scholarship; discussion of scope of application/generalizability; identification of unique contribution(s) to scholarship in a discipline or field	2
S19 Limitations	Trustworthiness and limitations of findings	2
**Other**		
S20 Conflicts of interest	Potential sources of influence or perceived influence on study conduct and conclusions; how these were managed	2
S21 Funding	Sources of funding and other support; role of funders in data collection, interpretation, and reporting	2

Young, T.K.; Tabish, T.; Young, S.K.; Healey, G. Patient transportation in Canada's northern territories: patterns, costs and providers' perspectives. Rural Remote Health 2019, 19, 5113, doi:10.22605/RRH5113.

STROBE Statement—checklist of items that should be included in reports of observational studies

	Item No	Recommendation	Recommendation met? (Y/N)
**Title and abstract**	1	(*a*) Indicate the study's design with a commonly used term in the title or the abstract	Y
		(*b*) Provide in the abstract an informative and balanced summary of what was done and what was found	Y
**Introduction**			
Background/rationale	2	Explain the scientific background and rationale for the investigation being reported	Y
Objectives	3	State specific objectives, including any prespecified hypotheses	Y
**Methods**			
Study design	4	Present key elements of study design early in the paper	Y
Setting	5	Describe the setting, locations, and relevant dates, including periods of recruitment, exposure, follow-up, and data collection	Y
Participants	6	(*a*) *Cohort study*—Give the eligibility criteria, and the sources and methods of selection of participants. Describe methods of follow-up<br>*Case-control study*—Give the eligibility criteria, and the sources and methods of case ascertainment and control selection. Give the rationale for the choice of cases and controls<br>*Cross-sectional study*—Give the eligibility criteria, and the sources and methods of selection of participants	Y
		(*b*) *Cohort study*—For matched studies, give matching criteria and number of exposed and unexposed<br>*Case-control study*—For matched studies, give matching criteria and the number of controls per case	N/A
Variables	7	Clearly define all outcomes, exposures, predictors, potential confounders, and effect modifiers. Give diagnostic criteria, if applicable	Y
Data sources/ measurement	8*	For each variable of interest, give sources of data and details of methods of assessment (measurement). Describe comparability of assessment methods if there is more than one group	Y
Bias	9	Describe any efforts to address potential	N

		sources of bias	
Study size	10	Explain how the study size was arrived at	N
Quantitative variables	11	Explain how quantitative variables were handled in the analyses. If applicable, describe which groupings were chosen and why	Y
Statistical methods	12	(*a*) Describe all statistical methods, including those used to control for confounding	N
		(*b*) Describe any methods used to examine subgroups and interactions	N/A
		(*c*) Explain how missing data were addressed	N
		(*d*) *Cohort study*—If applicable, explain how loss to follow-up was addressed<br>*Case-control study*—If applicable, explain how matching of cases and controls was addressed<br>*Cross-sectional study*—If applicable, describe analytical methods taking account of sampling strategy	N/A
		(*e*) Describe any sensitivity analyses	N/A
**Results**			
Participants	13*	(a) Report numbers of individuals at each stage of study—eg numbers potentially eligible, examined for eligibility, confirmed eligible, included in the study, completing follow-up, and analysed	N/A
		(b) Give reasons for non-participation at each stage	N
		(c) Consider use of a flow diagram	N
Descriptive data	14*	(a) Give characteristics of study participants (eg demographic, clinical, social) and information on exposures and potential confounders	N
		(b) Indicate number of participants with missing data for each variable of interest	N
		(c) *Cohort study*—Summarise follow-up time (eg, average and total amount)	N/A
Outcome data	15*	*Cohort study*—Report numbers of outcome events or summary measures over time	N/A
		Case-control study—Report numbers in each exposure category, or summary measures of exposure	N/A
		Cross-sectional study—Report numbers of outcome events or summary measures	*Y*
Main results	16	(*a*) Give unadjusted estimates and, if applicable, confounder-adjusted estimates and their precision (eg, 95% confidence interval). Make clear which confounders were adjusted for and why they were included	N/A
		(*b*) Report category boundaries when continuous variables were categorized	N/A

		(*c*) If relevant, consider translating estimates of relative risk into absolute risk for a meaningful time period	N/A
Other analyses	17	Report other analyses done—eg analyses of subgroups and interactions, and sensitivity analyses	N/A
**Discussion**			
Key results	18	Summarise key results with reference to study objectives	Y
Limitations	19	Discuss limitations of the study, taking into account sources of potential bias or imprecision. Discuss both direction and magnitude of any potential bias	Y
Interpretation	20	Give a cautious overall interpretation of results considering objectives, limitations, multiplicity of analyses, results from similar studies, and other relevant evidence	Y
Generalisability	21	Discuss the generalisability (external validity) of the study results	N/A
**Other information**			
Funding	22	Give the source of funding and the role of the funders for the present study and, if applicable, for the original study on which the present article is based	N

Chamberlain, M.; Barclay, K. Psychosocial costs of transferring indigenous women from their community for birth. Midwifery 2000, 16, 116-122, doi:10.1054/midw.1999.0202.

Standards for Reporting Qualitative Research (SRQR)

**No. Topic**	**Item**	**0 – no 1 – maybe 2-Yes**
**Title and abstract**		
S1 Title	Concise description of the nature and topic of the study identifying the study as qualitative or indicating the approach (e.g., ethnography, grounded theory) or data collection methods (e.g., interview, focus group) is recommended	2
S2 Abstract	Summary of key elements of the study using the abstract format of the intended publication; typically includes objective, methods, results, and conclusions	2
**Introduction**		
S3 Problem formulation	Description and significance of the problem/phenomenon studied; review of relevant theory and empirical work; problem statement	2
S4 Purpose or research question	Purpose of the study and specific objectives or questions	2
**Methods**		
S5 Qualitative approach and research paradigm	Qualitative approach (e.g., ethnography, grounded theory, case study, phenomenology, narrative research) and guiding theory if appropriate; identifying the research paradigm (e.g., positivist, constructivist/interpretivist) is also recommended	1
S6 Researcher characteristics and reflexivity	Researchers' characteristics that may influence the research, including personal attributes, qualifications/experience, relationship with participants, assumptions, or presuppositions; potential or actual interaction between researchers' characteristics and the research questions, approach, methods, results, or transferability	1
S7 Context	Setting/site and salient contextual factors; rationale[a]	2
S8 Sampling strategy	How and why research participants, documents, or events were selected; criteria for deciding when no further sampling was necessary (e.g., sampling saturation); rationale[a]	2
S9 Ethical issues pertaining to human subjects	Documentation of approval by an appropriate ethics review board and participant consent, or explanation for lack thereof; other confidentiality and data security issues	2
S10 Data collection methods	Types of data collected; details of data collection procedures including (as appropriate) start and stop dates of data collection and analysis, iterative process, triangulation of sources/methods,	2

	and modification of procedures in response to evolving study findings; rationale[a]	
S11 Data collection instruments and technologies	Description of instruments (e.g., interview guides, questionnaires) and devices (e.g., audio recorders) used for data collection; if/how the instrument(s) changed over the course of the study	2
S12 Units of study	Number and relevant characteristics of participants, documents, or events included in the study; level of participation (could be reported in results)	2
S13 Data processing	Methods for processing data prior to and during analysis, including transcription, data entry, data management and security, verification of data integrity, data coding, and anonymization/deidentification of excerpts	2
S14 Data analysis	Process by which inferences, themes, etc., were identified and developed, including researchers involved in data analysis; usually references a specific paradigm or approach; rationale[a]	2
S15 Techniques to enhance trustworthiness	Techniques to enhance trustworthiness and credibility of data analysis (e.g., member checking, audit trail, triangulation); rationale[a]	2
**Results/Findings**		
S16 Synthesis and interpretation	Main findings (e.g., interpretations, inferences, and themes); might include development of a theory or model, or integration with prior research or theory	2
S17 Links to empirical data	Evidence (e.g., quotes, field notes, text excerpts, photographs) to substantiate analytic findings	2
**Discussion**		
S18 Integration with prior work, implications, transferability, and contribution(s) to the field	Short summary of main findings; explanation of how findings and conclusions connect to, support, elaborate on, or challenge conclusions of earlier scholarship; discussion of scope of application/generalizability; identification of unique contribution(s) to scholarship in a discipline or field	2
S19 Limitations	Trustworthiness and limitations of findings	2
**Other**		
S20 Conflicts of interest	Potential sources of influence or perceived influence on study conduct and conclusions; how these were managed	1
S21 Funding	Sources of funding and other support; role of funders in data collection, interpretation, and reporting	2

Sheffield, H.A.; Sheffield, C.A. Nasal CPAP on paediatric air transport in the Canadian Arctic: A case series. Paediatr Child Health 2019, 24, e94-e97, doi:10.1093/pch/pxy088.

STROBE Statement—checklist of items that should be included in reports of observational studies

	Item No	Recommendation	Recommendation met? (Y/N)
**Title and abstract**	1	(*a*) Indicate the study's design with a commonly used term in the title or the abstract	Y
		(*b*) Provide in the abstract an informative and balanced summary of what was done and what was found	Y
**Introduction**			
Background/rationale	2	Explain the scientific background and rationale for the investigation being reported	Y
Objectives	3	State specific objectives, including any prespecified hypotheses	Y
**Methods**			
Study design	4	Present key elements of study design early in the paper	Y
Setting	5	Describe the setting, locations, and relevant dates, including periods of recruitment, exposure, follow-up, and data collection	Y
Participants	6	(*a*) *Cohort study*—Give the eligibility criteria, and the sources and methods of selection of participants. Describe methods of follow-up *Case-control study*—Give the eligibility criteria, and the sources and methods of case ascertainment and control selection. Give the rationale for the choice of cases and controls *Cross-sectional study*—Give the eligibility criteria, and the sources and methods of selection of participants	Y
		(*b*) *Cohort study*—For matched studies, give matching criteria and number of exposed and unexposed *Case-control study*—For matched studies, give matching criteria and the number of controls per case	N/A
Variables	7	Clearly define all outcomes, exposures, predictors, potential confounders, and effect modifiers. Give diagnostic criteria, if applicable	N/A
Data sources/ measurement	8*	For each variable of interest, give sources of data and details of methods of assessment (measurement). Describe comparability of assessment methods if there is more than one group	N/A
Bias	9	Describe any efforts to address potential	N

		sources of bias	
Study size	10	Explain how the study size was arrived at	N/A
Quantitative variables	11	Explain how quantitative variables were handled in the analyses. If applicable, describe which groupings were chosen and why	N/A
Statistical methods	12	(*a*) Describe all statistical methods, including those used to control for confounding	N/A
		(*b*) Describe any methods used to examine subgroups and interactions	N/A
		(*c*) Explain how missing data were addressed	N/A
		(*d*) *Cohort study*—If applicable, explain how loss to follow-up was addressed<br>*Case-control study*—If applicable, explain how matching of cases and controls was addressed<br>*Cross-sectional study*—If applicable, describe analytical methods taking account of sampling strategy	N/A
		(*e*) Describe any sensitivity analyses	N/A
**Results**			
Participants	13*	(a) Report numbers of individuals at each stage of study—eg numbers potentially eligible, examined for eligibility, confirmed eligible, included in the study, completing follow-up, and analysed	N/A
		(b) Give reasons for non-participation at each stage	N/A
		(c) Consider use of a flow diagram	N/A
Descriptive data	14*	(a) Give characteristics of study participants (eg demographic, clinical, social) and information on exposures and potential confounders	Y
		(b) Indicate number of participants with missing data for each variable of interest	N/A
		(c) *Cohort study*—Summarise follow-up time (eg, average and total amount)	N/A
Outcome data	15*	*Cohort study*—Report numbers of outcome events or summary measures over time	N/A
		Case-control study—Report numbers in each exposure category, or summary measures of exposure	*Y*
		Cross-sectional study—Report numbers of outcome events or summary measures	N/A
Main results	16	(*a*) Give unadjusted estimates and, if applicable, confounder-adjusted estimates and their precision (eg, 95% confidence interval). Make clear which confounders were adjusted for and why they were included	N/A
		(*b*) Report category boundaries when continuous variables were categorized	N/A

		(*c*) If relevant, consider translating estimates of relative risk into absolute risk for a meaningful time period	N/A
Other analyses	17	Report other analyses done—eg analyses of subgroups and interactions, and sensitivity analyses	N/A
**Discussion**			
Key results	18	Summarise key results with reference to study objectives	
Limitations	19	Discuss limitations of the study, taking into account sources of potential bias or imprecision. Discuss both direction and magnitude of any potential bias	Y
Interpretation	20	Give a cautious overall interpretation of results considering objectives, limitations, multiplicity of analyses, results from similar studies, and other relevant evidence	Y
Generalisability	21	Discuss the generalisability (external validity) of the study results	N/A
**Other information**			
Funding	22	Give the source of funding and the role of the funders for the present study and, if applicable, for the original study on which the present article is based	Y

Jull, J.; Sheppard, A.J.; Hizaka, A.; Inuit Medical Interpreter, T.; Barton, G.; Doering, P.; Dorschner, D.; Edgecombe, N.; Ellis, M.; Graham, I.D.; et al. Experiences of Inuit in Canada who travel from remote settings for cancer care and impacts on decision making. BMC Health Serv Res 2021, 21, 328, doi:10.1186/s12913-021-06303-9.

Standards for Reporting Qualitative Research (SRQR)

**No. Topic**	**Item**	**0 – no 1 – maybe 2-Yes**
**Title and abstract**		
S1 Title	Concise description of the nature and topic of the study identifying the study as qualitative or indicating the approach (e.g., ethnography, grounded theory) or data collection methods (e.g., interview, focus group) is recommended	1
S2 Abstract	Summary of key elements of the study using the abstract format of the intended publication; typically includes objective, methods, results, and conclusions	2
**Introduction**		
S3 Problem formulation	Description and significance of the problem/phenomenon studied; review of relevant theory and empirical work; problem statement	2
S4 Purpose or research question	Purpose of the study and specific objectives or questions	2
**Methods**		
S5 Qualitative approach and research paradigm	Qualitative approach (e.g., ethnography, grounded theory, case study, phenomenology, narrative research) and guiding theory if appropriate; identifying the research paradigm (e.g., positivist, constructivist/interpretivist) is also recommended	2
S6 Researcher characteristics and reflexivity	Researchers' characteristics that may influence the research, including personal attributes, qualifications/experience, relationship with participants, assumptions, or presuppositions; potential or actual interaction between researchers' characteristics and the research questions, approach, methods, results, or transferability	2
S7 Context	Setting/site and salient contextual factors; rationale[a]	2
S8 Sampling strategy	How and why research participants, documents, or events were selected; criteria for deciding when no further sampling was necessary (e.g., sampling saturation); rationale[a]	1
S9 Ethical issues pertaining to human subjects	Documentation of approval by an appropriate ethics review board and participant consent, or explanation for lack thereof; other confidentiality and data security issues	2
S10 Data collection	Types of data collected; details of data collection procedures	2

methods	including (as appropriate) start and stop dates of data collection and analysis, iterative process, triangulation of sources/methods, and modification of procedures in response to evolving study findings; rationale[a]	
S11 Data collection instruments and technologies	Description of instruments (e.g., interview guides, questionnaires) and devices (e.g., audio recorders) used for data collection; if/how the instrument(s) changed over the course of the study	2
S12 Units of study	Number and relevant characteristics of participants, documents, or events included in the study; level of participation (could be reported in results)	2
S13 Data processing	Methods for processing data prior to and during analysis, including transcription, data entry, data management and security, verification of data integrity, data coding, and anonymization/deidentification of excerpts	2
S14 Data analysis	Process by which inferences, themes, etc., were identified and developed, including researchers involved in data analysis; usually references a specific paradigm or approach; rationale[a]	2
S15 Techniques to enhance trustworthiness	Techniques to enhance trustworthiness and credibility of data analysis (e.g., member checking, audit trail, triangulation); rationale[a]	2
**Results/Findings**		
S16 Synthesis and interpretation	Main findings (e.g., interpretations, inferences, and themes); might include development of a theory or model, or integration with prior research or theory	2
S17 Links to empirical data	Evidence (e.g., quotes, field notes, text excerpts, photographs) to substantiate analytic findings	2
**Discussion**		
S18 Integration with prior work, implications, transferability, and contribution(s) to the field	Short summary of main findings; explanation of how findings and conclusions connect to, support, elaborate on, or challenge conclusions of earlier scholarship; discussion of scope of application/generalizability; identification of unique contribution(s) to scholarship in a discipline or field	2
S19 Limitations	Trustworthiness and limitations of findings	2
**Other**		
S20 Conflicts of interest	Potential sources of influence or perceived influence on study conduct and conclusions; how these were managed	2
S21 Funding	Sources of funding and other support; role of funders in data collection, interpretation, and reporting	2

Mendez, I.; Jong, M.; Keays-White, D.; Turner, G. The use of remote presence for health care delivery in a northern Inuit community: a feasibility study. International Journal of Circumpolar Health 2013, 72, 21112, doi:10.3402/ijch.v72i0.21112.

STROBE Statement—checklist of items that should be included in reports of observational studies

	Item No	Recommendation	Recommendation met? (Y/N)
**Title and abstract**	1	(*a*) Indicate the study's design with a commonly used term in the title or the abstract	Y
		(*b*) Provide in the abstract an informative and balanced summary of what was done and what was found	Y
**Introduction**			
Background/rationale	2	Explain the scientific background and rationale for the investigation being reported	Y
Objectives	3	State specific objectives, including any prespecified hypotheses	Y
**Methods**			
Study design	4	Present key elements of study design early in the paper	Y
Setting	5	Describe the setting, locations, and relevant dates, including periods of recruitment, exposure, follow-up, and data collection	Y
Participants	6	(*a*) *Cohort study*—Give the eligibility criteria, and the sources and methods of selection of participants. Describe methods of follow-up *Case-control study*—Give the eligibility criteria, and the sources and methods of case ascertainment and control selection. Give the rationale for the choice of cases and controls *Cross-sectional study*—Give the eligibility criteria, and the sources and methods of selection of participants	N/A
		(*b*) *Cohort study*—For matched studies, give matching criteria and number of exposed and unexposed *Case-control study*—For matched studies, give matching criteria and the number of controls per case	N/A
Variables	7	Clearly define all outcomes, exposures, predictors, potential confounders, and effect modifiers. Give diagnostic criteria, if applicable	N/A
Data sources/ measurement	8*	For each variable of interest, give sources of data and details of methods of assessment (measurement). Describe comparability of assessment methods if there is more than one group	Y

Bias	9	Describe any efforts to address potential sources of bias	N/A
Study size	10	Explain how the study size was arrived at	N/A
Quantitative variables	11	Explain how quantitative variables were handled in the analyses. If applicable, describe which groupings were chosen and why	N/A
Statistical methods	12	(*a*) Describe all statistical methods, including those used to control for confounding	N/A
		(*b*) Describe any methods used to examine subgroups and interactions	N/A
		(*c*) Explain how missing data were addressed	N
		(*d*) *Cohort study*—If applicable, explain how loss to follow-up was addressed<br>*Case-control study*—If applicable, explain how matching of cases and controls was addressed<br>*Cross-sectional study*—If applicable, describe analytical methods taking account of sampling strategy	N/A
		(*e*) Describe any sensitivity analyses	N/A
**Results**			
Participants	13*	(a) Report numbers of individuals at each stage of study—eg numbers potentially eligible, examined for eligibility, confirmed eligible, included in the study, completing follow-up, and analysed	Y
		(b) Give reasons for non-participation at each stage	N
		(c) Consider use of a flow diagram	N/A
Descriptive data	14*	(a) Give characteristics of study participants (eg demographic, clinical, social) and information on exposures and potential confounders	N
		(b) Indicate number of participants with missing data for each variable of interest	N/A
		(c) *Cohort study*—Summarise follow-up time (eg, average and total amount)	N/A
Outcome data	15*	*Cohort study*—Report numbers of outcome events or summary measures over time	N/A
		Case-control study—Report numbers in each exposure category, or summary measures of exposure	N/A
		Cross-sectional study—Report numbers of outcome events or summary measures	*Y*
Main results	16	(*a*) Give unadjusted estimates and, if applicable, confounder-adjusted estimates and their precision (eg, 95% confidence interval). Make clear which confounders were adjusted for and why they were included	N/A
		(*b*) Report category boundaries when	N/A

		continuous variables were categorized	
		(*c*) If relevant, consider translating estimates of relative risk into absolute risk for a meaningful time period	N/A
Other analyses	17	Report other analyses done—eg analyses of subgroups and interactions, and sensitivity analyses	N/A
**Discussion**			
Key results	18	Summarise key results with reference to study objectives	Y
Limitations	19	Discuss limitations of the study, taking into account sources of potential bias or imprecision. Discuss both direction and magnitude of any potential bias	N
Interpretation	20	Give a cautious overall interpretation of results considering objectives, limitations, multiplicity of analyses, results from similar studies, and other relevant evidence	Y
Generalisability	21	Discuss the generalisability (external validity) of the study results	N/A
**Other information**			
Funding	22	Give the source of funding and the role of the funders for the present study and, if applicable, for the original study on which the present article is based	Y

Douglas, V.; Chan, H.M.; Wesche, S.; Dickson, C.; Kassi, N.; Netro, L.; Williams, M. Reconciling traditional knowledge, food security, and climate change: experience from Old Crow, YT, Canada. Prog Community Health Partnersh 2014, 8, 21-27, doi:10.1353/cpr.2014.0007.

Standards for Reporting Qualitative Research (SRQR)

No. Topic	Item	0 – no 1 – maybe 2-Yes
**Title and abstract**		
S1 Title	Concise description of the nature and topic of the study identifying the study as qualitative or indicating the approach (e.g., ethnography, grounded theory) or data collection methods (e.g., interview, focus group) is recommended	2
S2 Abstract	Summary of key elements of the study using the abstract format of the intended publication; typically includes objective, methods, results, and conclusions	2
**Introduction**		
S3 Problem formulation	Description and significance of the problem/phenomenon studied; review of relevant theory and empirical work; problem statement	2
S4 Purpose or research question	Purpose of the study and specific objectives or questions	2
**Methods**		
S5 Qualitative approach and research paradigm	Qualitative approach (e.g., ethnography, grounded theory, case study, phenomenology, narrative research) and guiding theory if appropriate; identifying the research paradigm (e.g., positivist, constructivist/interpretivist) is also recommended	2
S6 Researcher characteristics and reflexivity	Researchers' characteristics that may influence the research, including personal attributes, qualifications/experience, relationship with participants, assumptions, or presuppositions; potential or actual interaction between researchers' characteristics and the research questions, approach, methods, results, or transferability	2
S7 Context	Setting/site and salient contextual factors; rationale[a]	2
S8 Sampling strategy	How and why research participants, documents, or events were selected; criteria for deciding when no further sampling was necessary (e.g., sampling saturation); rationale[a]	2
S9 Ethical issues pertaining to human subjects	Documentation of approval by an appropriate ethics review board and participant consent, or explanation for lack thereof; other confidentiality and data security issues	2
S10 Data collection methods	Types of data collected; details of data collection procedures including (as appropriate) start and stop dates of data collection	2

	and analysis, iterative process, triangulation of sources/methods, and modification of procedures in response to evolving study findings; rationale[a]	
S11 Data collection instruments and technologies	Description of instruments (e.g., interview guides, questionnaires) and devices (e.g., audio recorders) used for data collection; if/how the instrument(s) changed over the course of the study	2
S12 Units of study	Number and relevant characteristics of participants, documents, or events included in the study; level of participation (could be reported in results)	2
S13 Data processing	Methods for processing data prior to and during analysis, including transcription, data entry, data management and security, verification of data integrity, data coding, and anonymization/deidentification of excerpts	2
S14 Data analysis	Process by which inferences, themes, etc., were identified and developed, including researchers involved in data analysis; usually references a specific paradigm or approach; rationale[a]	2
S15 Techniques to enhance trustworthiness	Techniques to enhance trustworthiness and credibility of data analysis (e.g., member checking, audit trail, triangulation); rationale[a]	2
**Results/Findings**		
S16 Synthesis and interpretation	Main findings (e.g., interpretations, inferences, and themes); might include development of a theory or model, or integration with prior research or theory	2
S17 Links to empirical data	Evidence (e.g., quotes, field notes, text excerpts, photographs) to substantiate analytic findings	2
**Discussion**		
S18 Integration with prior work, implications, transferability, and contribution(s) to the field	Short summary of main findings; explanation of how findings and conclusions connect to, support, elaborate on, or challenge conclusions of earlier scholarship; discussion of scope of application/generalizability; identification of unique contribution(s) to scholarship in a discipline or field	2
S19 Limitations	Trustworthiness and limitations of findings	0
**Other**		
S20 Conflicts of interest	Potential sources of influence or perceived influence on study conduct and conclusions; how these were managed	0
S21 Funding	Sources of funding and other support; role of funders in data collection, interpretation, and reporting	0

Guo, Y.; Berrang-Ford, L.; Ford, J.; Lardeau, M.P.; Edge, V.; Patterson, K.; Team, I.R.; Harper, S.L. Seasonal prevalence and determinants of food insecurity in Iqaluit, Nunavut. Int J Circumpolar Health 2015, 74, 27284, doi:10.3402/ijch.v74.27284.

STROBE Statement—checklist of items that should be included in reports of observational studies

	Item No	Recommendation	Recommendation met? (Y/N)
**Title and abstract**	1	(*a*) Indicate the study's design with a commonly used term in the title or the abstract	Y
		(*b*) Provide in the abstract an informative and balanced summary of what was done and what was found	Y
**Introduction**			
Background/rationale	2	Explain the scientific background and rationale for the investigation being reported	Y
Objectives	3	State specific objectives, including any prespecified hypotheses	Y
**Methods**			
Study design	4	Present key elements of study design early in the paper	Y
Setting	5	Describe the setting, locations, and relevant dates, including periods of recruitment, exposure, follow-up, and data collection	Y
Participants	6	(*a*) *Cohort study*—Give the eligibility criteria, and the sources and methods of selection of participants. Describe methods of follow-up<br>*Case-control study*—Give the eligibility criteria, and the sources and methods of case ascertainment and control selection. Give the rationale for the choice of cases and controls<br>*Cross-sectional study*—Give the eligibility criteria, and the sources and methods of selection of participants	Y
		(*b*) *Cohort study*—For matched studies, give matching criteria and number of exposed and unexposed<br>*Case-control study*—For matched studies, give matching criteria and the number of controls per case	N/A
Variables	7	Clearly define all outcomes, exposures, predictors, potential confounders, and effect modifiers. Give diagnostic criteria, if applicable	Y
Data sources/ measurement	8*	For each variable of interest, give sources of data and details of methods of assessment (measurement). Describe comparability of assessment methods if there is more than one group	Y
Bias	9	Describe any efforts to address potential sources of bias	Y

Study size	10	Explain how the study size was arrived at	Y
Quantitative variables	11	Explain how quantitative variables were handled in the analyses. If applicable, describe which groupings were chosen and why	Y
Statistical methods	12	(*a*) Describe all statistical methods, including those used to control for confounding	Y
		(*b*) Describe any methods used to examine subgroups and interactions	Y
		(*c*) Explain how missing data were addressed	Y
		(*d*) *Cohort study*—If applicable, explain how loss to follow-up was addressed<br>*Case-control study*—If applicable, explain how matching of cases and controls was addressed<br>*Cross-sectional study*—If applicable, describe analytical methods taking account of sampling strategy	N/A
		(*e*) Describe any sensitivity analyses	N
**Results**			
Participants	13*	(a) Report numbers of individuals at each stage of study—eg numbers potentially eligible, examined for eligibility, confirmed eligible, included in the study, completing follow-up, and analysed	Y
		(b) Give reasons for non-participation at each stage	N
		(c) Consider use of a flow diagram	N
Descriptive data	14*	(a) Give characteristics of study participants (eg demographic, clinical, social) and information on exposures and potential confounders	Y
		(b) Indicate number of participants with missing data for each variable of interest	Y
		(c) *Cohort study*—Summarise follow-up time (eg, average and total amount)	N/A
Outcome data	15*	*Cohort study*—Report numbers of outcome events or summary measures over time	N/A
		Case-control study—Report numbers in each exposure category, or summary measures of exposure	N/A
		Cross-sectional study—Report numbers of outcome events or summary measures	Y
Main results	16	(*a*) Give unadjusted estimates and, if applicable, confounder-adjusted estimates and their precision (eg, 95% confidence interval). Make clear which confounders were adjusted for and why they were included	Y
		(*b*) Report category boundaries when continuous variables were categorized	N/A
		(*c*) If relevant, consider translating estimates of relative risk into absolute risk for a meaningful time period	N/A
Other analyses	17	Report other analyses done—eg analyses of subgroups and interactions, and sensitivity analyses	Y

**Discussion**			
Key results	18	Summarise key results with reference to study objectives	Y
Limitations	19	Discuss limitations of the study, taking into account sources of potential bias or imprecision. Discuss both direction and magnitude of any potential bias	Y
Interpretation	20	Give a cautious overall interpretation of results considering objectives, limitations, multiplicity of analyses, results from similar studies, and other relevant evidence	Y
Generalisability	21	Discuss the generalisability (external validity) of the study results	N
**Other information**			
Funding	22	Give the source of funding and the role of the funders for the present study and, if applicable, for the original study on which the present article is based	Y

Huet, C.; Ford, J.D.; Edge, V.L.; Shirley, J.; King, N.; Team, I.R.; Harper, S.L. Food insecurity and food consumption by season in households with children in an Arctic city: a cross-sectional study. BMC Public Health 2017, 17, 578, doi:10.1186/s12889-017-4393-6.

STROBE Statement—checklist of items that should be included in reports of observational studies

	**Item No**	**Recommendation**	**Recommendation met? (Y/N)**
**Title and abstract**	1	(*a*) Indicate the study's design with a commonly used term in the title or the abstract	Y
		(*b*) Provide in the abstract an informative and balanced summary of what was done and what was found	Y
**Introduction**			
Background/rationale	2	Explain the scientific background and rationale for the investigation being reported	Y
Objectives	3	State specific objectives, including any prespecified hypotheses	Y
**Methods**			
Study design	4	Present key elements of study design early in the paper	Y
Setting	5	Describe the setting, locations, and relevant dates, including periods of recruitment, exposure, follow-up, and data collection	Y
Participants	6	(*a*) *Cohort study*—Give the eligibility criteria, and the sources and methods of selection of participants. Describe methods of follow-up<br>*Case-control study*—Give the eligibility criteria, and the sources and methods of case ascertainment and control selection. Give the rationale for the choice of cases and controls<br>*Cross-sectional study*—Give the eligibility criteria, and the sources and methods of selection of participants	Y
		(*b*) *Cohort study*—For matched studies, give matching criteria and number of exposed and unexposed<br>*Case-control study*—For matched studies, give matching criteria and the number of controls per case	N/A
Variables	7	Clearly define all outcomes, exposures, predictors, potential confounders, and effect modifiers. Give diagnostic criteria, if applicable	Y
Data sources/ measurement	8*	For each variable of interest, give sources of data and details of methods of assessment (measurement). Describe comparability of assessment methods if there is more than one group	Y
Bias	9	Describe any efforts to address potential sources of bias	Y

Study size	10	Explain how the study size was arrived at	Y
Quantitative variables	11	Explain how quantitative variables were handled in the analyses. If applicable, describe which groupings were chosen and why	Y
Statistical methods	12	(*a*) Describe all statistical methods, including those used to control for confounding	Y
		(*b*) Describe any methods used to examine subgroups and interactions	Y
		(*c*) Explain how missing data were addressed	N
		(*d*) *Cohort study*—If applicable, explain how loss to follow-up was addressed<br>*Case-control study*—If applicable, explain how matching of cases and controls was addressed<br>*Cross-sectional study*—If applicable, describe analytical methods taking account of sampling strategy	N/A
		(*e*) Describe any sensitivity analyses	
**Results**			
Participants	13*	(a) Report numbers of individuals at each stage of study—eg numbers potentially eligible, examined for eligibility, confirmed eligible, included in the study, completing follow-up, and analysed	Y
		(b) Give reasons for non-participation at each stage	Y
		(c) Consider use of a flow diagram	N
Descriptive data	14*	(a) Give characteristics of study participants (eg demographic, clinical, social) and information on exposures and potential confounders	Y
		(b) Indicate number of participants with missing data for each variable of interest	N
		(c) *Cohort study*—Summarise follow-up time (eg, average and total amount)	N/A
Outcome data	15*	*Cohort study*—Report numbers of outcome events or summary measures over time	N/A
		Case-control study—Report numbers in each exposure category, or summary measures of exposure	N/A
		Cross-sectional study—Report numbers of outcome events or summary measures	Y
Main results	16	(*a*) Give unadjusted estimates and, if applicable, confounder-adjusted estimates and their precision (eg, 95% confidence interval). Make clear which confounders were adjusted for and why they were included	Y
		(*b*) Report category boundaries when continuous variables were categorized	N/A
		(*c*) If relevant, consider translating estimates of relative risk into absolute risk for a meaningful time period	N/A
Other analyses	17	Report other analyses done—eg analyses of subgroups and interactions, and sensitivity analyses	Y

**Discussion**			
Key results	18	Summarise key results with reference to study objectives	Y
Limitations	19	Discuss limitations of the study, taking into account sources of potential bias or imprecision. Discuss both direction and magnitude of any potential bias	Y
Interpretation	20	Give a cautious overall interpretation of results considering objectives, limitations, multiplicity of analyses, results from similar studies, and other relevant evidence	Y
Generalisability	21	Discuss the generalisability (external validity) of the study results	Y
**Other information**			
Funding	22	Give the source of funding and the role of the funders for the present study and, if applicable, for the original study on which the present article is based	Y

Huet, C.; Rosol, R.; Egeland, G.M. The prevalence of food insecurity is high and the diet quality poor in Inuit communities. J Nutr 2012, 142, 541-547, doi:10.3945/jn.111.149278.

STROBE Statement—checklist of items that should be included in reports of observational studies

	Item No	Recommendation	Recommendation met? (Y/N)
**Title and abstract**	1	(*a*) Indicate the study's design with a commonly used term in the title or the abstract	Y
		(*b*) Provide in the abstract an informative and balanced summary of what was done and what was found	Y
**Introduction**			
Background/rationale	2	Explain the scientific background and rationale for the investigation being reported	Y
Objectives	3	State specific objectives, including any prespecified hypotheses	Y
**Methods**			
Study design	4	Present key elements of study design early in the paper	Y
Setting	5	Describe the setting, locations, and relevant dates, including periods of recruitment, exposure, follow-up, and data collection	Y
Participants	6	(*a*) *Cohort study*—Give the eligibility criteria, and the sources and methods of selection of participants. Describe methods of follow-up<br>*Case-control study*—Give the eligibility criteria, and the sources and methods of case ascertainment and control selection. Give the rationale for the choice of cases and controls<br>*Cross-sectional study*—Give the eligibility criteria, and the sources and methods of selection of participants	Y
		(*b*) *Cohort study*—For matched studies, give matching criteria and number of exposed and unexposed<br>*Case-control study*—For matched studies, give matching criteria and the number of controls per case	N/A
Variables	7	Clearly define all outcomes, exposures, predictors, potential confounders, and effect modifiers. Give diagnostic criteria, if applicable	Y
Data sources/ measurement	8*	For each variable of interest, give sources of data and details of methods of assessment (measurement). Describe comparability of assessment methods if there is more than one group	Y
Bias	9	Describe any efforts to address potential sources of bias	N
Study size	10	Explain how the study size was arrived at	Y

Quantitative variables	11	Explain how quantitative variables were handled in the analyses. If applicable, describe which groupings were chosen and why	Y
Statistical methods	12	(*a*) Describe all statistical methods, including those used to control for confounding	Y
		(*b*) Describe any methods used to examine subgroups and interactions	Y
		(*c*) Explain how missing data were addressed	N
		(*d*) *Cohort study*—If applicable, explain how loss to follow-up was addressed<br>*Case-control study*—If applicable, explain how matching of cases and controls was addressed<br>*Cross-sectional study*—If applicable, describe analytical methods taking account of sampling strategy	N/A
		(*e*) Describe any sensitivity analyses	N
**Results**			
Participants	13*	(a) Report numbers of individuals at each stage of study—eg numbers potentially eligible, examined for eligibility, confirmed eligible, included in the study, completing follow-up, and analysed	Y
		(b) Give reasons for non-participation at each stage	Y
		(c) Consider use of a flow diagram	N
Descriptive data	14*	(a) Give characteristics of study participants (eg demographic, clinical, social) and information on exposures and potential confounders	Y
		(b) Indicate number of participants with missing data for each variable of interest	N
		(c) *Cohort study*—Summarise follow-up time (eg, average and total amount)	N/A
Outcome data	15*	*Cohort study*—Report numbers of outcome events or summary measures over time	N/A
		Case-control study—Report numbers in each exposure category, or summary measures of exposure	N/A
		Cross-sectional study—Report numbers of outcome events or summary measures	Y
Main results	16	(*a*) Give unadjusted estimates and, if applicable, confounder-adjusted estimates and their precision (eg, 95% confidence interval). Make clear which confounders were adjusted for and why they were included	Y
		(*b*) Report category boundaries when continuous variables were categorized	N/A
		(*c*) If relevant, consider translating estimates of relative risk into absolute risk for a meaningful time period	N/A
Other analyses	17	Report other analyses done—eg analyses of subgroups and interactions, and sensitivity analyses	Y
**Discussion**			

Key results	18	Summarise key results with reference to study objectives	Y
Limitations	19	Discuss limitations of the study, taking into account sources of potential bias or imprecision. Discuss both direction and magnitude of any potential bias	Y
Interpretation	20	Give a cautious overall interpretation of results considering objectives, limitations, multiplicity of analyses, results from similar studies, and other relevant evidence	Y
Generalisability	21	Discuss the generalisability (external validity) of the study results	N
**Other information**			
Funding	22	Give the source of funding and the role of the funders for the present study and, if applicable, for the original study on which the present article is based	Y

Skinner, K.; Hanning, R.M.; Desjardins, E.; Tsuji, L.J. Giving voice to food insecurity in a remote indigenous community in subarctic Ontario, Canada: traditional ways, ways to cope, ways forward. BMC Public Health 2013, 13, 427, doi:10.1186/1471-2458-13-427.

Standards for Reporting Qualitative Research (SRQR)

**No. Topic**	**Item**	**0 – no 1 – maybe 2-Yes**
**Title and abstract**		
S1 Title	Concise description of the nature and topic of the study identifying the study as qualitative or indicating the approach (e.g., ethnography, grounded theory) or data collection methods (e.g., interview, focus group) is recommended	2
S2 Abstract	Summary of key elements of the study using the abstract format of the intended publication; typically includes objective, methods, results, and conclusions	2
**Introduction**		
S3 Problem formulation	Description and significance of the problem/phenomenon studied; review of relevant theory and empirical work; problem statement	2
S4 Purpose or research question	Purpose of the study and specific objectives or questions	2
**Methods**		
S5 Qualitative approach and research paradigm	Qualitative approach (e.g., ethnography, grounded theory, case study, phenomenology, narrative research) and guiding theory if appropriate; identifying the research paradigm (e.g., positivist, constructivist/interpretivist) is also recommended	2
S6 Researcher characteristics and reflexivity	Researchers' characteristics that may influence the research, including personal attributes, qualifications/experience, relationship with participants, assumptions, or presuppositions; potential or actual interaction between researchers' characteristics and the research questions, approach, methods, results, or transferability	2
S7 Context	Setting/site and salient contextual factors; rationale[a]	2
S8 Sampling strategy	How and why research participants, documents, or events were selected; criteria for deciding when no further sampling was necessary (e.g., sampling saturation); rationale[a]	2
S9 Ethical issues pertaining to human subjects	Documentation of approval by an appropriate ethics review board and participant consent, or explanation for lack thereof; other confidentiality and data security issues	2
S10 Data collection methods	Types of data collected; details of data collection procedures including (as appropriate) start and stop dates of data collection	2

	and analysis, iterative process, triangulation of sources/methods, and modification of procedures in response to evolving study findings; rationale[a]	
S11 Data collection instruments and technologies	Description of instruments (e.g., interview guides, questionnaires) and devices (e.g., audio recorders) used for data collection; if/how the instrument(s) changed over the course of the study	2
S12 Units of study	Number and relevant characteristics of participants, documents, or events included in the study; level of participation (could be reported in results)	2
S13 Data processing	Methods for processing data prior to and during analysis, including transcription, data entry, data management and security, verification of data integrity, data coding, and anonymization/deidentification of excerpts	2
S14 Data analysis	Process by which inferences, themes, etc., were identified and developed, including researchers involved in data analysis; usually references a specific paradigm or approach; rationale[a]	2
S15 Techniques to enhance trustworthiness	Techniques to enhance trustworthiness and credibility of data analysis (e.g., member checking, audit trail, triangulation); rationale[a]	2
**Results/Findings**		
S16 Synthesis and interpretation	Main findings (e.g., interpretations, inferences, and themes); might include development of a theory or model, or integration with prior research or theory	2
S17 Links to empirical data	Evidence (e.g., quotes, field notes, text excerpts, photographs) to substantiate analytic findings	2
**Discussion**		
S18 Integration with prior work, implications, transferability, and contribution(s) to the field	Short summary of main findings; explanation of how findings and conclusions connect to, support, elaborate on, or challenge conclusions of earlier scholarship; discussion of scope of application/generalizability; identification of unique contribution(s) to scholarship in a discipline or field	2
S19 Limitations	Trustworthiness and limitations of findings	2
**Other**		
S20 Conflicts of interest	Potential sources of influence or perceived influence on study conduct and conclusions; how these were managed	2
S21 Funding	Sources of funding and other support; role of funders in data collection, interpretation, and reporting	2

Skinner, K.; Hanning, R.M.; Tsuji, L.J. Prevalence and severity of household food insecurity of First Nations people living in an on-reserve, sub-Arctic community within the Mushkegowuk Territory. Public Health Nutr 2014, 17, 31-39, doi:10.1017/S1368980013001705.

STROBE Statement—checklist of items that should be included in reports of observational studies

	Item No	Recommendation	Recommendation met? (Y/N)
**Title and abstract**	1	(*a*) Indicate the study's design with a commonly used term in the title or the abstract	Y
		(*b*) Provide in the abstract an informative and balanced summary of what was done and what was found	Y
**Introduction**			
Background/rationale	2	Explain the scientific background and rationale for the investigation being reported	Y
Objectives	3	State specific objectives, including any prespecified hypotheses	Y
**Methods**			
Study design	4	Present key elements of study design early in the paper	Y
Setting	5	Describe the setting, locations, and relevant dates, including periods of recruitment, exposure, follow-up, and data collection	Y
Participants	6	(*a*) *Cohort study*—Give the eligibility criteria, and the sources and methods of selection of participants. Describe methods of follow-up<br>*Case-control study*—Give the eligibility criteria, and the sources and methods of case ascertainment and control selection. Give the rationale for the choice of cases and controls<br>*Cross-sectional study*—Give the eligibility criteria, and the sources and methods of selection of participants	Y
		(*b*) *Cohort study*—For matched studies, give matching criteria and number of exposed and unexposed<br>*Case-control study*—For matched studies, give matching criteria and the number of controls per case	N/A
Variables	7	Clearly define all outcomes, exposures, predictors, potential confounders, and effect modifiers. Give diagnostic criteria, if applicable	Y
Data sources/ measurement	8*	For each variable of interest, give sources of data and details of methods of assessment (measurement). Describe comparability of assessment methods if there is more than one group	Y
Bias	9	Describe any efforts to address potential sources of bias	N

Study size	10	Explain how the study size was arrived at	Y
Quantitative variables	11	Explain how quantitative variables were handled in the analyses. If applicable, describe which groupings were chosen and why	Y
Statistical methods	12	(*a*) Describe all statistical methods, including those used to control for confounding	Y
		(*b*) Describe any methods used to examine subgroups and interactions	Y
		(*c*) Explain how missing data were addressed	N
		(*d*) *Cohort study*—If applicable, explain how loss to follow-up was addressed<br>*Case-control study*—If applicable, explain how matching of cases and controls was addressed<br>*Cross-sectional study*—If applicable, describe analytical methods taking account of sampling strategy	N/A
		(*e*) Describe any sensitivity analyses	N
**Results**			
Participants	13*	(a) Report numbers of individuals at each stage of study—eg numbers potentially eligible, examined for eligibility, confirmed eligible, included in the study, completing follow-up, and analysed	Y
		(b) Give reasons for non-participation at each stage	Y
		(c) Consider use of a flow diagram	N
Descriptive data	14*	(a) Give characteristics of study participants (eg demographic, clinical, social) and information on exposures and potential confounders	Y
		(b) Indicate number of participants with missing data for each variable of interest	N
		(c) *Cohort study*—Summarise follow-up time (eg, average and total amount)	N/A
Outcome data	15*	*Cohort study*—Report numbers of outcome events or summary measures over time	N/A
		Case-control study—Report numbers in each exposure category, or summary measures of exposure	N/A
		Cross-sectional study—Report numbers of outcome events or summary measures	Y
Main results	16	(*a*) Give unadjusted estimates and, if applicable, confounder-adjusted estimates and their precision (eg, 95% confidence interval). Make clear which confounders were adjusted for and why they were included	Y
		(*b*) Report category boundaries when continuous variables were categorized	N/A
		(*c*) If relevant, consider translating estimates of relative risk into absolute risk for a meaningful time period	N/A
Other analyses	17	Report other analyses done—eg analyses of subgroups and interactions, and sensitivity analyses	Y

**Discussion**			
Key results	18	Summarise key results with reference to study objectives	Y
Limitations	19	Discuss limitations of the study, taking into account sources of potential bias or imprecision. Discuss both direction and magnitude of any potential bias	Y
Interpretation	20	Give a cautious overall interpretation of results considering objectives, limitations, multiplicity of analyses, results from similar studies, and other relevant evidence	Y
Generalisability	21	Discuss the generalisability (external validity) of the study results	N
**Other information**			
Funding	22	Give the source of funding and the role of the funders for the present study and, if applicable, for the original study on which the present article is based	Y

Ready, E. Sharing-based social capital associated with harvest production and wealth in the Canadian Arctic. PLoS One 2018, 13, e0193759, doi:10.1371/journal.pone.0193759.

STROBE Statement—checklist of items that should be included in reports of observational studies

	Item No	Recommendation	Recommendation met? (Y/N)
**Title and abstract**	1	(*a*) Indicate the study's design with a commonly used term in the title or the abstract	Y
		(*b*) Provide in the abstract an informative and balanced summary of what was done and what was found	Y
**Introduction**			
Background/rationale	2	Explain the scientific background and rationale for the investigation being reported	Y
Objectives	3	State specific objectives, including any prespecified hypotheses	Y
**Methods**			
Study design	4	Present key elements of study design early in the paper	Y
Setting	5	Describe the setting, locations, and relevant dates, including periods of recruitment, exposure, follow-up, and data collection	Y
Participants	6	(*a*) *Cohort study*—Give the eligibility criteria, and the sources and methods of selection of participants. Describe methods of follow-up<br>*Case-control study*—Give the eligibility criteria, and the sources and methods of case ascertainment and control selection. Give the rationale for the choice of cases and controls<br>*Cross-sectional study*—Give the eligibility criteria, and the sources and methods of selection of participants	Y
		(*b*) *Cohort study*—For matched studies, give matching criteria and number of exposed and unexposed<br>*Case-control study*—For matched studies, give matching criteria and the number of controls per case	N/A
Variables	7	Clearly define all outcomes, exposures, predictors, potential confounders, and effect modifiers. Give diagnostic criteria, if applicable	Y
Data sources/ measurement	8*	For each variable of interest, give sources of data and details of methods of assessment (measurement). Describe comparability of assessment methods if there is more than one group	Y
Bias	9	Describe any efforts to address potential sources of bias	M/A
Study size	10	Explain how the study size was arrived at	Y

Quantitative variables	11	Explain how quantitative variables were handled in the analyses. If applicable, describe which groupings were chosen and why	Y
Statistical methods	12	(*a*) Describe all statistical methods, including those used to control for confounding	Y
		(*b*) Describe any methods used to examine subgroups and interactions	N/A
		(*c*) Explain how missing data were addressed	Y
		(*d*) *Cohort study*—If applicable, explain how loss to follow-up was addressed<br>*Case-control study*—If applicable, explain how matching of cases and controls was addressed<br>*Cross-sectional study*—If applicable, describe analytical methods taking account of sampling strategy	N/A
		(*e*) Describe any sensitivity analyses	N/A
**Results**			
Participants	13*	(a) Report numbers of individuals at each stage of study—eg numbers potentially eligible, examined for eligibility, confirmed eligible, included in the study, completing follow-up, and analysed	Y
		(b) Give reasons for non-participation at each stage	N
		(c) Consider use of a flow diagram	N
Descriptive data	14*	(a) Give characteristics of study participants (eg demographic, clinical, social) and information on exposures and potential confounders	Y
		(b) Indicate number of participants with missing data for each variable of interest	N/A
		(c) *Cohort study*—Summarise follow-up time (eg, average and total amount)	N/A
Outcome data	15*	*Cohort study*—Report numbers of outcome events or summary measures over time	N/A
		Case-control study—Report numbers in each exposure category, or summary measures of exposure	N/A
		Cross-sectional study—Report numbers of outcome events or summary measures	N
Main results	16	(*a*) Give unadjusted estimates and, if applicable, confounder-adjusted estimates and their precision (eg, 95% confidence interval). Make clear which confounders were adjusted for and why they were included	Y
		(*b*) Report category boundaries when continuous variables were categorized	N/A
		(*c*) If relevant, consider translating estimates of relative risk into absolute risk for a meaningful time period	N/A
Other analyses	17	Report other analyses done—eg analyses of subgroups and interactions, and sensitivity analyses	Y
**Discussion**			

Key results	18	Summarise key results with reference to study objectives	Y
Limitations	19	Discuss limitations of the study, taking into account sources of potential bias or imprecision. Discuss both direction and magnitude of any potential bias	N
Interpretation	20	Give a cautious overall interpretation of results considering objectives, limitations, multiplicity of analyses, results from similar studies, and other relevant evidence	Y
Generalisability	21	Discuss the generalisability (external validity) of the study results	N
**Other information**			
Funding	22	Give the source of funding and the role of the funders for the present study and, if applicable, for the original study on which the present article is based	Y

Teh, L.; Pirkle, C.; Furgal, C.; Fillion, M.; Lucas, M. Psychometric validation of the household food insecurity access scale among Inuit pregnant women from Northern Quebec. PLoS One 2017, 12, e0178708, doi:10.1371/journal.pone.0178708.

STROBE Statement—checklist of items that should be included in reports of observational studies

	Item No	Recommendation	Recommendation met? (Y/N)
**Title and abstract**	1	(*a*) Indicate the study's design with a commonly used term in the title or the abstract	Y
		(*b*) Provide in the abstract an informative and balanced summary of what was done and what was found	Y
**Introduction**			
Background/rationale	2	Explain the scientific background and rationale for the investigation being reported	Y
Objectives	3	State specific objectives, including any prespecified hypotheses	Y
**Methods**			
Study design	4	Present key elements of study design early in the paper	Y
Setting	5	Describe the setting, locations, and relevant dates, including periods of recruitment, exposure, follow-up, and data collection	Y
Participants	6	(*a*) *Cohort study*—Give the eligibility criteria, and the sources and methods of selection of participants. Describe methods of follow-up<br>*Case-control study*—Give the eligibility criteria, and the sources and methods of case ascertainment and control selection. Give the rationale for the choice of cases and controls<br>*Cross-sectional study*—Give the eligibility criteria, and the sources and methods of selection of participants	Y
		(*b*) *Cohort study*—For matched studies, give matching criteria and number of exposed and unexposed<br>*Case-control study*—For matched studies, give matching criteria and the number of controls per case	N/A
Variables	7	Clearly define all outcomes, exposures, predictors, potential confounders, and effect modifiers. Give diagnostic criteria, if applicable	Y
Data sources/ measurement	8*	For each variable of interest, give sources of data and details of methods of assessment (measurement). Describe comparability of assessment methods if there is more than one group	Y
Bias	9	Describe any efforts to address potential sources of bias	N

Study size	10	Explain how the study size was arrived at	N
Quantitative variables	11	Explain how quantitative variables were handled in the analyses. If applicable, describe which groupings were chosen and why	Y
Statistical methods	12	(*a*) Describe all statistical methods, including those used to control for confounding	Y
		(*b*) Describe any methods used to examine subgroups and interactions	Y
		(*c*) Explain how missing data were addressed	N
		(*d*) *Cohort study*—If applicable, explain how loss to follow-up was addressed<br>*Case-control study*—If applicable, explain how matching of cases and controls was addressed<br>*Cross-sectional study*—If applicable, describe analytical methods taking account of sampling strategy	N/A
		(*e*) Describe any sensitivity analyses	N
**Results**			
Participants	13*	(a) Report numbers of individuals at each stage of study—eg numbers potentially eligible, examined for eligibility, confirmed eligible, included in the study, completing follow-up, and analysed	Y
		(b) Give reasons for non-participation at each stage	N
		(c) Consider use of a flow diagram	N
Descriptive data	14*	(a) Give characteristics of study participants (eg demographic, clinical, social) and information on exposures and potential confounders	Y
		(b) Indicate number of participants with missing data for each variable of interest	N
		(c) *Cohort study*—Summarise follow-up time (eg, average and total amount)	N/A
Outcome data	15*	*Cohort study*—Report numbers of outcome events or summary measures over time	N/A
		Case-control study—Report numbers in each exposure category, or summary measures of exposure	N/A
		Cross-sectional study—Report numbers of outcome events or summary measures	Y
Main results	16	(*a*) Give unadjusted estimates and, if applicable, confounder-adjusted estimates and their precision (eg, 95% confidence interval). Make clear which confounders were adjusted for and why they were included	Y
		(*b*) Report category boundaries when continuous variables were categorized	N/A
		(*c*) If relevant, consider translating estimates of relative risk into absolute risk for a meaningful time period	N/A
Other analyses	17	Report other analyses done—eg analyses of subgroups and interactions, and sensitivity analyses	Y

**Discussion**			
Key results	18	Summarise key results with reference to study objectives	Y
Limitations	19	Discuss limitations of the study, taking into account sources of potential bias or imprecision. Discuss both direction and magnitude of any potential bias	Y
Interpretation	20	Give a cautious overall interpretation of results considering objectives, limitations, multiplicity of analyses, results from similar studies, and other relevant evidence	Y
Generalisability	21	Discuss the generalisability (external validity) of the study results	Y
**Other information**			
Funding	22	Give the source of funding and the role of the funders for the present study and, if applicable, for the original study on which the present article is based	Y

Ford, J.; Lardeau, M.P.; Vanderbilt, W. The characteristics and experience of community food program users in arctic Canada: a case study from Iqaluit, Nunavut. BMC Public Health 2012, 12, 464, doi:10.1186/1471-2458-12-464.

Standards for Reporting Qualitative Research (SRQR)

**No. Topic**	**Item**	**0 – no 1 – maybe 2-Yes**
**Title and abstract**		
S1 Title	Concise description of the nature and topic of the study identifying the study as qualitative or indicating the approach (e.g., ethnography, grounded theory) or data collection methods (e.g., interview, focus group) is recommended	2
S2 Abstract	Summary of key elements of the study using the abstract format of the intended publication; typically includes objective, methods, results, and conclusions	2
**Introduction**		
S3 Problem formulation	Description and significance of the problem/phenomenon studied; review of relevant theory and empirical work; problem statement	2
S4 Purpose or research question	Purpose of the study and specific objectives or questions	2
**Methods**		
S5 Qualitative approach and research paradigm	Qualitative approach (e.g., ethnography, grounded theory, case study, phenomenology, narrative research) and guiding theory if appropriate; identifying the research paradigm (e.g., positivist, constructivist/interpretivist) is also recommended	2
S6 Researcher characteristics and reflexivity	Researchers' characteristics that may influence the research, including personal attributes, qualifications/experience, relationship with participants, assumptions, or presuppositions; potential or actual interaction between researchers' characteristics and the research questions, approach, methods, results, or transferability	2
S7 Context	Setting/site and salient contextual factors; rationale[a]	2
S8 Sampling strategy	How and why research participants, documents, or events were selected; criteria for deciding when no further sampling was necessary (e.g., sampling saturation); rationale[a]	2
S9 Ethical issues pertaining to human subjects	Documentation of approval by an appropriate ethics review board and participant consent, or explanation for lack thereof; other confidentiality and data security issues	2
S10 Data collection methods	Types of data collected; details of data collection procedures including (as appropriate) start and stop dates of data collection	2

	and analysis, iterative process, triangulation of sources/methods, and modification of procedures in response to evolving study findings; rationale[a]	
S11 Data collection instruments and technologies	Description of instruments (e.g., interview guides, questionnaires) and devices (e.g., audio recorders) used for data collection; if/how the instrument(s) changed over the course of the study	2
S12 Units of study	Number and relevant characteristics of participants, documents, or events included in the study; level of participation (could be reported in results)	2
S13 Data processing	Methods for processing data prior to and during analysis, including transcription, data entry, data management and security, verification of data integrity, data coding, and anonymization/deidentification of excerpts	2
S14 Data analysis	Process by which inferences, themes, etc., were identified and developed, including researchers involved in data analysis; usually references a specific paradigm or approach; rationale[a]	2
S15 Techniques to enhance trustworthiness	Techniques to enhance trustworthiness and credibility of data analysis (e.g., member checking, audit trail, triangulation); rationale[a]	1
**Results/Findings**		
S16 Synthesis and interpretation	Main findings (e.g., interpretations, inferences, and themes); might include development of a theory or model, or integration with prior research or theory	2
S17 Links to empirical data	Evidence (e.g., quotes, field notes, text excerpts, photographs) to substantiate analytic findings	2
**Discussion**		
S18 Integration with prior work, implications, transferability, and contribution(s) to the field	Short summary of main findings; explanation of how findings and conclusions connect to, support, elaborate on, or challenge conclusions of earlier scholarship; discussion of scope of application/generalizability; identification of unique contribution(s) to scholarship in a discipline or field	2
S19 Limitations	Trustworthiness and limitations of findings	0
**Other**		
S20 Conflicts of interest	Potential sources of influence or perceived influence on study conduct and conclusions; how these were managed	2
S21 Funding	Sources of funding and other support; role of funders in data collection, interpretation, and reporting	2

Ford, J.D.; Lardeau, M.P.; Blackett, H.; Chatwood, S.; Kurszewski, D. Community food program use in Inuvik, Northwest Territories. BMC Public Health 2013, 13, 970, doi:10.1186/1471-2458-13-970.

Standards for Reporting Qualitative Research (SRQR)

**No. Topic**	**Item**	**0 – no 1 – maybe 2-Yes**
**Title and abstract**		
S1 Title	Concise description of the nature and topic of the study identifying the study as qualitative or indicating the approach (e.g., ethnography, grounded theory) or data collection methods (e.g., interview, focus group) is recommended	0
S2 Abstract	Summary of key elements of the study using the abstract format of the intended publication; typically includes objective, methods, results, and conclusions	2
**Introduction**		
S3 Problem formulation	Description and significance of the problem/phenomenon studied; review of relevant theory and empirical work; problem statement	2
S4 Purpose or research question	Purpose of the study and specific objectives or questions	2
**Methods**		
S5 Qualitative approach and research paradigm	Qualitative approach (e.g., ethnography, grounded theory, case study, phenomenology, narrative research) and guiding theory if appropriate; identifying the research paradigm (e.g., positivist, constructivist/interpretivist) is also recommended	2
S6 Researcher characteristics and reflexivity	Researchers' characteristics that may influence the research, including personal attributes, qualifications/experience, relationship with participants, assumptions, or presuppositions; potential or actual interaction between researchers' characteristics and the research questions, approach, methods, results, or transferability	2
S7 Context	Setting/site and salient contextual factors; rationale[a]	2
S8 Sampling strategy	How and why research participants, documents, or events were selected; criteria for deciding when no further sampling was necessary (e.g., sampling saturation); rationale[a]	2
S9 Ethical issues pertaining to human subjects	Documentation of approval by an appropriate ethics review board and participant consent, or explanation for lack thereof; other confidentiality and data security issues	2
S10 Data collection methods	Types of data collected; details of data collection procedures including (as appropriate) start and stop dates of data collection and analysis, iterative process, triangulation of sources/methods,	2

	and modification of procedures in response to evolving study findings; rationale[a]	
S11 Data collection instruments and technologies	Description of instruments (e.g., interview guides, questionnaires) and devices (e.g., audio recorders) used for data collection; if/how the instrument(s) changed over the course of the study	2
S12 Units of study	Number and relevant characteristics of participants, documents, or events included in the study; level of participation (could be reported in results)	2
S13 Data processing	Methods for processing data prior to and during analysis, including transcription, data entry, data management and security, verification of data integrity, data coding, and anonymization/deidentification of excerpts	2
S14 Data analysis	Process by which inferences, themes, etc., were identified and developed, including researchers involved in data analysis; usually references a specific paradigm or approach; rationale[a]	2
S15 Techniques to enhance trustworthiness	Techniques to enhance trustworthiness and credibility of data analysis (e.g., member checking, audit trail, triangulation); rationale[a]	2
**Results/Findings**		
S16 Synthesis and interpretation	Main findings (e.g., interpretations, inferences, and themes); might include development of a theory or model, or integration with prior research or theory	2
S17 Links to empirical data	Evidence (e.g., quotes, field notes, text excerpts, photographs) to substantiate analytic findings	2
**Discussion**		
S18 Integration with prior work, implications, transferability, and contribution(s) to the field	Short summary of main findings; explanation of how findings and conclusions connect to, support, elaborate on, or challenge conclusions of earlier scholarship; discussion of scope of application/generalizability; identification of unique contribution(s) to scholarship in a discipline or field	2
S19 Limitations	Trustworthiness and limitations of findings	2
**Other**		
S20 Conflicts of interest	Potential sources of influence or perceived influence on study conduct and conclusions; how these were managed	2
S21 Funding	Sources of funding and other support; role of funders in data collection, interpretation, and reporting	2

Chan, H.M.; Fediuk, K.; Hamilton, S.; Rostas, L.; Caughey, A.; Kuhnlein, H.; Egeland, G.; Loring, E. Food security in Nunavut, Canada: barriers and recommendations. Int J Circumpolar Health 2006, 65, 416-431, doi:10.3402/ijch.v65i5.18132.

Standards for Reporting Qualitative Research (SRQR)

**No. Topic**	**Item**	**0 – no 1 – maybe 2-Yes**
**Title and abstract**		
S1 Title	Concise description of the nature and topic of the study identifying the study as qualitative or indicating the approach (e.g., ethnography, grounded theory) or data collection methods (e.g., interview, focus group) is recommended	1
S2 Abstract	Summary of key elements of the study using the abstract format of the intended publication; typically includes objective, methods, results, and conclusions	2
**Introduction**		
S3 Problem formulation	Description and significance of the problem/phenomenon studied; review of relevant theory and empirical work; problem statement	2
S4 Purpose or research question	Purpose of the study and specific objectives or questions	2
**Methods**		
S5 Qualitative approach and research paradigm	Qualitative approach (e.g., ethnography, grounded theory, case study, phenomenology, narrative research) and guiding theory if appropriate; identifying the research paradigm (e.g., positivist, constructivist/interpretivist) is also recommended	2
S6 Researcher characteristics and reflexivity	Researchers' characteristics that may influence the research, including personal attributes, qualifications/experience, relationship with participants, assumptions, or presuppositions; potential or actual interaction between researchers' characteristics and the research questions, approach, methods, results, or transferability	1
S7 Context	Setting/site and salient contextual factors; rationale[a]	2
S8 Sampling strategy	How and why research participants, documents, or events were selected; criteria for deciding when no further sampling was necessary (e.g., sampling saturation); rationale[a]	1
S9 Ethical issues pertaining to human subjects	Documentation of approval by an appropriate ethics review board and participant consent, or explanation for lack thereof; other confidentiality and data security issues	2
S10 Data collection methods	Types of data collected; details of data collection procedures including (as appropriate) start and stop dates of data collection	2

	and analysis, iterative process, triangulation of sources/methods, and modification of procedures in response to evolving study findings; rationale[a]	
S11 Data collection instruments and technologies	Description of instruments (e.g., interview guides, questionnaires) and devices (e.g., audio recorders) used for data collection; if/how the instrument(s) changed over the course of the study	2
S12 Units of study	Number and relevant characteristics of participants, documents, or events included in the study; level of participation (could be reported in results)	2
S13 Data processing	Methods for processing data prior to and during analysis, including transcription, data entry, data management and security, verification of data integrity, data coding, and anonymization/deidentification of excerpts	2
S14 Data analysis	Process by which inferences, themes, etc., were identified and developed, including researchers involved in data analysis; usually references a specific paradigm or approach; rationale[a]	2
S15 Techniques to enhance trustworthiness	Techniques to enhance trustworthiness and credibility of data analysis (e.g., member checking, audit trail, triangulation); rationale[a]	2
**Results/Findings**		
S16 Synthesis and interpretation	Main findings (e.g., interpretations, inferences, and themes); might include development of a theory or model, or integration with prior research or theory	2
S17 Links to empirical data	Evidence (e.g., quotes, field notes, text excerpts, photographs) to substantiate analytic findings	2
**Discussion**		
S18 Integration with prior work, implications, transferability, and contribution(s) to the field	Short summary of main findings; explanation of how findings and conclusions connect to, support, elaborate on, or challenge conclusions of earlier scholarship; discussion of scope of application/generalizability; identification of unique contribution(s) to scholarship in a discipline or field	2
S19 Limitations	Trustworthiness and limitations of findings	2
**Other**		
S20 Conflicts of interest	Potential sources of influence or perceived influence on study conduct and conclusions; how these were managed	2
S21 Funding	Sources of funding and other support; role of funders in data collection, interpretation, and reporting	2

Lambden, J.; Receveur, O.; Kuhnlein, H.V. Traditional food attributes must be included in studies of food security in the Canadian Arctic. Int J Circumpolar Health 2007, 66, 308-319, doi:10.3402/ijch.v66i4.18272.

STROBE Statement—checklist of items that should be included in reports of observational studies

	Item No	Recommendation	Recommendation met? (Y/N)
**Title and abstract**	1	(*a*) Indicate the study's design with a commonly used term in the title or the abstract	Y
		(*b*) Provide in the abstract an informative and balanced summary of what was done and what was found	Y
**Introduction**			
Background/rationale	2	Explain the scientific background and rationale for the investigation being reported	Y
Objectives	3	State specific objectives, including any prespecified hypotheses	Y
**Methods**			
Study design	4	Present key elements of study design early in the paper	Y
Setting	5	Describe the setting, locations, and relevant dates, including periods of recruitment, exposure, follow-up, and data collection	Y
Participants	6	(*a*) *Cohort study*—Give the eligibility criteria, and the sources and methods of selection of participants. Describe methods of follow-up<br>*Case-control study*—Give the eligibility criteria, and the sources and methods of case ascertainment and control selection. Give the rationale for the choice of cases and controls<br>*Cross-sectional study*—Give the eligibility criteria, and the sources and methods of selection of participants	Y
		(*b*) *Cohort study*—For matched studies, give matching criteria and number of exposed and unexposed<br>*Case-control study*—For matched studies, give matching criteria and the number of controls per case	N/A
Variables	7	Clearly define all outcomes, exposures, predictors, potential confounders, and effect modifiers. Give diagnostic criteria, if applicable	Y
Data sources/ measurement	8*	For each variable of interest, give sources of data and details of methods of assessment (measurement). Describe comparability of assessment methods if there is more than one group	Y
Bias	9	Describe any efforts to address potential sources of bias	N

Study size	10	Explain how the study size was arrived at	N/A
Quantitative variables	11	Explain how quantitative variables were handled in the analyses. If applicable, describe which groupings were chosen and why	Y
Statistical methods	12	(*a*) Describe all statistical methods, including those used to control for confounding	Y
		(*b*) Describe any methods used to examine subgroups and interactions	Y
		(*c*) Explain how missing data were addressed	N
		(*d*) *Cohort study*—If applicable, explain how loss to follow-up was addressed<br>*Case-control study*—If applicable, explain how matching of cases and controls was addressed<br>*Cross-sectional study*—If applicable, describe analytical methods taking account of sampling strategy	N/A
		(*e*) Describe any sensitivity analyses	N
**Results**			
Participants	13*	(a) Report numbers of individuals at each stage of study—eg numbers potentially eligible, examined for eligibility, confirmed eligible, included in the study, completing follow-up, and analysed	Y
		(b) Give reasons for non-participation at each stage	N
		(c) Consider use of a flow diagram	N
Descriptive data	14*	(a) Give characteristics of study participants (eg demographic, clinical, social) and information on exposures and potential confounders	Y
		(b) Indicate number of participants with missing data for each variable of interest	N/A
		(c) *Cohort study*—Summarise follow-up time (eg, average and total amount)	N/A
Outcome data	15*	*Cohort study*—Report numbers of outcome events or summary measures over time	N/A
		Case-control study—Report numbers in each exposure category, or summary measures of exposure	N/A
		Cross-sectional study—Report numbers of outcome events or summary measures	Y
Main results	16	(*a*) Give unadjusted estimates and, if applicable, confounder-adjusted estimates and their precision (eg, 95% confidence interval). Make clear which confounders were adjusted for and why they were included	Y
		(*b*) Report category boundaries when continuous variables were categorized	N/A
		(*c*) If relevant, consider translating estimates of relative risk into absolute risk for a meaningful time period	N/A
Other analyses	17	Report other analyses done—eg analyses of subgroups and interactions, and sensitivity analyses	Y

**Discussion**			
Key results	18	Summarise key results with reference to study objectives	Y
Limitations	19	Discuss limitations of the study, taking into account sources of potential bias or imprecision. Discuss both direction and magnitude of any potential bias	N
Interpretation	20	Give a cautious overall interpretation of results considering objectives, limitations, multiplicity of analyses, results from similar studies, and other relevant evidence	Y
Generalisability	21	Discuss the generalisability (external validity) of the study results	N
**Other information**			
Funding	22	Give the source of funding and the role of the funders for the present study and, if applicable, for the original study on which the present article is based	N

Lambden, J.; Receveur, O.; Marshall, J.; Kuhnlein, H.V. Traditional and market food access in Arctic Canada is affected by economic factors. Int J Circumpolar Health 2006, 65, 331-340, doi:10.3402/ijch.v65i4.18117.

STROBE Statement—checklist of items that should be included in reports of observational studies

	Item No	Recommendation	Recommendation met? (Y/N)
**Title and abstract**	1	(*a*) Indicate the study's design with a commonly used term in the title or the abstract	Y
		(*b*) Provide in the abstract an informative and balanced summary of what was done and what was found	Y
**Introduction**			
Background/rationale	2	Explain the scientific background and rationale for the investigation being reported	Y
Objectives	3	State specific objectives, including any prespecified hypotheses	Y
**Methods**			
Study design	4	Present key elements of study design early in the paper	Y
Setting	5	Describe the setting, locations, and relevant dates, including periods of recruitment, exposure, follow-up, and data collection	Y
Participants	6	(*a*) *Cohort study*—Give the eligibility criteria, and the sources and methods of selection of participants. Describe methods of follow-up<br>*Case-control study*—Give the eligibility criteria, and the sources and methods of case ascertainment and control selection. Give the rationale for the choice of cases and controls<br>*Cross-sectional study*—Give the eligibility criteria, and the sources and methods of selection of participants	Y
		(*b*) *Cohort study*—For matched studies, give matching criteria and number of exposed and unexposed<br>*Case-control study*—For matched studies, give matching criteria and the number of controls per case	N/A
Variables	7	Clearly define all outcomes, exposures, predictors, potential confounders, and effect modifiers. Give diagnostic criteria, if applicable	Y
Data sources/ measurement	8*	For each variable of interest, give sources of data and details of methods of assessment (measurement). Describe comparability of assessment methods if there is more than one group	Y
Bias	9	Describe any efforts to address potential sources of bias	Y

Study size	10	Explain how the study size was arrived at	N/A
Quantitative variables	11	Explain how quantitative variables were handled in the analyses. If applicable, describe which groupings were chosen and why	Y
Statistical methods	12	(*a*) Describe all statistical methods, including those used to control for confounding	Y
		(*b*) Describe any methods used to examine subgroups and interactions	Y
		(*c*) Explain how missing data were addressed	N
		(*d*) *Cohort study*—If applicable, explain how loss to follow-up was addressed<br>*Case-control study*—If applicable, explain how matching of cases and controls was addressed<br>*Cross-sectional study*—If applicable, describe analytical methods taking account of sampling strategy	N/A
		(*e*) Describe any sensitivity analyses	N
**Results**			
Participants	13*	(a) Report numbers of individuals at each stage of study—eg numbers potentially eligible, examined for eligibility, confirmed eligible, included in the study, completing follow-up, and analysed	Y
		(b) Give reasons for non-participation at each stage	N
		(c) Consider use of a flow diagram	N
Descriptive data	14*	(a) Give characteristics of study participants (eg demographic, clinical, social) and information on exposures and potential confounders	Y
		(b) Indicate number of participants with missing data for each variable of interest	N/A
		(c) *Cohort study*—Summarise follow-up time (eg, average and total amount)	N/A
Outcome data	15*	*Cohort study*—Report numbers of outcome events or summary measures over time	N/A
		Case-control study—Report numbers in each exposure category, or summary measures of exposure	N/A
		Cross-sectional study—Report numbers of outcome events or summary measures	Y
Main results	16	(*a*) Give unadjusted estimates and, if applicable, confounder-adjusted estimates and their precision (eg, 95% confidence interval). Make clear which confounders were adjusted for and why they were included	Y
		(*b*) Report category boundaries when continuous variables were categorized	N/A
		(*c*) If relevant, consider translating estimates of relative risk into absolute risk for a meaningful time period	N/A
Other analyses	17	Report other analyses done—eg analyses of subgroups and interactions, and sensitivity analyses	Y

**Discussion**			
Key results	18	Summarise key results with reference to study objectives	Y
Limitations	19	Discuss limitations of the study, taking into account sources of potential bias or imprecision. Discuss both direction and magnitude of any potential bias	N
Interpretation	20	Give a cautious overall interpretation of results considering objectives, limitations, multiplicity of analyses, results from similar studies, and other relevant evidence	Y
Generalisability	21	Discuss the generalisability (external validity) of the study results	N
**Other information**			
Funding	22	Give the source of funding and the role of the funders for the present study and, if applicable, for the original study on which the present article is based	N

Skinner, K.; Hanning, R.M.; Tsuji, L.J. Barriers and supports for healthy eating and physical activity for First Nation youths in northern Canada. Int J Circumpolar Health 2006, 65, 148-161, doi:10.3402/ijch.v65i2.18095.

Standards for Reporting Qualitative Research (SRQR)

**No. Topic**	**Item**	**0 – no 1 – maybe 2-Yes**
**Title and abstract**		
S1 Title	Concise description of the nature and topic of the study identifying the study as qualitative or indicating the approach (e.g., ethnography, grounded theory) or data collection methods (e.g., interview, focus group) is recommended	1
S2 Abstract	Summary of key elements of the study using the abstract format of the intended publication; typically includes objective, methods, results, and conclusions	2
**Introduction**		
S3 Problem formulation	Description and significance of the problem/phenomenon studied; review of relevant theory and empirical work; problem statement	2
S4 Purpose or research question	Purpose of the study and specific objectives or questions	2
**Methods**		
S5 Qualitative approach and research paradigm	Qualitative approach (e.g., ethnography, grounded theory, case study, phenomenology, narrative research) and guiding theory if appropriate; identifying the research paradigm (e.g., positivist, constructivist/interpretivist) is also recommended	2
S6 Researcher characteristics and reflexivity	Researchers' characteristics that may influence the research, including personal attributes, qualifications/experience, relationship with participants, assumptions, or presuppositions; potential or actual interaction between researchers' characteristics and the research questions, approach, methods, results, or transferability	2
S7 Context	Setting/site and salient contextual factors; rationale[a]	2
S8 Sampling strategy	How and why research participants, documents, or events were selected; criteria for deciding when no further sampling was necessary (e.g., sampling saturation); rationale[a]	2
S9 Ethical issues pertaining to human subjects	Documentation of approval by an appropriate ethics review board and participant consent, or explanation for lack thereof; other confidentiality and data security issues	2
S10 Data collection methods	Types of data collected; details of data collection procedures including (as appropriate) start and stop dates of data collection	2

	and analysis, iterative process, triangulation of sources/methods, and modification of procedures in response to evolving study findings; rationale[a]	
S11 Data collection instruments and technologies	Description of instruments (e.g., interview guides, questionnaires) and devices (e.g., audio recorders) used for data collection; if/how the instrument(s) changed over the course of the study	2
S12 Units of study	Number and relevant characteristics of participants, documents, or events included in the study; level of participation (could be reported in results)	2
S13 Data processing	Methods for processing data prior to and during analysis, including transcription, data entry, data management and security, verification of data integrity, data coding, and anonymization/deidentification of excerpts	2
S14 Data analysis	Process by which inferences, themes, etc., were identified and developed, including researchers involved in data analysis; usually references a specific paradigm or approach; rationale[a]	2
S15 Techniques to enhance trustworthiness	Techniques to enhance trustworthiness and credibility of data analysis (e.g., member checking, audit trail, triangulation); rationale[a]	2
**Results/Findings**		
S16 Synthesis and interpretation	Main findings (e.g., interpretations, inferences, and themes); might include development of a theory or model, or integration with prior research or theory	2
S17 Links to empirical data	Evidence (e.g., quotes, field notes, text excerpts, photographs) to substantiate analytic findings	2
**Discussion**		
S18 Integration with prior work, implications, transferability, and contribution(s) to the field	Short summary of main findings; explanation of how findings and conclusions connect to, support, elaborate on, or challenge conclusions of earlier scholarship; discussion of scope of application/generalizability; identification of unique contribution(s) to scholarship in a discipline or field	2
S19 Limitations	Trustworthiness and limitations of findings	0
**Other**		
S20 Conflicts of interest	Potential sources of influence or perceived influence on study conduct and conclusions; how these were managed	2
S21 Funding	Sources of funding and other support; role of funders in data collection, interpretation, and reporting	2

Collings, P.; Marten, M.G.; Pearce, T.; Young, A.G. Country food sharing networks, household structure, and implications for understanding food insecurity in Arctic Canada. Ecol Food Nutr 2016, 55, 30-49, doi:10.1080/03670244.2015.1072812.

STROBE Statement—checklist of items that should be included in reports of observational studies

	Item No	Recommendation	Recommendation met? (Y/N)
**Title and abstract**	1	(*a*) Indicate the study's design with a commonly used term in the title or the abstract	Y
		(*b*) Provide in the abstract an informative and balanced summary of what was done and what was found	Y
**Introduction**			
Background/rationale	2	Explain the scientific background and rationale for the investigation being reported	Y
Objectives	3	State specific objectives, including any prespecified hypotheses	Y
**Methods**			
Study design	4	Present key elements of study design early in the paper	Y
Setting	5	Describe the setting, locations, and relevant dates, including periods of recruitment, exposure, follow-up, and data collection	Y
Participants	6	(*a*) *Cohort study*—Give the eligibility criteria, and the sources and methods of selection of participants. Describe methods of follow-up<br>*Case-control study*—Give the eligibility criteria, and the sources and methods of case ascertainment and control selection. Give the rationale for the choice of cases and controls<br>*Cross-sectional study*—Give the eligibility criteria, and the sources and methods of selection of participants	Y
		(*b*) *Cohort study*—For matched studies, give matching criteria and number of exposed and unexposed<br>*Case-control study*—For matched studies, give matching criteria and the number of controls per case	N/A
Variables	7	Clearly define all outcomes, exposures, predictors, potential confounders, and effect modifiers. Give diagnostic criteria, if applicable	N
Data sources/ measurement	8*	For each variable of interest, give sources of data and details of methods of assessment (measurement). Describe comparability of assessment methods if there is more than one group	N
Bias	9	Describe any efforts to address potential sources of bias	N/A

Study size	10	Explain how the study size was arrived at	/A
Quantitative variables	11	Explain how quantitative variables were handled in the analyses. If applicable, describe which groupings were chosen and why	N
Statistical methods	12	(*a*) Describe all statistical methods, including those used to control for confounding	N
		(*b*) Describe any methods used to examine subgroups and interactions	N
		(*c*) Explain how missing data were addressed	N
		(*d*) *Cohort study*—If applicable, explain how loss to follow-up was addressed<br>*Case-control study*—If applicable, explain how matching of cases and controls was addressed<br>*Cross-sectional study*—If applicable, describe analytical methods taking account of sampling strategy	N/A
		(*e*) Describe any sensitivity analyses	
**Results**			
Participants	13*	(a) Report numbers of individuals at each stage of study—eg numbers potentially eligible, examined for eligibility, confirmed eligible, included in the study, completing follow-up, and analysed	Y
		(b) Give reasons for non-participation at each stage	Y
		(c) Consider use of a flow diagram	N
Descriptive data	14*	(a) Give characteristics of study participants (eg demographic, clinical, social) and information on exposures and potential confounders	Y
		(b) Indicate number of participants with missing data for each variable of interest	N
		(c) *Cohort study*—Summarise follow-up time (eg, average and total amount)	N/A
Outcome data	15*	*Cohort study*—Report numbers of outcome events or summary measures over time	N/A
		Case-control study—Report numbers in each exposure category, or summary measures of exposure	N/A
		Cross-sectional study—Report numbers of outcome events or summary measures	Y
Main results	16	(*a*) Give unadjusted estimates and, if applicable, confounder-adjusted estimates and their precision (eg, 95% confidence interval). Make clear which confounders were adjusted for and why they were included	Y
		(*b*) Report category boundaries when continuous variables were categorized	N/A
		(*c*) If relevant, consider translating estimates of relative risk into absolute risk for a meaningful time period	N/A
Other analyses	17	Report other analyses done—eg analyses of subgroups and interactions, and sensitivity analyses	Y

**Discussion**			
Key results	18	Summarise key results with reference to study objectives	Y
Limitations	19	Discuss limitations of the study, taking into account sources of potential bias or imprecision. Discuss both direction and magnitude of any potential bias	Y
Interpretation	20	Give a cautious overall interpretation of results considering objectives, limitations, multiplicity of analyses, results from similar studies, and other relevant evidence	Y
Generalisability	21	Discuss the generalisability (external validity) of the study results	N/A
**Other information**			
Funding	22	Give the source of funding and the role of the funders for the present study and, if applicable, for the original study on which the present article is based	Y

Organ, J.; Castleden, H.; Furgal, C.; Sheldon, T.; Hart, C. Contemporary programs in support of traditional ways: Inuit perspectives on community freezers as a mechanism to alleviate pressures of wild food access in Nain, Nunatsiavut. Health Place 2014, 30, 251-259, doi:10.1016/j.healthplace.2014.09.012.

Standards for Reporting Qualitative Research (SRQR)

**No. Topic**	**Item**	**0 – no 1 – maybe 2-Yes**
**Title and abstract**		
S1 Title	Concise description of the nature and topic of the study identifying the study as qualitative or indicating the approach (e.g., ethnography, grounded theory) or data collection methods (e.g., interview, focus group) is recommended	2
S2 Abstract	Summary of key elements of the study using the abstract format of the intended publication; typically includes objective, methods, results, and conclusions	2
**Introduction**		
S3 Problem formulation	Description and significance of the problem/phenomenon studied; review of relevant theory and empirical work; problem statement	2
S4 Purpose or research question	Purpose of the study and specific objectives or questions	2
**Methods**		
S5 Qualitative approach and research paradigm	Qualitative approach (e.g., ethnography, grounded theory, case study, phenomenology, narrative research) and guiding theory if appropriate; identifying the research paradigm (e.g., positivist, constructivist/interpretivist) is also recommended	2
S6 Researcher characteristics and reflexivity	Researchers' characteristics that may influence the research, including personal attributes, qualifications/experience, relationship with participants, assumptions, or presuppositions; potential or actual interaction between researchers' characteristics and the research questions, approach, methods, results, or transferability	2
S7 Context	Setting/site and salient contextual factors; rationale[a]	2
S8 Sampling strategy	How and why research participants, documents, or events were selected; criteria for deciding when no further sampling was necessary (e.g., sampling saturation); rationale[a]	2
S9 Ethical issues pertaining to human subjects	Documentation of approval by an appropriate ethics review board and participant consent, or explanation for lack thereof; other confidentiality and data security issues	2
S10 Data collection methods	Types of data collected; details of data collection procedures including (as appropriate) start and stop dates of data collection	2

	and analysis, iterative process, triangulation of sources/methods, and modification of procedures in response to evolving study findings; rationale[a]	
S11 Data collection instruments and technologies	Description of instruments (e.g., interview guides, questionnaires) and devices (e.g., audio recorders) used for data collection; if/how the instrument(s) changed over the course of the study	2
S12 Units of study	Number and relevant characteristics of participants, documents, or events included in the study; level of participation (could be reported in results)	2
S13 Data processing	Methods for processing data prior to and during analysis, including transcription, data entry, data management and security, verification of data integrity, data coding, and anonymization/deidentification of excerpts	2
S14 Data analysis	Process by which inferences, themes, etc., were identified and developed, including researchers involved in data analysis; usually references a specific paradigm or approach; rationale[a]	2
S15 Techniques to enhance trustworthiness	Techniques to enhance trustworthiness and credibility of data analysis (e.g., member checking, audit trail, triangulation); rationale[a]	2
**Results/Findings**		
S16 Synthesis and interpretation	Main findings (e.g., interpretations, inferences, and themes); might include development of a theory or model, or integration with prior research or theory	2
S17 Links to empirical data	Evidence (e.g., quotes, field notes, text excerpts, photographs) to substantiate analytic findings	2
**Discussion**		
S18 Integration with prior work, implications, transferability, and contribution(s) to the field	Short summary of main findings; explanation of how findings and conclusions connect to, support, elaborate on, or challenge conclusions of earlier scholarship; discussion of scope of application/generalizability; identification of unique contribution(s) to scholarship in a discipline or field	2
S19 Limitations	Trustworthiness and limitations of findings	0
**Other**		
S20 Conflicts of interest	Potential sources of influence or perceived influence on study conduct and conclusions; how these were managed	0
S21 Funding	Sources of funding and other support; role of funders in data collection, interpretation, and reporting	0

Spiegelaar, N.F.; Tsuji, L.J. Impact of Euro-Canadian agrarian practices: in search of sustainable import-substitution strategies to enhance food security in subarctic Ontario, Canada. Rural Remote Health 2013, 13, 2211.

Standards for Reporting Qualitative Research (SRQR)

**No. Topic**	**Item**	**0 – no 1 – maybe 2-Yes**
**Title and abstract**		
S1 Title	Concise description of the nature and topic of the study identifying the study as qualitative or indicating the approach (e.g., ethnography, grounded theory) or data collection methods (e.g., interview, focus group) is recommended	1
S2 Abstract	Summary of key elements of the study using the abstract format of the intended publication; typically includes objective, methods, results, and conclusions	2
**Introduction**		
S3 Problem formulation	Description and significance of the problem/phenomenon studied; review of relevant theory and empirical work; problem statement	2
S4 Purpose or research question	Purpose of the study and specific objectives or questions	2
**Methods**		
S5 Qualitative approach and research paradigm	Qualitative approach (e.g., ethnography, grounded theory, case study, phenomenology, narrative research) and guiding theory if appropriate; identifying the research paradigm (e.g., positivist, constructivist/interpretivist) is also recommended	1
S6 Researcher characteristics and reflexivity	Researchers' characteristics that may influence the research, including personal attributes, qualifications/experience, relationship with participants, assumptions, or presuppositions; potential or actual interaction between researchers' characteristics and the research questions, approach, methods, results, or transferability	2
S7 Context	Setting/site and salient contextual factors; rationale[a]	2
S8 Sampling strategy	How and why research participants, documents, or events were selected; criteria for deciding when no further sampling was necessary (e.g., sampling saturation); rationale[a]	2
S9 Ethical issues pertaining to human subjects	Documentation of approval by an appropriate ethics review board and participant consent, or explanation for lack thereof; other confidentiality and data security issues	2
S10 Data collection methods	Types of data collected; details of data collection procedures including (as appropriate) start and stop dates of data collection	2

	and analysis, iterative process, triangulation of sources/methods, and modification of procedures in response to evolving study findings; rationale[a]	
S11 Data collection instruments and technologies	Description of instruments (e.g., interview guides, questionnaires) and devices (e.g., audio recorders) used for data collection; if/how the instrument(s) changed over the course of the study	2
S12 Units of study	Number and relevant characteristics of participants, documents, or events included in the study; level of participation (could be reported in results)	2
S13 Data processing	Methods for processing data prior to and during analysis, including transcription, data entry, data management and security, verification of data integrity, data coding, and anonymization/deidentification of excerpts	2
S14 Data analysis	Process by which inferences, themes, etc., were identified and developed, including researchers involved in data analysis; usually references a specific paradigm or approach; rationale[a]	2
S15 Techniques to enhance trustworthiness	Techniques to enhance trustworthiness and credibility of data analysis (e.g., member checking, audit trail, triangulation); rationale[a]	0
**Results/Findings**		
S16 Synthesis and interpretation	Main findings (e.g., interpretations, inferences, and themes); might include development of a theory or model, or integration with prior research or theory	2
S17 Links to empirical data	Evidence (e.g., quotes, field notes, text excerpts, photographs) to substantiate analytic findings	2
**Discussion**		
S18 Integration with prior work, implications, transferability, and contribution(s) to the field	Short summary of main findings; explanation of how findings and conclusions connect to, support, elaborate on, or challenge conclusions of earlier scholarship; discussion of scope of application/generalizability; identification of unique contribution(s) to scholarship in a discipline or field	2
S19 Limitations	Trustworthiness and limitations of findings	0
**Other**		
S20 Conflicts of interest	Potential sources of influence or perceived influence on study conduct and conclusions; how these were managed	0
S21 Funding	Sources of funding and other support; role of funders in data collection, interpretation, and reporting	0

Nancarrow, T.L.; Chan, H.M. Observations of environmental changes and potential dietary impacts in two communities in Nunavut, Canada. Rural Remote Health 2010, 10, 1370.

Standards for Reporting Qualitative Research (SRQR)

No. Topic	Item	0 – no<br>1 – maybe<br>2-Yes
**Title and abstract**		
S1 Title	Concise description of the nature and topic of the study identifying the study as qualitative or indicating the approach (e.g., ethnography, grounded theory) or data collection methods (e.g., interview, focus group) is recommended	2
S2 Abstract	Summary of key elements of the study using the abstract format of the intended publication; typically includes objective, methods, results, and conclusions	2
**Introduction**		
S3 Problem formulation	Description and significance of the problem/phenomenon studied; review of relevant theory and empirical work; problem statement	2
S4 Purpose or research question	Purpose of the study and specific objectives or questions	2
**Methods**		
S5 Qualitative approach and research paradigm	Qualitative approach (e.g., ethnography, grounded theory, case study, phenomenology, narrative research) and guiding theory if appropriate; identifying the research paradigm (e.g., positivist, constructivist/interpretivist) is also recommended	1
S6 Researcher characteristics and reflexivity	Researchers' characteristics that may influence the research, including personal attributes, qualifications/experience, relationship with participants, assumptions, or presuppositions; potential or actual interaction between researchers' characteristics and the research questions, approach, methods, results, or transferability	0
S7 Context	Setting/site and salient contextual factors; rationale[a]	2
S8 Sampling strategy	How and why research participants, documents, or events were selected; criteria for deciding when no further sampling was necessary (e.g., sampling saturation); rationale[a]	2
S9 Ethical issues pertaining to human subjects	Documentation of approval by an appropriate ethics review board and participant consent, or explanation for lack thereof; other confidentiality and data security issues	2
S10 Data collection methods	Types of data collected; details of data collection procedures including (as appropriate) start and stop dates of data collection and analysis, iterative process, triangulation of sources/methods,	2

	and modification of procedures in response to evolving study findings; rationale[a]	
S11 Data collection instruments and technologies	Description of instruments (e.g., interview guides, questionnaires) and devices (e.g., audio recorders) used for data collection; if/how the instrument(s) changed over the course of the study	2
S12 Units of study	Number and relevant characteristics of participants, documents, or events included in the study; level of participation (could be reported in results)	2
S13 Data processing	Methods for processing data prior to and during analysis, including transcription, data entry, data management and security, verification of data integrity, data coding, and anonymization/deidentification of excerpts	2
S14 Data analysis	Process by which inferences, themes, etc., were identified and developed, including researchers involved in data analysis; usually references a specific paradigm or approach; rationale[a]	2
S15 Techniques to enhance trustworthiness	Techniques to enhance trustworthiness and credibility of data analysis (e.g., member checking, audit trail, triangulation); rationale[a]	2
**Results/Findings**		
S16 Synthesis and interpretation	Main findings (e.g., interpretations, inferences, and themes); might include development of a theory or model, or integration with prior research or theory	2
S17 Links to empirical data	Evidence (e.g., quotes, field notes, text excerpts, photographs) to substantiate analytic findings	2
**Discussion**		
S18 Integration with prior work, implications, transferability, and contribution(s) to the field	Short summary of main findings; explanation of how findings and conclusions connect to, support, elaborate on, or challenge conclusions of earlier scholarship; discussion of scope of application/generalizability; identification of unique contribution(s) to scholarship in a discipline or field	2
S19 Limitations	Trustworthiness and limitations of findings	0
**Other**		
S20 Conflicts of interest	Potential sources of influence or perceived influence on study conduct and conclusions; how these were managed	2
S21 Funding	Sources of funding and other support; role of funders in data collection, interpretation, and reporting	2

Wesche, S.D.; Chan, H.M. Adapting to the impacts of climate change on food security among Inuit in the Western Canadian Arctic. Ecohealth 2010, 7, 361-373, doi:10.1007/s10393-010-0344-8.

Standards for Reporting Qualitative Research (SRQR)

**No. Topic**	**Item**	**0 – no<br>1 – maybe<br>2-Yes**
**Title and abstract**		
S1 Title	Concise description of the nature and topic of the study identifying the study as qualitative or indicating the approach (e.g., ethnography, grounded theory) or data collection methods (e.g., interview, focus group) is recommended	0
S2 Abstract	Summary of key elements of the study using the abstract format of the intended publication; typically includes objective, methods, results, and conclusions	1
**Introduction**		
S3 Problem formulation	Description and significance of the problem/phenomenon studied; review of relevant theory and empirical work; problem statement	1
S4 Purpose or research question	Purpose of the study and specific objectives or questions	1
**Methods**		
S5 Qualitative approach and research paradigm	Qualitative approach (e.g., ethnography, grounded theory, case study, phenomenology, narrative research) and guiding theory if appropriate; identifying the research paradigm (e.g., positivist, constructivist/interpretivist) is also recommended	2
S6 Researcher characteristics and reflexivity	Researchers' characteristics that may influence the research, including personal attributes, qualifications/experience, relationship with participants, assumptions, or presuppositions; potential or actual interaction between researchers' characteristics and the research questions, approach, methods, results, or transferability	0
S7 Context	Setting/site and salient contextual factors; rationale[a]	2
S8 Sampling strategy	How and why research participants, documents, or events were selected; criteria for deciding when no further sampling was necessary (e.g., sampling saturation); rationale[a]	2
S9 Ethical issues pertaining to human subjects	Documentation of approval by an appropriate ethics review board and participant consent, or explanation for lack thereof; other confidentiality and data security issues	N/A
S10 Data collection methods	Types of data collected; details of data collection procedures including (as appropriate) start and stop dates of data collection and analysis, iterative process, triangulation of sources/methods,	2

	and modification of procedures in response to evolving study findings; rationale[a]	
S11 Data collection instruments and technologies	Description of instruments (e.g., interview guides, questionnaires) and devices (e.g., audio recorders) used for data collection; if/how the instrument(s) changed over the course of the study	2
S12 Units of study	Number and relevant characteristics of participants, documents, or events included in the study; level of participation (could be reported in results)	2
S13 Data processing	Methods for processing data prior to and during analysis, including transcription, data entry, data management and security, verification of data integrity, data coding, and anonymization/deidentification of excerpts	2
S14 Data analysis	Process by which inferences, themes, etc., were identified and developed, including researchers involved in data analysis; usually references a specific paradigm or approach; rationale[a]	1
S15 Techniques to enhance trustworthiness	Techniques to enhance trustworthiness and credibility of data analysis (e.g., member checking, audit trail, triangulation); rationale[a]	0
**Results/Findings**		
S16 Synthesis and interpretation	Main findings (e.g., interpretations, inferences, and themes); might include development of a theory or model, or integration with prior research or theory	2
S17 Links to empirical data	Evidence (e.g., quotes, field notes, text excerpts, photographs) to substantiate analytic findings	2
**Discussion**		
S18 Integration with prior work, implications, transferability, and contribution(s) to the field	Short summary of main findings; explanation of how findings and conclusions connect to, support, elaborate on, or challenge conclusions of earlier scholarship; discussion of scope of application/generalizability; identification of unique contribution(s) to scholarship in a discipline or field	2
S19 Limitations	Trustworthiness and limitations of findings	0
**Other**		
S20 Conflicts of interest	Potential sources of influence or perceived influence on study conduct and conclusions; how these were managed	1
S21 Funding	Sources of funding and other support; role of funders in data collection, interpretation, and reporting	2

Kirmayer, L.J.; Boothroyd, L.J.; Hodgins, S. Attempted suicide among Inuit youth: psychosocial correlates and implications for prevention. Can J Psychiatry 1998, 43, 816-822, doi:10.1177/070674379804300806.

STROBE Statement—checklist of items that should be included in reports of observational studies

	Item No	Recommendation	Recommendation met? (Y/N)
**Title and abstract**	1	(*a*) Indicate the study's design with a commonly used term in the title or the abstract	Y
		(*b*) Provide in the abstract an informative and balanced summary of what was done and what was found	Y
**Introduction**			
Background/rationale	2	Explain the scientific background and rationale for the investigation being reported	Y
Objectives	3	State specific objectives, including any prespecified hypotheses	Y
**Methods**			
Study design	4	Present key elements of study design early in the paper	Y
Setting	5	Describe the setting, locations, and relevant dates, including periods of recruitment, exposure, follow-up, and data collection	Y
Participants	6	(*a*) *Cohort study*—Give the eligibility criteria, and the sources and methods of selection of participants. Describe methods of follow-up<br>*Case-control study*—Give the eligibility criteria, and the sources and methods of case ascertainment and control selection. Give the rationale for the choice of cases and controls<br>*Cross-sectional study*—Give the eligibility criteria, and the sources and methods of selection of participants	Y
		(*b*) *Cohort study*—For matched studies, give matching criteria and number of exposed and unexposed<br>*Case-control study*—For matched studies, give matching criteria and the number of controls per case	N/A
Variables	7	Clearly define all outcomes, exposures, predictors, potential confounders, and effect modifiers. Give diagnostic criteria, if applicable	Y
Data sources/ measurement	8*	For each variable of interest, give sources of data and details of methods of assessment (measurement). Describe comparability of assessment methods if there is more than one group	Y

Bias	9	Describe any efforts to address potential sources of bias	N
Study size	10	Explain how the study size was arrived at	Y
Quantitative variables	11	Explain how quantitative variables were handled in the analyses. If applicable, describe which groupings were chosen and why	Y
Statistical methods	12	(*a*) Describe all statistical methods, including those used to control for confounding	Y
		(*b*) Describe any methods used to examine subgroups and interactions	Y
		(*c*) Explain how missing data were addressed	Y
		(*d*) *Cohort study*—If applicable, explain how loss to follow-up was addressed<br>*Case-control study*—If applicable, explain how matching of cases and controls was addressed<br>*Cross-sectional study*—If applicable, describe analytical methods taking account of sampling strategy	N/A
		(*e*) Describe any sensitivity analyses	Y
**Results**			
Participants	13*	(a) Report numbers of individuals at each stage of study—eg numbers potentially eligible, examined for eligibility, confirmed eligible, included in the study, completing follow-up, and analysed	
		(b) Give reasons for non-participation at each stage	
		(c) Consider use of a flow diagram	
Descriptive data	14*	(a) Give characteristics of study participants (eg demographic, clinical, social) and information on exposures and potential confounders	Y
		(b) Indicate number of participants with missing data for each variable of interest	N
		(c) *Cohort study*—Summarise follow-up time (eg, average and total amount)	N/A
Outcome data	15*	*Cohort study*—Report numbers of outcome events or summary measures over time	N/A
		Case-control study—Report numbers in each exposure category, or summary measures of exposure	N/A
		Cross-sectional study—Report numbers of outcome events or summary measures	*Y*
Main results	16	(*a*) Give unadjusted estimates and, if applicable, confounder-adjusted estimates and their precision (eg, 95% confidence interval). Make clear which confounders were adjusted for and why they were included	Y
		(*b*) Report category boundaries when	N/A

		continuous variables were categorized	
		(*c*) If relevant, consider translating estimates of relative risk into absolute risk for a meaningful time period	N/A
Other analyses	17	Report other analyses done—eg analyses of subgroups and interactions, and sensitivity analyses	Y
**Discussion**			
Key results	18	Summarise key results with reference to study objectives	Y
Limitations	19	Discuss limitations of the study, taking into account sources of potential bias or imprecision. Discuss both direction and magnitude of any potential bias	Y
Interpretation	20	Give a cautious overall interpretation of results considering objectives, limitations, multiplicity of analyses, results from similar studies, and other relevant evidence	Y
Generalisability	21	Discuss the generalisability (external validity) of the study results	Y
**Other information**			
Funding	22	Give the source of funding and the role of the funders for the present study and, if applicable, for the original study on which the present article is based	Y

Fortin, M.; Muckle, G.; Anassour-Laouan-Sidi, E.; Jacobson, S.W.; Jacobson, J.L.; Belanger, R.E. Trajectories of Alcohol Use and Binge Drinking Among Pregnant Inuit Women. Alcohol Alcohol 2016, 51, 339-346, doi:10.1093/alcalc/agv112.

STROBE Statement—checklist of items that should be included in reports of observational studies

	Item No	Recommendation	Recommendation met? (Y/N)
**Title and abstract**	1	(*a*) Indicate the study's design with a commonly used term in the title or the abstract	Y
		(*b*) Provide in the abstract an informative and balanced summary of what was done and what was found	Y
**Introduction**			
Background/rationale	2	Explain the scientific background and rationale for the investigation being reported	Y
Objectives	3	State specific objectives, including any prespecified hypotheses	Y
**Methods**			
Study design	4	Present key elements of study design early in the paper	Y
Setting	5	Describe the setting, locations, and relevant dates, including periods of recruitment, exposure, follow-up, and data collection	Y
Participants	6	(*a*) *Cohort study*—Give the eligibility criteria, and the sources and methods of selection of participants. Describe methods of follow-up<br>*Case-control study*—Give the eligibility criteria, and the sources and methods of case ascertainment and control selection. Give the rationale for the choice of cases and controls<br>*Cross-sectional study*—Give the eligibility criteria, and the sources and methods of selection of participants	Y
		(*b*) *Cohort study*—For matched studies, give matching criteria and number of exposed and unexposed<br>*Case-control study*—For matched studies, give matching criteria and the number of controls per case	N/A
Variables	7	Clearly define all outcomes, exposures, predictors, potential confounders, and effect modifiers. Give diagnostic criteria, if applicable	Y
Data sources/ measurement	8*	For each variable of interest, give sources of data and details of methods of assessment (measurement). Describe comparability of assessment methods if there is more than one group	Y

Bias	9	Describe any efforts to address potential sources of bias	N
Study size	10	Explain how the study size was arrived at	Y
Quantitative variables	11	Explain how quantitative variables were handled in the analyses. If applicable, describe which groupings were chosen and why	Y
Statistical methods	12	(*a*) Describe all statistical methods, including those used to control for confounding	Y
		(*b*) Describe any methods used to examine subgroups and interactions	Y
		(*c*) Explain how missing data were addressed	Y
		(*d*) *Cohort study*—If applicable, explain how loss to follow-up was addressed<br>*Case-control study*—If applicable, explain how matching of cases and controls was addressed<br>*Cross-sectional study*—If applicable, describe analytical methods taking account of sampling strategy	N/A
		(*e*) Describe any sensitivity analyses	N
**Results**			
Participants	13*	(a) Report numbers of individuals at each stage of study—eg numbers potentially eligible, examined for eligibility, confirmed eligible, included in the study, completing follow-up, and analysed	Y
		(b) Give reasons for non-participation at each stage	Y
		(c) Consider use of a flow diagram	N
Descriptive data	14*	(a) Give characteristics of study participants (eg demographic, clinical, social) and information on exposures and potential confounders	Y
		(b) Indicate number of participants with missing data for each variable of interest	Y
		(c) *Cohort study*—Summarise follow-up time (eg, average and total amount)	N/A
Outcome data	15*	*Cohort study*—Report numbers of outcome events or summary measures over time	N/A
		Case-control study—Report numbers in each exposure category, or summary measures of exposure	N/A
		Cross-sectional study—Report numbers of outcome events or summary measures	*Y*
Main results	16	(*a*) Give unadjusted estimates and, if applicable, confounder-adjusted estimates and their precision (eg, 95% confidence interval). Make clear which confounders were adjusted for and why they were included	Y
		(*b*) Report category boundaries when	N/A

		continuous variables were categorized	
		(*c*) If relevant, consider translating estimates of relative risk into absolute risk for a meaningful time period	N/A
Other analyses	17	Report other analyses done—eg analyses of subgroups and interactions, and sensitivity analyses	Y
**Discussion**			
Key results	18	Summarise key results with reference to study objectives	Y
Limitations	19	Discuss limitations of the study, taking into account sources of potential bias or imprecision. Discuss both direction and magnitude of any potential bias	Y
Interpretation	20	Give a cautious overall interpretation of results considering objectives, limitations, multiplicity of analyses, results from similar studies, and other relevant evidence	Y
Generalisability	21	Discuss the generalisability (external validity) of the study results	N
**Other information**			
Funding	22	Give the source of funding and the role of the funders for the present study and, if applicable, for the original study on which the present article is based	Y

Decaluwe, B.; Fortin, M.; Moisan, C.; Muckle, G.; Belanger, R.E. Drinking motives supporting binge drinking of Inuit adolescents. Can J Public Health 2019, 110, 414-421, doi:10.17269/s41997-019-00212-5.

STROBE Statement—checklist of items that should be included in reports of observational studies

	Item No	Recommendation	Recommendation met? (Y/N)
**Title and abstract**	1	(*a*) Indicate the study's design with a commonly used term in the title or the abstract	Y
		(*b*) Provide in the abstract an informative and balanced summary of what was done and what was found	Y
**Introduction**			
Background/rationale	2	Explain the scientific background and rationale for the investigation being reported	Y
Objectives	3	State specific objectives, including any prespecified hypotheses	Y
**Methods**			
Study design	4	Present key elements of study design early in the paper	Y
Setting	5	Describe the setting, locations, and relevant dates, including periods of recruitment, exposure, follow-up, and data collection	Y
Participants	6	(*a*) *Cohort study*—Give the eligibility criteria, and the sources and methods of selection of participants. Describe methods of follow-up<br>*Case-control study*—Give the eligibility criteria, and the sources and methods of case ascertainment and control selection. Give the rationale for the choice of cases and controls<br>*Cross-sectional study*—Give the eligibility criteria, and the sources and methods of selection of participants	Y
		(*b*) *Cohort study*—For matched studies, give matching criteria and number of exposed and unexposed<br>*Case-control study*—For matched studies, give matching criteria and the number of controls per case	N/A
Variables	7	Clearly define all outcomes, exposures, predictors, potential confounders, and effect modifiers. Give diagnostic criteria, if applicable	Y
Data sources/ measurement	8*	For each variable of interest, give sources of data and details of methods of assessment (measurement). Describe comparability of assessment methods if there is more than one group	Y

Bias	9	Describe any efforts to address potential sources of bias	N
Study size	10	Explain how the study size was arrived at	Y
Quantitative variables	11	Explain how quantitative variables were handled in the analyses. If applicable, describe which groupings were chosen and why	Y
Statistical methods	12	(*a*) Describe all statistical methods, including those used to control for confounding	Y
		(*b*) Describe any methods used to examine subgroups and interactions	Y
		(*c*) Explain how missing data were addressed	Y
		(*d*) *Cohort study*—If applicable, explain how loss to follow-up was addressed *Case-control study*—If applicable, explain how matching of cases and controls was addressed *Cross-sectional study*—If applicable, describe analytical methods taking account of sampling strategy	N/A
		(*e*) Describe any sensitivity analyses	Y
**Results**			
Participants	13*	(a) Report numbers of individuals at each stage of study—eg numbers potentially eligible, examined for eligibility, confirmed eligible, included in the study, completing follow-up, and analysed	Y
		(b) Give reasons for non-participation at each stage	N/A
		(c) Consider use of a flow diagram	N
Descriptive data	14*	(a) Give characteristics of study participants (eg demographic, clinical, social) and information on exposures and potential confounders	Y
		(b) Indicate number of participants with missing data for each variable of interest	Y
		(c) *Cohort study*—Summarise follow-up time (eg, average and total amount)	N/A
Outcome data	15*	*Cohort study*—Report numbers of outcome events or summary measures over time	N/A
		Case-control study—Report numbers in each exposure category, or summary measures of exposure	N/A
		Cross-sectional study—Report numbers of outcome events or summary measures	*Y*
Main results	16	(*a*) Give unadjusted estimates and, if applicable, confounder-adjusted estimates and their precision (eg, 95% confidence interval). Make clear which confounders were adjusted for and why they were included	*Y*
		(*b*) Report category boundaries when	N/A

		continuous variables were categorized	
		(*c*) If relevant, consider translating estimates of relative risk into absolute risk for a meaningful time period	N/A
Other analyses	17	Report other analyses done—eg analyses of subgroups and interactions, and sensitivity analyses	Y
**Discussion**			
Key results	18	Summarise key results with reference to study objectives	Y
Limitations	19	Discuss limitations of the study, taking into account sources of potential bias or imprecision. Discuss both direction and magnitude of any potential bias	Y
Interpretation	20	Give a cautious overall interpretation of results considering objectives, limitations, multiplicity of analyses, results from similar studies, and other relevant evidence	Y
Generalisability	21	Discuss the generalisability (external validity) of the study results	Y
**Other information**			
Funding	22	Give the source of funding and the role of the funders for the present study and, if applicable, for the original study on which the present article is based	Y

Haggarty, J.M.; Cernovsky, Z.; Bedard, M.; Merskey, H. Suicidality in a sample of Arctic households. Suicide Life Threat Behav 2008, 38, 699-707, doi:10.1521/suli.2008.38.6.699.

STROBE Statement—checklist of items that should be included in reports of observational studies

	Item No	Recommendation	Recommendation met? (Y/N)
**Title and abstract**	1	(*a*) Indicate the study's design with a commonly used term in the title or the abstract	Y
		(*b*) Provide in the abstract an informative and balanced summary of what was done and what was found	Y
**Introduction**			
Background/rationale	2	Explain the scientific background and rationale for the investigation being reported	Y
Objectives	3	State specific objectives, including any prespecified hypotheses	Y
**Methods**			
Study design	4	Present key elements of study design early in the paper	Y
Setting	5	Describe the setting, locations, and relevant dates, including periods of recruitment, exposure, follow-up, and data collection	Y
Participants	6	(*a*) *Cohort study*—Give the eligibility criteria, and the sources and methods of selection of participants. Describe methods of follow-up<br>*Case-control study*—Give the eligibility criteria, and the sources and methods of case ascertainment and control selection. Give the rationale for the choice of cases and controls<br>*Cross-sectional study*—Give the eligibility criteria, and the sources and methods of selection of participants	Y
		(*b*) *Cohort study*—For matched studies, give matching criteria and number of exposed and unexposed<br>*Case-control study*—For matched studies, give matching criteria and the number of controls per case	N/A
Variables	7	Clearly define all outcomes, exposures, predictors, potential confounders, and effect modifiers. Give diagnostic criteria, if applicable	Y
Data sources/ measurement	8*	For each variable of interest, give sources of data and details of methods of assessment (measurement). Describe comparability of assessment methods if there is more than one group	Y
Bias	9	Describe any efforts to address potential	N

		sources of bias	
Study size	10	Explain how the study size was arrived at	Y
Quantitative variables	11	Explain how quantitative variables were handled in the analyses. If applicable, describe which groupings were chosen and why	Y
Statistical methods	12	(*a*) Describe all statistical methods, including those used to control for confounding	Y
		(*b*) Describe any methods used to examine subgroups and interactions	Y
		(*c*) Explain how missing data were addressed	N
		(*d*) *Cohort study*—If applicable, explain how loss to follow-up was addressed<br>*Case-control study*—If applicable, explain how matching of cases and controls was addressed<br>*Cross-sectional study*—If applicable, describe analytical methods taking account of sampling strategy	N/A
		(*e*) Describe any sensitivity analyses	N
**Results**			
Participants	13*	(a) Report numbers of individuals at each stage of study—eg numbers potentially eligible, examined for eligibility, confirmed eligible, included in the study, completing follow-up, and analysed	Y
		(b) Give reasons for non-participation at each stage	N
		(c) Consider use of a flow diagram	N
Descriptive data	14*	(a) Give characteristics of study participants (eg demographic, clinical, social) and information on exposures and potential confounders	Y
		(b) Indicate number of participants with missing data for each variable of interest	Y
		(c) *Cohort study*—Summarise follow-up time (eg, average and total amount)	N/A
Outcome data	15*	*Cohort study*—Report numbers of outcome events or summary measures over time	N/A
		Case-control study—Report numbers in each exposure category, or summary measures of exposure	N/A
		Cross-sectional study—Report numbers of outcome events or summary measures	*Y*
Main results	16	(*a*) Give unadjusted estimates and, if applicable, confounder-adjusted estimates and their precision (eg, 95% confidence interval). Make clear which confounders were adjusted for and why they were included	Y
		(*b*) Report category boundaries when continuous variables were categorized	N/A

		(*c*) If relevant, consider translating estimates of relative risk into absolute risk for a meaningful time period	N/A
Other analyses	17	Report other analyses done—eg analyses of subgroups and interactions, and sensitivity analyses	Y
**Discussion**			
Key results	18	Summarise key results with reference to study objectives	Y
Limitations	19	Discuss limitations of the study, taking into account sources of potential bias or imprecision. Discuss both direction and magnitude of any potential bias	Y
Interpretation	20	Give a cautious overall interpretation of results considering objectives, limitations, multiplicity of analyses, results from similar studies, and other relevant evidence	YY
Generalisability	21	Discuss the generalisability (external validity) of the study results	Y
**Other information**			
Funding	22	Give the source of funding and the role of the funders for the present study and, if applicable, for the original study on which the present article is based	N

Wood, D.S. Alcohol controls and violence in Nunavut: a comparison of wet and dry communities. Int J Circumpolar Health 2011, 70, 19-28, doi:10.3402/ijch.v70i1.17801.

STROBE Statement—checklist of items that should be included in reports of observational studies

	Item No	Recommendation	Recommendation met? (Y/N)
**Title and abstract**	1	(*a*) Indicate the study's design with a commonly used term in the title or the abstract	Y
		(*b*) Provide in the abstract an informative and balanced summary of what was done and what was found	Y
**Introduction**			
Background/rationale	2	Explain the scientific background and rationale for the investigation being reported	Y
Objectives	3	State specific objectives, including any prespecified hypotheses	Y
**Methods**			
Study design	4	Present key elements of study design early in the paper	Y
Setting	5	Describe the setting, locations, and relevant dates, including periods of recruitment, exposure, follow-up, and data collection	Y
Participants	6	(*a*) *Cohort study*—Give the eligibility criteria, and the sources and methods of selection of participants. Describe methods of follow-up<br>*Case-control study*—Give the eligibility criteria, and the sources and methods of case ascertainment and control selection. Give the rationale for the choice of cases and controls<br>*Cross-sectional study*—Give the eligibility criteria, and the sources and methods of selection of participants	N/A
		(*b*) *Cohort study*—For matched studies, give matching criteria and number of exposed and unexposed<br>*Case-control study*—For matched studies, give matching criteria and the number of controls per case	N/A
Variables	7	Clearly define all outcomes, exposures, predictors, potential confounders, and effect modifiers. Give diagnostic criteria, if applicable	Y
Data sources/ measurement	8*	For each variable of interest, give sources of data and details of methods of assessment (measurement). Describe comparability of assessment methods if there is more than one group	Y
Bias	9	Describe any efforts to address potential	N

		sources of bias	
Study size	10	Explain how the study size was arrived at	N/A
Quantitative variables	11	Explain how quantitative variables were handled in the analyses. If applicable, describe which groupings were chosen and why	Y
Statistical methods	12	(*a*) Describe all statistical methods, including those used to control for confounding	Y
		(*b*) Describe any methods used to examine subgroups and interactions	Y
		(*c*) Explain how missing data were addressed	Y
		(*d*) *Cohort study*—If applicable, explain how loss to follow-up was addressed<br>*Case-control study*—If applicable, explain how matching of cases and controls was addressed<br>*Cross-sectional study*—If applicable, describe analytical methods taking account of sampling strategy	N/A
		(*e*) Describe any sensitivity analyses	N/A
**Results**			
Participants	13*	(a) Report numbers of individuals at each stage of study—eg numbers potentially eligible, examined for eligibility, confirmed eligible, included in the study, completing follow-up, and analysed	N/A
		(b) Give reasons for non-participation at each stage	N/A
		(c) Consider use of a flow diagram	N/A
Descriptive data	14*	(a) Give characteristics of study participants (eg demographic, clinical, social) and information on exposures and potential confounders	Y
		(b) Indicate number of participants with missing data for each variable of interest	Y
		(c) *Cohort study*—Summarise follow-up time (eg, average and total amount)	N/A
Outcome data	15*	*Cohort study*—Report numbers of outcome events or summary measures over time	N/A
		Case-control study—Report numbers in each exposure category, or summary measures of exposure	N/A
		Cross-sectional study—Report numbers of outcome events or summary measures	*Y*
Main results	16	(*a*) Give unadjusted estimates and, if applicable, confounder-adjusted estimates and their precision (eg, 95% confidence interval). Make clear which confounders were adjusted for and why they were included	Y
		(*b*) Report category boundaries when continuous variables were categorized	N/A

		(*c*) If relevant, consider translating estimates of relative risk into absolute risk for a meaningful time period	N/A
Other analyses	17	Report other analyses done—eg analyses of subgroups and interactions, and sensitivity analyses	Y
**Discussion**			
Key results	18	Summarise key results with reference to study objectives	Y
Limitations	19	Discuss limitations of the study, taking into account sources of potential bias or imprecision. Discuss both direction and magnitude of any potential bias	Y
Interpretation	20	Give a cautious overall interpretation of results considering objectives, limitations, multiplicity of analyses, results from similar studies, and other relevant evidence	Y
Generalisability	21	Discuss the generalisability (external validity) of the study results	N/A
**Other information**			
Funding	22	Give the source of funding and the role of the funders for the present study and, if applicable, for the original study on which the present article is based	N

Collins, P.Y.; Delgado, R.A., Jr.; Apok, C.; Baez, L.; Bjerregaard, P.; Chatwood, S.; Chipp, C.; Crawford, A.; Crosby, A.; Dillard, D.; et al. RISING SUN: Prioritized Outcomes for Suicide Prevention in the Arctic. Psychiatr Serv 2019, 70, 152-155, doi:10.1176/appi.ps.201700505.

Standards for Reporting Qualitative Research (SRQR)

**No. Topic**	**Item**	**0 – no 1 – maybe 2-Yes**
**Title and abstract**		
S1 Title	Concise description of the nature and topic of the study identifying the study as qualitative or indicating the approach (e.g., ethnography, grounded theory) or data collection methods (e.g., interview, focus group) is recommended	2
S2 Abstract	Summary of key elements of the study using the abstract format of the intended publication; typically includes objective, methods, results, and conclusions	2
**Introduction**		
S3 Problem formulation	Description and significance of the problem/phenomenon studied; review of relevant theory and empirical work; problem statement	2
S4 Purpose or research question	Purpose of the study and specific objectives or questions	2
**Methods**		
S5 Qualitative approach and research paradigm	Qualitative approach (e.g., ethnography, grounded theory, case study, phenomenology, narrative research) and guiding theory if appropriate; identifying the research paradigm (e.g., positivist, constructivist/interpretivist) is also recommended	0
S6 Researcher characteristics and reflexivity	Researchers' characteristics that may influence the research, including personal attributes, qualifications/experience, relationship with participants, assumptions, or presuppositions; potential or actual interaction between researchers' characteristics and the research questions, approach, methods, results, or transferability	0
S7 Context	Setting/site and salient contextual factors; rationale[a]	2
S8 Sampling strategy	How and why research participants, documents, or events were selected; criteria for deciding when no further sampling was necessary (e.g., sampling saturation); rationale[a]	2
S9 Ethical issues pertaining to human subjects	Documentation of approval by an appropriate ethics review board and participant consent, or explanation for lack thereof; other confidentiality and data security issues	0
S10 Data collection methods	Types of data collected; details of data collection procedures including (as appropriate) start and stop dates of data collection	2

	and analysis, iterative process, triangulation of sources/methods, and modification of procedures in response to evolving study findings; rationale[a]	
S11 Data collection instruments and technologies	Description of instruments (e.g., interview guides, questionnaires) and devices (e.g., audio recorders) used for data collection; if/how the instrument(s) changed over the course of the study	0
S12 Units of study	Number and relevant characteristics of participants, documents, or events included in the study; level of participation (could be reported in results)	2
S13 Data processing	Methods for processing data prior to and during analysis, including transcription, data entry, data management and security, verification of data integrity, data coding, and anonymization/deidentification of excerpts	0
S14 Data analysis	Process by which inferences, themes, etc., were identified and developed, including researchers involved in data analysis; usually references a specific paradigm or approach; rationale[a]	0
S15 Techniques to enhance trustworthiness	Techniques to enhance trustworthiness and credibility of data analysis (e.g., member checking, audit trail, triangulation); rationale[a]	1
**Results/Findings**		
S16 Synthesis and interpretation	Main findings (e.g., interpretations, inferences, and themes); might include development of a theory or model, or integration with prior research or theory	2
S17 Links to empirical data	Evidence (e.g., quotes, field notes, text excerpts, photographs) to substantiate analytic findings	2
**Discussion**		
S18 Integration with prior work, implications, transferability, and contribution(s) to the field	Short summary of main findings; explanation of how findings and conclusions connect to, support, elaborate on, or challenge conclusions of earlier scholarship; discussion of scope of application/generalizability; identification of unique contribution(s) to scholarship in a discipline or field	2
S19 Limitations	Trustworthiness and limitations of findings	0
**Other**		
S20 Conflicts of interest	Potential sources of influence or perceived influence on study conduct and conclusions; how these were managed	2
S21 Funding	Sources of funding and other support; role of funders in data collection, interpretation, and reporting	2

Petrasek MacDonald, J.; Cunsolo Willox, A.; Ford, J.D.; Shiwak, I.; Wood, M.; Team, I.; Rigolet Inuit Community, G. Protective factors for mental health and well-being in a changing climate: Perspectives from Inuit youth in Nunatsiavut, Labrador. Soc Sci Med 2015, 141, 133-141,

Standards for Reporting Qualitative Research (SRQR)

**No. Topic**	**Item**	**0 – no<br>1 – maybe<br>2-Yes**
**Title and abstract**		
S1 Title	Concise description of the nature and topic of the study identifying the study as qualitative or indicating the approach (e.g., ethnography, grounded theory) or data collection methods (e.g., interview, focus group) is recommended	2
S2 Abstract	Summary of key elements of the study using the abstract format of the intended publication; typically includes objective, methods, results, and conclusions	2
**Introduction**		
S3 Problem formulation	Description and significance of the problem/phenomenon studied; review of relevant theory and empirical work; problem statement	2
S4 Purpose or research question	Purpose of the study and specific objectives or questions	2
**Methods**		
S5 Qualitative approach and research paradigm	Qualitative approach (e.g., ethnography, grounded theory, case study, phenomenology, narrative research) and guiding theory if appropriate; identifying the research paradigm (e.g., positivist, constructivist/interpretivist) is also recommended	0
S6 Researcher characteristics and reflexivity	Researchers' characteristics that may influence the research, including personal attributes, qualifications/experience, relationship with participants, assumptions, or presuppositions; potential or actual interaction between researchers' characteristics and the research questions, approach, methods, results, or transferability	0
S7 Context	Setting/site and salient contextual factors; rationale[a]	1
S8 Sampling strategy	How and why research participants, documents, or events were selected; criteria for deciding when no further sampling was necessary (e.g., sampling saturation); rationale[a]	2
S9 Ethical issues pertaining to human subjects	Documentation of approval by an appropriate ethics review board and participant consent, or explanation for lack thereof; other confidentiality and data security issues	0
S10 Data collection methods	Types of data collected; details of data collection procedures including (as appropriate) start and stop dates of data collection	2

	and analysis, iterative process, triangulation of sources/methods, and modification of procedures in response to evolving study findings; rationale[a]	
S11 Data collection instruments and technologies	Description of instruments (e.g., interview guides, questionnaires) and devices (e.g., audio recorders) used for data collection; if/how the instrument(s) changed over the course of the study	2
S12 Units of study	Number and relevant characteristics of participants, documents, or events included in the study; level of participation (could be reported in results)	1
S13 Data processing	Methods for processing data prior to and during analysis, including transcription, data entry, data management and security, verification of data integrity, data coding, and anonymization/deidentification of excerpts	2
S14 Data analysis	Process by which inferences, themes, etc., were identified and developed, including researchers involved in data analysis; usually references a specific paradigm or approach; rationale[a]	2
S15 Techniques to enhance trustworthiness	Techniques to enhance trustworthiness and credibility of data analysis (e.g., member checking, audit trail, triangulation); rationale[a]	2
**Results/Findings**		
S16 Synthesis and interpretation	Main findings (e.g., interpretations, inferences, and themes); might include development of a theory or model, or integration with prior research or theory	2
S17 Links to empirical data	Evidence (e.g., quotes, field notes, text excerpts, photographs) to substantiate analytic findings	2
**Discussion**		
S18 Integration with prior work, implications, transferability, and contribution(s) to the field	Short summary of main findings; explanation of how findings and conclusions connect to, support, elaborate on, or challenge conclusions of earlier scholarship; discussion of scope of application/generalizability; identification of unique contribution(s) to scholarship in a discipline or field	2
S19 Limitations	Trustworthiness and limitations of findings	0
**Other**		
S20 Conflicts of interest	Potential sources of influence or perceived influence on study conduct and conclusions; how these were managed	0
S21 Funding	Sources of funding and other support; role of funders in data collection, interpretation, and reporting	2

Kral, M.J. "The weight on our shoulders is too much, and we are falling": Suicide among Inuit male youth in Nunavut, Canada. Med Anthropol Q 2013, 27, 63-83, doi:10.1111/maq.12016.

Standards for Reporting Qualitative Research (SRQR)

No. Topic	Item	0 – no<br>1 – maybe<br>2-Yes
**Title and abstract**		
S1 Title	Concise description of the nature and topic of the study identifying the study as qualitative or indicating the approach (e.g., ethnography, grounded theory) or data collection methods (e.g., interview, focus group) is recommended	2
S2 Abstract	Summary of key elements of the study using the abstract format of the intended publication; typically includes objective, methods, results, and conclusions	2
**Introduction**		
S3 Problem formulation	Description and significance of the problem/phenomenon studied; review of relevant theory and empirical work; problem statement	2
S4 Purpose or research question	Purpose of the study and specific objectives or questions	2
**Methods**		
S5 Qualitative approach and research paradigm	Qualitative approach (e.g., ethnography, grounded theory, case study, phenomenology, narrative research) and guiding theory if appropriate; identifying the research paradigm (e.g., positivist, constructivist/interpretivist) is also recommended	2
S6 Researcher characteristics and reflexivity	Researchers' characteristics that may influence the research, including personal attributes, qualifications/experience, relationship with participants, assumptions, or presuppositions; potential or actual interaction between researchers' characteristics and the research questions, approach, methods, results, or transferability	2
S7 Context	Setting/site and salient contextual factors; rationale[a]	2
S8 Sampling strategy	How and why research participants, documents, or events were selected; criteria for deciding when no further sampling was necessary (e.g., sampling saturation); rationale[a]	2
S9 Ethical issues pertaining to human subjects	Documentation of approval by an appropriate ethics review board and participant consent, or explanation for lack thereof; other confidentiality and data security issues	2
S10 Data collection methods	Types of data collected; details of data collection procedures including (as appropriate) start and stop dates of data collection and analysis, iterative process, triangulation of sources/methods,	2

	and modification of procedures in response to evolving study findings; rationale[a]	
S11 Data collection instruments and technologies	Description of instruments (e.g., interview guides, questionnaires) and devices (e.g., audio recorders) used for data collection; if/how the instrument(s) changed over the course of the study	2
S12 Units of study	Number and relevant characteristics of participants, documents, or events included in the study; level of participation (could be reported in results)	2
S13 Data processing	Methods for processing data prior to and during analysis, including transcription, data entry, data management and security, verification of data integrity, data coding, and anonymization/deidentification of excerpts	2
S14 Data analysis	Process by which inferences, themes, etc., were identified and developed, including researchers involved in data analysis; usually references a specific paradigm or approach; rationale[a]	2
S15 Techniques to enhance trustworthiness	Techniques to enhance trustworthiness and credibility of data analysis (e.g., member checking, audit trail, triangulation); rationale[a]	2
**Results/Findings**		
S16 Synthesis and interpretation	Main findings (e.g., interpretations, inferences, and themes); might include development of a theory or model, or integration with prior research or theory	2
S17 Links to empirical data	Evidence (e.g., quotes, field notes, text excerpts, photographs) to substantiate analytic findings	2
**Discussion**		
S18 Integration with prior work, implications, transferability, and contribution(s) to the field	Short summary of main findings; explanation of how findings and conclusions connect to, support, elaborate on, or challenge conclusions of earlier scholarship; discussion of scope of application/generalizability; identification of unique contribution(s) to scholarship in a discipline or field	2
S19 Limitations	Trustworthiness and limitations of findings	0
**Other**		
S20 Conflicts of interest	Potential sources of influence or perceived influence on study conduct and conclusions; how these were managed	0
S21 Funding	Sources of funding and other support; role of funders in data collection, interpretation, and reporting	0

Law S. F.; Hutton E. M. Community Psychiatry in the Canadian Arctic – Reflections From A 1-year Continuous Consultation Series in Iqaluit, Nunavut. Canadian Journal of Community Mental Health 2007, 26, 123-140, doi:10.7870/cjcmh-2007-0032.

STROBE Statement—checklist of items that should be included in reports of observational studies

	Item No	Recommendation	Recommendation met? (Y/N)
**Title and abstract**	1	(*a*) Indicate the study's design with a commonly used term in the title or the abstract	Y
		(*b*) Provide in the abstract an informative and balanced summary of what was done and what was found	Y
**Introduction**			
Background/rationale	2	Explain the scientific background and rationale for the investigation being reported	Y
Objectives	3	State specific objectives, including any prespecified hypotheses	Y
**Methods**			
Study design	4	Present key elements of study design early in the paper	Y
Setting	5	Describe the setting, locations, and relevant dates, including periods of recruitment, exposure, follow-up, and data collection	Y
Participants	6	(*a*) *Cohort study*—Give the eligibility criteria, and the sources and methods of selection of participants. Describe methods of follow-up<br>*Case-control study*—Give the eligibility criteria, and the sources and methods of case ascertainment and control selection. Give the rationale for the choice of cases and controls<br>*Cross-sectional study*—Give the eligibility criteria, and the sources and methods of selection of participants	N/A
		(*b*) *Cohort study*—For matched studies, give matching criteria and number of exposed and unexposed<br>*Case-control study*—For matched studies, give matching criteria and the number of controls per case	N/A
Variables	7	Clearly define all outcomes, exposures, predictors, potential confounders, and effect modifiers. Give diagnostic criteria, if applicable	Y
Data sources/ measurement	8*	For each variable of interest, give sources of data and details of methods of assessment (measurement). Describe comparability of assessment methods if there is more than one group	Y

Bias	9	Describe any efforts to address potential sources of bias	N
Study size	10	Explain how the study size was arrived at	Y
Quantitative variables	11	Explain how quantitative variables were handled in the analyses. If applicable, describe which groupings were chosen and why	Y
Statistical methods	12	(*a*) Describe all statistical methods, including those used to control for confounding	Y
		(*b*) Describe any methods used to examine subgroups and interactions	Y
		(*c*) Explain how missing data were addressed	N
		(*d*) *Cohort study*—If applicable, explain how loss to follow-up was addressed<br>*Case-control study*—If applicable, explain how matching of cases and controls was addressed<br>*Cross-sectional study*—If applicable, describe analytical methods taking account of sampling strategy	N/A
		(*e*) Describe any sensitivity analyses	N/A
**Results**			
Participants	13*	(a) Report numbers of individuals at each stage of study—eg numbers potentially eligible, examined for eligibility, confirmed eligible, included in the study, completing follow-up, and analysed	Y
		(b) Give reasons for non-participation at each stage	N/A
		(c) Consider use of a flow diagram	N/A
Descriptive data	14*	(a) Give characteristics of study participants (eg demographic, clinical, social) and information on exposures and potential confounders	Y
		(b) Indicate number of participants with missing data for each variable of interest	Y
		(c) *Cohort study*—Summarise follow-up time (eg, average and total amount)	N/A
Outcome data	15*	*Cohort study*—Report numbers of outcome events or summary measures over time	N/A
		Case-control study—Report numbers in each exposure category, or summary measures of exposure	N/A
		Cross-sectional study—Report numbers of outcome events or summary measures	*Y*
Main results	16	(*a*) Give unadjusted estimates and, if applicable, confounder-adjusted estimates and their precision (eg, 95% confidence interval). Make clear which confounders were adjusted for and why they were included	N/A
		(*b*) Report category boundaries when	N/A

		continuous variables were categorized	
		(*c*) If relevant, consider translating estimates of relative risk into absolute risk for a meaningful time period	N/A
Other analyses	17	Report other analyses done—eg analyses of subgroups and interactions, and sensitivity analyses	Y
**Discussion**			
Key results	18	Summarise key results with reference to study objectives	Y
Limitations	19	Discuss limitations of the study, taking into account sources of potential bias or imprecision. Discuss both direction and magnitude of any potential bias	Y
Interpretation	20	Give a cautious overall interpretation of results considering objectives, limitations, multiplicity of analyses, results from similar studies, and other relevant evidence	Y
Generalisability	21	Discuss the generalisability (external validity) of the study results	Y
**Other information**			
Funding	22	Give the source of funding and the role of the funders for the present study and, if applicable, for the original study on which the present article is based	N

O'Neill, L.; George, S.; Koehn, C.; Shepard, B. Informal and formal mental health: preliminary qualitative findings. Int J Circumpolar Health 2013, 72, doi:10.3402/ijch.v72i0.21203.

Standards for Reporting Qualitative Research (SRQR)

**No. Topic**	**Item**	**0 – no<br>1 – maybe<br>2-Yes**
**Title and abstract**		
S1 Title	Concise description of the nature and topic of the study identifying the study as qualitative or indicating the approach (e.g., ethnography, grounded theory) or data collection methods (e.g., interview, focus group) is recommended	2
S2 Abstract	Summary of key elements of the study using the abstract format of the intended publication; typically includes objective, methods, results, and conclusions	2
**Introduction**		
S3 Problem formulation	Description and significance of the problem/phenomenon studied; review of relevant theory and empirical work; problem statement	2
S4 Purpose or research question	Purpose of the study and specific objectives or questions	2
**Methods**		
S5 Qualitative approach and research paradigm	Qualitative approach (e.g., ethnography, grounded theory, case study, phenomenology, narrative research) and guiding theory if appropriate; identifying the research paradigm (e.g., positivist, constructivist/interpretivist) is also recommended	0
S6 Researcher characteristics and reflexivity	Researchers' characteristics that may influence the research, including personal attributes, qualifications/experience, relationship with participants, assumptions, or presuppositions; potential or actual interaction between researchers' characteristics and the research questions, approach, methods, results, or transferability	0
S7 Context	Setting/site and salient contextual factors; rationale[a]	2
S8 Sampling strategy	How and why research participants, documents, or events were selected; criteria for deciding when no further sampling was necessary (e.g., sampling saturation); rationale[a]	2
S9 Ethical issues pertaining to human subjects	Documentation of approval by an appropriate ethics review board and participant consent, or explanation for lack thereof; other confidentiality and data security issues	0
S10 Data collection methods	Types of data collected; details of data collection procedures including (as appropriate) start and stop dates of data collection and analysis, iterative process, triangulation of sources/methods,	2

	and modification of procedures in response to evolving study findings; rationale[a]	
S11 Data collection instruments and technologies	Description of instruments (e.g., interview guides, questionnaires) and devices (e.g., audio recorders) used for data collection; if/how the instrument(s) changed over the course of the study	2
S12 Units of study	Number and relevant characteristics of participants, documents, or events included in the study; level of participation (could be reported in results)	2
S13 Data processing	Methods for processing data prior to and during analysis, including transcription, data entry, data management and security, verification of data integrity, data coding, and anonymization/deidentification of excerpts	2
S14 Data analysis	Process by which inferences, themes, etc., were identified and developed, including researchers involved in data analysis; usually references a specific paradigm or approach; rationale[a]	2
S15 Techniques to enhance trustworthiness	Techniques to enhance trustworthiness and credibility of data analysis (e.g., member checking, audit trail, triangulation); rationale[a]	2
**Results/Findings**		
S16 Synthesis and interpretation	Main findings (e.g., interpretations, inferences, and themes); might include development of a theory or model, or integration with prior research or theory	2
S17 Links to empirical data	Evidence (e.g., quotes, field notes, text excerpts, photographs) to substantiate analytic findings	2
**Discussion**		
S18 Integration with prior work, implications, transferability, and contribution(s) to the field	Short summary of main findings; explanation of how findings and conclusions connect to, support, elaborate on, or challenge conclusions of earlier scholarship; discussion of scope of application/generalizability; identification of unique contribution(s) to scholarship in a discipline or field	2
S19 Limitations	Trustworthiness and limitations of findings	2
**Other**		
S20 Conflicts of interest	Potential sources of influence or perceived influence on study conduct and conclusions; how these were managed	2
S21 Funding	Sources of funding and other support; role of funders in data collection, interpretation, and reporting	2

Fortin, M.; Belanger, R.E.; Boucher, O.; Muckle, G. Temporal trends of alcohol and drug use among Inuit of Northern Quebec, Canada. Int J Circumpolar Health 2015, 74, 29146, doi:10.3402/ijch.v74.29146.

STROBE Statement—checklist of items that should be included in reports of observational studies

	Item No	Recommendation	Recommendation met? (Y/N)
**Title and abstract**	1	(*a*) Indicate the study's design with a commonly used term in the title or the abstract	Y
		(*b*) Provide in the abstract an informative and balanced summary of what was done and what was found	Y
**Introduction**			
Background/rationale	2	Explain the scientific background and rationale for the investigation being reported	Y
Objectives	3	State specific objectives, including any prespecified hypotheses	Y
**Methods**			
Study design	4	Present key elements of study design early in the paper	Y
Setting	5	Describe the setting, locations, and relevant dates, including periods of recruitment, exposure, follow-up, and data collection	Y
Participants	6	(*a*) *Cohort study*—Give the eligibility criteria, and the sources and methods of selection of participants. Describe methods of follow-up *Case-control study*—Give the eligibility criteria, and the sources and methods of case ascertainment and control selection. Give the rationale for the choice of cases and controls *Cross-sectional study*—Give the eligibility criteria, and the sources and methods of selection of participants	N/A
		(*b*) *Cohort study*—For matched studies, give matching criteria and number of exposed and unexposed *Case-control study*—For matched studies, give matching criteria and the number of controls per case	N/A
Variables	7	Clearly define all outcomes, exposures, predictors, potential confounders, and effect modifiers. Give diagnostic criteria, if applicable	Y
Data sources/ measurement	8*	For each variable of interest, give sources of data and details of methods of assessment (measurement). Describe comparability of assessment methods if there is more than one group	Y

Bias	9	Describe any efforts to address potential sources of bias	N
Study size	10	Explain how the study size was arrived at	Y
Quantitative variables	11	Explain how quantitative variables were handled in the analyses. If applicable, describe which groupings were chosen and why	Y
Statistical methods	12	(*a*) Describe all statistical methods, including those used to control for confounding	Y
		(*b*) Describe any methods used to examine subgroups and interactions	Y
		(*c*) Explain how missing data were addressed	N
		(*d*) *Cohort study*—If applicable, explain how loss to follow-up was addressed<br>*Case-control study*—If applicable, explain how matching of cases and controls was addressed<br>*Cross-sectional study*—If applicable, describe analytical methods taking account of sampling strategy	N/A
		(*e*) Describe any sensitivity analyses	N/A
**Results**			
Participants	13*	(a) Report numbers of individuals at each stage of study—eg numbers potentially eligible, examined for eligibility, confirmed eligible, included in the study, completing follow-up, and analysed	N/A
		(b) Give reasons for non-participation at each stage	N/A
		(c) Consider use of a flow diagram	N/A
Descriptive data	14*	(a) Give characteristics of study participants (eg demographic, clinical, social) and information on exposures and potential confounders	Y
		(b) Indicate number of participants with missing data for each variable of interest	N
		(c) *Cohort study*—Summarise follow-up time (eg, average and total amount)	N/A
Outcome data	15*	*Cohort study*—Report numbers of outcome events or summary measures over time	N/A
		Case-control study—Report numbers in each exposure category, or summary measures of exposure	N/A
		Cross-sectional study—Report numbers of outcome events or summary measures	*Y*
Main results	16	(*a*) Give unadjusted estimates and, if applicable, confounder-adjusted estimates and their precision (eg, 95% confidence interval). Make clear which confounders were adjusted for and why they were included	Y
		(*b*) Report category boundaries when	N/A

		continuous variables were categorized	
		(*c*) If relevant, consider translating estimates of relative risk into absolute risk for a meaningful time period	N/A
Other analyses	17	Report other analyses done—eg analyses of subgroups and interactions, and sensitivity analyses	Y
**Discussion**			
Key results	18	Summarise key results with reference to study objectives	Y
Limitations	19	Discuss limitations of the study, taking into account sources of potential bias or imprecision. Discuss both direction and magnitude of any potential bias	N
Interpretation	20	Give a cautious overall interpretation of results considering objectives, limitations, multiplicity of analyses, results from similar studies, and other relevant evidence	Y
Generalisability	21	Discuss the generalisability (external validity) of the study results	N
**Other information**			
Funding	22	Give the source of funding and the role of the funders for the present study and, if applicable, for the original study on which the present article is based	Y

Kirmayer, L.J.; Malus, M.; Boothroyd, L.J. Suicide attempts among Inuit youth: a community survey of prevalence and risk factors. Acta Psychiatr Scand 1996, 94, 8-17, doi:10.1111/j.1600-0447.1996.tb09818.x.

STROBE Statement—checklist of items that should be included in reports of observational studies

	Item No	Recommendation	Recommendation met? (Y/N)
**Title and abstract**	1	(*a*) Indicate the study's design with a commonly used term in the title or the abstract	Y
		(*b*) Provide in the abstract an informative and balanced summary of what was done and what was found	Y
**Introduction**			
Background/rationale	2	Explain the scientific background and rationale for the investigation being reported	Y
Objectives	3	State specific objectives, including any prespecified hypotheses	Y
**Methods**			
Study design	4	Present key elements of study design early in the paper	Y
Setting	5	Describe the setting, locations, and relevant dates, including periods of recruitment, exposure, follow-up, and data collection	Y
Participants	6	(*a*) *Cohort study*—Give the eligibility criteria, and the sources and methods of selection of participants. Describe methods of follow-up<br>*Case-control study*—Give the eligibility criteria, and the sources and methods of case ascertainment and control selection. Give the rationale for the choice of cases and controls<br>*Cross-sectional study*—Give the eligibility criteria, and the sources and methods of selection of participants	Y
		(*b*) *Cohort study*—For matched studies, give matching criteria and number of exposed and unexposed<br>*Case-control study*—For matched studies, give matching criteria and the number of controls per case	N/A
Variables	7	Clearly define all outcomes, exposures, predictors, potential confounders, and effect modifiers. Give diagnostic criteria, if applicable	Y
Data sources/ measurement	8*	For each variable of interest, give sources of data and details of methods of assessment (measurement). Describe comparability of assessment methods if there is more than one group	Y

Bias	9	Describe any efforts to address potential sources of bias	N
Study size	10	Explain how the study size was arrived at	Y
Quantitative variables	11	Explain how quantitative variables were handled in the analyses. If applicable, describe which groupings were chosen and why	Y
Statistical methods	12	(*a*) Describe all statistical methods, including those used to control for confounding	Y
		(*b*) Describe any methods used to examine subgroups and interactions	Y
		(*c*) Explain how missing data were addressed	Y
		(*d*) *Cohort study*—If applicable, explain how loss to follow-up was addressed<br>*Case-control study*—If applicable, explain how matching of cases and controls was addressed<br>*Cross-sectional study*—If applicable, describe analytical methods taking account of sampling strategy	N
		(*e*) Describe any sensitivity analyses	Y
**Results**			
Participants	13*	(a) Report numbers of individuals at each stage of study—eg numbers potentially eligible, examined for eligibility, confirmed eligible, included in the study, completing follow-up, and analysed	Y
		(b) Give reasons for non-participation at each stage	Y
		(c) Consider use of a flow diagram	N
Descriptive data	14*	(a) Give characteristics of study participants (eg demographic, clinical, social) and information on exposures and potential confounders	Y
		(b) Indicate number of participants with missing data for each variable of interest	Y
		(c) *Cohort study*—Summarise follow-up time (eg, average and total amount)	N/A
Outcome data	15*	*Cohort study*—Report numbers of outcome events or summary measures over time	N/A
		Case-control study—Report numbers in each exposure category, or summary measures of exposure	N/A
		Cross-sectional study—Report numbers of outcome events or summary measures	*Y*
Main results	16	(*a*) Give unadjusted estimates and, if applicable, confounder-adjusted estimates and their precision (eg, 95% confidence interval). Make clear which confounders were adjusted for and why they were included	Y
		(*b*) Report category boundaries when	N/A

		continuous variables were categorized	
		(*c*) If relevant, consider translating estimates of relative risk into absolute risk for a meaningful time period	N/A
Other analyses	17	Report other analyses done—eg analyses of subgroups and interactions, and sensitivity analyses	Y
**Discussion**			
Key results	18	Summarise key results with reference to study objectives	Y
Limitations	19	Discuss limitations of the study, taking into account sources of potential bias or imprecision. Discuss both direction and magnitude of any potential bias	N
Interpretation	20	Give a cautious overall interpretation of results considering objectives, limitations, multiplicity of analyses, results from similar studies, and other relevant evidence	Y
Generalisability	21	Discuss the generalisability (external validity) of the study results	N
**Other information**			
Funding	22	Give the source of funding and the role of the funders for the present study and, if applicable, for the original study on which the present article is based	N

Pollock, N.J.; Mulay, S.; Valcour, J.; Jong, M. Suicide Rates in Aboriginal Communities in Labrador, Canada. Am J Public Health 2016, 106, 1309-1315, doi:10.2105/AJPH.2016.303151.

STROBE Statement—checklist of items that should be included in reports of observational studies

	Item No	Recommendation	Recommendation met? (Y/N)
**Title and abstract**	1	(*a*) Indicate the study's design with a commonly used term in the title or the abstract	Y
		(*b*) Provide in the abstract an informative and balanced summary of what was done and what was found	Y
**Introduction**			
Background/rationale	2	Explain the scientific background and rationale for the investigation being reported	Y
Objectives	3	State specific objectives, including any prespecified hypotheses	Y
**Methods**			
Study design	4	Present key elements of study design early in the paper	Y
Setting	5	Describe the setting, locations, and relevant dates, including periods of recruitment, exposure, follow-up, and data collection	Y
Participants	6	(*a*) *Cohort study*—Give the eligibility criteria, and the sources and methods of selection of participants. Describe methods of follow-up<br>*Case-control study*—Give the eligibility criteria, and the sources and methods of case ascertainment and control selection. Give the rationale for the choice of cases and controls<br>*Cross-sectional study*—Give the eligibility criteria, and the sources and methods of selection of participants	N/A
		(*b*) *Cohort study*—For matched studies, give matching criteria and number of exposed and unexposed<br>*Case-control study*—For matched studies, give matching criteria and the number of controls per case	N/A
Variables	7	Clearly define all outcomes, exposures, predictors, potential confounders, and effect modifiers. Give diagnostic criteria, if applicable	Y
Data sources/ measurement	8*	For each variable of interest, give sources of data and details of methods of assessment (measurement). Describe comparability of assessment methods if there is more than one group	Y
Bias	9	Describe any efforts to address potential	N

		sources of bias	
Study size	10	Explain how the study size was arrived at	N/A
Quantitative variables	11	Explain how quantitative variables were handled in the analyses. If applicable, describe which groupings were chosen and why	Y
Statistical methods	12	(*a*) Describe all statistical methods, including those used to control for confounding	Y
		(*b*) Describe any methods used to examine subgroups and interactions	Y
		(*c*) Explain how missing data were addressed	N
		(*d*) *Cohort study*—If applicable, explain how loss to follow-up was addressed<br>*Case-control study*—If applicable, explain how matching of cases and controls was addressed<br>*Cross-sectional study*—If applicable, describe analytical methods taking account of sampling strategy	N/A
		(*e*) Describe any sensitivity analyses	N/A
**Results**			
Participants	13*	(a) Report numbers of individuals at each stage of study—eg numbers potentially eligible, examined for eligibility, confirmed eligible, included in the study, completing follow-up, and analysed	N/A
		(b) Give reasons for non-participation at each stage	N/A
		(c) Consider use of a flow diagram	N/A
Descriptive data	14*	(a) Give characteristics of study participants (eg demographic, clinical, social) and information on exposures and potential confounders	Y
		(b) Indicate number of participants with missing data for each variable of interest	N/A
		(c) *Cohort study*—Summarise follow-up time (eg, average and total amount)	N/A
Outcome data	15*	*Cohort study*—Report numbers of outcome events or summary measures over time	N/A
		Case-control study—Report numbers in each exposure category, or summary measures of exposure	N/A
		Cross-sectional study—Report numbers of outcome events or summary measures	*Y*
Main results	16	(*a*) Give unadjusted estimates and, if applicable, confounder-adjusted estimates and their precision (eg, 95% confidence interval). Make clear which confounders were adjusted for and why they were included	Y
		(*b*) Report category boundaries when continuous variables were categorized	N/A

		(*c*) If relevant, consider translating estimates of relative risk into absolute risk for a meaningful time period	N/A
Other analyses	17	Report other analyses done—eg analyses of subgroups and interactions, and sensitivity analyses	N/A
**Discussion**			
Key results	18	Summarise key results with reference to study objectives	Y
Limitations	19	Discuss limitations of the study, taking into account sources of potential bias or imprecision. Discuss both direction and magnitude of any potential bias	Y
Interpretation	20	Give a cautious overall interpretation of results considering objectives, limitations, multiplicity of analyses, results from similar studies, and other relevant evidence	Y
Generalisability	21	Discuss the generalisability (external validity) of the study results	N/A
**Other information**			
Funding	22	Give the source of funding and the role of the funders for the present study and, if applicable, for the original study on which the present article is based	N/A

Tan, J.C.; Maranzan, K.A.; Boone, M.; Vander Velde, J.; Levy, S. Caller characteristics, call contents, and types of assistance provided by caller sex and age group in a Canadian Inuit crisis line in Nunavut, 1991-2001. Suicide Life Threat Behav 2012, 42, 210-216, doi:10.1111/j.1943-278X.2012.00083.x.

Mixed Methods Appraisal Tool (MMAT), version 2018

Category of study designs	Methodological quality criteria	Responses			
		Yes	No	Can't tell	Comments
Screening questions (for all types)	S1. Are there clear research questions?	X			
	S2. Do the collected data allow to address the research questions?	X			
	Further appraisal may not be feasible or appropriate when the answer is 'No' or 'Can't tell' to one or both screening questions.				
1. Qualitative	1.1. Is the qualitative approach appropriate to answer the research question?	X			
	1.2. Are the qualitative data collection methods adequate to address the research question?	X			
	1.3. Are the findings adequately derived from the data?	X			
	1.4. Is the interpretation of results sufficiently substantiated by data?	X			
	1.5. Is there coherence between qualitative data sources, collection, analysis and interpretation?	X			
2. Quantitative randomized controlled trials	2.1. Is randomization appropriately performed?				N/A
	2.2. Are the groups comparable at baseline?				N/A
	2.3. Are there complete outcome data?				N/A
	2.4. Are outcome assessors blinded to the intervention provided?				N/A
	2.5 Did the participants adhere to the assigned intervention?				N/A
3. Quantitative non-randomized	3.1. Are the participants representative of the target population?				N/A
	3.2. Are measurements appropriate regarding both the outcome and intervention (or exposure)?				N/A
	3.3. Are there complete outcome data?				N/A
	3.4. Are the confounders accounted for in the design and analysis?				N/A
	3.5. During the study period, is the intervention administered (or exposure occurred) as intended?				N/A
4. Quantitative descriptive	4.1. Is the sampling strategy relevant to address the research question?	X			
	4.2. Is the sample representative of the target population?	X			
	4.3. Are the measurements appropriate?	X			
	4.4. Is the risk of nonresponse bias low?			X	

	4.5. Is the statistical analysis appropriate to answer the research question?	X			
5. Mixed methods	5.1. Is there an adequate rationale for using a mixed methods design to address the research question?	X			
	5.2. Are the different components of the study effectively integrated to answer the research question?	X			
	5.3. Are the outputs of the integration of qualitative and quantitative components adequately interpreted?	X			
	5.4. Are divergences and inconsistencies between quantitative and qualitative results adequately addressed?	X			
	5.5. Do the different components of the study adhere to the quality criteria of each tradition of the methods involved?	X			

Logie, C.H.; Lys, C.L.; Sokolovic, N.; Mackay, K.I.; Donkers, H.; Kanbari, A.; Pooyak, S.; Loppie, C. Contextual factors associated with depression among Northern and Indigenous adolescents in the Northwest Territories, Canada. Glob Ment Health (Camb) 2021, 8, e22, doi:10.1017/gmh.2021.21.

STROBE Statement—checklist of items that should be included in reports of observational studies

	Item No	Recommendation	Recommendation met? (Y/N)
**Title and abstract**	1	(*a*) Indicate the study's design with a commonly used term in the title or the abstract	Y
		(*b*) Provide in the abstract an informative and balanced summary of what was done and what was found	Y
**Introduction**			
Background/rationale	2	Explain the scientific background and rationale for the investigation being reported	Y
Objectives	3	State specific objectives, including any prespecified hypotheses	Y
**Methods**			
Study design	4	Present key elements of study design early in the paper	Y
Setting	5	Describe the setting, locations, and relevant dates, including periods of recruitment, exposure, follow-up, and data collection	Y
Participants	6	(*a*) *Cohort study*—Give the eligibility criteria, and the sources and methods of selection of participants. Describe methods of follow-up<br>*Case-control study*—Give the eligibility criteria, and the sources and methods of case ascertainment and control selection. Give the rationale for the choice of cases and controls<br>*Cross-sectional study*—Give the eligibility criteria, and the sources and methods of selection of participants	Y
		(*b*) *Cohort study*—For matched studies, give matching criteria and number of exposed and unexposed<br>*Case-control study*—For matched studies, give matching criteria and the number of controls per case	
Variables	7	Clearly define all outcomes, exposures, predictors, potential confounders, and effect modifiers. Give diagnostic criteria, if applicable	Y
Data sources/ measurement	8*	For each variable of interest, give sources of data and details of methods of assessment (measurement). Describe comparability of assessment methods if there is more than one group	Y

Bias	9	Describe any efforts to address potential sources of bias	N
Study size	10	Explain how the study size was arrived at	N
Quantitative variables	11	Explain how quantitative variables were handled in the analyses. If applicable, describe which groupings were chosen and why	Y
Statistical methods	12	(*a*) Describe all statistical methods, including those used to control for confounding	Y
		(*b*) Describe any methods used to examine subgroups and interactions	N/A
		(*c*) Explain how missing data were addressed	Y
		(*d*) *Cohort study*—If applicable, explain how loss to follow-up was addressed<br>*Case-control study*—If applicable, explain how matching of cases and controls was addressed<br>*Cross-sectional study*—If applicable, describe analytical methods taking account of sampling strategy	
		(*e*) Describe any sensitivity analyses	
**Results**			
Participants	13*	(a) Report numbers of individuals at each stage of study—eg numbers potentially eligible, examined for eligibility, confirmed eligible, included in the study, completing follow-up, and analysed	Y
		(b) Give reasons for non-participation at each stage	N/A
		(c) Consider use of a flow diagram	N/A
Descriptive data	14*	(a) Give characteristics of study participants (eg demographic, clinical, social) and information on exposures and potential confounders	Y
		(b) Indicate number of participants with missing data for each variable of interest	Y
		(c) *Cohort study*—Summarise follow-up time (eg, average and total amount)	
Outcome data	15*	*Cohort study*—Report numbers of outcome events or summary measures over time	
		Case-control study—Report numbers in each exposure category, or summary measures of exposure	
		Cross-sectional study—Report numbers of outcome events or summary measures	*Y*
Main results	16	(*a*) Give unadjusted estimates and, if applicable, confounder-adjusted estimates and their precision (eg, 95% confidence interval). Make clear which confounders were adjusted for and why they were included	Y
		(*b*) Report category boundaries when	

		continuous variables were categorized	
		(*c*) If relevant, consider translating estimates of relative risk into absolute risk for a meaningful time period	
Other analyses	17	Report other analyses done—eg analyses of subgroups and interactions, and sensitivity analyses	N/A
**Discussion**			
Key results	18	Summarise key results with reference to study objectives	Y
Limitations	19	Discuss limitations of the study, taking into account sources of potential bias or imprecision. Discuss both direction and magnitude of any potential bias	Y
Interpretation	20	Give a cautious overall interpretation of results considering objectives, limitations, multiplicity of analyses, results from similar studies, and other relevant evidence	Y
Generalisability	21	Discuss the generalisability (external validity) of the study results	Y
**Other information**			
Funding	22	Give the source of funding and the role of the funders for the present study and, if applicable, for the original study on which the present article is based	Y

Haggarty, J.M.; Cernovsky, Z.; Husni, M.; Minor, K.; Kermeen, P.; Merskey, H. Seasonal affective disorder in an Arctic community. Acta Psychiatr Scand 2002, 105, 378-384, doi:10.1034/j.1600-0447.2002.1o185.x.

STROBE Statement—checklist of items that should be included in reports of observational studies

	Item No	Recommendation	Recommendation met? (Y/N)
**Title and abstract**	1	(*a*) Indicate the study's design with a commonly used term in the title or the abstract	Y
		(*b*) Provide in the abstract an informative and balanced summary of what was done and what was found	Y
**Introduction**			
Background/rationale	2	Explain the scientific background and rationale for the investigation being reported	Y
Objectives	3	State specific objectives, including any prespecified hypotheses	Y
**Methods**			
Study design	4	Present key elements of study design early in the paper	Y
Setting	5	Describe the setting, locations, and relevant dates, including periods of recruitment, exposure, follow-up, and data collection	Y
Participants	6	(*a*) *Cohort study*—Give the eligibility criteria, and the sources and methods of selection of participants. Describe methods of follow-up<br>*Case-control study*—Give the eligibility criteria, and the sources and methods of case ascertainment and control selection. Give the rationale for the choice of cases and controls<br>*Cross-sectional study*—Give the eligibility criteria, and the sources and methods of selection of participants	Y
		(*b*) *Cohort study*—For matched studies, give matching criteria and number of exposed and unexposed<br>*Case-control study*—For matched studies, give matching criteria and the number of controls per case	N/A
Variables	7	Clearly define all outcomes, exposures, predictors, potential confounders, and effect modifiers. Give diagnostic criteria, if applicable	Y
Data sources/ measurement	8*	For each variable of interest, give sources of data and details of methods of assessment (measurement). Describe comparability of assessment methods if there is more than one	Y

		group	
Bias	9	Describe any efforts to address potential sources of bias	N
Study size	10	Explain how the study size was arrived at	Y
Quantitative variables	11	Explain how quantitative variables were handled in the analyses. If applicable, describe which groupings were chosen and why	Y
Statistical methods	12	(*a*) Describe all statistical methods, including those used to control for confounding	Y
		(*b*) Describe any methods used to examine subgroups and interactions	Y
		(*c*) Explain how missing data were addressed	Y
		(*d*) *Cohort study*—If applicable, explain how loss to follow-up was addressed<br>*Case-control study*—If applicable, explain how matching of cases and controls was addressed<br>*Cross-sectional study*—If applicable, describe analytical methods taking account of sampling strategy	N/A
		(*e*) Describe any sensitivity analyses	N/A
**Results**			
Participants	13*	(a) Report numbers of individuals at each stage of study—eg numbers potentially eligible, examined for eligibility, confirmed eligible, included in the study, completing follow-up, and analysed	Y
		(b) Give reasons for non-participation at each stage	Y
		(c) Consider use of a flow diagram	N
Descriptive data	14*	(a) Give characteristics of study participants (eg demographic, clinical, social) and information on exposures and potential confounders	Y
		(b) Indicate number of participants with missing data for each variable of interest	Y
		(c) *Cohort study*—Summarise follow-up time (eg, average and total amount)	N/A
Outcome data	15*	*Cohort study*—Report numbers of outcome events or summary measures over time	N/A
		Case-control study—Report numbers in each exposure category, or summary measures of exposure	N/A
		Cross-sectional study—Report numbers of outcome events or summary measures	*Y*
Main results	16	(*a*) Give unadjusted estimates and, if applicable, confounder-adjusted estimates and their precision (eg, 95% confidence interval). Make clear which confounders were adjusted for and why they were included	Y

		(*b*) Report category boundaries when continuous variables were categorized	N/A
		(*c*) If relevant, consider translating estimates of relative risk into absolute risk for a meaningful time period	N/A
Other analyses	17	Report other analyses done—eg analyses of subgroups and interactions, and sensitivity analyses	Y
**Discussion**			
Key results	18	Summarise key results with reference to study objectives	Y
Limitations	19	Discuss limitations of the study, taking into account sources of potential bias or imprecision. Discuss both direction and magnitude of any potential bias	N
Interpretation	20	Give a cautious overall interpretation of results considering objectives, limitations, multiplicity of analyses, results from similar studies, and other relevant evidence	Y
Generalisability	21	Discuss the generalisability (external validity) of the study results	N
**Other information**			
Funding	22	Give the source of funding and the role of the funders for the present study and, if applicable, for the original study on which the present article is based	N

Middleton, J.; Cunsolo, A.; Pollock, N.; Jones-Bitton, A.; Wood, M.; Shiwak, I.; Flowers, C.; Harper, S.L. Temperature and place associations with Inuit mental health in the context of climate change. Environ Res 2021, 198, 111166, doi:10.1016/j.envres.2021.111166.

STROBE Statement—checklist of items that should be included in reports of observational studies

	**Item No**	**Recommendation**	**Recommendation met? (Y/N)**
**Title and abstract**	1	(*a*) Indicate the study's design with a commonly used term in the title or the abstract	Y
		(*b*) Provide in the abstract an informative and balanced summary of what was done and what was found	Y
**Introduction**			
Background/rationale	2	Explain the scientific background and rationale for the investigation being reported	Y
Objectives	3	State specific objectives, including any prespecified hypotheses	Y
**Methods**			
Study design	4	Present key elements of study design early in the paper	Y
Setting	5	Describe the setting, locations, and relevant dates, including periods of recruitment, exposure, follow-up, and data collection	Y
Participants	6	(*a*) *Cohort study*—Give the eligibility criteria, and the sources and methods of selection of participants. Describe methods of follow-up<br>*Case-control study*—Give the eligibility criteria, and the sources and methods of case ascertainment and control selection. Give the rationale for the choice of cases and controls<br>*Cross-sectional study*—Give the eligibility criteria, and the sources and methods of selection of participants	N/A
		(*b*) *Cohort study*—For matched studies, give matching criteria and number of exposed and unexposed<br>*Case-control study*—For matched studies, give matching criteria and the number of controls per case	
Variables	7	Clearly define all outcomes, exposures, predictors, potential confounders, and effect modifiers. Give diagnostic criteria, if applicable	Y
Data sources/ measurement	8*	For each variable of interest, give sources of data and details of methods of assessment (measurement). Describe comparability of assessment methods if there is more than one group	Y

Bias	9	Describe any efforts to address potential sources of bias	N/A
Study size	10	Explain how the study size was arrived at	N/A
Quantitative variables	11	Explain how quantitative variables were handled in the analyses. If applicable, describe which groupings were chosen and why	Y
Statistical methods	12	(*a*) Describe all statistical methods, including those used to control for confounding	Y
		(*b*) Describe any methods used to examine subgroups and interactions	Y
		(*c*) Explain how missing data were addressed	
		(*d*) *Cohort study*—If applicable, explain how loss to follow-up was addressed *Case-control study*—If applicable, explain how matching of cases and controls was addressed *Cross-sectional study*—If applicable, describe analytical methods taking account of sampling strategy	
		(*e*) Describe any sensitivity analyses	Y
**Results**			
Participants	13*	(a) Report numbers of individuals at each stage of study—eg numbers potentially eligible, examined for eligibility, confirmed eligible, included in the study, completing follow-up, and analysed	N/A
		(b) Give reasons for non-participation at each stage	N/A
		(c) Consider use of a flow diagram	N/A
Descriptive data	14*	(a) Give characteristics of study participants (eg demographic, clinical, social) and information on exposures and potential confounders	N/A
		(b) Indicate number of participants with missing data for each variable of interest	N/A
		(c) *Cohort study*—Summarise follow-up time (eg, average and total amount)	
Outcome data	15*	*Cohort study*—Report numbers of outcome events or summary measures over time	
		Case-control study—Report numbers in each exposure category, or summary measures of exposure	
		Cross-sectional study—Report numbers of outcome events or summary measures	*Y*
Main results	16	(*a*) Give unadjusted estimates and, if applicable, confounder-adjusted estimates and their precision (eg, 95% confidence interval). Make clear which confounders were adjusted for and why they were included	Y
		(*b*) Report category boundaries when	

		continuous variables were categorized	
		(*c*) If relevant, consider translating estimates of relative risk into absolute risk for a meaningful time period	
Other analyses	17	Report other analyses done—eg analyses of subgroups and interactions, and sensitivity analyses	Y
**Discussion**			
Key results	18	Summarise key results with reference to study objectives	Y
Limitations	19	Discuss limitations of the study, taking into account sources of potential bias or imprecision. Discuss both direction and magnitude of any potential bias	Y
Interpretation	20	Give a cautious overall interpretation of results considering objectives, limitations, multiplicity of analyses, results from similar studies, and other relevant evidence	Y
Generalisability	21	Discuss the generalisability (external validity) of the study results	Y
**Other information**			
Funding	22	Give the source of funding and the role of the funders for the present study and, if applicable, for the original study on which the present article is based	Y

Galloway, T.; Johnson-Down, L.; Egeland, G.M. Socioeconomic and Cultural Correlates of Diet Quality in the Canadian Arctic: Results from the 2007-2008 Inuit Health Survey. Can J Diet Pract Res 2015, 76, 117-125, doi:10.3148/cjdpr-2015-006.

STROBE Statement—checklist of items that should be included in reports of observational studies

	Item No	Recommendation	Recommendation met? (Y/N)
**Title and abstract**	1	(*a*) Indicate the study's design with a commonly used term in the title or the abstract	Y
		(*b*) Provide in the abstract an informative and balanced summary of what was done and what was found	Y
**Introduction**			
Background/rationale	2	Explain the scientific background and rationale for the investigation being reported	Y
Objectives	3	State specific objectives, including any prespecified hypotheses	Y
**Methods**			
Study design	4	Present key elements of study design early in the paper	Y
Setting	5	Describe the setting, locations, and relevant dates, including periods of recruitment, exposure, follow-up, and data collection	Y
Participants	6	(*a*) *Cohort study*—Give the eligibility criteria, and the sources and methods of selection of participants. Describe methods of follow-up *Case-control study*—Give the eligibility criteria, and the sources and methods of case ascertainment and control selection. Give the rationale for the choice of cases and controls *Cross-sectional study*—Give the eligibility criteria, and the sources and methods of selection of participants	N/A
		(*b*) *Cohort study*—For matched studies, give matching criteria and number of exposed and unexposed *Case-control study*—For matched studies, give matching criteria and the number of controls per case	N/A
Variables	7	Clearly define all outcomes, exposures, predictors, potential confounders, and effect modifiers. Give diagnostic criteria, if applicable	Y
Data sources/ measurement	8*	For each variable of interest, give sources of data and details of methods of assessment (measurement). Describe comparability of assessment methods if there is more than one group	Y

Bias	9	Describe any efforts to address potential sources of bias	N
Study size	10	Explain how the study size was arrived at	Y
Quantitative variables	11	Explain how quantitative variables were handled in the analyses. If applicable, describe which groupings were chosen and why	Y
Statistical methods	12	(*a*) Describe all statistical methods, including those used to control for confounding	Y
		(*b*) Describe any methods used to examine subgroups and interactions	Y
		(*c*) Explain how missing data were addressed	N
		(*d*) *Cohort study*—If applicable, explain how loss to follow-up was addressed<br>*Case-control study*—If applicable, explain how matching of cases and controls was addressed<br>*Cross-sectional study*—If applicable, describe analytical methods taking account of sampling strategy	N/A
		(*e*) Describe any sensitivity analyses	Y
**Results**			
Participants	13*	(a) Report numbers of individuals at each stage of study—eg numbers potentially eligible, examined for eligibility, confirmed eligible, included in the study, completing follow-up, and analysed	N/A
		(b) Give reasons for non-participation at each stage	N/A
		(c) Consider use of a flow diagram	N/A
Descriptive data	14*	(a) Give characteristics of study participants (eg demographic, clinical, social) and information on exposures and potential confounders	Y
		(b) Indicate number of participants with missing data for each variable of interest	Y
		(c) *Cohort study*—Summarise follow-up time (eg, average and total amount)	N/A
Outcome data	15*	*Cohort study*—Report numbers of outcome events or summary measures over time	N/A
		Case-control study—Report numbers in each exposure category, or summary measures of exposure	N/A
		Cross-sectional study—Report numbers of outcome events or summary measures	*Y*
Main results	16	(*a*) Give unadjusted estimates and, if applicable, confounder-adjusted estimates and their precision (eg, 95% confidence interval). Make clear which confounders were adjusted for and why they were included	Y
		(*b*) Report category boundaries when	N/A

		continuous variables were categorized	
		(*c*) If relevant, consider translating estimates of relative risk into absolute risk for a meaningful time period	N/A
Other analyses	17	Report other analyses done—eg analyses of subgroups and interactions, and sensitivity analyses	Y
**Discussion**			
Key results	18	Summarise key results with reference to study objectives	Y
Limitations	19	Discuss limitations of the study, taking into account sources of potential bias or imprecision. Discuss both direction and magnitude of any potential bias	Y
Interpretation	20	Give a cautious overall interpretation of results considering objectives, limitations, multiplicity of analyses, results from similar studies, and other relevant evidence	Y
Generalisability	21	Discuss the generalisability (external validity) of the study results	Y
**Other information**			
Funding	22	Give the source of funding and the role of the funders for the present study and, if applicable, for the original study on which the present article is based	Y

Zienczuk, N.; Egeland, G.M. Association between socioeconomic status and overweight and obesity among Inuit adults: International Polar Year Inuit Health Survey, 2007-2008. Int J Circumpolar Health 2012, 71, 1-7, doi:10.3402/ijch.v71i0.18419.

STROBE Statement—checklist of items that should be included in reports of observational studies

	Item No	Recommendation	Recommendation met? (Y/N)
**Title and abstract**	1	(*a*) Indicate the study's design with a commonly used term in the title or the abstract	Y
		(*b*) Provide in the abstract an informative and balanced summary of what was done and what was found	Y
**Introduction**			
Background/rationale	2	Explain the scientific background and rationale for the investigation being reported	Y
Objectives	3	State specific objectives, including any prespecified hypotheses	Y
**Methods**			
Study design	4	Present key elements of study design early in the paper	Y
Setting	5	Describe the setting, locations, and relevant dates, including periods of recruitment, exposure, follow-up, and data collection	Y
Participants	6	(*a*) *Cohort study*—Give the eligibility criteria, and the sources and methods of selection of participants. Describe methods of follow-up<br>*Case-control study*—Give the eligibility criteria, and the sources and methods of case ascertainment and control selection. Give the rationale for the choice of cases and controls<br>*Cross-sectional study*—Give the eligibility criteria, and the sources and methods of selection of participants	Y
		(*b*) *Cohort study*—For matched studies, give matching criteria and number of exposed and unexposed<br>*Case-control study*—For matched studies, give matching criteria and the number of controls per case	N/A
Variables	7	Clearly define all outcomes, exposures, predictors, potential confounders, and effect modifiers. Give diagnostic criteria, if applicable	Y
Data sources/ measurement	8*	For each variable of interest, give sources of data and details of methods of assessment (measurement). Describe comparability of assessment methods if there is more than one group	Y

Bias	9	Describe any efforts to address potential sources of bias	N
Study size	10	Explain how the study size was arrived at	Y
Quantitative variables	11	Explain how quantitative variables were handled in the analyses. If applicable, describe which groupings were chosen and why	Y
Statistical methods	12	(*a*) Describe all statistical methods, including those used to control for confounding	Y
		(*b*) Describe any methods used to examine subgroups and interactions	Y
		(*c*) Explain how missing data were addressed	N
		(*d*) *Cohort study*—If applicable, explain how loss to follow-up was addressed<br>*Case-control study*—If applicable, explain how matching of cases and controls was addressed<br>*Cross-sectional study*—If applicable, describe analytical methods taking account of sampling strategy	N/A
		(*e*) Describe any sensitivity analyses	N
**Results**			
Participants	13*	(a) Report numbers of individuals at each stage of study—eg numbers potentially eligible, examined for eligibility, confirmed eligible, included in the study, completing follow-up, and analysed	Y
		(b) Give reasons for non-participation at each stage	N/A
		(c) Consider use of a flow diagram	N
Descriptive data	14*	(a) Give characteristics of study participants (eg demographic, clinical, social) and information on exposures and potential confounders	Y
		(b) Indicate number of participants with missing data for each variable of interest	Y
		(c) *Cohort study*—Summarise follow-up time (eg, average and total amount)	N/A
Outcome data	15*	*Cohort study*—Report numbers of outcome events or summary measures over time	N/A
		Case-control study—Report numbers in each exposure category, or summary measures of exposure	N/A
		Cross-sectional study—Report numbers of outcome events or summary measures	*Y*
Main results	16	(*a*) Give unadjusted estimates and, if applicable, confounder-adjusted estimates and their precision (eg, 95% confidence interval). Make clear which confounders were adjusted for and why they were included	Y
		(*b*) Report category boundaries when	N/A

		continuous variables were categorized	
		(*c*) If relevant, consider translating estimates of relative risk into absolute risk for a meaningful time period	N/A
Other analyses	17	Report other analyses done—eg analyses of subgroups and interactions, and sensitivity analyses	Y
**Discussion**			
Key results	18	Summarise key results with reference to study objectives	Y
Limitations	19	Discuss limitations of the study, taking into account sources of potential bias or imprecision. Discuss both direction and magnitude of any potential bias	Y
Interpretation	20	Give a cautious overall interpretation of results considering objectives, limitations, multiplicity of analyses, results from similar studies, and other relevant evidence	Y
Generalisability	21	Discuss the generalisability (external validity) of the study results	N
**Other information**			
Funding	22	Give the source of funding and the role of the funders for the present study and, if applicable, for the original study on which the present article is based	Y

Basham, C.A.; Karim, M.E. Multimorbidity prevalence in Canada: a comparison of Northern Territories with Provinces, 2013/14. Int J Circumpolar Health 2019, 78, 1607703, doi:10.1080/22423982.2019.1607703.

STROBE Statement—checklist of items that should be included in reports of observational studies

	Item No	Recommendation	Recommendation met? (Y/N)
**Title and abstract**	1	(*a*) Indicate the study's design with a commonly used term in the title or the abstract	Y
		(*b*) Provide in the abstract an informative and balanced summary of what was done and what was found	Y
**Introduction**			
Background/rationale	2	Explain the scientific background and rationale for the investigation being reported	Y
Objectives	3	State specific objectives, including any prespecified hypotheses	Y
**Methods**			
Study design	4	Present key elements of study design early in the paper	Y
Setting	5	Describe the setting, locations, and relevant dates, including periods of recruitment, exposure, follow-up, and data collection	Y
Participants	6	(*a*) *Cohort study*—Give the eligibility criteria, and the sources and methods of selection of participants. Describe methods of follow-up *Case-control study*—Give the eligibility criteria, and the sources and methods of case ascertainment and control selection. Give the rationale for the choice of cases and controls *Cross-sectional study*—Give the eligibility criteria, and the sources and methods of selection of participants	N/A
		(*b*) *Cohort study*—For matched studies, give matching criteria and number of exposed and unexposed *Case-control study*—For matched studies, give matching criteria and the number of controls per case	N/A
Variables	7	Clearly define all outcomes, exposures, predictors, potential confounders, and effect modifiers. Give diagnostic criteria, if applicable	Y
Data sources/ measurement	8*	For each variable of interest, give sources of data and details of methods of assessment (measurement). Describe comparability of assessment methods if there is more than one group	Y

Bias	9	Describe any efforts to address potential sources of bias	B
Study size	10	Explain how the study size was arrived at	Y
Quantitative variables	11	Explain how quantitative variables were handled in the analyses. If applicable, describe which groupings were chosen and why	Y
Statistical methods	12	(*a*) Describe all statistical methods, including those used to control for confounding	Y
		(*b*) Describe any methods used to examine subgroups and interactions	Y
		(*c*) Explain how missing data were addressed	Y
		(*d*) *Cohort study*—If applicable, explain how loss to follow-up was addressed *Case-control study*—If applicable, explain how matching of cases and controls was addressed *Cross-sectional study*—If applicable, describe analytical methods taking account of sampling strategy	N/A
		(*e*) Describe any sensitivity analyses	Y
**Results**			
Participants	13*	(a) Report numbers of individuals at each stage of study—eg numbers potentially eligible, examined for eligibility, confirmed eligible, included in the study, completing follow-up, and analysed	Y
		(b) Give reasons for non-participation at each stage	N/A
		(c) Consider use of a flow diagram	Y
Descriptive data	14*	(a) Give characteristics of study participants (eg demographic, clinical, social) and information on exposures and potential confounders	Y
		(b) Indicate number of participants with missing data for each variable of interest	Y
		(c) *Cohort study*—Summarise follow-up time (eg, average and total amount)	N/A
Outcome data	15*	*Cohort study*—Report numbers of outcome events or summary measures over time	N/A
		Case-control study—Report numbers in each exposure category, or summary measures of exposure	N/A
		Cross-sectional study—Report numbers of outcome events or summary measures	*Y*
Main results	16	(*a*) Give unadjusted estimates and, if applicable, confounder-adjusted estimates and their precision (eg, 95% confidence interval). Make clear which confounders were adjusted for and why they were included	Y
		(*b*) Report category boundaries when	N/A

		continuous variables were categorized	
		(*c*) If relevant, consider translating estimates of relative risk into absolute risk for a meaningful time period	N/A
Other analyses	17	Report other analyses done—eg analyses of subgroups and interactions, and sensitivity analyses	Y
**Discussion**			
Key results	18	Summarise key results with reference to study objectives	Y
Limitations	19	Discuss limitations of the study, taking into account sources of potential bias or imprecision. Discuss both direction and magnitude of any potential bias	Y
Interpretation	20	Give a cautious overall interpretation of results considering objectives, limitations, multiplicity of analyses, results from similar studies, and other relevant evidence	Y
Generalisability	21	Discuss the generalisability (external validity) of the study results	Y
**Other information**			
Funding	22	Give the source of funding and the role of the funders for the present study and, if applicable, for the original study on which the present article is based	Y

Hopping, B.N.; Erber, E.; Mead, E.; Sheehy, T.; Roache, C.; Sharma, S. Socioeconomic indicators and frequency of traditional food, junk food, and fruit and vegetable consumption amongst Inuit adults in the Canadian Arctic. J Hum Nutr Diet 2010, 23 Suppl 1, 51-58, doi:10.1111/j.1365-277X.2010.01100.x.

STROBE Statement—checklist of items that should be included in reports of observational studies

	Item No	Recommendation	Recommendation met? (Y/N)
**Title and abstract**	1	(*a*) Indicate the study's design with a commonly used term in the title or the abstract	Y
		(*b*) Provide in the abstract an informative and balanced summary of what was done and what was found	Y
**Introduction**			
Background/rationale	2	Explain the scientific background and rationale for the investigation being reported	Y
Objectives	3	State specific objectives, including any prespecified hypotheses	Y
**Methods**			
Study design	4	Present key elements of study design early in the paper	Y
Setting	5	Describe the setting, locations, and relevant dates, including periods of recruitment, exposure, follow-up, and data collection	Y
Participants	6	(*a*) *Cohort study*—Give the eligibility criteria, and the sources and methods of selection of participants. Describe methods of follow-up<br>*Case-control study*—Give the eligibility criteria, and the sources and methods of case ascertainment and control selection. Give the rationale for the choice of cases and controls<br>*Cross-sectional study*—Give the eligibility criteria, and the sources and methods of selection of participants	Y
		(*b*) *Cohort study*—For matched studies, give matching criteria and number of exposed and unexposed<br>*Case-control study*—For matched studies, give matching criteria and the number of controls per case	N/A
Variables	7	Clearly define all outcomes, exposures, predictors, potential confounders, and effect modifiers. Give diagnostic criteria, if applicable	Y
Data sources/ measurement	8*	For each variable of interest, give sources of data and details of methods of assessment (measurement). Describe comparability of assessment methods if there is more than one group	Y

Bias	9	Describe any efforts to address potential sources of bias	Y
Study size	10	Explain how the study size was arrived at	Y
Quantitative variables	11	Explain how quantitative variables were handled in the analyses. If applicable, describe which groupings were chosen and why	Y
Statistical methods	12	(*a*) Describe all statistical methods, including those used to control for confounding	Y
		(*b*) Describe any methods used to examine subgroups and interactions	Y
		(*c*) Explain how missing data were addressed	N
		(*d*) *Cohort study*—If applicable, explain how loss to follow-up was addressed<br>*Case-control study*—If applicable, explain how matching of cases and controls was addressed<br>*Cross-sectional study*—If applicable, describe analytical methods taking account of sampling strategy	N/A
		(*e*) Describe any sensitivity analyses	N
**Results**			
Participants	13*	(a) Report numbers of individuals at each stage of study—eg numbers potentially eligible, examined for eligibility, confirmed eligible, included in the study, completing follow-up, and analysed	N
		(b) Give reasons for non-participation at each stage	N
		(c) Consider use of a flow diagram	N
Descriptive data	14*	(a) Give characteristics of study participants (eg demographic, clinical, social) and information on exposures and potential confounders	Y
		(b) Indicate number of participants with missing data for each variable of interest	N
		(c) *Cohort study*—Summarise follow-up time (eg, average and total amount)	N/A
Outcome data	15*	*Cohort study*—Report numbers of outcome events or summary measures over time	N/A
		Case-control study—Report numbers in each exposure category, or summary measures of exposure	N/A
		Cross-sectional study—Report numbers of outcome events or summary measures	*Y*
Main results	16	(*a*) Give unadjusted estimates and, if applicable, confounder-adjusted estimates and their precision (eg, 95% confidence interval). Make clear which confounders were adjusted for and why they were included	Y
		(*b*) Report category boundaries when	N/A

		continuous variables were categorized	
		(*c*) If relevant, consider translating estimates of relative risk into absolute risk for a meaningful time period	N/A
Other analyses	17	Report other analyses done—eg analyses of subgroups and interactions, and sensitivity analyses	Y
**Discussion**			
Key results	18	Summarise key results with reference to study objectives	Y
Limitations	19	Discuss limitations of the study, taking into account sources of potential bias or imprecision. Discuss both direction and magnitude of any potential bias	Y
Interpretation	20	Give a cautious overall interpretation of results considering objectives, limitations, multiplicity of analyses, results from similar studies, and other relevant evidence	Y
Generalisability	21	Discuss the generalisability (external validity) of the study results	N
**Other information**			
Funding	22	Give the source of funding and the role of the funders for the present study and, if applicable, for the original study on which the present article is based	Y

Schmidt, R.; Hrenchuk, C.; Bopp, J.; Poole, N. Trajectories of women's homelessness in Canada's 3 northern territories. Int J Circumpolar Health 2015, 74, 29778, doi:10.3402/ijch.v74.29778.

Standards for Reporting Qualitative Research (SRQR)

**No. Topic**	**Item**	**0 – no 1 – maybe 2-Yes**
**Title and abstract**		
S1 Title	Concise description of the nature and topic of the study identifying the study as qualitative or indicating the approach (e.g., ethnography, grounded theory) or data collection methods (e.g., interview, focus group) is recommended	2
S2 Abstract	Summary of key elements of the study using the abstract format of the intended publication; typically includes objective, methods, results, and conclusions	2
**Introduction**		
S3 Problem formulation	Description and significance of the problem/phenomenon studied; review of relevant theory and empirical work; problem statement	2
S4 Purpose or research question	Purpose of the study and specific objectives or questions	2
**Methods**		
S5 Qualitative approach and research paradigm	Qualitative approach (e.g., ethnography, grounded theory, case study, phenomenology, narrative research) and guiding theory if appropriate; identifying the research paradigm (e.g., positivist, constructivist/interpretivist) is also recommended	0
S6 Researcher characteristics and reflexivity	Researchers' characteristics that may influence the research, including personal attributes, qualifications/experience, relationship with participants, assumptions, or presuppositions; potential or actual interaction between researchers' characteristics and the research questions, approach, methods, results, or transferability	0
S7 Context	Setting/site and salient contextual factors; rationale[a]	0
S8 Sampling strategy	How and why research participants, documents, or events were selected; criteria for deciding when no further sampling was necessary (e.g., sampling saturation); rationale[a]	2
S9 Ethical issues pertaining to human subjects	Documentation of approval by an appropriate ethics review board and participant consent, or explanation for lack thereof; other confidentiality and data security issues	2
S10 Data collection methods	Types of data collected; details of data collection procedures including (as appropriate) start and stop dates of data collection and analysis, iterative process, triangulation of sources/methods,	2

	and modification of procedures in response to evolving study findings; rationale[a]	
S11 Data collection instruments and technologies	Description of instruments (e.g., interview guides, questionnaires) and devices (e.g., audio recorders) used for data collection; if/how the instrument(s) changed over the course of the study	2
S12 Units of study	Number and relevant characteristics of participants, documents, or events included in the study; level of participation (could be reported in results)	2
S13 Data processing	Methods for processing data prior to and during analysis, including transcription, data entry, data management and security, verification of data integrity, data coding, and anonymization/deidentification of excerpts	2
S14 Data analysis	Process by which inferences, themes, etc., were identified and developed, including researchers involved in data analysis; usually references a specific paradigm or approach; rationale[a]	2
S15 Techniques to enhance trustworthiness	Techniques to enhance trustworthiness and credibility of data analysis (e.g., member checking, audit trail, triangulation); rationale[a]	2
**Results/Findings**		
S16 Synthesis and interpretation	Main findings (e.g., interpretations, inferences, and themes); might include development of a theory or model, or integration with prior research or theory	2
S17 Links to empirical data	Evidence (e.g., quotes, field notes, text excerpts, photographs) to substantiate analytic findings	2
**Discussion**		
S18 Integration with prior work, implications, transferability, and contribution(s) to the field	Short summary of main findings; explanation of how findings and conclusions connect to, support, elaborate on, or challenge conclusions of earlier scholarship; discussion of scope of application/generalizability; identification of unique contribution(s) to scholarship in a discipline or field	2
S19 Limitations	Trustworthiness and limitations of findings	
**Other**		
S20 Conflicts of interest	Potential sources of influence or perceived influence on study conduct and conclusions; how these were managed	2
S21 Funding	Sources of funding and other support; role of funders in data collection, interpretation, and reporting	2

Baron, M.; Riva, M.; Fletcher, C. The social determinants of healthy ageing in the Canadian Arctic. Int J Circumpolar Health 2019, 78, 1630234, doi:10.1080/22423982.2019.1630234.

STROBE Statement—checklist of items that should be included in reports of observational studies

	Item No	Recommendation	Recommendation met? (Y/N)
**Title and abstract**	1	(*a*) Indicate the study's design with a commonly used term in the title or the abstract	Y
		(*b*) Provide in the abstract an informative and balanced summary of what was done and what was found	Y
**Introduction**			
Background/rationale	2	Explain the scientific background and rationale for the investigation being reported	Y
Objectives	3	State specific objectives, including any prespecified hypotheses	Y
**Methods**			
Study design	4	Present key elements of study design early in the paper	Y
Setting	5	Describe the setting, locations, and relevant dates, including periods of recruitment, exposure, follow-up, and data collection	Y
Participants	6	(*a*) *Cohort study*—Give the eligibility criteria, and the sources and methods of selection of participants. Describe methods of follow-up<br>*Case-control study*—Give the eligibility criteria, and the sources and methods of case ascertainment and control selection. Give the rationale for the choice of cases and controls<br>*Cross-sectional study*—Give the eligibility criteria, and the sources and methods of selection of participants	N/A
		(*b*) *Cohort study*—For matched studies, give matching criteria and number of exposed and unexposed<br>*Case-control study*—For matched studies, give matching criteria and the number of controls per case	N/A
Variables	7	Clearly define all outcomes, exposures, predictors, potential confounders, and effect modifiers. Give diagnostic criteria, if applicable	Y
Data sources/ measurement	8*	For each variable of interest, give sources of data and details of methods of assessment (measurement). Describe comparability of assessment methods if there is more than one group	Y
Bias	9	Describe any efforts to address potential	N

		sources of bias	
Study size	10	Explain how the study size was arrived at	Y
Quantitative variables	11	Explain how quantitative variables were handled in the analyses. If applicable, describe which groupings were chosen and why	Y
Statistical methods	12	(*a*) Describe all statistical methods, including those used to control for confounding	Y
		(*b*) Describe any methods used to examine subgroups and interactions	Y
		(*c*) Explain how missing data were addressed	Y
		(*d*) *Cohort study*—If applicable, explain how loss to follow-up was addressed<br>*Case-control study*—If applicable, explain how matching of cases and controls was addressed<br>*Cross-sectional study*—If applicable, describe analytical methods taking account of sampling strategy	N/A
		(*e*) Describe any sensitivity analyses	Y
**Results**			
Participants	13*	(a) Report numbers of individuals at each stage of study—eg numbers potentially eligible, examined for eligibility, confirmed eligible, included in the study, completing follow-up, and analysed	N/A
		(b) Give reasons for non-participation at each stage	N/A
		(c) Consider use of a flow diagram	N/A
Descriptive data	14*	(a) Give characteristics of study participants (eg demographic, clinical, social) and information on exposures and potential confounders	Y
		(b) Indicate number of participants with missing data for each variable of interest	Y
		(c) *Cohort study*—Summarise follow-up time (eg, average and total amount)	N/A
Outcome data	15*	*Cohort study*—Report numbers of outcome events or summary measures over time	N/A
		Case-control study—Report numbers in each exposure category, or summary measures of exposure	N/A
		Cross-sectional study—Report numbers of outcome events or summary measures	*Y*
Main results	16	(*a*) Give unadjusted estimates and, if applicable, confounder-adjusted estimates and their precision (eg, 95% confidence interval). Make clear which confounders were adjusted for and why they were included	Y
		(*b*) Report category boundaries when continuous variables were categorized	N/A

		(*c*) If relevant, consider translating estimates of relative risk into absolute risk for a meaningful time period	N/A
Other analyses	17	Report other analyses done—eg analyses of subgroups and interactions, and sensitivity analyses	Y
**Discussion**			
Key results	18	Summarise key results with reference to study objectives	Y
Limitations	19	Discuss limitations of the study, taking into account sources of potential bias or imprecision. Discuss both direction and magnitude of any potential bias	Y
Interpretation	20	Give a cautious overall interpretation of results considering objectives, limitations, multiplicity of analyses, results from similar studies, and other relevant evidence	Y
Generalisability	21	Discuss the generalisability (external validity) of the study results	N
**Other information**			
Funding	22	Give the source of funding and the role of the funders for the present study and, if applicable, for the original study on which the present article is based	Y

Logie, C.H.; Lys, C.L.; Mackay, K.; MacNeill, N.; Pauchulo, A.; Yasseen, A.S., 3rd. Syndemic Factors Associated with Safer Sex Efficacy Among Northern and Indigenous Adolescents in Arctic Canada. Int J Behav Med 2019, 26, 449-453, doi:10.1007/s12529-019-09797-0.

STROBE Statement—checklist of items that should be included in reports of observational studies

	Item No	Recommendation	Recommendation met? (Y/N)
**Title and abstract**	1	(*a*) Indicate the study's design with a commonly used term in the title or the abstract	Y
		(*b*) Provide in the abstract an informative and balanced summary of what was done and what was found	Y
**Introduction**			
Background/rationale	2	Explain the scientific background and rationale for the investigation being reported	Y
Objectives	3	State specific objectives, including any prespecified hypotheses	Y
**Methods**			
Study design	4	Present key elements of study design early in the paper	Y
Setting	5	Describe the setting, locations, and relevant dates, including periods of recruitment, exposure, follow-up, and data collection	Y
Participants	6	(*a*) *Cohort study*—Give the eligibility criteria, and the sources and methods of selection of participants. Describe methods of follow-up<br>*Case-control study*—Give the eligibility criteria, and the sources and methods of case ascertainment and control selection. Give the rationale for the choice of cases and controls<br>*Cross-sectional study*—Give the eligibility criteria, and the sources and methods of selection of participants	Y
		(*b*) *Cohort study*—For matched studies, give matching criteria and number of exposed and unexposed<br>*Case-control study*—For matched studies, give matching criteria and the number of controls per case	N/A
Variables	7	Clearly define all outcomes, exposures, predictors, potential confounders, and effect modifiers. Give diagnostic criteria, if applicable	Y
Data sources/ measurement	8*	For each variable of interest, give sources of data and details of methods of assessment (measurement). Describe comparability of assessment methods if there is more than one group	Y

Bias	9	Describe any efforts to address potential sources of bias	N
Study size	10	Explain how the study size was arrived at	Y
Quantitative variables	11	Explain how quantitative variables were handled in the analyses. If applicable, describe which groupings were chosen and why	Y
Statistical methods	12	(*a*) Describe all statistical methods, including those used to control for confounding	Y
		(*b*) Describe any methods used to examine subgroups and interactions	Y
		(*c*) Explain how missing data were addressed	N
		(*d*) *Cohort study*—If applicable, explain how loss to follow-up was addressed<br>*Case-control study*—If applicable, explain how matching of cases and controls was addressed<br>*Cross-sectional study*—If applicable, describe analytical methods taking account of sampling strategy	N/A
		(*e*) Describe any sensitivity analyses	Y
**Results**			
Participants	13*	(a) Report numbers of individuals at each stage of study—eg numbers potentially eligible, examined for eligibility, confirmed eligible, included in the study, completing follow-up, and analysed	N
		(b) Give reasons for non-participation at each stage	N
		(c) Consider use of a flow diagram	N
Descriptive data	14*	(a) Give characteristics of study participants (eg demographic, clinical, social) and information on exposures and potential confounders	Y
		(b) Indicate number of participants with missing data for each variable of interest	Y
		(c) *Cohort study*—Summarise follow-up time (eg, average and total amount)	N/A
Outcome data	15*	*Cohort study*—Report numbers of outcome events or summary measures over time	N/A
		Case-control study—Report numbers in each exposure category, or summary measures of exposure	N/A
		Cross-sectional study—Report numbers of outcome events or summary measures	*Y*
Main results	16	(*a*) Give unadjusted estimates and, if applicable, confounder-adjusted estimates and their precision (eg, 95% confidence interval). Make clear which confounders were adjusted for and why they were included	Y
		(*b*) Report category boundaries when	N/A

		continuous variables were categorized	
		(*c*) If relevant, consider translating estimates of relative risk into absolute risk for a meaningful time period	N/A
Other analyses	17	Report other analyses done—eg analyses of subgroups and interactions, and sensitivity analyses	Y
**Discussion**			
Key results	18	Summarise key results with reference to study objectives	Y
Limitations	19	Discuss limitations of the study, taking into account sources of potential bias or imprecision. Discuss both direction and magnitude of any potential bias	Y
Interpretation	20	Give a cautious overall interpretation of results considering objectives, limitations, multiplicity of analyses, results from similar studies, and other relevant evidence	Y
Generalisability	21	Discuss the generalisability (external validity) of the study results	N
**Other information**			
Funding	22	Give the source of funding and the role of the funders for the present study and, if applicable, for the original study on which the present article is based	Y

Young, T.K. Sociocultural and behavioural determinants of obesity among Inuit in the central Canadian Arctic. Soc Sci Med 1996, 43, 1665-1671, doi:10.1016/s0277-9536(96)00075-5.

STROBE Statement—checklist of items that should be included in reports of observational studies

	**Item No**	**Recommendation**	**Recommendation met? (Y/N)**
**Title and abstract**	1	(*a*) Indicate the study's design with a commonly used term in the title or the abstract	Y
		(*b*) Provide in the abstract an informative and balanced summary of what was done and what was found	Y
**Introduction**			
Background/rationale	2	Explain the scientific background and rationale for the investigation being reported	Y
Objectives	3	State specific objectives, including any prespecified hypotheses	Y
**Methods**			
Study design	4	Present key elements of study design early in the paper	Y
Setting	5	Describe the setting, locations, and relevant dates, including periods of recruitment, exposure, follow-up, and data collection	Y
Participants	6	(*a*) *Cohort study*—Give the eligibility criteria, and the sources and methods of selection of participants. Describe methods of follow-up *Case-control study*—Give the eligibility criteria, and the sources and methods of case ascertainment and control selection. Give the rationale for the choice of cases and controls *Cross-sectional study*—Give the eligibility criteria, and the sources and methods of selection of participants	Y
		(*b*) *Cohort study*—For matched studies, give matching criteria and number of exposed and unexposed *Case-control study*—For matched studies, give matching criteria and the number of controls per case	N/A
Variables	7	Clearly define all outcomes, exposures, predictors, potential confounders, and effect modifiers. Give diagnostic criteria, if applicable	Y
Data sources/ measurement	8*	For each variable of interest, give sources of data and details of methods of assessment (measurement). Describe comparability of assessment methods if there is more than one group	Y
Bias	9	Describe any efforts to address potential	Y

		sources of bias	
Study size	10	Explain how the study size was arrived at	Y
Quantitative variables	11	Explain how quantitative variables were handled in the analyses. If applicable, describe which groupings were chosen and why	Y
Statistical methods	12	(*a*) Describe all statistical methods, including those used to control for confounding	Y
		(*b*) Describe any methods used to examine subgroups and interactions	N
		(*c*) Explain how missing data were addressed	Y
		(*d*) *Cohort study*—If applicable, explain how loss to follow-up was addressed<br>*Case-control study*—If applicable, explain how matching of cases and controls was addressed<br>*Cross-sectional study*—If applicable, describe analytical methods taking account of sampling strategy	N/A
		(*e*) Describe any sensitivity analyses	N
**Results**			
Participants	13*	(a) Report numbers of individuals at each stage of study—eg numbers potentially eligible, examined for eligibility, confirmed eligible, included in the study, completing follow-up, and analysed	Y
		(b) Give reasons for non-participation at each stage	Y
		(c) Consider use of a flow diagram	N
Descriptive data	14*	(a) Give characteristics of study participants (eg demographic, clinical, social) and information on exposures and potential confounders	Y
		(b) Indicate number of participants with missing data for each variable of interest	Y
		(c) *Cohort study*—Summarise follow-up time (eg, average and total amount)	
Outcome data	15*	*Cohort study*—Report numbers of outcome events or summary measures over time	N/A
		Case-control study—Report numbers in each exposure category, or summary measures of exposure	N/A
		Cross-sectional study—Report numbers of outcome events or summary measures	*Y*
Main results	16	(*a*) Give unadjusted estimates and, if applicable, confounder-adjusted estimates and their precision (eg, 95% confidence interval). Make clear which confounders were adjusted for and why they were included	N/A
		(*b*) Report category boundaries when continuous variables were categorized	N/A

		(*c*) If relevant, consider translating estimates of relative risk into absolute risk for a meaningful time period	N/A
Other analyses	17	Report other analyses done—eg analyses of subgroups and interactions, and sensitivity analyses	Y
**Discussion**			
Key results	18	Summarise key results with reference to study objectives	Y
Limitations	19	Discuss limitations of the study, taking into account sources of potential bias or imprecision. Discuss both direction and magnitude of any potential bias	N
Interpretation	20	Give a cautious overall interpretation of results considering objectives, limitations, multiplicity of analyses, results from similar studies, and other relevant evidence	Y
Generalisability	21	Discuss the generalisability (external validity) of the study results	N
**Other information**			
Funding	22	Give the source of funding and the role of the funders for the present study and, if applicable, for the original study on which the present article is based	Y

Young, M.G.; Manion, K. Harm reduction through housing first: an assessment of the Emergency Warming Centre in Inuvik, Canada. Harm Reduct J 2017, 14, 8, doi:10.1186/s12954-016-0128-8.

Standards for Reporting Qualitative Research (SRQR)

**No. Topic**	**Item**	**0 – no 1 – maybe 2-Yes**
**Title and abstract**		
S1 Title	Concise description of the nature and topic of the study identifying the study as qualitative or indicating the approach (e.g., ethnography, grounded theory) or data collection methods (e.g., interview, focus group) is recommended	2
S2 Abstract	Summary of key elements of the study using the abstract format of the intended publication; typically includes objective, methods, results, and conclusions	2
**Introduction**		
S3 Problem formulation	Description and significance of the problem/phenomenon studied; review of relevant theory and empirical work; problem statement	2
S4 Purpose or research question	Purpose of the study and specific objectives or questions	2
**Methods**		
S5 Qualitative approach and research paradigm	Qualitative approach (e.g., ethnography, grounded theory, case study, phenomenology, narrative research) and guiding theory if appropriate; identifying the research paradigm (e.g., positivist, constructivist/interpretivist) is also recommended	0
S6 Researcher characteristics and reflexivity	Researchers' characteristics that may influence the research, including personal attributes, qualifications/experience, relationship with participants, assumptions, or presuppositions; potential or actual interaction between researchers' characteristics and the research questions, approach, methods, results, or transferability	0
S7 Context	Setting/site and salient contextual factors; rationale[a]	2
S8 Sampling strategy	How and why research participants, documents, or events were selected; criteria for deciding when no further sampling was necessary (e.g., sampling saturation); rationale[a]	2
S9 Ethical issues pertaining to human subjects	Documentation of approval by an appropriate ethics review board and participant consent, or explanation for lack thereof; other confidentiality and data security issues	2
S10 Data collection methods	Types of data collected; details of data collection procedures including (as appropriate) start and stop dates of data collection and analysis, iterative process, triangulation of sources/methods,	2

	and modification of procedures in response to evolving study findings; rationale[a]	
S11 Data collection instruments and technologies	Description of instruments (e.g., interview guides, questionnaires) and devices (e.g., audio recorders) used for data collection; if/how the instrument(s) changed over the course of the study	2
S12 Units of study	Number and relevant characteristics of participants, documents, or events included in the study; level of participation (could be reported in results)	2
S13 Data processing	Methods for processing data prior to and during analysis, including transcription, data entry, data management and security, verification of data integrity, data coding, and anonymization/deidentification of excerpts	2
S14 Data analysis	Process by which inferences, themes, etc., were identified and developed, including researchers involved in data analysis; usually references a specific paradigm or approach; rationale[a]	2
S15 Techniques to enhance trustworthiness	Techniques to enhance trustworthiness and credibility of data analysis (e.g., member checking, audit trail, triangulation); rationale[a]	2
**Results/Findings**		
S16 Synthesis and interpretation	Main findings (e.g., interpretations, inferences, and themes); might include development of a theory or model, or integration with prior research or theory	2
S17 Links to empirical data	Evidence (e.g., quotes, field notes, text excerpts, photographs) to substantiate analytic findings	2
**Discussion**		
S18 Integration with prior work, implications, transferability, and contribution(s) to the field	Short summary of main findings; explanation of how findings and conclusions connect to, support, elaborate on, or challenge conclusions of earlier scholarship; discussion of scope of application/generalizability; identification of unique contribution(s) to scholarship in a discipline or field	2
S19 Limitations	Trustworthiness and limitations of findings	0
**Other**		
S20 Conflicts of interest	Potential sources of influence or perceived influence on study conduct and conclusions; how these were managed	2
S21 Funding	Sources of funding and other support; role of funders in data collection, interpretation, and reporting	2

Chatwood, S.; Paulette, F.; Baker, G.R.; Eriksen, A.M.A.; Hansen, K.L.; Eriksen, H.; Hiratsuka, V.; Lavoie, J.; Lou, W.; Mauro, I.; et al. Indigenous Values and Health Systems Stewardship in Circumpolar Countries. Int J Environ Res Public Health 2017, 14, doi:10.3390/ijerph14121462.

Standards for Reporting Qualitative Research (SRQR)

**No. Topic**	**Item**	**0 – no 1 – maybe 2-Yes**
**Title and abstract**		
S1 Title	Concise description of the nature and topic of the study identifying the study as qualitative or indicating the approach (e.g., ethnography, grounded theory) or data collection methods (e.g., interview, focus group) is recommended	0
S2 Abstract	Summary of key elements of the study using the abstract format of the intended publication; typically includes objective, methods, results, and conclusions	2
**Introduction**		
S3 Problem formulation	Description and significance of the problem/phenomenon studied; review of relevant theory and empirical work; problem statement	2
S4 Purpose or research question	Purpose of the study and specific objectives or questions	2
**Methods**		
S5 Qualitative approach and research paradigm	Qualitative approach (e.g., ethnography, grounded theory, case study, phenomenology, narrative research) and guiding theory if appropriate; identifying the research paradigm (e.g., positivist, constructivist/interpretivist) is also recommended	2
S6 Researcher characteristics and reflexivity	Researchers' characteristics that may influence the research, including personal attributes, qualifications/experience, relationship with participants, assumptions, or presuppositions; potential or actual interaction between researchers' characteristics and the research questions, approach, methods, results, or transferability	2
S7 Context	Setting/site and salient contextual factors; rationale[a]	2
S8 Sampling strategy	How and why research participants, documents, or events were selected; criteria for deciding when no further sampling was necessary (e.g., sampling saturation); rationale[a]	2
S9 Ethical issues pertaining to human subjects	Documentation of approval by an appropriate ethics review board and participant consent, or explanation for lack thereof; other confidentiality and data security issues	0
S10 Data collection methods	Types of data collected; details of data collection procedures including (as appropriate) start and stop dates of data collection	2

	and analysis, iterative process, triangulation of sources/methods, and modification of procedures in response to evolving study findings; rationale[a]	
S11 Data collection instruments and technologies	Description of instruments (e.g., interview guides, questionnaires) and devices (e.g., audio recorders) used for data collection; if/how the instrument(s) changed over the course of the study	2
S12 Units of study	Number and relevant characteristics of participants, documents, or events included in the study; level of participation (could be reported in results)	2
S13 Data processing	Methods for processing data prior to and during analysis, including transcription, data entry, data management and security, verification of data integrity, data coding, and anonymization/deidentification of excerpts	2
S14 Data analysis	Process by which inferences, themes, etc., were identified and developed, including researchers involved in data analysis; usually references a specific paradigm or approach; rationale[a]	2
S15 Techniques to enhance trustworthiness	Techniques to enhance trustworthiness and credibility of data analysis (e.g., member checking, audit trail, triangulation); rationale[a]	2
**Results/Findings**		
S16 Synthesis and interpretation	Main findings (e.g., interpretations, inferences, and themes); might include development of a theory or model, or integration with prior research or theory	2
S17 Links to empirical data	Evidence (e.g., quotes, field notes, text excerpts, photographs) to substantiate analytic findings	2
**Discussion**		
S18 Integration with prior work, implications, transferability, and contribution(s) to the field	Short summary of main findings; explanation of how findings and conclusions connect to, support, elaborate on, or challenge conclusions of earlier scholarship; discussion of scope of application/generalizability; identification of unique contribution(s) to scholarship in a discipline or field	2
S19 Limitations	Trustworthiness and limitations of findings	2
**Other**		
S20 Conflicts of interest	Potential sources of influence or perceived influence on study conduct and conclusions; how these were managed	2
S21 Funding	Sources of funding and other support; role of funders in data collection, interpretation, and reporting	2

Kral, M.J.; Idlout, L.; Minore, J.B.; Dyck, R.J.; Kirmayer, L.J. Unikkaartuit: meanings of well-being, unhappiness, health, and community change among Inuit in Nunavut, Canada. Am J Community Psychol 2011, 48, 426-438, doi:10.1007/s10464-011-9431-4.

Standards for Reporting Qualitative Research (SRQR)

**No. Topic**	**Item**	**0 – no 1 – maybe 2-Yes**
**Title and abstract**		
S1 Title	Concise description of the nature and topic of the study identifying the study as qualitative or indicating the approach (e.g., ethnography, grounded theory) or data collection methods (e.g., interview, focus group) is recommended	1
S2 Abstract	Summary of key elements of the study using the abstract format of the intended publication; typically includes objective, methods, results, and conclusions	2
**Introduction**		
S3 Problem formulation	Description and significance of the problem/phenomenon studied; review of relevant theory and empirical work; problem statement	2
S4 Purpose or research question	Purpose of the study and specific objectives or questions	2
**Methods**		
S5 Qualitative approach and research paradigm	Qualitative approach (e.g., ethnography, grounded theory, case study, phenomenology, narrative research) and guiding theory if appropriate; identifying the research paradigm (e.g., positivist, constructivist/interpretivist) is also recommended	2
S6 Researcher characteristics and reflexivity	Researchers' characteristics that may influence the research, including personal attributes, qualifications/experience, relationship with participants, assumptions, or presuppositions; potential or actual interaction between researchers' characteristics and the research questions, approach, methods, results, or transferability	2
S7 Context	Setting/site and salient contextual factors; rationale[a]	2
S8 Sampling strategy	How and why research participants, documents, or events were selected; criteria for deciding when no further sampling was necessary (e.g., sampling saturation); rationale[a]	2
S9 Ethical issues pertaining to human subjects	Documentation of approval by an appropriate ethics review board and participant consent, or explanation for lack thereof; other confidentiality and data security issues	2
S10 Data collection methods	Types of data collected; details of data collection procedures including (as appropriate) start and stop dates of data collection	2

	and analysis, iterative process, triangulation of sources/methods, and modification of procedures in response to evolving study findings; rationale[a]	
S11 Data collection instruments and technologies	Description of instruments (e.g., interview guides, questionnaires) and devices (e.g., audio recorders) used for data collection; if/how the instrument(s) changed over the course of the study	2
S12 Units of study	Number and relevant characteristics of participants, documents, or events included in the study; level of participation (could be reported in results)	2
S13 Data processing	Methods for processing data prior to and during analysis, including transcription, data entry, data management and security, verification of data integrity, data coding, and anonymization/deidentification of excerpts	2
S14 Data analysis	Process by which inferences, themes, etc., were identified and developed, including researchers involved in data analysis; usually references a specific paradigm or approach; rationale[a]	2
S15 Techniques to enhance trustworthiness	Techniques to enhance trustworthiness and credibility of data analysis (e.g., member checking, audit trail, triangulation); rationale[a]	2
**Results/Findings**		
S16 Synthesis and interpretation	Main findings (e.g., interpretations, inferences, and themes); might include development of a theory or model, or integration with prior research or theory	2
S17 Links to empirical data	Evidence (e.g., quotes, field notes, text excerpts, photographs) to substantiate analytic findings	2
**Discussion**		
S18 Integration with prior work, implications, transferability, and contribution(s) to the field	Short summary of main findings; explanation of how findings and conclusions connect to, support, elaborate on, or challenge conclusions of earlier scholarship; discussion of scope of application/generalizability; identification of unique contribution(s) to scholarship in a discipline or field	2
S19 Limitations	Trustworthiness and limitations of findings	0
**Other**		
S20 Conflicts of interest	Potential sources of influence or perceived influence on study conduct and conclusions; how these were managed	2
S21 Funding	Sources of funding and other support; role of funders in data collection, interpretation, and reporting	2

Newell, S.L.; Dion, M.L.; Doubleday, N.C. Cultural continuity and Inuit health in Arctic Canada. J Epidemiol Community Health 2020, 74, 64-70, doi:10.1136/jech-2018-211856.

STROBE Statement—checklist of items that should be included in reports of observational studies

	Item No	Recommendation	Recommendation met? (Y/N)
**Title and abstract**	1	(*a*) Indicate the study's design with a commonly used term in the title or the abstract	Y
		(*b*) Provide in the abstract an informative and balanced summary of what was done and what was found	Y
**Introduction**			
Background/rationale	2	Explain the scientific background and rationale for the investigation being reported	Y
Objectives	3	State specific objectives, including any prespecified hypotheses	Y
**Methods**			
Study design	4	Present key elements of study design early in the paper	Y
Setting	5	Describe the setting, locations, and relevant dates, including periods of recruitment, exposure, follow-up, and data collection	Y
Participants	6	(*a*) *Cohort study*—Give the eligibility criteria, and the sources and methods of selection of participants. Describe methods of follow-up<br>*Case-control study*—Give the eligibility criteria, and the sources and methods of case ascertainment and control selection. Give the rationale for the choice of cases and controls<br>*Cross-sectional study*—Give the eligibility criteria, and the sources and methods of selection of participants	Y
		(*b*) *Cohort study*—For matched studies, give matching criteria and number of exposed and unexposed<br>*Case-control study*—For matched studies, give matching criteria and the number of controls per case	N/A
Variables	7	Clearly define all outcomes, exposures, predictors, potential confounders, and effect modifiers. Give diagnostic criteria, if applicable	Y
Data sources/ measurement	8*	For each variable of interest, give sources of data and details of methods of assessment (measurement). Describe comparability of assessment methods if there is more than one group	Y
Bias	9	Describe any efforts to address potential sources of bias	N/A
Study size	10	Explain how the study size was arrived at	Y

Quantitative variables	11	Explain how quantitative variables were handled in the analyses. If applicable, describe which groupings were chosen and why	Y
Statistical methods	12	(*a*) Describe all statistical methods, including those used to control for confounding	Y
		(*b*) Describe any methods used to examine subgroups and interactions	Y
		(*c*) Explain how missing data were addressed	Y
		(*d*) *Cohort study*—If applicable, explain how loss to follow-up was addressed<br>*Case-control study*—If applicable, explain how matching of cases and controls was addressed<br>*Cross-sectional study*—If applicable, describe analytical methods taking account of sampling strategy	N/A
		(*e*) Describe any sensitivity analyses	N
**Results**			
Participants	13*	(a) Report numbers of individuals at each stage of study—eg numbers potentially eligible, examined for eligibility, confirmed eligible, included in the study, completing follow-up, and analysed	Y
		(b) Give reasons for non-participation at each stage	N/A
		(c) Consider use of a flow diagram	N
Descriptive data	14*	(a) Give characteristics of study participants (eg demographic, clinical, social) and information on exposures and potential confounders	Y
		(b) Indicate number of participants with missing data for each variable of interest	Y
		(c) *Cohort study*—Summarise follow-up time (eg, average and total amount)	N/A
Outcome data	15*	*Cohort study*—Report numbers of outcome events or summary measures over time	N/A
		Case-control study—Report numbers in each exposure category, or summary measures of exposure	N/A
		Cross-sectional study—Report numbers of outcome events or summary measures	N
Main results	16	(*a*) Give unadjusted estimates and, if applicable, confounder-adjusted estimates and their precision (eg, 95% confidence interval). Make clear which confounders were adjusted for and why they were included	Y
		(*b*) Report category boundaries when continuous variables were categorized	Y
		(*c*) If relevant, consider translating estimates of relative risk into absolute risk for a meaningful time period	N/A
Other analyses	17	Report other analyses done—eg analyses of subgroups and interactions, and sensitivity analyses	Y
**Discussion**			

Key results	18	Summarise key results with reference to study objectives	Y
Limitations	19	Discuss limitations of the study, taking into account sources of potential bias or imprecision. Discuss both direction and magnitude of any potential bias	Y
Interpretation	20	Give a cautious overall interpretation of results considering objectives, limitations, multiplicity of analyses, results from similar studies, and other relevant evidence	Y
Generalisability	21	Discuss the generalisability (external validity) of the study results	N/A
**Other information**			
Funding	22	Give the source of funding and the role of the funders for the present study and, if applicable, for the original study on which the present article is based	Y

Emanuelsen, K.; Pearce, T.; Oakes, J.; Harper, S.L.; Ford, J.D. Sewing and Inuit women's health in the Canadian Arctic. Soc Sci Med 2020, 265, 113523, doi:10.1016/j.socscimed.2020.113523.

Standards for Reporting Qualitative Research (SRQR)

**No. Topic**	**Item**	**0 – no 1 – maybe 2-Yes**
**Title and abstract**		
S1 Title	Concise description of the nature and topic of the study identifying the study as qualitative or indicating the approach (e.g., ethnography, grounded theory) or data collection methods (e.g., interview, focus group) is recommended	0
S2 Abstract	Summary of key elements of the study using the abstract format of the intended publication; typically includes objective, methods, results, and conclusions	2
**Introduction**		
S3 Problem formulation	Description and significance of the problem/phenomenon studied; review of relevant theory and empirical work; problem statement	2
S4 Purpose or research question	Purpose of the study and specific objectives or questions	2
**Methods**		
S5 Qualitative approach and research paradigm	Qualitative approach (e.g., ethnography, grounded theory, case study, phenomenology, narrative research) and guiding theory if appropriate; identifying the research paradigm (e.g., positivist, constructivist/interpretivist) is also recommended	2
S6 Researcher characteristics and reflexivity	Researchers' characteristics that may influence the research, including personal attributes, qualifications/experience, relationship with participants, assumptions, or presuppositions; potential or actual interaction between researchers' characteristics and the research questions, approach, methods, results, or transferability	2
S7 Context	Setting/site and salient contextual factors; rationale[a]	2
S8 Sampling strategy	How and why research participants, documents, or events were selected; criteria for deciding when no further sampling was necessary (e.g., sampling saturation); rationale[a]	2
S9 Ethical issues pertaining to human subjects	Documentation of approval by an appropriate ethics review board and participant consent, or explanation for lack thereof; other confidentiality and data security issues	2
S10 Data collection methods	Types of data collected; details of data collection procedures including (as appropriate) start and stop dates of data collection and analysis, iterative process, triangulation of sources/methods,	2

	and modification of procedures in response to evolving study findings; rationale[a]	
S11 Data collection instruments and technologies	Description of instruments (e.g., interview guides, questionnaires) and devices (e.g., audio recorders) used for data collection; if/how the instrument(s) changed over the course of the study	2
S12 Units of study	Number and relevant characteristics of participants, documents, or events included in the study; level of participation (could be reported in results)	2
S13 Data processing	Methods for processing data prior to and during analysis, including transcription, data entry, data management and security, verification of data integrity, data coding, and anonymization/deidentification of excerpts	2
S14 Data analysis	Process by which inferences, themes, etc., were identified and developed, including researchers involved in data analysis; usually references a specific paradigm or approach; rationale[a]	2
S15 Techniques to enhance trustworthiness	Techniques to enhance trustworthiness and credibility of data analysis (e.g., member checking, audit trail, triangulation); rationale[a]	2
**Results/Findings**		
S16 Synthesis and interpretation	Main findings (e.g., interpretations, inferences, and themes); might include development of a theory or model, or integration with prior research or theory	2
S17 Links to empirical data	Evidence (e.g., quotes, field notes, text excerpts, photographs) to substantiate analytic findings	2
**Discussion**		
S18 Integration with prior work, implications, transferability, and contribution(s) to the field	Short summary of main findings; explanation of how findings and conclusions connect to, support, elaborate on, or challenge conclusions of earlier scholarship; discussion of scope of application/generalizability; identification of unique contribution(s) to scholarship in a discipline or field	20
S19 Limitations	Trustworthiness and limitations of findings	
**Other**		
S20 Conflicts of interest	Potential sources of influence or perceived influence on study conduct and conclusions; how these were managed	0
S21 Funding	Sources of funding and other support; role of funders in data collection, interpretation, and reporting	2

Glass, C.T.R.; Giles, A.R. Community-based risk messaging in Inuvik, Northwest Territories, Canada. Health Promot Int 2020, 35, 555-561, doi:10.1093/heapro/daz042.

Standards for Reporting Qualitative Research (SRQR)

**No. Topic**	**Item**	**0 – no 1 – maybe 2-Yes**
**Title and abstract**		
S1 Title	Concise description of the nature and topic of the study identifying the study as qualitative or indicating the approach (e.g., ethnography, grounded theory) or data collection methods (e.g., interview, focus group) is recommended	1
S2 Abstract	Summary of key elements of the study using the abstract format of the intended publication; typically includes objective, methods, results, and conclusions	2
**Introduction**		
S3 Problem formulation	Description and significance of the problem/phenomenon studied; review of relevant theory and empirical work; problem statement	2
S4 Purpose or research question	Purpose of the study and specific objectives or questions	2
**Methods**		
S5 Qualitative approach and research paradigm	Qualitative approach (e.g., ethnography, grounded theory, case study, phenomenology, narrative research) and guiding theory if appropriate; identifying the research paradigm (e.g., positivist, constructivist/interpretivist) is also recommended	2
S6 Researcher characteristics and reflexivity	Researchers' characteristics that may influence the research, including personal attributes, qualifications/experience, relationship with participants, assumptions, or presuppositions; potential or actual interaction between researchers' characteristics and the research questions, approach, methods, results, or transferability	2
S7 Context	Setting/site and salient contextual factors; rationale[a]	2
S8 Sampling strategy	How and why research participants, documents, or events were selected; criteria for deciding when no further sampling was necessary (e.g., sampling saturation); rationale[a]	2
S9 Ethical issues pertaining to human subjects	Documentation of approval by an appropriate ethics review board and participant consent, or explanation for lack thereof; other confidentiality and data security issues	2
S10 Data collection methods	Types of data collected; details of data collection procedures including (as appropriate) start and stop dates of data collection and analysis, iterative process, triangulation of sources/methods,	2

	and modification of procedures in response to evolving study findings; rationale[a]	
S11 Data collection instruments and technologies	Description of instruments (e.g., interview guides, questionnaires) and devices (e.g., audio recorders) used for data collection; if/how the instrument(s) changed over the course of the study	2
S12 Units of study	Number and relevant characteristics of participants, documents, or events included in the study; level of participation (could be reported in results)	2
S13 Data processing	Methods for processing data prior to and during analysis, including transcription, data entry, data management and security, verification of data integrity, data coding, and anonymization/deidentification of excerpts	2
S14 Data analysis	Process by which inferences, themes, etc., were identified and developed, including researchers involved in data analysis; usually references a specific paradigm or approach; rationale[a]	2
S15 Techniques to enhance trustworthiness	Techniques to enhance trustworthiness and credibility of data analysis (e.g., member checking, audit trail, triangulation); rationale[a]	2
**Results/Findings**		
S16 Synthesis and interpretation	Main findings (e.g., interpretations, inferences, and themes); might include development of a theory or model, or integration with prior research or theory	2
S17 Links to empirical data	Evidence (e.g., quotes, field notes, text excerpts, photographs) to substantiate analytic findings	2
**Discussion**		
S18 Integration with prior work, implications, transferability, and contribution(s) to the field	Short summary of main findings; explanation of how findings and conclusions connect to, support, elaborate on, or challenge conclusions of earlier scholarship; discussion of scope of application/generalizability; identification of unique contribution(s) to scholarship in a discipline or field	2
S19 Limitations	Trustworthiness and limitations of findings	0
**Other**		
S20 Conflicts of interest	Potential sources of influence or perceived influence on study conduct and conclusions; how these were managed	1
S21 Funding	Sources of funding and other support; role of funders in data collection, interpretation, and reporting	2

Moller, H. "Double culturedness": the "capital" of Inuit nurses. Int J Circumpolar Health 2013, 72, doi:10.3402/ijch.v72i0.21266.

Standards for Reporting Qualitative Research (SRQR)

**No. Topic**	**Item**	**0 – no 1 – maybe 2-Yes**
**Title and abstract**		
S1 Title	Concise description of the nature and topic of the study identifying the study as qualitative or indicating the approach (e.g., ethnography, grounded theory) or data collection methods (e.g., interview, focus group) is recommended	1
S2 Abstract	Summary of key elements of the study using the abstract format of the intended publication; typically includes objective, methods, results, and conclusions	2
**Introduction**		
S3 Problem formulation	Description and significance of the problem/phenomenon studied; review of relevant theory and empirical work; problem statement	2
S4 Purpose or research question	Purpose of the study and specific objectives or questions	2
**Methods**		
S5 Qualitative approach and research paradigm	Qualitative approach (e.g., ethnography, grounded theory, case study, phenomenology, narrative research) and guiding theory if appropriate; identifying the research paradigm (e.g., positivist, constructivist/interpretivist) is also recommended	2
S6 Researcher characteristics and reflexivity	Researchers' characteristics that may influence the research, including personal attributes, qualifications/experience, relationship with participants, assumptions, or presuppositions; potential or actual interaction between researchers' characteristics and the research questions, approach, methods, results, or transferability	2
S7 Context	Setting/site and salient contextual factors; rationale[a]	2
S8 Sampling strategy	How and why research participants, documents, or events were selected; criteria for deciding when no further sampling was necessary (e.g., sampling saturation); rationale[a]	2
S9 Ethical issues pertaining to human subjects	Documentation of approval by an appropriate ethics review board and participant consent, or explanation for lack thereof; other confidentiality and data security issues	2
S10 Data collection methods	Types of data collected; details of data collection procedures including (as appropriate) start and stop dates of data collection and analysis, iterative process, triangulation of sources/methods,	2

	and modification of procedures in response to evolving study findings; rationale[a]	
S11 Data collection instruments and technologies	Description of instruments (e.g., interview guides, questionnaires) and devices (e.g., audio recorders) used for data collection; if/how the instrument(s) changed over the course of the study	2
S12 Units of study	Number and relevant characteristics of participants, documents, or events included in the study; level of participation (could be reported in results)	2
S13 Data processing	Methods for processing data prior to and during analysis, including transcription, data entry, data management and security, verification of data integrity, data coding, and anonymization/deidentification of excerpts	2
S14 Data analysis	Process by which inferences, themes, etc., were identified and developed, including researchers involved in data analysis; usually references a specific paradigm or approach; rationale[a]	2
S15 Techniques to enhance trustworthiness	Techniques to enhance trustworthiness and credibility of data analysis (e.g., member checking, audit trail, triangulation); rationale[a]	1
**Results/Findings**		
S16 Synthesis and interpretation	Main findings (e.g., interpretations, inferences, and themes); might include development of a theory or model, or integration with prior research or theory	2
S17 Links to empirical data	Evidence (e.g., quotes, field notes, text excerpts, photographs) to substantiate analytic findings	2
**Discussion**		
S18 Integration with prior work, implications, transferability, and contribution(s) to the field	Short summary of main findings; explanation of how findings and conclusions connect to, support, elaborate on, or challenge conclusions of earlier scholarship; discussion of scope of application/generalizability; identification of unique contribution(s) to scholarship in a discipline or field	2
S19 Limitations	Trustworthiness and limitations of findings	0
**Other**		
S20 Conflicts of interest	Potential sources of influence or perceived influence on study conduct and conclusions; how these were managed	2
S21 Funding	Sources of funding and other support; role of funders in data collection, interpretation, and reporting	2

Hordyk, S.R.; Macdonald, M.E.; Brassard, P. Inuit interpreters engaged in end-of-life care in Nunavik, Northern Quebec. Int J Circumpolar Health 2017, 76, 1291868, doi:10.1080/22423982.2017.1291868.

Standards for Reporting Qualitative Research (SRQR)

No. Topic	Item	0 – no<br>1 – maybe<br>2-Yes
**Title and abstract**		
S1 Title	Concise description of the nature and topic of the study identifying the study as qualitative or indicating the approach (e.g., ethnography, grounded theory) or data collection methods (e.g., interview, focus group) is recommended	1
S2 Abstract	Summary of key elements of the study using the abstract format of the intended publication; typically includes objective, methods, results, and conclusions	2
**Introduction**		
S3 Problem formulation	Description and significance of the problem/phenomenon studied; review of relevant theory and empirical work; problem statement	2
S4 Purpose or research question	Purpose of the study and specific objectives or questions	2
**Methods**		
S5 Qualitative approach and research paradigm	Qualitative approach (e.g., ethnography, grounded theory, case study, phenomenology, narrative research) and guiding theory if appropriate; identifying the research paradigm (e.g., positivist, constructivist/interpretivist) is also recommended	2
S6 Researcher characteristics and reflexivity	Researchers' characteristics that may influence the research, including personal attributes, qualifications/experience, relationship with participants, assumptions, or presuppositions; potential or actual interaction between researchers' characteristics and the research questions, approach, methods, results, or transferability	1
S7 Context	Setting/site and salient contextual factors; rationale[a]	2
S8 Sampling strategy	How and why research participants, documents, or events were selected; criteria for deciding when no further sampling was necessary (e.g., sampling saturation); rationale[a]	2
S9 Ethical issues pertaining to human subjects	Documentation of approval by an appropriate ethics review board and participant consent, or explanation for lack thereof; other confidentiality and data security issues	2
S10 Data collection methods	Types of data collected; details of data collection procedures including (as appropriate) start and stop dates of data collection and analysis, iterative process, triangulation of sources/methods,	2

	and modification of procedures in response to evolving study findings; rationale[a]	
S11 Data collection instruments and technologies	Description of instruments (e.g., interview guides, questionnaires) and devices (e.g., audio recorders) used for data collection; if/how the instrument(s) changed over the course of the study	1
S12 Units of study	Number and relevant characteristics of participants, documents, or events included in the study; level of participation (could be reported in results)	1
S13 Data processing	Methods for processing data prior to and during analysis, including transcription, data entry, data management and security, verification of data integrity, data coding, and anonymization/deidentification of excerpts	1
S14 Data analysis	Process by which inferences, themes, etc., were identified and developed, including researchers involved in data analysis; usually references a specific paradigm or approach; rationale[a]	2
S15 Techniques to enhance trustworthiness	Techniques to enhance trustworthiness and credibility of data analysis (e.g., member checking, audit trail, triangulation); rationale[a]	1
**Results/Findings**		
S16 Synthesis and interpretation	Main findings (e.g., interpretations, inferences, and themes); might include development of a theory or model, or integration with prior research or theory	2
S17 Links to empirical data	Evidence (e.g., quotes, field notes, text excerpts, photographs) to substantiate analytic findings	2
**Discussion**		
S18 Integration with prior work, implications, transferability, and contribution(s) to the field	Short summary of main findings; explanation of how findings and conclusions connect to, support, elaborate on, or challenge conclusions of earlier scholarship; discussion of scope of application/generalizability; identification of unique contribution(s) to scholarship in a discipline or field	2
S19 Limitations	Trustworthiness and limitations of findings	0
**Other**		
S20 Conflicts of interest	Potential sources of influence or perceived influence on study conduct and conclusions; how these were managed	2
S21 Funding	Sources of funding and other support; role of funders in data collection, interpretation, and reporting	2

Kovesi, T.; Gilbert, N.L.; Stocco, C.; Fugler, D.; Dales, R.E.; Guay, M.; Miller, J.D. Indoor air quality and the risk of lower respiratory tract infections in young Canadian Inuit children. CMAJ 2007, 177, 155-160, doi:10.1503/cmaj.061574.

STROBE Statement—checklist of items that should be included in reports of observational studies

	Item No	Recommendation	Recommendation met? (Y/N)
**Title and abstract**	1	(*a*) Indicate the study's design with a commonly used term in the title or the abstract	Y
		(*b*) Provide in the abstract an informative and balanced summary of what was done and what was found	Y
**Introduction**			
Background/rationale	2	Explain the scientific background and rationale for the investigation being reported	Y
Objectives	3	State specific objectives, including any prespecified hypotheses	Y
**Methods**			
Study design	4	Present key elements of study design early in the paper	Y
Setting	5	Describe the setting, locations, and relevant dates, including periods of recruitment, exposure, follow-up, and data collection	Y
Participants	6	(*a*) *Cohort study*—Give the eligibility criteria, and the sources and methods of selection of participants. Describe methods of follow-up *Case-control study*—Give the eligibility criteria, and the sources and methods of case ascertainment and control selection. Give the rationale for the choice of cases and controls *Cross-sectional study*—Give the eligibility criteria, and the sources and methods of selection of participants	Y
		(*b*) *Cohort study*—For matched studies, give matching criteria and number of exposed and unexposed *Case-control study*—For matched studies, give matching criteria and the number of controls per case	N/A
Variables	7	Clearly define all outcomes, exposures, predictors, potential confounders, and effect modifiers. Give diagnostic criteria, if applicable	Y
Data sources/ measurement	8*	For each variable of interest, give sources of data and details of methods of assessment (measurement). Describe comparability of assessment methods if there is more than one group	Y

Bias	9	Describe any efforts to address potential sources of bias	Y
Study size	10	Explain how the study size was arrived at	N
Quantitative variables	11	Explain how quantitative variables were handled in the analyses. If applicable, describe which groupings were chosen and why	Y
Statistical methods	12	(*a*) Describe all statistical methods, including those used to control for confounding	Y
		(*b*) Describe any methods used to examine subgroups and interactions	Y
		(*c*) Explain how missing data were addressed	N
		(*d*) *Cohort study*—If applicable, explain how loss to follow-up was addressed<br>*Case-control study*—If applicable, explain how matching of cases and controls was addressed<br>*Cross-sectional study*—If applicable, describe analytical methods taking account of sampling strategy	N/A
		(*e*) Describe any sensitivity analyses	N
**Results**			
Participants	13*	(a) Report numbers of individuals at each stage of study—eg numbers potentially eligible, examined for eligibility, confirmed eligible, included in the study, completing follow-up, and analysed	N
		(b) Give reasons for non-participation at each stage	N
		(c) Consider use of a flow diagram	N
Descriptive data	14*	(a) Give characteristics of study participants (eg demographic, clinical, social) and information on exposures and potential confounders	Y
		(b) Indicate number of participants with missing data for each variable of interest	N
		(c) *Cohort study*—Summarise follow-up time (eg, average and total amount)	N/A
Outcome data	15*	*Cohort study*—Report numbers of outcome events or summary measures over time	N/A
		Case-control study—Report numbers in each exposure category, or summary measures of exposure	N/A
		Cross-sectional study—Report numbers of outcome events or summary measures	*Y*
Main results	16	(*a*) Give unadjusted estimates and, if applicable, confounder-adjusted estimates and their precision (eg, 95% confidence interval). Make clear which confounders were adjusted for and why they were included	Y
		(*b*) Report category boundaries when	N/A

		continuous variables were categorized	
		(*c*) If relevant, consider translating estimates of relative risk into absolute risk for a meaningful time period	N/A
Other analyses	17	Report other analyses done—eg analyses of subgroups and interactions, and sensitivity analyses	Y
**Discussion**			
Key results	18	Summarise key results with reference to study objectives	Y
Limitations	19	Discuss limitations of the study, taking into account sources of potential bias or imprecision. Discuss both direction and magnitude of any potential bias	Y
Interpretation	20	Give a cautious overall interpretation of results considering objectives, limitations, multiplicity of analyses, results from similar studies, and other relevant evidence	Y
Generalisability	21	Discuss the generalisability (external validity) of the study results	N
**Other information**			
Funding	22	Give the source of funding and the role of the funders for the present study and, if applicable, for the original study on which the present article is based	Y

Pepin, C.; Muckle, G.; Moisan, C.; Forget-Dubois, N.; Riva, M. Household overcrowding and psychological distress among Nunavik Inuit adolescents: a longitudinal study. Int J Circumpolar Health 2018, 77, 1541395, doi:10.1080/22423982.2018.1541395.

STROBE Statement—checklist of items that should be included in reports of observational studies

	Item No	Recommendation	Recommendation met? (Y/N)
**Title and abstract**	1	(*a*) Indicate the study's design with a commonly used term in the title or the abstract	Y
		(*b*) Provide in the abstract an informative and balanced summary of what was done and what was found	Y
**Introduction**			
Background/rationale	2	Explain the scientific background and rationale for the investigation being reported	Y
Objectives	3	State specific objectives, including any prespecified hypotheses	Y
**Methods**			
Study design	4	Present key elements of study design early in the paper	Y
Setting	5	Describe the setting, locations, and relevant dates, including periods of recruitment, exposure, follow-up, and data collection	Y
Participants	6	(*a*) *Cohort study*—Give the eligibility criteria, and the sources and methods of selection of participants. Describe methods of follow-up<br>*Case-control study*—Give the eligibility criteria, and the sources and methods of case ascertainment and control selection. Give the rationale for the choice of cases and controls<br>*Cross-sectional study*—Give the eligibility criteria, and the sources and methods of selection of participants	Y
		(*b*) *Cohort study*—For matched studies, give matching criteria and number of exposed and unexposed<br>*Case-control study*—For matched studies, give matching criteria and the number of controls per case	N/A
Variables	7	Clearly define all outcomes, exposures, predictors, potential confounders, and effect modifiers. Give diagnostic criteria, if applicable	Y
Data sources/ measurement	8*	For each variable of interest, give sources of data and details of methods of assessment (measurement). Describe comparability of assessment methods if there is more than one group	Y

Bias	9	Describe any efforts to address potential sources of bias	Y
Study size	10	Explain how the study size was arrived at	Y
Quantitative variables	11	Explain how quantitative variables were handled in the analyses. If applicable, describe which groupings were chosen and why	Y
Statistical methods	12	(*a*) Describe all statistical methods, including those used to control for confounding	Y
		(*b*) Describe any methods used to examine subgroups and interactions	Y
		(*c*) Explain how missing data were addressed	Y
		(*d*) *Cohort study*—If applicable, explain how loss to follow-up was addressed<br>*Case-control study*—If applicable, explain how matching of cases and controls was addressed<br>*Cross-sectional study*—If applicable, describe analytical methods taking account of sampling strategy	N/A
		(*e*) Describe any sensitivity analyses	Y
**Results**			
Participants	13*	(a) Report numbers of individuals at each stage of study—eg numbers potentially eligible, examined for eligibility, confirmed eligible, included in the study, completing follow-up, and analysed	Y
		(b) Give reasons for non-participation at each stage	Y
		(c) Consider use of a flow diagram	N
Descriptive data	14*	(a) Give characteristics of study participants (eg demographic, clinical, social) and information on exposures and potential confounders	Y
		(b) Indicate number of participants with missing data for each variable of interest	Y
		(c) *Cohort study*—Summarise follow-up time (eg, average and total amount)	N/A
Outcome data	15*	*Cohort study*—Report numbers of outcome events or summary measures over time	N/A
		Case-control study—Report numbers in each exposure category, or summary measures of exposure	N/A
		Cross-sectional study—Report numbers of outcome events or summary measures	*Y*
Main results	16	(*a*) Give unadjusted estimates and, if applicable, confounder-adjusted estimates and their precision (eg, 95% confidence interval). Make clear which confounders were adjusted for and why they were included	Y
		(*b*) Report category boundaries when	N/A

		continuous variables were categorized	
		(*c*) If relevant, consider translating estimates of relative risk into absolute risk for a meaningful time period	N/A
Other analyses	17	Report other analyses done—eg analyses of subgroups and interactions, and sensitivity analyses	Y
**Discussion**			
Key results	18	Summarise key results with reference to study objectives	Y
Limitations	19	Discuss limitations of the study, taking into account sources of potential bias or imprecision. Discuss both direction and magnitude of any potential bias	Y
Interpretation	20	Give a cautious overall interpretation of results considering objectives, limitations, multiplicity of analyses, results from similar studies, and other relevant evidence	Y
Generalisability	21	Discuss the generalisability (external validity) of the study results	N
**Other information**			
Funding	22	Give the source of funding and the role of the funders for the present study and, if applicable, for the original study on which the present article is based	Y

Young, T.K.; Mollins, C.J. The impact of housing on health: an ecologic study from the Canadian Arctic. Arctic Med Res 1996, 55, 52-61.

STROBE Statement—checklist of items that should be included in reports of observational studies

	Item No	Recommendation	Recommendation met? (Y/N)
**Title and abstract**	1	(*a*) Indicate the study's design with a commonly used term in the title or the abstract	Y
		(*b*) Provide in the abstract an informative and balanced summary of what was done and what was found	Y
**Introduction**			
Background/rationale	2	Explain the scientific background and rationale for the investigation being reported	Y
Objectives	3	State specific objectives, including any prespecified hypotheses	Y
**Methods**			
Study design	4	Present key elements of study design early in the paper	Y
Setting	5	Describe the setting, locations, and relevant dates, including periods of recruitment, exposure, follow-up, and data collection	Y
Participants	6	(*a*) *Cohort study*—Give the eligibility criteria, and the sources and methods of selection of participants. Describe methods of follow-up *Case-control study*—Give the eligibility criteria, and the sources and methods of case ascertainment and control selection. Give the rationale for the choice of cases and controls *Cross-sectional study*—Give the eligibility criteria, and the sources and methods of selection of participants	N/A
		(*b*) *Cohort study*—For matched studies, give matching criteria and number of exposed and unexposed *Case-control study*—For matched studies, give matching criteria and the number of controls per case	N/A
Variables	7	Clearly define all outcomes, exposures, predictors, potential confounders, and effect modifiers. Give diagnostic criteria, if applicable	Y
Data sources/ measurement	8*	For each variable of interest, give sources of data and details of methods of assessment (measurement). Describe comparability of assessment methods if there is more than one group	Y
Bias	9	Describe any efforts to address potential	N

		sources of bias	
Study size	10	Explain how the study size was arrived at	Y
Quantitative variables	11	Explain how quantitative variables were handled in the analyses. If applicable, describe which groupings were chosen and why	N
Statistical methods	12	(*a*) Describe all statistical methods, including those used to control for confounding	N
		(*b*) Describe any methods used to examine subgroups and interactions	Y
		(*c*) Explain how missing data were addressed	N
		(*d*) *Cohort study*—If applicable, explain how loss to follow-up was addressed<br>*Case-control study*—If applicable, explain how matching of cases and controls was addressed<br>*Cross-sectional study*—If applicable, describe analytical methods taking account of sampling strategy	N/A
		(*e*) Describe any sensitivity analyses	N
**Results**			
Participants	13*	(a) Report numbers of individuals at each stage of study—eg numbers potentially eligible, examined for eligibility, confirmed eligible, included in the study, completing follow-up, and analysed	N/A
		(b) Give reasons for non-participation at each stage	N/A
		(c) Consider use of a flow diagram	N
Descriptive data	14*	(a) Give characteristics of study participants (eg demographic, clinical, social) and information on exposures and potential confounders	N
		(b) Indicate number of participants with missing data for each variable of interest	N
		(c) *Cohort study*—Summarise follow-up time (eg, average and total amount)	N/A
Outcome data	15*	*Cohort study*—Report numbers of outcome events or summary measures over time	N/A
		Case-control study—Report numbers in each exposure category, or summary measures of exposure	N/A
		Cross-sectional study—Report numbers of outcome events or summary measures	*Y*
Main results	16	(*a*) Give unadjusted estimates and, if applicable, confounder-adjusted estimates and their precision (eg, 95% confidence interval). Make clear which confounders were adjusted for and why they were included	Y
		(*b*) Report category boundaries when continuous variables were categorized	N/A

		(*c*) If relevant, consider translating estimates of relative risk into absolute risk for a meaningful time period	N/A
Other analyses	17	Report other analyses done—eg analyses of subgroups and interactions, and sensitivity analyses	Y
**Discussion**			
Key results	18	Summarise key results with reference to study objectives	Y
Limitations	19	Discuss limitations of the study, taking into account sources of potential bias or imprecision. Discuss both direction and magnitude of any potential bias	N
Interpretation	20	Give a cautious overall interpretation of results considering objectives, limitations, multiplicity of analyses, results from similar studies, and other relevant evidence	Y
Generalisability	21	Discuss the generalisability (external validity) of the study results	N
**Other information**			
Funding	22	Give the source of funding and the role of the funders for the present study and, if applicable, for the original study on which the present article is based	Y

Ruiz-Castell, M.; Muckle, G.; Dewailly, E.; Jacobson, J.L.; Jacobson, S.W.; Ayotte, P.; Riva, M. Household crowding and food insecurity among Inuit families with school-aged children in the Canadian Arctic. Am J Public Health 2015, 105, e122-132, doi:10.2105/AJPH.2014.302290.

STROBE Statement—checklist of items that should be included in reports of observational studies

	**Item No**	**Recommendation**	**Recommendation met? (Y/N)**
**Title and abstract**	1	(*a*) Indicate the study's design with a commonly used term in the title or the abstract	Y
		(*b*) Provide in the abstract an informative and balanced summary of what was done and what was found	Y
**Introduction**			
Background/rationale	2	Explain the scientific background and rationale for the investigation being reported	Y
Objectives	3	State specific objectives, including any prespecified hypotheses	Y
**Methods**			
Study design	4	Present key elements of study design early in the paper	Y
Setting	5	Describe the setting, locations, and relevant dates, including periods of recruitment, exposure, follow-up, and data collection	Y
Participants	6	(*a*) *Cohort study*—Give the eligibility criteria, and the sources and methods of selection of participants. Describe methods of follow-up *Case-control study*—Give the eligibility criteria, and the sources and methods of case ascertainment and control selection. Give the rationale for the choice of cases and controls *Cross-sectional study*—Give the eligibility criteria, and the sources and methods of selection of participants	Y
		(*b*) *Cohort study*—For matched studies, give matching criteria and number of exposed and unexposed *Case-control study*—For matched studies, give matching criteria and the number of controls per case	N/A
Variables	7	Clearly define all outcomes, exposures, predictors, potential confounders, and effect modifiers. Give diagnostic criteria, if applicable	Y
Data sources/ measurement	8*	For each variable of interest, give sources of data and details of methods of assessment (measurement). Describe comparability of assessment methods if there is more than one group	Y

Bias	9	Describe any efforts to address potential sources of bias	N
Study size	10	Explain how the study size was arrived at	Y
Quantitative variables	11	Explain how quantitative variables were handled in the analyses. If applicable, describe which groupings were chosen and why	Y
Statistical methods	12	(*a*) Describe all statistical methods, including those used to control for confounding	Y
		(*b*) Describe any methods used to examine subgroups and interactions	Y
		(*c*) Explain how missing data were addressed	Y
		(*d*) *Cohort study*—If applicable, explain how loss to follow-up was addressed *Case-control study*—If applicable, explain how matching of cases and controls was addressed *Cross-sectional study*—If applicable, describe analytical methods taking account of sampling strategy	N/A
		(*e*) Describe any sensitivity analyses	Y
**Results**			
Participants	13*	(a) Report numbers of individuals at each stage of study—eg numbers potentially eligible, examined for eligibility, confirmed eligible, included in the study, completing follow-up, and analysed	Y
		(b) Give reasons for non-participation at each stage	Y
		(c) Consider use of a flow diagram	N
Descriptive data	14*	(a) Give characteristics of study participants (eg demographic, clinical, social) and information on exposures and potential confounders	Y
		(b) Indicate number of participants with missing data for each variable of interest	Y
		(c) *Cohort study*—Summarise follow-up time (eg, average and total amount)	N/A
Outcome data	15*	*Cohort study*—Report numbers of outcome events or summary measures over time	N/A
		Case-control study—Report numbers in each exposure category, or summary measures of exposure	N/A
		Cross-sectional study—Report numbers of outcome events or summary measures	*Y*
Main results	16	(*a*) Give unadjusted estimates and, if applicable, confounder-adjusted estimates and their precision (eg, 95% confidence interval). Make clear which confounders were adjusted for and why they were included	Y
		(*b*) Report category boundaries when	N/A

		continuous variables were categorized	
		(*c*) If relevant, consider translating estimates of relative risk into absolute risk for a meaningful time period	N/A
Other analyses	17	Report other analyses done—eg analyses of subgroups and interactions, and sensitivity analyses	Y
**Discussion**			
Key results	18	Summarise key results with reference to study objectives	Y
Limitations	19	Discuss limitations of the study, taking into account sources of potential bias or imprecision. Discuss both direction and magnitude of any potential bias	Y
Interpretation	20	Give a cautious overall interpretation of results considering objectives, limitations, multiplicity of analyses, results from similar studies, and other relevant evidence	Y
Generalisability	21	Discuss the generalisability (external validity) of the study results	N
**Other information**			
Funding	22	Give the source of funding and the role of the funders for the present study and, if applicable, for the original study on which the present article is based	Y

Daley, K.; Castleden, H.; Jamieson, R.; Furgal, C.; Ell, L. Municipal water quantities and health in Nunavut households: an exploratory case study in Coral Harbour, Nunavut, Canada. Int J Circumpolar Health 2014, 73, 1-10, doi:10.3402/ijch.v73.23843.

Standards for Reporting Qualitative Research (SRQR)

**No. Topic**	**Item**	**0 – no 1 – maybe 2-Yes**
**Title and abstract**		
S1 Title	Concise description of the nature and topic of the study identifying the study as qualitative or indicating the approach (e.g., ethnography, grounded theory) or data collection methods (e.g., interview, focus group) is recommended	2
S2 Abstract	Summary of key elements of the study using the abstract format of the intended publication; typically includes objective, methods, results, and conclusions	2
**Introduction**		
S3 Problem formulation	Description and significance of the problem/phenomenon studied; review of relevant theory and empirical work; problem statement	2
S4 Purpose or research question	Purpose of the study and specific objectives or questions	2
**Methods**		
S5 Qualitative approach and research paradigm	Qualitative approach (e.g., ethnography, grounded theory, case study, phenomenology, narrative research) and guiding theory if appropriate; identifying the research paradigm (e.g., positivist, constructivist/interpretivist) is also recommended	2
S6 Researcher characteristics and reflexivity	Researchers' characteristics that may influence the research, including personal attributes, qualifications/experience, relationship with participants, assumptions, or presuppositions; potential or actual interaction between researchers' characteristics and the research questions, approach, methods, results, or transferability	2
S7 Context	Setting/site and salient contextual factors; rationale[a]	2
S8 Sampling strategy	How and why research participants, documents, or events were selected; criteria for deciding when no further sampling was necessary (e.g., sampling saturation); rationale[a]	2
S9 Ethical issues pertaining to human subjects	Documentation of approval by an appropriate ethics review board and participant consent, or explanation for lack thereof; other confidentiality and data security issues	2
S10 Data collection methods	Types of data collected; details of data collection procedures including (as appropriate) start and stop dates of data collection	2

	and analysis, iterative process, triangulation of sources/methods, and modification of procedures in response to evolving study findings; rationale[a]	
S11 Data collection instruments and technologies	Description of instruments (e.g., interview guides, questionnaires) and devices (e.g., audio recorders) used for data collection; if/how the instrument(s) changed over the course of the study	2
S12 Units of study	Number and relevant characteristics of participants, documents, or events included in the study; level of participation (could be reported in results)	2
S13 Data processing	Methods for processing data prior to and during analysis, including transcription, data entry, data management and security, verification of data integrity, data coding, and anonymization/deidentification of excerpts	2
S14 Data analysis	Process by which inferences, themes, etc., were identified and developed, including researchers involved in data analysis; usually references a specific paradigm or approach; rationale[a]	2
S15 Techniques to enhance trustworthiness	Techniques to enhance trustworthiness and credibility of data analysis (e.g., member checking, audit trail, triangulation); rationale[a]	2
**Results/Findings**		
S16 Synthesis and interpretation	Main findings (e.g., interpretations, inferences, and themes); might include development of a theory or model, or integration with prior research or theory	2
S17 Links to empirical data	Evidence (e.g., quotes, field notes, text excerpts, photographs) to substantiate analytic findings	2
**Discussion**		
S18 Integration with prior work, implications, transferability, and contribution(s) to the field	Short summary of main findings; explanation of how findings and conclusions connect to, support, elaborate on, or challenge conclusions of earlier scholarship; discussion of scope of application/generalizability; identification of unique contribution(s) to scholarship in a discipline or field	2
S19 Limitations	Trustworthiness and limitations of findings	2
**Other**		
S20 Conflicts of interest	Potential sources of influence or perceived influence on study conduct and conclusions; how these were managed	2
S21 Funding	Sources of funding and other support; role of funders in data collection, interpretation, and reporting	2

Daley, K.; Castleden, H.; Jamieson, R.; Furgal, C.; Ell, L. Water systems, sanitation, and public health risks in remote communities: Inuit resident perspectives from the Canadian Arctic. Social Science & Medicine 2015, 135, 124-132.

Standards for Reporting Qualitative Research (SRQR)

**No. Topic**	**Item**	**0 – no<br>1 – maybe<br>2-Yes**
**Title and abstract**		
S1 Title	Concise description of the nature and topic of the study identifying the study as qualitative or indicating the approach (e.g., ethnography, grounded theory) or data collection methods (e.g., interview, focus group) is recommended	2
S2 Abstract	Summary of key elements of the study using the abstract format of the intended publication; typically includes objective, methods, results, and conclusions	2
**Introduction**		
S3 Problem formulation	Description and significance of the problem/phenomenon studied; review of relevant theory and empirical work; problem statement	2
S4 Purpose or research question	Purpose of the study and specific objectives or questions	2
**Methods**		
S5 Qualitative approach and research paradigm	Qualitative approach (e.g., ethnography, grounded theory, case study, phenomenology, narrative research) and guiding theory if appropriate; identifying the research paradigm (e.g., positivist, constructivist/interpretivist) is also recommended	2
S6 Researcher characteristics and reflexivity	Researchers' characteristics that may influence the research, including personal attributes, qualifications/experience, relationship with participants, assumptions, or presuppositions; potential or actual interaction between researchers' characteristics and the research questions, approach, methods, results, or transferability	2
S7 Context	Setting/site and salient contextual factors; rationale[a]	2
S8 Sampling strategy	How and why research participants, documents, or events were selected; criteria for deciding when no further sampling was necessary (e.g., sampling saturation); rationale[a]	2
S9 Ethical issues pertaining to human subjects	Documentation of approval by an appropriate ethics review board and participant consent, or explanation for lack thereof; other confidentiality and data security issues	2
S10 Data collection methods	Types of data collected; details of data collection procedures including (as appropriate) start and stop dates of data collection	2

	and analysis, iterative process, triangulation of sources/methods, and modification of procedures in response to evolving study findings; rationale[a]	
S11 Data collection instruments and technologies	Description of instruments (e.g., interview guides, questionnaires) and devices (e.g., audio recorders) used for data collection; if/how the instrument(s) changed over the course of the study	2
S12 Units of study	Number and relevant characteristics of participants, documents, or events included in the study; level of participation (could be reported in results)	2
S13 Data processing	Methods for processing data prior to and during analysis, including transcription, data entry, data management and security, verification of data integrity, data coding, and anonymization/deidentification of excerpts	2
S14 Data analysis	Process by which inferences, themes, etc., were identified and developed, including researchers involved in data analysis; usually references a specific paradigm or approach; rationale[a]	2
S15 Techniques to enhance trustworthiness	Techniques to enhance trustworthiness and credibility of data analysis (e.g., member checking, audit trail, triangulation); rationale[a]	2
**Results/Findings**		
S16 Synthesis and interpretation	Main findings (e.g., interpretations, inferences, and themes); might include development of a theory or model, or integration with prior research or theory	2
S17 Links to empirical data	Evidence (e.g., quotes, field notes, text excerpts, photographs) to substantiate analytic findings	2
**Discussion**		
S18 Integration with prior work, implications, transferability, and contribution(s) to the field	Short summary of main findings; explanation of how findings and conclusions connect to, support, elaborate on, or challenge conclusions of earlier scholarship; discussion of scope of application/generalizability; identification of unique contribution(s) to scholarship in a discipline or field	2
S19 Limitations	Trustworthiness and limitations of findings	0
**Other**		
S20 Conflicts of interest	Potential sources of influence or perceived influence on study conduct and conclusions; how these were managed	2
S21 Funding	Sources of funding and other support; role of funders in data collection, interpretation, and reporting	2

Akande, V.O.; Ruiter, R.A.C.; Kremers, S.P.J. Environmental and Motivational Determinants of Physical Activity among Canadian Inuit in the Arctic. Int J Environ Res Public Health 2019, 16, doi:10.3390/ijerph16132437.

STROBE Statement—checklist of items that should be included in reports of observational studies

	Item No	Recommendation	Recommendation met? (Y/N)
**Title and abstract**	1	(*a*) Indicate the study's design with a commonly used term in the title or the abstract	Y
		(*b*) Provide in the abstract an informative and balanced summary of what was done and what was found	Y
**Introduction**			
Background/rationale	2	Explain the scientific background and rationale for the investigation being reported	Y
Objectives	3	State specific objectives, including any prespecified hypotheses	Y
**Methods**			
Study design	4	Present key elements of study design early in the paper	Y
Setting	5	Describe the setting, locations, and relevant dates, including periods of recruitment, exposure, follow-up, and data collection	Y
Participants	6	(*a*) *Cohort study*—Give the eligibility criteria, and the sources and methods of selection of participants. Describe methods of follow-up<br>*Case-control study*—Give the eligibility criteria, and the sources and methods of case ascertainment and control selection. Give the rationale for the choice of cases and controls<br>*Cross-sectional study*—Give the eligibility criteria, and the sources and methods of selection of participants	Y
		(*b*) *Cohort study*—For matched studies, give matching criteria and number of exposed and unexposed<br>*Case-control study*—For matched studies, give matching criteria and the number of controls per case	N/A
Variables	7	Clearly define all outcomes, exposures, predictors, potential confounders, and effect modifiers. Give diagnostic criteria, if applicable	Y
Data sources/ measurement	8*	For each variable of interest, give sources of data and details of methods of assessment (measurement). Describe comparability of assessment methods if there is more than one group	Y

Bias	9	Describe any efforts to address potential sources of bias	Y
Study size	10	Explain how the study size was arrived at	Y
Quantitative variables	11	Explain how quantitative variables were handled in the analyses. If applicable, describe which groupings were chosen and why	Y
Statistical methods	12	(*a*) Describe all statistical methods, including those used to control for confounding	Y
		(*b*) Describe any methods used to examine subgroups and interactions	Y
		(*c*) Explain how missing data were addressed	Y
		(*d*) *Cohort study*—If applicable, explain how loss to follow-up was addressed *Case-control study*—If applicable, explain how matching of cases and controls was addressed *Cross-sectional study*—If applicable, describe analytical methods taking account of sampling strategy	N/A
		(*e*) Describe any sensitivity analyses	N
**Results**			
Participants	13*	(a) Report numbers of individuals at each stage of study—eg numbers potentially eligible, examined for eligibility, confirmed eligible, included in the study, completing follow-up, and analysed	Y
		(b) Give reasons for non-participation at each stage	N
		(c) Consider use of a flow diagram	N
Descriptive data	14*	(a) Give characteristics of study participants (eg demographic, clinical, social) and information on exposures and potential confounders	Y
		(b) Indicate number of participants with missing data for each variable of interest	Y
		(c) *Cohort study*—Summarise follow-up time (eg, average and total amount)	N/A
Outcome data	15*	*Cohort study*—Report numbers of outcome events or summary measures over time	N/A
		Case-control study—Report numbers in each exposure category, or summary measures of exposure	N/A
		Cross-sectional study—Report numbers of outcome events or summary measures	*Y*
Main results	16	(*a*) Give unadjusted estimates and, if applicable, confounder-adjusted estimates and their precision (eg, 95% confidence interval). Make clear which confounders were adjusted for and why they were included	Y
		(*b*) Report category boundaries when	N/A

		continuous variables were categorized	
		(*c*) If relevant, consider translating estimates of relative risk into absolute risk for a meaningful time period	N/A
Other analyses	17	Report other analyses done—eg analyses of subgroups and interactions, and sensitivity analyses	Y
**Discussion**			
Key results	18	Summarise key results with reference to study objectives	Y
Limitations	19	Discuss limitations of the study, taking into account sources of potential bias or imprecision. Discuss both direction and magnitude of any potential bias	Y
Interpretation	20	Give a cautious overall interpretation of results considering objectives, limitations, multiplicity of analyses, results from similar studies, and other relevant evidence	Y
Generalisability	21	Discuss the generalisability (external validity) of the study results	N
**Other information**			
Funding	22	Give the source of funding and the role of the funders for the present study and, if applicable, for the original study on which the present article is based	Y

Baron, M.; Fletcher, C.; Riva, M. Aging, Health and Place from the Perspective of Elders in an Inuit Community. J Cross Cult Gerontol 2020, 35, 133-153, doi:10.1007/s10823-020-09398-5.

Standards for Reporting Qualitative Research (SRQR)

No. Topic	Item	0 – no 1 – maybe 2-Yes
**Title and abstract**		
S1 Title	Concise description of the nature and topic of the study identifying the study as qualitative or indicating the approach (e.g., ethnography, grounded theory) or data collection methods (e.g., interview, focus group) is recommended	2
S2 Abstract	Summary of key elements of the study using the abstract format of the intended publication; typically includes objective, methods, results, and conclusions	2
**Introduction**		
S3 Problem formulation	Description and significance of the problem/phenomenon studied; review of relevant theory and empirical work; problem statement	2
S4 Purpose or research question	Purpose of the study and specific objectives or questions	2
**Methods**		
S5 Qualitative approach and research paradigm	Qualitative approach (e.g., ethnography, grounded theory, case study, phenomenology, narrative research) and guiding theory if appropriate; identifying the research paradigm (e.g., positivist, constructivist/interpretivist) is also recommended	2
S6 Researcher characteristics and reflexivity	Researchers' characteristics that may influence the research, including personal attributes, qualifications/experience, relationship with participants, assumptions, or presuppositions; potential or actual interaction between researchers' characteristics and the research questions, approach, methods, results, or transferability	2
S7 Context	Setting/site and salient contextual factors; rationale[a]	2
S8 Sampling strategy	How and why research participants, documents, or events were selected; criteria for deciding when no further sampling was necessary (e.g., sampling saturation); rationale[a]	2
S9 Ethical issues pertaining to human subjects	Documentation of approval by an appropriate ethics review board and participant consent, or explanation for lack thereof; other confidentiality and data security issues	2
S10 Data collection methods	Types of data collected; details of data collection procedures including (as appropriate) start and stop dates of data collection and analysis, iterative process, triangulation of sources/methods,	2

	and modification of procedures in response to evolving study findings; rationale[a]	
S11 Data collection instruments and technologies	Description of instruments (e.g., interview guides, questionnaires) and devices (e.g., audio recorders) used for data collection; if/how the instrument(s) changed over the course of the study	2
S12 Units of study	Number and relevant characteristics of participants, documents, or events included in the study; level of participation (could be reported in results)	2
S13 Data processing	Methods for processing data prior to and during analysis, including transcription, data entry, data management and security, verification of data integrity, data coding, and anonymization/deidentification of excerpts	2
S14 Data analysis	Process by which inferences, themes, etc., were identified and developed, including researchers involved in data analysis; usually references a specific paradigm or approach; rationale[a]	2
S15 Techniques to enhance trustworthiness	Techniques to enhance trustworthiness and credibility of data analysis (e.g., member checking, audit trail, triangulation); rationale[a]	2
**Results/Findings**		
S16 Synthesis and interpretation	Main findings (e.g., interpretations, inferences, and themes); might include development of a theory or model, or integration with prior research or theory	2
S17 Links to empirical data	Evidence (e.g., quotes, field notes, text excerpts, photographs) to substantiate analytic findings	2
**Discussion**		
S18 Integration with prior work, implications, transferability, and contribution(s) to the field	Short summary of main findings; explanation of how findings and conclusions connect to, support, elaborate on, or challenge conclusions of earlier scholarship; discussion of scope of application/generalizability; identification of unique contribution(s) to scholarship in a discipline or field	2
S19 Limitations	Trustworthiness and limitations of findings	2
**Other**		
S20 Conflicts of interest	Potential sources of influence or perceived influence on study conduct and conclusions; how these were managed	2
S21 Funding	Sources of funding and other support; role of funders in data collection, interpretation, and reporting	2

Akande, V.O.; Fawehinmi, T.O.; Ruiter, R.A.C.; Kremers, S.P.J. Healthy Dietary Choices and Physical Activity Participation in the Canadian Arctic: Understanding Nunavut Inuit Perspectives on the Barriers and Enablers. Int J Environ Res Public Health 2021, 18, doi:10.3390/ijerph18030940.

Standards for Reporting Qualitative Research (SRQR)

**No. Topic**	**Item**	**0 – no 1 – maybe 2-Yes**
**Title and abstract**		
S1 Title	Concise description of the nature and topic of the study identifying the study as qualitative or indicating the approach (e.g., ethnography, grounded theory) or data collection methods (e.g., interview, focus group) is recommended	1
S2 Abstract	Summary of key elements of the study using the abstract format of the intended publication; typically includes objective, methods, results, and conclusions	2
**Introduction**		
S3 Problem formulation	Description and significance of the problem/phenomenon studied; review of relevant theory and empirical work; problem statement	2
S4 Purpose or research question	Purpose of the study and specific objectives or questions	2
**Methods**		
S5 Qualitative approach and research paradigm	Qualitative approach (e.g., ethnography, grounded theory, case study, phenomenology, narrative research) and guiding theory if appropriate; identifying the research paradigm (e.g., positivist, constructivist/interpretivist) is also recommended	2
S6 Researcher characteristics and reflexivity	Researchers' characteristics that may influence the research, including personal attributes, qualifications/experience, relationship with participants, assumptions, or presuppositions; potential or actual interaction between researchers' characteristics and the research questions, approach, methods, results, or transferability	2
S7 Context	Setting/site and salient contextual factors; rationale[a]	2
S8 Sampling strategy	How and why research participants, documents, or events were selected; criteria for deciding when no further sampling was necessary (e.g., sampling saturation); rationale[a]	2
S9 Ethical issues pertaining to human subjects	Documentation of approval by an appropriate ethics review board and participant consent, or explanation for lack thereof; other confidentiality and data security issues	2
S10 Data collection methods	Types of data collected; details of data collection procedures including (as appropriate) start and stop dates of data collection	2

	and analysis, iterative process, triangulation of sources/methods, and modification of procedures in response to evolving study findings; rationale[a]	
S11 Data collection instruments and technologies	Description of instruments (e.g., interview guides, questionnaires) and devices (e.g., audio recorders) used for data collection; if/how the instrument(s) changed over the course of the study	2
S12 Units of study	Number and relevant characteristics of participants, documents, or events included in the study; level of participation (could be reported in results)	2
S13 Data processing	Methods for processing data prior to and during analysis, including transcription, data entry, data management and security, verification of data integrity, data coding, and anonymization/deidentification of excerpts	2
S14 Data analysis	Process by which inferences, themes, etc., were identified and developed, including researchers involved in data analysis; usually references a specific paradigm or approach; rationale[a]	2
S15 Techniques to enhance trustworthiness	Techniques to enhance trustworthiness and credibility of data analysis (e.g., member checking, audit trail, triangulation); rationale[a]	2
**Results/Findings**		
S16 Synthesis and interpretation	Main findings (e.g., interpretations, inferences, and themes); might include development of a theory or model, or integration with prior research or theory	2
S17 Links to empirical data	Evidence (e.g., quotes, field notes, text excerpts, photographs) to substantiate analytic findings	2
**Discussion**		
S18 Integration with prior work, implications, transferability, and contribution(s) to the field	Short summary of main findings; explanation of how findings and conclusions connect to, support, elaborate on, or challenge conclusions of earlier scholarship; discussion of scope of application/generalizability; identification of unique contribution(s) to scholarship in a discipline or field	2
S19 Limitations	Trustworthiness and limitations of findings	2
**Other**		
S20 Conflicts of interest	Potential sources of influence or perceived influence on study conduct and conclusions; how these were managed	2
S21 Funding	Sources of funding and other support; role of funders in data collection, interpretation, and reporting	2

Willox, A.C.; Harper, S.L.; Edge, V.L.; Landman, K.; Houle, K.; Ford, J.D.; Govt, R.I.C. The land enriches the soul: On climatic and environmental change, affect, and emotional health and well-being in Rigolet, Nunatsiavut, Canada. Emot Space Soc 2013, 6, 14-24, doi:10.1016/j.emospa.2011.08.005.

Standards for Reporting Qualitative Research (SRQR)

No. Topic	Item	0 – no<br>1 – maybe<br>2-Yes
**Title and abstract**		
S1 Title	Concise description of the nature and topic of the study identifying the study as qualitative or indicating the approach (e.g., ethnography, grounded theory) or data collection methods (e.g., interview, focus group) is recommended	1
S2 Abstract	Summary of key elements of the study using the abstract format of the intended publication; typically includes objective, methods, results, and conclusions	2
**Introduction**		
S3 Problem formulation	Description and significance of the problem/phenomenon studied; review of relevant theory and empirical work; problem statement	2
S4 Purpose or research question	Purpose of the study and specific objectives or questions	2
**Methods**		
S5 Qualitative approach and research paradigm	Qualitative approach (e.g., ethnography, grounded theory, case study, phenomenology, narrative research) and guiding theory if appropriate; identifying the research paradigm (e.g., positivist, constructivist/interpretivist) is also recommended	0
S6 Researcher characteristics and reflexivity	Researchers' characteristics that may influence the research, including personal attributes, qualifications/experience, relationship with participants, assumptions, or presuppositions; potential or actual interaction between researchers' characteristics and the research questions, approach, methods, results, or transferability	2
S7 Context	Setting/site and salient contextual factors; rationale[a]	2
S8 Sampling strategy	How and why research participants, documents, or events were selected; criteria for deciding when no further sampling was necessary (e.g., sampling saturation); rationale[a]	2
S9 Ethical issues pertaining to human subjects	Documentation of approval by an appropriate ethics review board and participant consent, or explanation for lack thereof; other confidentiality and data security issues	1
S10 Data collection methods	Types of data collected; details of data collection procedures including (as appropriate) start and stop dates of data collection	2

	and analysis, iterative process, triangulation of sources/methods, and modification of procedures in response to evolving study findings; rationale[a]	
S11 Data collection instruments and technologies	Description of instruments (e.g., interview guides, questionnaires) and devices (e.g., audio recorders) used for data collection; if/how the instrument(s) changed over the course of the study	2
S12 Units of study	Number and relevant characteristics of participants, documents, or events included in the study; level of participation (could be reported in results)	2
S13 Data processing	Methods for processing data prior to and during analysis, including transcription, data entry, data management and security, verification of data integrity, data coding, and anonymization/deidentification of excerpts	2
S14 Data analysis	Process by which inferences, themes, etc., were identified and developed, including researchers involved in data analysis; usually references a specific paradigm or approach; rationale[a]	2
S15 Techniques to enhance trustworthiness	Techniques to enhance trustworthiness and credibility of data analysis (e.g., member checking, audit trail, triangulation); rationale[a]	2
**Results/Findings**		
S16 Synthesis and interpretation	Main findings (e.g., interpretations, inferences, and themes); might include development of a theory or model, or integration with prior research or theory	2
S17 Links to empirical data	Evidence (e.g., quotes, field notes, text excerpts, photographs) to substantiate analytic findings	2
**Discussion**		
S18 Integration with prior work, implications, transferability, and contribution(s) to the field	Short summary of main findings; explanation of how findings and conclusions connect to, support, elaborate on, or challenge conclusions of earlier scholarship; discussion of scope of application/generalizability; identification of unique contribution(s) to scholarship in a discipline or field	2
S19 Limitations	Trustworthiness and limitations of findings	0
**Other**		
S20 Conflicts of interest	Potential sources of influence or perceived influence on study conduct and conclusions; how these were managed	0
S21 Funding	Sources of funding and other support; role of funders in data collection, interpretation, and reporting	2

Affleck, W.; Oliffe, J.L.; Inukpuk, M.M.; Tempier, R.; Darroch, F.; Crawford, A.; Séguin, M.S. Suicide amongst young Inuit males: The perspectives of Inuit health and wellness workers in Nunavik. Ssm-Qual Res Health 2022, 2, doi:ARTN 100069

Standards for Reporting Qualitative Research (SRQR)

No. Topic	Item	0 – no<br>1 – maybe<br>2-Yes
**Title and abstract**		
S1 Title	Concise description of the nature and topic of the study identifying the study as qualitative or indicating the approach (e.g., ethnography, grounded theory) or data collection methods (e.g., interview, focus group) is recommended	1
S2 Abstract	Summary of key elements of the study using the abstract format of the intended publication; typically includes objective, methods, results, and conclusions	2
**Introduction**		
S3 Problem formulation	Description and significance of the problem/phenomenon studied; review of relevant theory and empirical work; problem statement	2
S4 Purpose or research question	Purpose of the study and specific objectives or questions	2
**Methods**		
S5 Qualitative approach and research paradigm	Qualitative approach (e.g., ethnography, grounded theory, case study, phenomenology, narrative research) and guiding theory if appropriate; identifying the research paradigm (e.g., positivist, constructivist/interpretivist) is also recommended	0
S6 Researcher characteristics and reflexivity	Researchers' characteristics that may influence the research, including personal attributes, qualifications/experience, relationship with participants, assumptions, or presuppositions; potential or actual interaction between researchers' characteristics and the research questions, approach, methods, results, or transferability	1
S7 Context	Setting/site and salient contextual factors; rationale[a]	2
S8 Sampling strategy	How and why research participants, documents, or events were selected; criteria for deciding when no further sampling was necessary (e.g., sampling saturation); rationale[a]	2
S9 Ethical issues pertaining to human subjects	Documentation of approval by an appropriate ethics review board and participant consent, or explanation for lack thereof; other confidentiality and data security issues	2
S10 Data collection methods	Types of data collected; details of data collection procedures including (as appropriate) start and stop dates of data collection	2

	and analysis, iterative process, triangulation of sources/methods, and modification of procedures in response to evolving study findings; rationale[a]	
S11 Data collection instruments and technologies	Description of instruments (e.g., interview guides, questionnaires) and devices (e.g., audio recorders) used for data collection; if/how the instrument(s) changed over the course of the study	2
S12 Units of study	Number and relevant characteristics of participants, documents, or events included in the study; level of participation (could be reported in results)	2
S13 Data processing	Methods for processing data prior to and during analysis, including transcription, data entry, data management and security, verification of data integrity, data coding, and anonymization/deidentification of excerpts	2
S14 Data analysis	Process by which inferences, themes, etc., were identified and developed, including researchers involved in data analysis; usually references a specific paradigm or approach; rationale[a]	2
S15 Techniques to enhance trustworthiness	Techniques to enhance trustworthiness and credibility of data analysis (e.g., member checking, audit trail, triangulation); rationale[a]	2
**Results/Findings**		
S16 Synthesis and interpretation	Main findings (e.g., interpretations, inferences, and themes); might include development of a theory or model, or integration with prior research or theory	2
S17 Links to empirical data	Evidence (e.g., quotes, field notes, text excerpts, photographs) to substantiate analytic findings	2
**Discussion**		
S18 Integration with prior work, implications, transferability, and contribution(s) to the field	Short summary of main findings; explanation of how findings and conclusions connect to, support, elaborate on, or challenge conclusions of earlier scholarship; discussion of scope of application/generalizability; identification of unique contribution(s) to scholarship in a discipline or field	2
S19 Limitations	Trustworthiness and limitations of findings	2
**Other**		
S20 Conflicts of interest	Potential sources of influence or perceived influence on study conduct and conclusions; how these were managed	2
S21 Funding	Sources of funding and other support; role of funders in data collection, interpretation, and reporting	2

Poliakova, N.; Riva, M.; Fletcher, C.; Desrochers-Couture, M.; Courtemanche, Y.; Moisan, C.; Fraser, S.; Pepin, C.; Belanger, R.E.; Muckle, G. Sociocultural factors in relation to mental health within the Inuit population of Nunavik. Can J Public Health 2024, 115, 83-95, doi:10.17269/s41997-022-00705-w.

STROBE Statement—checklist of items that should be included in reports of observational studies

	Item No	Recommendation	Recommendation met? (Y/N)
**Title and abstract**	1	(*a*) Indicate the study's design with a commonly used term in the title or the abstract	Y
		(*b*) Provide in the abstract an informative and balanced summary of what was done and what was found	Y
**Introduction**			
Background/rationale	2	Explain the scientific background and rationale for the investigation being reported	Y
Objectives	3	State specific objectives, including any prespecified hypotheses	Y
**Methods**			
Study design	4	Present key elements of study design early in the paper	Y
Setting	5	Describe the setting, locations, and relevant dates, including periods of recruitment, exposure, follow-up, and data collection	Y
Participants	6	(*a*) *Cohort study*—Give the eligibility criteria, and the sources and methods of selection of participants. Describe methods of follow-up<br>*Case-control study*—Give the eligibility criteria, and the sources and methods of case ascertainment and control selection. Give the rationale for the choice of cases and controls<br>*Cross-sectional study*—Give the eligibility criteria, and the sources and methods of selection of participants	Y
		(*b*) *Cohort study*—For matched studies, give matching criteria and number of exposed and unexposed<br>*Case-control study*—For matched studies, give matching criteria and the number of controls per case	N/A
Variables	7	Clearly define all outcomes, exposures, predictors, potential confounders, and effect modifiers. Give diagnostic criteria, if applicable	Y
Data sources/ measurement	8*	For each variable of interest, give sources of data and details of methods of assessment (measurement). Describe comparability of assessment methods if there is more than one	Y

		group	
Bias	9	Describe any efforts to address potential sources of bias	N
Study size	10	Explain how the study size was arrived at	Y
Quantitative variables	11	Explain how quantitative variables were handled in the analyses. If applicable, describe which groupings were chosen and why	Y
Statistical methods	12	(*a*) Describe all statistical methods, including those used to control for confounding	Y
		(*b*) Describe any methods used to examine subgroups and interactions	Y
		(*c*) Explain how missing data were addressed	N
		(*d*) *Cohort study*—If applicable, explain how loss to follow-up was addressed<br>*Case-control study*—If applicable, explain how matching of cases and controls was addressed<br>*Cross-sectional study*—If applicable, describe analytical methods taking account of sampling strategy	N/A
		(*e*) Describe any sensitivity analyses	N/A
**Results**			
Participants	13*	(a) Report numbers of individuals at each stage of study—eg numbers potentially eligible, examined for eligibility, confirmed eligible, included in the study, completing follow-up, and analysed	Y
		(b) Give reasons for non-participation at each stage	N/A
		(c) Consider use of a flow diagram	N/A
Descriptive data	14*	(a) Give characteristics of study participants (eg demographic, clinical, social) and information on exposures and potential confounders	Y
		(b) Indicate number of participants with missing data for each variable of interest	N/A
		(c) *Cohort study*—Summarise follow-up time (eg, average and total amount)	N/A
Outcome data	15*	*Cohort study*—Report numbers of outcome events or summary measures over time	N/A
		Case-control study—Report numbers in each exposure category, or summary measures of exposure	N/A
		Cross-sectional study—Report numbers of outcome events or summary measures	Y
Main results	16	(*a*) Give unadjusted estimates and, if applicable, confounder-adjusted estimates and their precision (eg, 95% confidence interval). Make clear which confounders were adjusted for and why they were included	Y

		(*b*) Report category boundaries when continuous variables were categorized	N/A
		(*c*) If relevant, consider translating estimates of relative risk into absolute risk for a meaningful time period	N/A
Other analyses	17	Report other analyses done—eg analyses of subgroups and interactions, and sensitivity analyses	Y
**Discussion**			
Key results	18	Summarise key results with reference to study objectives	Y
Limitations	19	Discuss limitations of the study, taking into account sources of potential bias or imprecision. Discuss both direction and magnitude of any potential bias	Y
Interpretation	20	Give a cautious overall interpretation of results considering objectives, limitations, multiplicity of analyses, results from similar studies, and other relevant evidence	Y
Generalisability	21	Discuss the generalisability (external validity) of the study results	N
**Other information**			
Funding	22	Give the source of funding and the role of the funders for the present study and, if applicable, for the original study on which the present article is based	Y

Beaudoin, V.; Seguin, M.; Chawky, N.; Affleck, W.; Chachamovich, E.; Turecki, G. Protective Factors in the Inuit Population of Nunavut: A Comparative Study of People Who Died by Suicide, People Who Attempted Suicide, and People Who Never Attempted Suicide. Int J Environ Res Public Health 2018, 15, doi:10.3390/ijerph15010144.

Mixed Methods Appraisal Tool (MMAT), version 2018

**Category of study designs**	**Methodological quality criteria**	**Responses**			
		Yes	No	Can't tell	Comments
Screening questions (for all types)	S1. Are there clear research questions?	X			
	S2. Do the collected data allow to address the research questions?	X			
	Further appraisal may not be feasible or appropriate when the answer is 'No' or 'Can't tell' to one or both screening questions.				
1. Qualitative	1.1. Is the qualitative approach appropriate to answer the research question?	N/A			
	1.2. Are the qualitative data collection methods adequate to address the research question?	N/A			
	1.3. Are the findings adequately derived from the data?	N/A			
	1.4. Is the interpretation of results sufficiently substantiated by data?	N/A			
	1.5. Is there coherence between qualitative data sources, collection, analysis and interpretation?	N/A			
2. Quantitative randomized controlled trials	2.1. Is randomization appropriately performed?	N/A			
	2.2. Are the groups comparable at baseline?	N/A			
	2.3. Are there complete outcome data?	N/A			
	2.4. Are outcome assessors blinded to the intervention provided?	N/A			
	2.5 Did the participants adhere to the assigned intervention?	N/A			
3. Quantitative non-randomized	3.1. Are the participants representative of the target population?	N/A			
	3.2. Are measurements appropriate regarding both the outcome and intervention (or exposure)?	N/A			
	3.3. Are there complete outcome data?	N/A			
	3.4. Are the confounders accounted for in the design and analysis?	N/A			
	3.5. During the study period, is the intervention administered (or exposure occurred) as intended?	N/A			
4. Quantitative descriptive	4.1. Is the sampling strategy relevant to address the research question?	N/A			
	4.2. Is the sample representative of the target population?	N/A			
	4.3. Are the measurements appropriate?	N/A			
	4.4. Is the risk of nonresponse bias low?	N/A			

	4.5. Is the statistical analysis appropriate to answer the research question?	N/A			
5. Mixed methods	5.1. Is there an adequate rationale for using a mixed methods design to address the research question?	X			
	5.2. Are the different components of the study effectively integrated to answer the research question?	X			
	5.3. Are the outputs of the integration of qualitative and quantitative components adequately interpreted?	X			
	5.4. Are divergences and inconsistencies between quantitative and qualitative results adequately addressed?	X			
	5.5. Do the different components of the study adhere to the quality criteria of each tradition of the methods involved?	X			

Hansen, V.; Sabo, A.; Korn, J.; MacLean, D.; Riget, F.F.; Clausen, D.S.; Cubley, J. Indoor radon survey in Whitehorse, Canada, and dose assessment. J Radiol Prot 2023, 43, doi:10.1088/1361-6498/acb82a.

STROBE Statement—checklist of items that should be included in reports of observational studies

	Item No	Recommendation	Recommendation met? (Y/N)
**Title and abstract**	1	(*a*) Indicate the study's design with a commonly used term in the title or the abstract	Y
		(*b*) Provide in the abstract an informative and balanced summary of what was done and what was found	Y
**Introduction**			
Background/rationale	2	Explain the scientific background and rationale for the investigation being reported	Y
Objectives	3	State specific objectives, including any prespecified hypotheses	Y
**Methods**			
Study design	4	Present key elements of study design early in the paper	Y
Setting	5	Describe the setting, locations, and relevant dates, including periods of recruitment, exposure, follow-up, and data collection	Y
Participants	6	(*a*) *Cohort study*—Give the eligibility criteria, and the sources and methods of selection of participants. Describe methods of follow-up *Case-control study*—Give the eligibility criteria, and the sources and methods of case ascertainment and control selection. Give the rationale for the choice of cases and controls *Cross-sectional study*—Give the eligibility criteria, and the sources and methods of selection of participants	Y
		(*b*) *Cohort study*—For matched studies, give matching criteria and number of exposed and unexposed *Case-control study*—For matched studies, give matching criteria and the number of controls per case	N/A
Variables	7	Clearly define all outcomes, exposures, predictors, potential confounders, and effect modifiers. Give diagnostic criteria, if applicable	Y
Data sources/ measurement	8*	For each variable of interest, give sources of data and details of methods of assessment (measurement). Describe comparability of assessment methods if there is more than one group	Y

Bias	9	Describe any efforts to address potential sources of bias	Y
Study size	10	Explain how the study size was arrived at	Y
Quantitative variables	11	Explain how quantitative variables were handled in the analyses. If applicable, describe which groupings were chosen and why	Y
Statistical methods	12	(*a*) Describe all statistical methods, including those used to control for confounding	Y
		(*b*) Describe any methods used to examine subgroups and interactions	Y
		(*c*) Explain how missing data were addressed	N/A
		(*d*) *Cohort study*—If applicable, explain how loss to follow-up was addressed<br>*Case-control study*—If applicable, explain how matching of cases and controls was addressed<br>*Cross-sectional study*—If applicable, describe analytical methods taking account of sampling strategy	N/A
		(*e*) Describe any sensitivity analyses	N/A
**Results**			
Participants	13*	(a) Report numbers of individuals at each stage of study—eg numbers potentially eligible, examined for eligibility, confirmed eligible, included in the study, completing follow-up, and analysed	Y
		(b) Give reasons for non-participation at each stage	N/A
		(c) Consider use of a flow diagram	N/A
Descriptive data	14*	(a) Give characteristics of study participants (eg demographic, clinical, social) and information on exposures and potential confounders	N/A
		(b) Indicate number of participants with missing data for each variable of interest	N/A
		(c) *Cohort study*—Summarise follow-up time (eg, average and total amount)	N/A
Outcome data	15*	*Cohort study*—Report numbers of outcome events or summary measures over time	N/A
		Case-control study—Report numbers in each exposure category, or summary measures of exposure	N/A
		Cross-sectional study—Report numbers of outcome events or summary measures	N/A
Main results	16	(*a*) Give unadjusted estimates and, if applicable, confounder-adjusted estimates and their precision (eg, 95% confidence interval). Make clear which confounders were adjusted for and why they were included	N/A
		(*b*) Report category boundaries when	N/A

		continuous variables were categorized	
		(*c*) If relevant, consider translating estimates of relative risk into absolute risk for a meaningful time period	N/A
Other analyses	17	Report other analyses done—eg analyses of subgroups and interactions, and sensitivity analyses	Y
**Discussion**			
Key results	18	Summarise key results with reference to study objectives	Y
Limitations	19	Discuss limitations of the study, taking into account sources of potential bias or imprecision. Discuss both direction and magnitude of any potential bias	Y
Interpretation	20	Give a cautious overall interpretation of results considering objectives, limitations, multiplicity of analyses, results from similar studies, and other relevant evidence	Y
Generalisability	21	Discuss the generalisability (external validity) of the study results	Y
**Other information**			
Funding	22	Give the source of funding and the role of the funders for the present study and, if applicable, for the original study on which the present article is based	N

Cassivi, A.; Carabin, A.; Dorea, C.; Rodriguez, M.J.; Guilherme, S. Domestic access to water in a decentralized truck-to-cistern system: a case study in the Northern Village of Kangiqsualujjuaq, Nunavik (Canada). J Water Health 2024, 22, 797-810, doi:10.2166/wh.2024.246.

Mixed Methods Appraisal Tool (MMAT), version 2018

**Category of study designs**	**Methodological quality criteria**	**Responses**			
		Yes	No	Can't tell	Comments
Screening questions (for all types)	S1. Are there clear research questions?	X			
	S2. Do the collected data allow to address the research questions?	X			
	Further appraisal may not be feasible or appropriate when the answer is 'No' or 'Can't tell' to one or both screening questions.				
1. Qualitative	1.1. Is the qualitative approach appropriate to answer the research question?	N/A			
	1.2. Are the qualitative data collection methods adequate to address the research question?	N/A			
	1.3. Are the findings adequately derived from the data?	N/A			
	1.4. Is the interpretation of results sufficiently substantiated by data?	N/A			
	1.5. Is there coherence between qualitative data sources, collection, analysis and interpretation?	N/A			
2. Quantitative randomized controlled trials	2.1. Is randomization appropriately performed?	N/A			
	2.2. Are the groups comparable at baseline?	N/A			
	2.3. Are there complete outcome data?	N/A			
	2.4. Are outcome assessors blinded to the intervention provided?	N/A			
	2.5 Did the participants adhere to the assigned intervention?	N/A			
3. Quantitative non-randomized	3.1. Are the participants representative of the target population?	N/A			
	3.2. Are measurements appropriate regarding both the outcome and intervention (or exposure)?	N/A			
	3.3. Are there complete outcome data?	N/A			
	3.4. Are the confounders accounted for in the design and analysis?	N/A			
	3.5. During the study period, is the intervention administered (or exposure occurred) as intended?	N/A			
4. Quantitative descriptive	4.1. Is the sampling strategy relevant to address the research question?	N/A			
	4.2. Is the sample representative of the target population?	N/A			
	4.3. Are the measurements appropriate?	N/A			
	4.4. Is the risk of nonresponse bias low?	N/A			

	4.5. Is the statistical analysis appropriate to answer the research question?	N/A			
5. Mixed methods	5.1. Is there an adequate rationale for using a mixed methods design to address the research question?	X			
	5.2. Are the different components of the study effectively integrated to answer the research question?	X			
	5.3. Are the outputs of the integration of qualitative and quantitative components adequately interpreted?	X			
	5.4. Are divergences and inconsistencies between quantitative and qualitative results adequately addressed?	X			
	5.5. Do the different components of the study adhere to the quality criteria of each tradition of the methods involved?		X		

Lavoie, J.G.; Clark, W.; McDonnell, L.; Toor, J.; Nickel, N.; Anang, P.; Kusugak, M.A.; Evaluardjuk-Palmer, T.; Brown, N.; Voisey Clark, G.; et al. Inuit mental health service utilisation in Manitoba: results from the qanuinngitsiarutiksait study. Int J Circumpolar Health 2024, 83, 2413228, doi:10.1080/22423982.2024.2413228.

STROBE Statement—checklist of items that should be included in reports of observational studies

	Item No	Recommendation	Recommendation met? (Y/N)
**Title and abstract**	1	(*a*) Indicate the study's design with a commonly used term in the title or the abstract	Y
		(*b*) Provide in the abstract an informative and balanced summary of what was done and what was found	Y
**Introduction**			
Background/rationale	2	Explain the scientific background and rationale for the investigation being reported	Y
Objectives	3	State specific objectives, including any prespecified hypotheses	Y
**Methods**			
Study design	4	Present key elements of study design early in the paper	Y
Setting	5	Describe the setting, locations, and relevant dates, including periods of recruitment, exposure, follow-up, and data collection	Y
Participants	6	(*a*) *Cohort study*—Give the eligibility criteria, and the sources and methods of selection of participants. Describe methods of follow-up<br>*Case-control study*—Give the eligibility criteria, and the sources and methods of case ascertainment and control selection. Give the rationale for the choice of cases and controls<br>*Cross-sectional study*—Give the eligibility criteria, and the sources and methods of selection of participants	Y
		(*b*) *Cohort study*—For matched studies, give matching criteria and number of exposed and unexposed<br>*Case-control study*—For matched studies, give matching criteria and the number of controls per case	N/A
Variables	7	Clearly define all outcomes, exposures, predictors, potential confounders, and effect modifiers. Give diagnostic criteria, if applicable	Y
Data sources/ measurement	8*	For each variable of interest, give sources of data and details of methods of assessment (measurement). Describe comparability of	Y

		assessment methods if there is more than one group	
Bias	9	Describe any efforts to address potential sources of bias	Y
Study size	10	Explain how the study size was arrived at	Y
Quantitative variables	11	Explain how quantitative variables were handled in the analyses. If applicable, describe which groupings were chosen and why	Y
Statistical methods	12	(*a*) Describe all statistical methods, including those used to control for confounding	N/A
		(*b*) Describe any methods used to examine subgroups and interactions	N/A
		(*c*) Explain how missing data were addressed	N/A
		(*d*) *Cohort study*—If applicable, explain how loss to follow-up was addressed<br>*Case-control study*—If applicable, explain how matching of cases and controls was addressed<br>*Cross-sectional study*—If applicable, describe analytical methods taking account of sampling strategy	N/A
		(*e*) Describe any sensitivity analyses	N/A
**Results**			
Participants	13*	(a) Report numbers of individuals at each stage of study—eg numbers potentially eligible, examined for eligibility, confirmed eligible, included in the study, completing follow-up, and analysed	Y
		(b) Give reasons for non-participation at each stage	N/A
		(c) Consider use of a flow diagram	N/A
Descriptive data	14*	(a) Give characteristics of study participants (eg demographic, clinical, social) and information on exposures and potential confounders	N/A
		(b) Indicate number of participants with missing data for each variable of interest	N/A
		(c) *Cohort study*—Summarise follow-up time (eg, average and total amount)	N/A
Outcome data	15*	*Cohort study*—Report numbers of outcome events or summary measures over time	N/A
		Case-control study—Report numbers in each exposure category, or summary measures of exposure	N/A
		Cross-sectional study—Report numbers of outcome events or summary measures	Y
Main results	16	(*a*) Give unadjusted estimates and, if applicable, confounder-adjusted estimates and their precision (eg, 95% confidence interval). Make clear which confounders were adjusted	N/A

		for and why they were included	
		(*b*) Report category boundaries when continuous variables were categorized	N/A
		(*c*) If relevant, consider translating estimates of relative risk into absolute risk for a meaningful time period	N/A
Other analyses	17	Report other analyses done—eg analyses of subgroups and interactions, and sensitivity analyses	Y
**Discussion**			
Key results	18	Summarise key results with reference to study objectives	Y
Limitations	19	Discuss limitations of the study, taking into account sources of potential bias or imprecision. Discuss both direction and magnitude of any potential bias	Y
Interpretation	20	Give a cautious overall interpretation of results considering objectives, limitations, multiplicity of analyses, results from similar studies, and other relevant evidence	Y
Generalisability	21	Discuss the generalisability (external validity) of the study results	Y
**Other information**			
Funding	22	Give the source of funding and the role of the funders for the present study and, if applicable, for the original study on which the present article is based	Y

.

Clark, W.; Lavoie, J.G.; McDonnell, L.; Nickel, N.; Anawak, J.; Brown, L.; Clark, G.; Evaluardjuk-Palmer, M.; Ford, F.; Dutton, R.; et al. Trends in Inuit health services utilisation in Manitoba: findings from the Qanuinngitsiarutiksait study. Int J Circumpolar Health 2022, 81, 2073069, doi:10.1080/22423982.2022.2073069.

STROBE Statement—checklist of items that should be included in reports of observational studies

	Item No	Recommendation	Recommendation met? (Y/N)
**Title and abstract**	1	(*a*) Indicate the study's design with a commonly used term in the title or the abstract	Y
		(*b*) Provide in the abstract an informative and balanced summary of what was done and what was found	Y
**Introduction**			
Background/rationale	2	Explain the scientific background and rationale for the investigation being reported	Y
Objectives	3	State specific objectives, including any prespecified hypotheses	Y
**Methods**			
Study design	4	Present key elements of study design early in the paper	Y
Setting	5	Describe the setting, locations, and relevant dates, including periods of recruitment, exposure, follow-up, and data collection	Y
Participants	6	(*a*) *Cohort study*—Give the eligibility criteria, and the sources and methods of selection of participants. Describe methods of follow-up<br>*Case-control study*—Give the eligibility criteria, and the sources and methods of case ascertainment and control selection. Give the rationale for the choice of cases and controls<br>*Cross-sectional study*—Give the eligibility criteria, and the sources and methods of selection of participants	Y
		(*b*) *Cohort study*—For matched studies, give matching criteria and number of exposed and unexposed<br>*Case-control study*—For matched studies, give matching criteria and the number of controls per case	N/A
Variables	7	Clearly define all outcomes, exposures, predictors, potential confounders, and effect modifiers. Give diagnostic criteria, if applicable	Y
Data sources/ measurement	8*	For each variable of interest, give sources of data and details of methods of assessment (measurement). Describe comparability of	Y

		assessment methods if there is more than one group	
Bias	9	Describe any efforts to address potential sources of bias	N
Study size	10	Explain how the study size was arrived at	N
Quantitative variables	11	Explain how quantitative variables were handled in the analyses. If applicable, describe which groupings were chosen and why	Y
Statistical methods	12	(*a*) Describe all statistical methods, including those used to control for confounding	N/A
		(*b*) Describe any methods used to examine subgroups and interactions	N/A
		(*c*) Explain how missing data were addressed	N/A
		(*d*) *Cohort study*—If applicable, explain how loss to follow-up was addressed<br>*Case-control study*—If applicable, explain how matching of cases and controls was addressed<br>*Cross-sectional study*—If applicable, describe analytical methods taking account of sampling strategy	N/A
		(*e*) Describe any sensitivity analyses	N/A
**Results**			
Participants	13*	(a) Report numbers of individuals at each stage of study—eg numbers potentially eligible, examined for eligibility, confirmed eligible, included in the study, completing follow-up, and analysed	Y
		(b) Give reasons for non-participation at each stage	N/A
		(c) Consider use of a flow diagram	N/A
Descriptive data	14*	(a) Give characteristics of study participants (eg demographic, clinical, social) and information on exposures and potential confounders	N/A
		(b) Indicate number of participants with missing data for each variable of interest	N/A
		(c) *Cohort study*—Summarise follow-up time (eg, average and total amount)	N/A
Outcome data	15*	*Cohort study*—Report numbers of outcome events or summary measures over time	N/A
		Case-control study—Report numbers in each exposure category, or summary measures of exposure	N/A
		Cross-sectional study—Report numbers of outcome events or summary measures	Y
Main results	16	(*a*) Give unadjusted estimates and, if applicable, confounder-adjusted estimates and their precision (eg, 95% confidence interval). Make clear which confounders were adjusted	N/A

		for and why they were included	
		(*b*) Report category boundaries when continuous variables were categorized	N/A
		(*c*) If relevant, consider translating estimates of relative risk into absolute risk for a meaningful time period	N/A
Other analyses	17	Report other analyses done—eg analyses of subgroups and interactions, and sensitivity analyses	Y
**Discussion**			
Key results	18	Summarise key results with reference to study objectives	Y
Limitations	19	Discuss limitations of the study, taking into account sources of potential bias or imprecision. Discuss both direction and magnitude of any potential bias	Y
Interpretation	20	Give a cautious overall interpretation of results considering objectives, limitations, multiplicity of analyses, results from similar studies, and other relevant evidence	Y
Generalisability	21	Discuss the generalisability (external validity) of the study results	Y
**Other information**			
Funding	22	Give the source of funding and the role of the funders for the present study and, if applicable, for the original study on which the present article is based	Y

.

Falk, R.; Topstad, D. Surgery in the western Canadian Arctic: The relative impact of family physicians with enhanced surgical skills working collaboratively with specialist surgeons. Can J Rural Med 2023, 28, 66-72, doi:10.4103/cjrm.cjrm_44_22.

STROBE Statement—checklist of items that should be included in reports of observational studies

	Item No	Recommendation	Recommendation met? (Y/N)
**Title and abstract**	1	(*a*) Indicate the study's design with a commonly used term in the title or the abstract	Y
		(*b*) Provide in the abstract an informative and balanced summary of what was done and what was found	Y
**Introduction**			
Background/rationale	2	Explain the scientific background and rationale for the investigation being reported	Y
Objectives	3	State specific objectives, including any prespecified hypotheses	Y
**Methods**			
Study design	4	Present key elements of study design early in the paper	Y
Setting	5	Describe the setting, locations, and relevant dates, including periods of recruitment, exposure, follow-up, and data collection	Y
Participants	6	(*a*) *Cohort study*—Give the eligibility criteria, and the sources and methods of selection of participants. Describe methods of follow-up<br>*Case-control study*—Give the eligibility criteria, and the sources and methods of case ascertainment and control selection. Give the rationale for the choice of cases and controls<br>*Cross-sectional study*—Give the eligibility criteria, and the sources and methods of selection of participants	N/A
		(*b*) *Cohort study*—For matched studies, give matching criteria and number of exposed and unexposed<br>*Case-control study*—For matched studies, give matching criteria and the number of controls per case	N/A
Variables	7	Clearly define all outcomes, exposures, predictors, potential confounders, and effect modifiers. Give diagnostic criteria, if applicable	Y
Data sources/ measurement	8*	For each variable of interest, give sources of data and details of methods of assessment (measurement). Describe comparability of assessment methods if there is more than one	Y

		group	
Bias	9	Describe any efforts to address potential sources of bias	N
Study size	10	Explain how the study size was arrived at	N
Quantitative variables	11	Explain how quantitative variables were handled in the analyses. If applicable, describe which groupings were chosen and why	Y
Statistical methods	12	(*a*) Describe all statistical methods, including those used to control for confounding	Y
		(*b*) Describe any methods used to examine subgroups and interactions	N/A
		(*c*) Explain how missing data were addressed	N/A
		(*d*) *Cohort study*—If applicable, explain how loss to follow-up was addressed<br>*Case-control study*—If applicable, explain how matching of cases and controls was addressed<br>*Cross-sectional study*—If applicable, describe analytical methods taking account of sampling strategy	N/A
		(*e*) Describe any sensitivity analyses	N/A
**Results**			
Participants	13*	(a) Report numbers of individuals at each stage of study—eg numbers potentially eligible, examined for eligibility, confirmed eligible, included in the study, completing follow-up, and analysed	N/A
		(b) Give reasons for non-participation at each stage	N/A
		(c) Consider use of a flow diagram	N/A
Descriptive data	14*	(a) Give characteristics of study participants (eg demographic, clinical, social) and information on exposures and potential confounders	N/A
		(b) Indicate number of participants with missing data for each variable of interest	N/A
		(c) *Cohort study*—Summarise follow-up time (eg, average and total amount)	N/A
Outcome data	15*	*Cohort study*—Report numbers of outcome events or summary measures over time	N/A
		Case-control study—Report numbers in each exposure category, or summary measures of exposure	N/A
		Cross-sectional study—Report numbers of outcome events or summary measures	Y
Main results	16	(*a*) Give unadjusted estimates and, if applicable, confounder-adjusted estimates and their precision (eg, 95% confidence interval). Make clear which confounders were adjusted for and why they were included	N/A

		(*b*) Report category boundaries when continuous variables were categorized	N/A
		(*c*) If relevant, consider translating estimates of relative risk into absolute risk for a meaningful time period	N/A
Other analyses	17	Report other analyses done—eg analyses of subgroups and interactions, and sensitivity analyses	Y
**Discussion**			
Key results	18	Summarise key results with reference to study objectives	Y
Limitations	19	Discuss limitations of the study, taking into account sources of potential bias or imprecision. Discuss both direction and magnitude of any potential bias	Y
Interpretation	20	Give a cautious overall interpretation of results considering objectives, limitations, multiplicity of analyses, results from similar studies, and other relevant evidence	Y
Generalisability	21	Discuss the generalisability (external validity) of the study results	Y
**Other information**			
Funding	22	Give the source of funding and the role of the funders for the present study and, if applicable, for the original study on which the present article is based	Y

Falk, R.; Topstad, D.; Lee, L. Surgical Task-Sharing in the Western Canadian Arctic: A Networked Model Between Family Physicians with Enhanced Surgical Skills and Specialist Surgeons. World J Surg 2022, 46, 1629-1636, doi:10.1007/s00268-022-06524-x.

Standards for Reporting Qualitative Research (SRQR)

**No. Topic**	**Item**	**0 – no 1 – maybe 2-Yes**
**Title and abstract**		
S1 Title	Concise description of the nature and topic of the study identifying the study as qualitative or indicating the approach (e.g., ethnography, grounded theory) or data collection methods (e.g., interview, focus group) is recommended	1
S2 Abstract	Summary of key elements of the study using the abstract format of the intended publication; typically includes objective, methods, results, and conclusions	2
**Introduction**		
S3 Problem formulation	Description and significance of the problem/phenomenon studied; review of relevant theory and empirical work; problem statement	2
S4 Purpose or research question	Purpose of the study and specific objectives or questions	2
**Methods**		
S5 Qualitative approach and research paradigm	Qualitative approach (e.g., ethnography, grounded theory, case study, phenomenology, narrative research) and guiding theory if appropriate; identifying the research paradigm (e.g., positivist, constructivist/interpretivist) is also recommended	2
S6 Researcher characteristics and reflexivity	Researchers' characteristics that may influence the research, including personal attributes, qualifications/experience, relationship with participants, assumptions, or presuppositions; potential or actual interaction between researchers' characteristics and the research questions, approach, methods, results, or transferability	1
S7 Context	Setting/site and salient contextual factors; rationale[a]	2
S8 Sampling strategy	How and why research participants, documents, or events were selected; criteria for deciding when no further sampling was necessary (e.g., sampling saturation); rationale[a]	2
S9 Ethical issues pertaining to human subjects	Documentation of approval by an appropriate ethics review board and participant consent, or explanation for lack thereof; other confidentiality and data security issues	2
S10 Data collection methods	Types of data collected; details of data collection procedures including (as appropriate) start and stop dates of data collection	2

	and analysis, iterative process, triangulation of sources/methods, and modification of procedures in response to evolving study findings; rationale[a]	
S11 Data collection instruments and technologies	Description of instruments (e.g., interview guides, questionnaires) and devices (e.g., audio recorders) used for data collection; if/how the instrument(s) changed over the course of the study	2
S12 Units of study	Number and relevant characteristics of participants, documents, or events included in the study; level of participation (could be reported in results)	2
S13 Data processing	Methods for processing data prior to and during analysis, including transcription, data entry, data management and security, verification of data integrity, data coding, and anonymization/deidentification of excerpts	2
S14 Data analysis	Process by which inferences, themes, etc., were identified and developed, including researchers involved in data analysis; usually references a specific paradigm or approach; rationale[a]	2
S15 Techniques to enhance trustworthiness	Techniques to enhance trustworthiness and credibility of data analysis (e.g., member checking, audit trail, triangulation); rationale[a]	0
**Results/Findings**		
S16 Synthesis and interpretation	Main findings (e.g., interpretations, inferences, and themes); might include development of a theory or model, or integration with prior research or theory	2
S17 Links to empirical data	Evidence (e.g., quotes, field notes, text excerpts, photographs) to substantiate analytic findings	2
**Discussion**		
S18 Integration with prior work, implications, transferability, and contribution(s) to the field	Short summary of main findings; explanation of how findings and conclusions connect to, support, elaborate on, or challenge conclusions of earlier scholarship; discussion of scope of application/generalizability; identification of unique contribution(s) to scholarship in a discipline or field	2
S19 Limitations	Trustworthiness and limitations of findings	2
**Other**		
S20 Conflicts of interest	Potential sources of influence or perceived influence on study conduct and conclusions; how these were managed	2
S21 Funding	Sources of funding and other support; role of funders in data collection, interpretation, and reporting	2

Tiwari, S.; Petrov, A.N.; Devlin, M.; Welford, M.; Golosov, N.; DeGroote, J.; Degai, T.; Ksenofontov, S. The second year of pandemic in the Arctic: examining spatiotemporal dynamics of the COVID-19 "Delta wave" in Arctic regions in 2021. International Journal of Circumpolar Health 2022, 81, doi:Artn 2109562

STROBE Statement—checklist of items that should be included in reports of observational studies

	Item No	Recommendation	Recommendation met? (Y/N)
**Title and abstract**	1	(*a*) Indicate the study's design with a commonly used term in the title or the abstract	Y
		(*b*) Provide in the abstract an informative and balanced summary of what was done and what was found	Y
**Introduction**			
Background/rationale	2	Explain the scientific background and rationale for the investigation being reported	Y
Objectives	3	State specific objectives, including any prespecified hypotheses	Y
**Methods**			
Study design	4	Present key elements of study design early in the paper	Y
Setting	5	Describe the setting, locations, and relevant dates, including periods of recruitment, exposure, follow-up, and data collection	Y
Participants	6	(*a*) *Cohort study*—Give the eligibility criteria, and the sources and methods of selection of participants. Describe methods of follow-up *Case-control study*—Give the eligibility criteria, and the sources and methods of case ascertainment and control selection. Give the rationale for the choice of cases and controls *Cross-sectional study*—Give the eligibility criteria, and the sources and methods of selection of participants	Y
		(*b*) *Cohort study*—For matched studies, give matching criteria and number of exposed and unexposed *Case-control study*—For matched studies, give matching criteria and the number of controls per case	N/A
Variables	7	Clearly define all outcomes, exposures, predictors, potential confounders, and effect modifiers. Give diagnostic criteria, if applicable	Y
Data sources/ measurement	8*	For each variable of interest, give sources of data and details of methods of assessment (measurement). Describe comparability of assessment methods if there is more than one group	Y

Bias	9	Describe any efforts to address potential sources of bias	N
Study size	10	Explain how the study size was arrived at	N/A
Quantitative variables	11	Explain how quantitative variables were handled in the analyses. If applicable, describe which groupings were chosen and why	N/A
Statistical methods	12	(*a*) Describe all statistical methods, including those used to control for confounding	N/A
		(*b*) Describe any methods used to examine subgroups and interactions	N/A
		(*c*) Explain how missing data were addressed	N
		(*d*) *Cohort study*—If applicable, explain how loss to follow-up was addressed<br>*Case-control study*—If applicable, explain how matching of cases and controls was addressed<br>*Cross-sectional study*—If applicable, describe analytical methods taking account of sampling strategy	N/A
		(*e*) Describe any sensitivity analyses	N/A
**Results**			
Participants	13*	(a) Report numbers of individuals at each stage of study—eg numbers potentially eligible, examined for eligibility, confirmed eligible, included in the study, completing follow-up, and analysed	Y
		(b) Give reasons for non-participation at each stage	N/A
		(c) Consider use of a flow diagram	N/A
Descriptive data	14*	(a) Give characteristics of study participants (eg demographic, clinical, social) and information on exposures and potential confounders	Y
		(b) Indicate number of participants with missing data for each variable of interest	N/A
		(c) *Cohort study*—Summarise follow-up time (eg, average and total amount)	N/A
Outcome data	15*	*Cohort study*—Report numbers of outcome events or summary measures over time	N/A
		Case-control study—Report numbers in each exposure category, or summary measures of exposure	N/A
		Cross-sectional study—Report numbers of outcome events or summary measures	*Y*
Main results	16	(*a*) Give unadjusted estimates and, if applicable, confounder-adjusted estimates and their precision (eg, 95% confidence interval). Make clear which confounders were adjusted for and why they were included	N/A
		(*b*) Report category boundaries when	N/A

		continuous variables were categorized	
		(*c*) If relevant, consider translating estimates of relative risk into absolute risk for a meaningful time period	N/A
Other analyses	17	Report other analyses done—eg analyses of subgroups and interactions, and sensitivity analyses	N/A
**Discussion**			
Key results	18	Summarise key results with reference to study objectives	Y
Limitations	19	Discuss limitations of the study, taking into account sources of potential bias or imprecision. Discuss both direction and magnitude of any potential bias	Y
Interpretation	20	Give a cautious overall interpretation of results considering objectives, limitations, multiplicity of analyses, results from similar studies, and other relevant evidence	Y
Generalisability	21	Discuss the generalisability (external validity) of the study results	N/A
**Other information**			
Funding	22	Give the source of funding and the role of the funders for the present study and, if applicable, for the original study on which the present article is based	Y

Vilches, T.N.; Abdollahi, E.; Cipriano, L.E.; Haworth-Brockman, M.; Keynan, Y.; Sheffield, H.; Langley, J.M.; Moghadas, S.M. Impact of non-pharmaceutical interventions and vaccination on COVID-19 outbreaks in Nunavut, Canada: a Canadian Immunization Research Network (CIRN) study. BMC Public Health 2022, 22, 1042, doi:10.1186/s12889-022-13432-1.

STROBE Statement—checklist of items that should be included in reports of observational studies

	Item No	Recommendation	Recommendation met? (Y/N)
**Title and abstract**	1	(*a*) Indicate the study's design with a commonly used term in the title or the abstract	Y
		(*b*) Provide in the abstract an informative and balanced summary of what was done and what was found	Y
**Introduction**			
Background/rationale	2	Explain the scientific background and rationale for the investigation being reported	Y
Objectives	3	State specific objectives, including any prespecified hypotheses	Y
**Methods**			
Study design	4	Present key elements of study design early in the paper	Y
Setting	5	Describe the setting, locations, and relevant dates, including periods of recruitment, exposure, follow-up, and data collection	Y
Participants	6	(*a*) *Cohort study*—Give the eligibility criteria, and the sources and methods of selection of participants. Describe methods of follow-up *Case-control study*—Give the eligibility criteria, and the sources and methods of case ascertainment and control selection. Give the rationale for the choice of cases and controls *Cross-sectional study*—Give the eligibility criteria, and the sources and methods of selection of participants	N/A
		(*b*) *Cohort study*—For matched studies, give matching criteria and number of exposed and unexposed *Case-control study*—For matched studies, give matching criteria and the number of controls per case	N/A
Variables	7	Clearly define all outcomes, exposures, predictors, potential confounders, and effect modifiers. Give diagnostic criteria, if applicable	Y
Data sources/ measurement	8*	For each variable of interest, give sources of data and details of methods of assessment (measurement). Describe comparability of	Y

		assessment methods if there is more than one group	
Bias	9	Describe any efforts to address potential sources of bias	Y
Study size	10	Explain how the study size was arrived at	N/A
Quantitative variables	11	Explain how quantitative variables were handled in the analyses. If applicable, describe which groupings were chosen and why	N/A
Statistical methods	12	(*a*) Describe all statistical methods, including those used to control for confounding	N/A
		(*b*) Describe any methods used to examine subgroups and interactions	N/A
		(*c*) Explain how missing data were addressed	N/A
		(*d*) *Cohort study*—If applicable, explain how loss to follow-up was addressed<br>*Case-control study*—If applicable, explain how matching of cases and controls was addressed<br>*Cross-sectional study*—If applicable, describe analytical methods taking account of sampling strategy	N/A
		(*e*) Describe any sensitivity analyses	N/A
**Results**			
Participants	13*	(a) Report numbers of individuals at each stage of study—eg numbers potentially eligible, examined for eligibility, confirmed eligible, included in the study, completing follow-up, and analysed	N/A
		(b) Give reasons for non-participation at each stage	N/A
		(c) Consider use of a flow diagram	N/A
Descriptive data	14*	(a) Give characteristics of study participants (eg demographic, clinical, social) and information on exposures and potential confounders	N/A
		(b) Indicate number of participants with missing data for each variable of interest	N/A
		(c) *Cohort study*—Summarise follow-up time (eg, average and total amount)	N/A
Outcome data	15*	*Cohort study*—Report numbers of outcome events or summary measures over time	N/A
		Case-control study—Report numbers in each exposure category, or summary measures of exposure	N/A
		Cross-sectional study—Report numbers of outcome events or summary measures	*Y*
Main results	16	(*a*) Give unadjusted estimates and, if applicable, confounder-adjusted estimates and their precision (eg, 95% confidence interval). Make clear which confounders were adjusted	N/A

		for and why they were included	
		(*b*) Report category boundaries when continuous variables were categorized	N/A
		(*c*) If relevant, consider translating estimates of relative risk into absolute risk for a meaningful time period	N/A
Other analyses	17	Report other analyses done—eg analyses of subgroups and interactions, and sensitivity analyses	Y
**Discussion**			
Key results	18	Summarise key results with reference to study objectives	Y
Limitations	19	Discuss limitations of the study, taking into account sources of potential bias or imprecision. Discuss both direction and magnitude of any potential bias	Y
Interpretation	20	Give a cautious overall interpretation of results considering objectives, limitations, multiplicity of analyses, results from similar studies, and other relevant evidence	Y
Generalisability	21	Discuss the generalisability (external validity) of the study results	N/A
**Other information**			
Funding	22	Give the source of funding and the role of the funders for the present study and, if applicable, for the original study on which the present article is based	Y

Healey Akearok, G.K.; Rana, Z. Community perspectives on COVID-19 outbreak and public health: Inuit positive protective pathways and lessons for Indigenous public health theory. Can J Public Health 2024, 115, 395-407, doi:10.17269/s41997-024-00863-z.

Standards for Reporting Qualitative Research (SRQR)

**No. Topic**	**Item**	**0 – no 1 – maybe 2-Yes**
**Title and abstract**		
S1 Title	Concise description of the nature and topic of the study identifying the study as qualitative or indicating the approach (e.g., ethnography, grounded theory) or data collection methods (e.g., interview, focus group) is recommended	0
S2 Abstract	Summary of key elements of the study using the abstract format of the intended publication; typically includes objective, methods, results, and conclusions	2
**Introduction**		
S3 Problem formulation	Description and significance of the problem/phenomenon studied; review of relevant theory and empirical work; problem statement	2
S4 Purpose or research question	Purpose of the study and specific objectives or questions	2
**Methods**		
S5 Qualitative approach and research paradigm	Qualitative approach (e.g., ethnography, grounded theory, case study, phenomenology, narrative research) and guiding theory if appropriate; identifying the research paradigm (e.g., positivist, constructivist/interpretivist) is also recommended	0
S6 Researcher characteristics and reflexivity	Researchers’ characteristics that may influence the research, including personal attributes, qualifications/experience, relationship with participants, assumptions, or presuppositions; potential or actual interaction between researchers’ characteristics and the research questions, approach, methods, results, or transferability	0
S7 Context	Setting/site and salient contextual factors; rationale[a]	2
S8 Sampling strategy	How and why research participants, documents, or events were selected; criteria for deciding when no further sampling was necessary (e.g., sampling saturation); rationale[a]	1
S9 Ethical issues pertaining to human subjects	Documentation of approval by an appropriate ethics review board and participant consent, or explanation for lack thereof; other confidentiality and data security issues	2
S10 Data collection methods	Types of data collected; details of data collection procedures including (as appropriate) start and stop dates of data collection	2

	and analysis, iterative process, triangulation of sources/methods, and modification of procedures in response to evolving study findings; rationale[a]	
S11 Data collection instruments and technologies	Description of instruments (e.g., interview guides, questionnaires) and devices (e.g., audio recorders) used for data collection; if/how the instrument(s) changed over the course of the study	1
S12 Units of study	Number and relevant characteristics of participants, documents, or events included in the study; level of participation (could be reported in results)	2
S13 Data processing	Methods for processing data prior to and during analysis, including transcription, data entry, data management and security, verification of data integrity, data coding, and anonymization/deidentification of excerpts	2
S14 Data analysis	Process by which inferences, themes, etc., were identified and developed, including researchers involved in data analysis; usually references a specific paradigm or approach; rationale[a]	2
S15 Techniques to enhance trustworthiness	Techniques to enhance trustworthiness and credibility of data analysis (e.g., member checking, audit trail, triangulation); rationale[a]	0
**Results/Findings**		
S16 Synthesis and interpretation	Main findings (e.g., interpretations, inferences, and themes); might include development of a theory or model, or integration with prior research or theory	2
S17 Links to empirical data	Evidence (e.g., quotes, field notes, text excerpts, photographs) to substantiate analytic findings	2
**Discussion**		
S18 Integration with prior work, implications, transferability, and contribution(s) to the field	Short summary of main findings; explanation of how findings and conclusions connect to, support, elaborate on, or challenge conclusions of earlier scholarship; discussion of scope of application/generalizability; identification of unique contribution(s) to scholarship in a discipline or field	2
S19 Limitations	Trustworthiness and limitations of findings	0
**Other**		
S20 Conflicts of interest	Potential sources of influence or perceived influence on study conduct and conclusions; how these were managed	2
S21 Funding	Sources of funding and other support; role of funders in data collection, interpretation, and reporting	2

Lavoie, J.G.; Clark, W.; McDonnell, L.; Nickel, N.; Dutton, R.; Kanayok, J.; Fowler-Woods, M.; Anawak, J.; Brown, N.; Voisey Clark, G.; et al. Cross-jurisdictional pandemic management: providers speaking on the experience of Nunavut Inuit accessing services in Manitoba during the COVID-19 pandemic. Int J Circumpolar Health 2023, 82, 2259122, doi:10.1080/22423982.2023.2259122.

Standards for Reporting Qualitative Research (SRQR)

**No. Topic**	**Item**	**0 – no 1 – maybe 2-Yes**
**Title and abstract**		
S1 Title	Concise description of the nature and topic of the study identifying the study as qualitative or indicating the approach (e.g., ethnography, grounded theory) or data collection methods (e.g., interview, focus group) is recommended	0
S2 Abstract	Summary of key elements of the study using the abstract format of the intended publication; typically includes objective, methods, results, and conclusions	2
**Introduction**		
S3 Problem formulation	Description and significance of the problem/phenomenon studied; review of relevant theory and empirical work; problem statement	2
S4 Purpose or research question	Purpose of the study and specific objectives or questions	2
**Methods**		
S5 Qualitative approach and research paradigm	Qualitative approach (e.g., ethnography, grounded theory, case study, phenomenology, narrative research) and guiding theory if appropriate; identifying the research paradigm (e.g., positivist, constructivist/interpretivist) is also recommended	0
S6 Researcher characteristics and reflexivity	Researchers' characteristics that may influence the research, including personal attributes, qualifications/experience, relationship with participants, assumptions, or presuppositions; potential or actual interaction between researchers' characteristics and the research questions, approach, methods, results, or transferability	0
S7 Context	Setting/site and salient contextual factors; rationale[a]	2
S8 Sampling strategy	How and why research participants, documents, or events were selected; criteria for deciding when no further sampling was necessary (e.g., sampling saturation); rationale[a]	2
S9 Ethical issues pertaining to human subjects	Documentation of approval by an appropriate ethics review board and participant consent, or explanation for lack thereof; other confidentiality and data security issues	2
S10 Data collection	Types of data collected; details of data collection procedures	2

methods	including (as appropriate) start and stop dates of data collection and analysis, iterative process, triangulation of sources/methods, and modification of procedures in response to evolving study findings; rationale[a]	
S11 Data collection instruments and technologies	Description of instruments (e.g., interview guides, questionnaires) and devices (e.g., audio recorders) used for data collection; if/how the instrument(s) changed over the course of the study	1
S12 Units of study	Number and relevant characteristics of participants, documents, or events included in the study; level of participation (could be reported in results)	2
S13 Data processing	Methods for processing data prior to and during analysis, including transcription, data entry, data management and security, verification of data integrity, data coding, and anonymization/deidentification of excerpts	2
S14 Data analysis	Process by which inferences, themes, etc., were identified and developed, including researchers involved in data analysis; usually references a specific paradigm or approach; rationale[a]	2
S15 Techniques to enhance trustworthiness	Techniques to enhance trustworthiness and credibility of data analysis (e.g., member checking, audit trail, triangulation); rationale[a]	2
**Results/Findings**		
S16 Synthesis and interpretation	Main findings (e.g., interpretations, inferences, and themes); might include development of a theory or model, or integration with prior research or theory	2
S17 Links to empirical data	Evidence (e.g., quotes, field notes, text excerpts, photographs) to substantiate analytic findings	2
**Discussion**		
S18 Integration with prior work, implications, transferability, and contribution(s) to the field	Short summary of main findings; explanation of how findings and conclusions connect to, support, elaborate on, or challenge conclusions of earlier scholarship; discussion of scope of application/generalizability; identification of unique contribution(s) to scholarship in a discipline or field	2
S19 Limitations	Trustworthiness and limitations of findings	0
**Other**		
S20 Conflicts of interest	Potential sources of influence or perceived influence on study conduct and conclusions; how these were managed	2
S21 Funding	Sources of funding and other support; role of funders in data collection, interpretation, and reporting	2

Gilbert, S.Z.; Walsh, D.E.; Levy, S.N.; Maksagak, B.; Milton, M.I.; Ford, J.D.; Hawley, N.L.; Dubrow, R. Determinants, effects, and coping strategies for low-yield periods of harvest: a qualitative study in two communities in Nunavut, Canada. Food Secur 2021, 13, 157-179, doi:10.1007/s12571-020-01112-0.

Standards for Reporting Qualitative Research (SRQR)

**No. Topic**	**Item**	**0 – no 1 – maybe 2-Yes**
**Title and abstract**		
S1 Title	Concise description of the nature and topic of the study identifying the study as qualitative or indicating the approach (e.g., ethnography, grounded theory) or data collection methods (e.g., interview, focus group) is recommended	2
S2 Abstract	Summary of key elements of the study using the abstract format of the intended publication; typically includes objective, methods, results, and conclusions	2
**Introduction**		
S3 Problem formulation	Description and significance of the problem/phenomenon studied; review of relevant theory and empirical work; problem statement	2
S4 Purpose or research question	Purpose of the study and specific objectives or questions	2
**Methods**		
S5 Qualitative approach and research paradigm	Qualitative approach (e.g., ethnography, grounded theory, case study, phenomenology, narrative research) and guiding theory if appropriate; identifying the research paradigm (e.g., positivist, constructivist/interpretivist) is also recommended	1
S6 Researcher characteristics and reflexivity	Researchers' characteristics that may influence the research, including personal attributes, qualifications/experience, relationship with participants, assumptions, or presuppositions; potential or actual interaction between researchers' characteristics and the research questions, approach, methods, results, or transferability	1
S7 Context	Setting/site and salient contextual factors; rationale[a]	2
S8 Sampling strategy	How and why research participants, documents, or events were selected; criteria for deciding when no further sampling was necessary (e.g., sampling saturation); rationale[a]	2
S9 Ethical issues pertaining to human subjects	Documentation of approval by an appropriate ethics review board and participant consent, or explanation for lack thereof; other confidentiality and data security issues	2
S10 Data collection methods	Types of data collected; details of data collection procedures including (as appropriate) start and stop dates of data collection	2

	and analysis, iterative process, triangulation of sources/methods, and modification of procedures in response to evolving study findings; rationale[a]	
S11 Data collection instruments and technologies	Description of instruments (e.g., interview guides, questionnaires) and devices (e.g., audio recorders) used for data collection; if/how the instrument(s) changed over the course of the study	2
S12 Units of study	Number and relevant characteristics of participants, documents, or events included in the study; level of participation (could be reported in results)	2
S13 Data processing	Methods for processing data prior to and during analysis, including transcription, data entry, data management and security, verification of data integrity, data coding, and anonymization/deidentification of excerpts	2
S14 Data analysis	Process by which inferences, themes, etc., were identified and developed, including researchers involved in data analysis; usually references a specific paradigm or approach; rationale[a]	2
S15 Techniques to enhance trustworthiness	Techniques to enhance trustworthiness and credibility of data analysis (e.g., member checking, audit trail, triangulation); rationale[a]	2
**Results/Findings**		
S16 Synthesis and interpretation	Main findings (e.g., interpretations, inferences, and themes); might include development of a theory or model, or integration with prior research or theory	2
S17 Links to empirical data	Evidence (e.g., quotes, field notes, text excerpts, photographs) to substantiate analytic findings	2
**Discussion**		
S18 Integration with prior work, implications, transferability, and contribution(s) to the field	Short summary of main findings; explanation of how findings and conclusions connect to, support, elaborate on, or challenge conclusions of earlier scholarship; discussion of scope of application/generalizability; identification of unique contribution(s) to scholarship in a discipline or field	2
S19 Limitations	Trustworthiness and limitations of findings	0
**Other**		
S20 Conflicts of interest	Potential sources of influence or perceived influence on study conduct and conclusions; how these were managed	2
S21 Funding	Sources of funding and other support; role of funders in data collection, interpretation, and reporting	2

Logie, C.H.; Lys, C.; Sokolovic, N.; Malama, K.; Mackay, K.I.; McNamee, C.; Lad, A.; Kanbari, A. Examining Pathways from Food Insecurity to Safer Sex Efficacy Among Northern and Indigenous Adolescents in the Northwest Territories, Canada. International Journal of Behavioral Medicine 2024, 31, 582-594, doi:10.1007/s12529-023-10195-w.

STROBE Statement—checklist of items that should be included in reports of observational studies

	Item No	Recommendation	Recommendation met? (Y/N)
**Title and abstract**	1	(*a*) Indicate the study's design with a commonly used term in the title or the abstract	Y
		(*b*) Provide in the abstract an informative and balanced summary of what was done and what was found	Y
**Introduction**			
Background/rationale	2	Explain the scientific background and rationale for the investigation being reported	Y
Objectives	3	State specific objectives, including any prespecified hypotheses	Y
**Methods**			
Study design	4	Present key elements of study design early in the paper	Y
Setting	5	Describe the setting, locations, and relevant dates, including periods of recruitment, exposure, follow-up, and data collection	Y
Participants	6	(*a*) *Cohort study*—Give the eligibility criteria, and the sources and methods of selection of participants. Describe methods of follow-up<br>*Case-control study*—Give the eligibility criteria, and the sources and methods of case ascertainment and control selection. Give the rationale for the choice of cases and controls<br>*Cross-sectional study*—Give the eligibility criteria, and the sources and methods of selection of participants	Y
		(*b*) *Cohort study*—For matched studies, give matching criteria and number of exposed and unexposed<br>*Case-control study*—For matched studies, give matching criteria and the number of controls per case	N/A
Variables	7	Clearly define all outcomes, exposures, predictors, potential confounders, and effect modifiers. Give diagnostic criteria, if applicable	Y
Data sources/ measurement	8*	For each variable of interest, give sources of data and details of methods of assessment (measurement). Describe comparability of	Y

		assessment methods if there is more than one group	
Bias	9	Describe any efforts to address potential sources of bias	N
Study size	10	Explain how the study size was arrived at	Y
Quantitative variables	11	Explain how quantitative variables were handled in the analyses. If applicable, describe which groupings were chosen and why	Y
Statistical methods	12	(*a*) Describe all statistical methods, including those used to control for confounding	Y
		(*b*) Describe any methods used to examine subgroups and interactions	Y
		(*c*) Explain how missing data were addressed	Y
		(*d*) *Cohort study*—If applicable, explain how loss to follow-up was addressed<br>*Case-control study*—If applicable, explain how matching of cases and controls was addressed<br>*Cross-sectional study*—If applicable, describe analytical methods taking account of sampling strategy	N/A
		(*e*) Describe any sensitivity analyses	N/A
**Results**			
Participants	13*	(a) Report numbers of individuals at each stage of study—eg numbers potentially eligible, examined for eligibility, confirmed eligible, included in the study, completing follow-up, and analysed	Y
		(b) Give reasons for non-participation at each stage	N/A
		(c) Consider use of a flow diagram	N/A
Descriptive data	14*	(a) Give characteristics of study participants (eg demographic, clinical, social) and information on exposures and potential confounders	Y
		(b) Indicate number of participants with missing data for each variable of interest	Y
		(c) *Cohort study*—Summarise follow-up time (eg, average and total amount)	N/A
Outcome data	15*	*Cohort study*—Report numbers of outcome events or summary measures over time	N/A
		Case-control study—Report numbers in each exposure category, or summary measures of exposure	N/A
		Cross-sectional study—Report numbers of outcome events or summary measures	*Y*
Main results	16	(*a*) Give unadjusted estimates and, if applicable, confounder-adjusted estimates and their precision (eg, 95% confidence interval). Make clear which confounders were adjusted	Y

		for and why they were included	
		(*b*) Report category boundaries when continuous variables were categorized	N/A
		(*c*) If relevant, consider translating estimates of relative risk into absolute risk for a meaningful time period	N/A
Other analyses	17	Report other analyses done—eg analyses of subgroups and interactions, and sensitivity analyses	Y
**Discussion**			
Key results	18	Summarise key results with reference to study objectives	Y
Limitations	19	Discuss limitations of the study, taking into account sources of potential bias or imprecision. Discuss both direction and magnitude of any potential bias	Y
Interpretation	20	Give a cautious overall interpretation of results considering objectives, limitations, multiplicity of analyses, results from similar studies, and other relevant evidence	Y
Generalisability	21	Discuss the generalisability (external validity) of the study results	Y
**Other information**			
Funding	22	Give the source of funding and the role of the funders for the present study and, if applicable, for the original study on which the present article is based	Y

.

Blom, C.D.B.; Steegeman, P.; Voss, C.; Sonneveld, B. Food in the cold: exploring food security and sovereignty in Whitehorse, Yukon. Int J Circumpolar Health 2022, 81, 2025992, doi:10.1080/22423982.2022.2025992.

Mixed Methods Appraisal Tool (MMAT), version 2018

**Category of study designs**	**Methodological quality criteria**	**Responses**			
		Yes	No	Can't tell	Comments
Screening questions (for all types)	S1. Are there clear research questions?	X			
	S2. Do the collected data allow to address the research questions?	X			
	Further appraisal may not be feasible or appropriate when the answer is 'No' or 'Can't tell' to one or both screening questions.				
1. Qualitative	1.1. Is the qualitative approach appropriate to answer the research question?	N/A			
	1.2. Are the qualitative data collection methods adequate to address the research question?	N/A			
	1.3. Are the findings adequately derived from the data?	N/A			
	1.4. Is the interpretation of results sufficiently substantiated by data?	N/A			
	1.5. Is there coherence between qualitative data sources, collection, analysis and interpretation?	N/A			
2. Quantitative randomized controlled trials	2.1. Is randomization appropriately performed?	N/A			
	2.2. Are the groups comparable at baseline?	N/A			
	2.3. Are there complete outcome data?	N/A			
	2.4. Are outcome assessors blinded to the intervention provided?	N/A			
	2.5 Did the participants adhere to the assigned intervention?	N/A			
3. Quantitative non-randomized	3.1. Are the participants representative of the target population?	N/A			
	3.2. Are measurements appropriate regarding both the outcome and intervention (or exposure)?	N/A			
	3.3. Are there complete outcome data?	N/A			
	3.4. Are the confounders accounted for in the design and analysis?	N/A			
	3.5. During the study period, is the intervention administered (or exposure occurred) as intended?	N/A			
4. Quantitative descriptive	4.1. Is the sampling strategy relevant to address the research question?	N/A			
	4.2. Is the sample representative of the target population?	N/A			
	4.3. Are the measurements appropriate?	N/A			
	4.4. Is the risk of nonresponse bias low?	N/A			

	4.5. Is the statistical analysis appropriate to answer the research question?	N/A			
5. Mixed methods	5.1. Is there an adequate rationale for using a mixed methods design to address the research question?	X			
	5.2. Are the different components of the study effectively integrated to answer the research question?	X			
	5.3. Are the outputs of the integration of qualitative and quantitative components adequately interpreted?	X			
	5.4. Are divergences and inconsistencies between quantitative and qualitative results adequately addressed?	X			
	5.5. Do the different components of the study adhere to the quality criteria of each tradition of the methods involved?	X	X		

Egeland, G.M.; Pacey, A.; Cao, Z.; Sobol, I. Food insecurity among Inuit preschoolers: Nunavut Inuit Child Health Survey, 2007-2008. CMAJ 2010, 182, 243-248, doi:10.1503/cmaj.091297.

STROBE Statement—checklist of items that should be included in reports of observational studies

	Item No	Recommendation	Recommendation met? (Y/N)
**Title and abstract**	1	(*a*) Indicate the study's design with a commonly used term in the title or the abstract	Y
		(*b*) Provide in the abstract an informative and balanced summary of what was done and what was found	Y
**Introduction**			
Background/rationale	2	Explain the scientific background and rationale for the investigation being reported	Y
Objectives	3	State specific objectives, including any prespecified hypotheses	Y
**Methods**			
Study design	4	Present key elements of study design early in the paper	Y
Setting	5	Describe the setting, locations, and relevant dates, including periods of recruitment, exposure, follow-up, and data collection	Y
Participants	6	(*a*) *Cohort study*—Give the eligibility criteria, and the sources and methods of selection of participants. Describe methods of follow-up<br>*Case-control study*—Give the eligibility criteria, and the sources and methods of case ascertainment and control selection. Give the rationale for the choice of cases and controls<br>*Cross-sectional study*—Give the eligibility criteria, and the sources and methods of selection of participants	Y
		(*b*) *Cohort study*—For matched studies, give matching criteria and number of exposed and unexposed<br>*Case-control study*—For matched studies, give matching criteria and the number of controls per case	N/A
Variables	7	Clearly define all outcomes, exposures, predictors, potential confounders, and effect modifiers. Give diagnostic criteria, if applicable	Y
Data sources/ measurement	8*	For each variable of interest, give sources of data and details of methods of assessment (measurement). Describe comparability of assessment methods if there is more than one group	Y

Bias	9	Describe any efforts to address potential sources of bias	N
Study size	10	Explain how the study size was arrived at	Y
Quantitative variables	11	Explain how quantitative variables were handled in the analyses. If applicable, describe which groupings were chosen and why	Y
Statistical methods	12	(*a*) Describe all statistical methods, including those used to control for confounding	Y
		(*b*) Describe any methods used to examine subgroups and interactions	N/A
		(*c*) Explain how missing data were addressed	Y
		(*d*) *Cohort study*—If applicable, explain how loss to follow-up was addressed<br>*Case-control study*—If applicable, explain how matching of cases and controls was addressed<br>*Cross-sectional study*—If applicable, describe analytical methods taking account of sampling strategy	Y
		(*e*) Describe any sensitivity analyses	N/A
**Results**			
Participants	13*	(a) Report numbers of individuals at each stage of study—eg numbers potentially eligible, examined for eligibility, confirmed eligible, included in the study, completing follow-up, and analysed	Y
		(b) Give reasons for non-participation at each stage	N/A
		(c) Consider use of a flow diagram	N/A
Descriptive data	14*	(a) Give characteristics of study participants (eg demographic, clinical, social) and information on exposures and potential confounders	Y
		(b) Indicate number of participants with missing data for each variable of interest	N/A
		(c) *Cohort study*—Summarise follow-up time (eg, average and total amount)	N/A
Outcome data	15*	*Cohort study*—Report numbers of outcome events or summary measures over time	N/A
		Case-control study—Report numbers in each exposure category, or summary measures of exposure	N/A
		Cross-sectional study—Report numbers of outcome events or summary measures	*Y*
Main results	16	(*a*) Give unadjusted estimates and, if applicable, confounder-adjusted estimates and their precision (eg, 95% confidence interval). Make clear which confounders were adjusted for and why they were included	Y
		(*b*) Report category boundaries when	N/A

		continuous variables were categorized	
		(*c*) If relevant, consider translating estimates of relative risk into absolute risk for a meaningful time period	N/A
Other analyses	17	Report other analyses done—eg analyses of subgroups and interactions, and sensitivity analyses	N/A
**Discussion**			
Key results	18	Summarise key results with reference to study objectives	Y
Limitations	19	Discuss limitations of the study, taking into account sources of potential bias or imprecision. Discuss both direction and magnitude of any potential bias	Y
Interpretation	20	Give a cautious overall interpretation of results considering objectives, limitations, multiplicity of analyses, results from similar studies, and other relevant evidence	Y
Generalisability	21	Discuss the generalisability (external validity) of the study results	N
**Other information**			
Funding	22	Give the source of funding and the role of the funders for the present study and, if applicable, for the original study on which the present article is based	Y

.

Egeland, G.M.; Johnson-Down, L.; Cao, Z.R.; Sheikh, N.; Weiler, H. Food insecurity and nutrition transition combine to affect nutrient intakes in Canadian arctic communities. J Nutr 2011, 141, 1746-1753, doi:10.3945/jn.111.139006.

STROBE Statement—checklist of items that should be included in reports of observational studies

	Item No	Recommendation	Recommendation met? (Y/N)
**Title and abstract**	1	(*a*) Indicate the study's design with a commonly used term in the title or the abstract	Y
		(*b*) Provide in the abstract an informative and balanced summary of what was done and what was found	Y
**Introduction**			
Background/rationale	2	Explain the scientific background and rationale for the investigation being reported	Y
Objectives	3	State specific objectives, including any prespecified hypotheses	Y
**Methods**			
Study design	4	Present key elements of study design early in the paper	Y
Setting	5	Describe the setting, locations, and relevant dates, including periods of recruitment, exposure, follow-up, and data collection	Y
Participants	6	(*a*) *Cohort study*—Give the eligibility criteria, and the sources and methods of selection of participants. Describe methods of follow-up<br>*Case-control study*—Give the eligibility criteria, and the sources and methods of case ascertainment and control selection. Give the rationale for the choice of cases and controls<br>*Cross-sectional study*—Give the eligibility criteria, and the sources and methods of selection of participants	Y
		(*b*) *Cohort study*—For matched studies, give matching criteria and number of exposed and unexposed<br>*Case-control study*—For matched studies, give matching criteria and the number of controls per case	N/A
Variables	7	Clearly define all outcomes, exposures, predictors, potential confounders, and effect modifiers. Give diagnostic criteria, if applicable	Y
Data sources/ measurement	8*	For each variable of interest, give sources of data and details of methods of assessment (measurement). Describe comparability of assessment methods if there is more than one	Y

		group	
Bias	9	Describe any efforts to address potential sources of bias	N
Study size	10	Explain how the study size was arrived at	Y
Quantitative variables	11	Explain how quantitative variables were handled in the analyses. If applicable, describe which groupings were chosen and why	Y
Statistical methods	12	(*a*) Describe all statistical methods, including those used to control for confounding	Y
		(*b*) Describe any methods used to examine subgroups and interactions	Y
		(*c*) Explain how missing data were addressed	Y
		(*d*) *Cohort study*—If applicable, explain how loss to follow-up was addressed<br>*Case-control study*—If applicable, explain how matching of cases and controls was addressed<br>*Cross-sectional study*—If applicable, describe analytical methods taking account of sampling strategy	N
		(*e*) Describe any sensitivity analyses	N/A
**Results**			
Participants	13*	(a) Report numbers of individuals at each stage of study—eg numbers potentially eligible, examined for eligibility, confirmed eligible, included in the study, completing follow-up, and analysed	Y
		(b) Give reasons for non-participation at each stage	N/A
		(c) Consider use of a flow diagram	N/A
Descriptive data	14*	(a) Give characteristics of study participants (eg demographic, clinical, social) and information on exposures and potential confounders	N
		(b) Indicate number of participants with missing data for each variable of interest	N/A
		(c) *Cohort study*—Summarise follow-up time (eg, average and total amount)	N/A
Outcome data	15*	*Cohort study*—Report numbers of outcome events or summary measures over time	N/A
		Case-control study—Report numbers in each exposure category, or summary measures of exposure	N/A
		Cross-sectional study—Report numbers of outcome events or summary measures	*Y*
Main results	16	(*a*) Give unadjusted estimates and, if applicable, confounder-adjusted estimates and their precision (eg, 95% confidence interval). Make clear which confounders were adjusted for and why they were included	Y

		(*b*) Report category boundaries when continuous variables were categorized	N/A
		(*c*) If relevant, consider translating estimates of relative risk into absolute risk for a meaningful time period	N/A
Other analyses	17	Report other analyses done—eg analyses of subgroups and interactions, and sensitivity analyses	N/A
**Discussion**			
Key results	18	Summarise key results with reference to study objectives	Y
Limitations	19	Discuss limitations of the study, taking into account sources of potential bias or imprecision. Discuss both direction and magnitude of any potential bias	Y
Interpretation	20	Give a cautious overall interpretation of results considering objectives, limitations, multiplicity of analyses, results from similar studies, and other relevant evidence	Y
Generalisability	21	Discuss the generalisability (external validity) of the study results	Y
**Other information**			
Funding	22	Give the source of funding and the role of the funders for the present study and, if applicable, for the original study on which the present article is based	N

.

St-Germain, A.F.; Galloway, T.; Tarasuk, V. Food insecurity in Nunavut following the introduction of Nutrition North Canada. CMAJ 2019, 191, E552-E558, doi:10.1503/cmaj.181617.

STROBE Statement—checklist of items that should be included in reports of observational studies

	Item No	Recommendation	Recommendation met? (Y/N)
**Title and abstract**	1	(*a*) Indicate the study's design with a commonly used term in the title or the abstract	Y
		(*b*) Provide in the abstract an informative and balanced summary of what was done and what was found	Y
**Introduction**			
Background/rationale	2	Explain the scientific background and rationale for the investigation being reported	Y
Objectives	3	State specific objectives, including any prespecified hypotheses	Y
**Methods**			
Study design	4	Present key elements of study design early in the paper	Y
Setting	5	Describe the setting, locations, and relevant dates, including periods of recruitment, exposure, follow-up, and data collection	Y
Participants	6	(*a*) *Cohort study*—Give the eligibility criteria, and the sources and methods of selection of participants. Describe methods of follow-up<br>*Case-control study*—Give the eligibility criteria, and the sources and methods of case ascertainment and control selection. Give the rationale for the choice of cases and controls<br>*Cross-sectional study*—Give the eligibility criteria, and the sources and methods of selection of participants	Y
		(*b*) *Cohort study*—For matched studies, give matching criteria and number of exposed and unexposed<br>*Case-control study*—For matched studies, give matching criteria and the number of controls per case	N/A
Variables	7	Clearly define all outcomes, exposures, predictors, potential confounders, and effect modifiers. Give diagnostic criteria, if applicable	Y
Data sources/ measurement	8*	For each variable of interest, give sources of data and details of methods of assessment (measurement). Describe comparability of assessment methods if there is more than one group	Y

Bias	9	Describe any efforts to address potential sources of bias	N
Study size	10	Explain how the study size was arrived at	Y
Quantitative variables	11	Explain how quantitative variables were handled in the analyses. If applicable, describe which groupings were chosen and why	Y
Statistical methods	12	(*a*) Describe all statistical methods, including those used to control for confounding	Y
		(*b*) Describe any methods used to examine subgroups and interactions	Y
		(*c*) Explain how missing data were addressed	Y
		(*d*) *Cohort study*—If applicable, explain how loss to follow-up was addressed<br>*Case-control study*—If applicable, explain how matching of cases and controls was addressed<br>*Cross-sectional study*—If applicable, describe analytical methods taking account of sampling strategy	Y
		(*e*) Describe any sensitivity analyses	N/A
**Results**			
Participants	13*	(a) Report numbers of individuals at each stage of study—eg numbers potentially eligible, examined for eligibility, confirmed eligible, included in the study, completing follow-up, and analysed	Y
		(b) Give reasons for non-participation at each stage	N/A
		(c) Consider use of a flow diagram	N/A
Descriptive data	14*	(a) Give characteristics of study participants (eg demographic, clinical, social) and information on exposures and potential confounders	Y
		(b) Indicate number of participants with missing data for each variable of interest	N/A
		(c) *Cohort study*—Summarise follow-up time (eg, average and total amount)	N/A
Outcome data	15*	*Cohort study*—Report numbers of outcome events or summary measures over time	N/A
		Case-control study—Report numbers in each exposure category, or summary measures of exposure	N/A
		Cross-sectional study—Report numbers of outcome events or summary measures	*Y*
Main results	16	(*a*) Give unadjusted estimates and, if applicable, confounder-adjusted estimates and their precision (eg, 95% confidence interval). Make clear which confounders were adjusted for and why they were included	Y
		(*b*) Report category boundaries when	N/A

		continuous variables were categorized	
		(*c*) If relevant, consider translating estimates of relative risk into absolute risk for a meaningful time period	N/A
Other analyses	17	Report other analyses done—eg analyses of subgroups and interactions, and sensitivity analyses	N/A
**Discussion**			
Key results	18	Summarise key results with reference to study objectives	Y
Limitations	19	Discuss limitations of the study, taking into account sources of potential bias or imprecision. Discuss both direction and magnitude of any potential bias	Y
Interpretation	20	Give a cautious overall interpretation of results considering objectives, limitations, multiplicity of analyses, results from similar studies, and other relevant evidence	Y
Generalisability	21	Discuss the generalisability (external validity) of the study results	N
**Other information**			
Funding	22	Give the source of funding and the role of the funders for the present study and, if applicable, for the original study on which the present article is based	Y

.

Ford, J.; Berrang-Ford, L. Food security in Igloolik, Nunavut: An exploratory study. Polar Record 2009, 45, 225-236, doi:10.1017/S0032247408008048.

STROBE Statement—checklist of items that should be included in reports of observational studies

	Item No	Recommendation	Recommendation met? (Y/N)
**Title and abstract**	1	(*a*) Indicate the study's design with a commonly used term in the title or the abstract	Y
		(*b*) Provide in the abstract an informative and balanced summary of what was done and what was found	Y
**Introduction**			
Background/rationale	2	Explain the scientific background and rationale for the investigation being reported	Y
Objectives	3	State specific objectives, including any prespecified hypotheses	Y
**Methods**			
Study design	4	Present key elements of study design early in the paper	Y
Setting	5	Describe the setting, locations, and relevant dates, including periods of recruitment, exposure, follow-up, and data collection	Y
Participants	6	(*a*) *Cohort study*—Give the eligibility criteria, and the sources and methods of selection of participants. Describe methods of follow-up<br>*Case-control study*—Give the eligibility criteria, and the sources and methods of case ascertainment and control selection. Give the rationale for the choice of cases and controls<br>*Cross-sectional study*—Give the eligibility criteria, and the sources and methods of selection of participants	Y
		(*b*) *Cohort study*—For matched studies, give matching criteria and number of exposed and unexposed<br>*Case-control study*—For matched studies, give matching criteria and the number of controls per case	N/A
Variables	7	Clearly define all outcomes, exposures, predictors, potential confounders, and effect modifiers. Give diagnostic criteria, if applicable	Y
Data sources/ measurement	8*	For each variable of interest, give sources of data and details of methods of assessment (measurement). Describe comparability of assessment methods if there is more than one group	Y

Bias	9	Describe any efforts to address potential sources of bias	N
Study size	10	Explain how the study size was arrived at	Y
Quantitative variables	11	Explain how quantitative variables were handled in the analyses. If applicable, describe which groupings were chosen and why	Y
Statistical methods	12	(*a*) Describe all statistical methods, including those used to control for confounding	Y
		(*b*) Describe any methods used to examine subgroups and interactions	Y
		(*c*) Explain how missing data were addressed	Y
		(*d*) *Cohort study*—If applicable, explain how loss to follow-up was addressed<br>*Case-control study*—If applicable, explain how matching of cases and controls was addressed<br>*Cross-sectional study*—If applicable, describe analytical methods taking account of sampling strategy	Y
		(*e*) Describe any sensitivity analyses	N/A
**Results**			
Participants	13*	(a) Report numbers of individuals at each stage of study—eg numbers potentially eligible, examined for eligibility, confirmed eligible, included in the study, completing follow-up, and analysed	Y
		(b) Give reasons for non-participation at each stage	N/A
		(c) Consider use of a flow diagram	N/A
Descriptive data	14*	(a) Give characteristics of study participants (eg demographic, clinical, social) and information on exposures and potential confounders	Y
		(b) Indicate number of participants with missing data for each variable of interest	N
		(c) *Cohort study*—Summarise follow-up time (eg, average and total amount)	N/A
Outcome data	15*	*Cohort study*—Report numbers of outcome events or summary measures over time	N/A
		Case-control study—Report numbers in each exposure category, or summary measures of exposure	N/A
		Cross-sectional study—Report numbers of outcome events or summary measures	*Y*
Main results	16	(*a*) Give unadjusted estimates and, if applicable, confounder-adjusted estimates and their precision (eg, 95% confidence interval). Make clear which confounders were adjusted for and why they were included	Y
		(*b*) Report category boundaries when	N/A

		continuous variables were categorized	
		(*c*) If relevant, consider translating estimates of relative risk into absolute risk for a meaningful time period	N/A
Other analyses	17	Report other analyses done—eg analyses of subgroups and interactions, and sensitivity analyses	N/A
**Discussion**			
Key results	18	Summarise key results with reference to study objectives	Y
Limitations	19	Discuss limitations of the study, taking into account sources of potential bias or imprecision. Discuss both direction and magnitude of any potential bias	Y
Interpretation	20	Give a cautious overall interpretation of results considering objectives, limitations, multiplicity of analyses, results from similar studies, and other relevant evidence	Y
Generalisability	21	Discuss the generalisability (external validity) of the study results	Y
**Other information**			
Funding	22	Give the source of funding and the role of the funders for the present study and, if applicable, for the original study on which the present article is based	Y

Beaumier, M.C.; Ford, J.D.; Tagalik, S. The food security of Inuit women in Arviat, Nunavut: the role of socio-economic factors and climate change. Polar Record 2015, 51, 550-559, doi:10.1017/S0032247414000618.

Standards for Reporting Qualitative Research (SRQR)

No. Topic	Item	0 – no<br>1 – maybe<br>2-Yes
**Title and abstract**		
S1 Title	Concise description of the nature and topic of the study identifying the study as qualitative or indicating the approach (e.g., ethnography, grounded theory) or data collection methods (e.g., interview, focus group) is recommended	0
S2 Abstract	Summary of key elements of the study using the abstract format of the intended publication; typically includes objective, methods, results, and conclusions	2
**Introduction**		
S3 Problem formulation	Description and significance of the problem/phenomenon studied; review of relevant theory and empirical work; problem statement	2
S4 Purpose or research question	Purpose of the study and specific objectives or questions	2
**Methods**		
S5 Qualitative approach and research paradigm	Qualitative approach (e.g., ethnography, grounded theory, case study, phenomenology, narrative research) and guiding theory if appropriate; identifying the research paradigm (e.g., positivist, constructivist/interpretivist) is also recommended	1
S6 Researcher characteristics and reflexivity	Researchers' characteristics that may influence the research, including personal attributes, qualifications/experience, relationship with participants, assumptions, or presuppositions; potential or actual interaction between researchers' characteristics and the research questions, approach, methods, results, or transferability	0
S7 Context	Setting/site and salient contextual factors; rationale[a]	2
S8 Sampling strategy	How and why research participants, documents, or events were selected; criteria for deciding when no further sampling was necessary (e.g., sampling saturation); rationale[a]	1
S9 Ethical issues pertaining to human subjects	Documentation of approval by an appropriate ethics review board and participant consent, or explanation for lack thereof; other confidentiality and data security issues	2
S10 Data collection methods	Types of data collected; details of data collection procedures including (as appropriate) start and stop dates of data collection	2

	and analysis, iterative process, triangulation of sources/methods, and modification of procedures in response to evolving study findings; rationale[a]	
S11 Data collection instruments and technologies	Description of instruments (e.g., interview guides, questionnaires) and devices (e.g., audio recorders) used for data collection; if/how the instrument(s) changed over the course of the study	2
S12 Units of study	Number and relevant characteristics of participants, documents, or events included in the study; level of participation (could be reported in results)	2
S13 Data processing	Methods for processing data prior to and during analysis, including transcription, data entry, data management and security, verification of data integrity, data coding, and anonymization/deidentification of excerpts	2
S14 Data analysis	Process by which inferences, themes, etc., were identified and developed, including researchers involved in data analysis; usually references a specific paradigm or approach; rationale[a]	2
S15 Techniques to enhance trustworthiness	Techniques to enhance trustworthiness and credibility of data analysis (e.g., member checking, audit trail, triangulation); rationale[a]	0
**Results/Findings**		
S16 Synthesis and interpretation	Main findings (e.g., interpretations, inferences, and themes); might include development of a theory or model, or integration with prior research or theory	2
S17 Links to empirical data	Evidence (e.g., quotes, field notes, text excerpts, photographs) to substantiate analytic findings	2
**Discussion**		
S18 Integration with prior work, implications, transferability, and contribution(s) to the field	Short summary of main findings; explanation of how findings and conclusions connect to, support, elaborate on, or challenge conclusions of earlier scholarship; discussion of scope of application/generalizability; identification of unique contribution(s) to scholarship in a discipline or field	2
S19 Limitations	Trustworthiness and limitations of findings	0
**Other**		
S20 Conflicts of interest	Potential sources of influence or perceived influence on study conduct and conclusions; how these were managed	2
S21 Funding	Sources of funding and other support; role of funders in data collection, interpretation, and reporting	2

Fraser, S.L.; Nadeau, L. Experience and representations of health and social services in a community of Nunavik. Contemp Nurse 2015, 51, 286-300, doi:10.1080/10376178.2016.1171728.

Standards for Reporting Qualitative Research (SRQR)

**No. Topic**	**Item**	**0 – no 1 – maybe 2-Yes**
**Title and abstract**		
S1 Title	Concise description of the nature and topic of the study identifying the study as qualitative or indicating the approach (e.g., ethnography, grounded theory) or data collection methods (e.g., interview, focus group) is recommended	0
S2 Abstract	Summary of key elements of the study using the abstract format of the intended publication; typically includes objective, methods, results, and conclusions	2
**Introduction**		
S3 Problem formulation	Description and significance of the problem/phenomenon studied; review of relevant theory and empirical work; problem statement	2
S4 Purpose or research question	Purpose of the study and specific objectives or questions	2
**Methods**		
S5 Qualitative approach and research paradigm	Qualitative approach (e.g., ethnography, grounded theory, case study, phenomenology, narrative research) and guiding theory if appropriate; identifying the research paradigm (e.g., positivist, constructivist/interpretivist) is also recommended	1
S6 Researcher characteristics and reflexivity	Researchers' characteristics that may influence the research, including personal attributes, qualifications/experience, relationship with participants, assumptions, or presuppositions; potential or actual interaction between researchers' characteristics and the research questions, approach, methods, results, or transferability	2
S7 Context	Setting/site and salient contextual factors; rationale[a]	2
S8 Sampling strategy	How and why research participants, documents, or events were selected; criteria for deciding when no further sampling was necessary (e.g., sampling saturation); rationale[a]	1
S9 Ethical issues pertaining to human subjects	Documentation of approval by an appropriate ethics review board and participant consent, or explanation for lack thereof; other confidentiality and data security issues	2
S10 Data collection methods	Types of data collected; details of data collection procedures including (as appropriate) start and stop dates of data collection and analysis, iterative process, triangulation of sources/methods,	2

	and modification of procedures in response to evolving study findings; rationale[a]	
S11 Data collection instruments and technologies	Description of instruments (e.g., interview guides, questionnaires) and devices (e.g., audio recorders) used for data collection; if/how the instrument(s) changed over the course of the study	2
S12 Units of study	Number and relevant characteristics of participants, documents, or events included in the study; level of participation (could be reported in results)	2
S13 Data processing	Methods for processing data prior to and during analysis, including transcription, data entry, data management and security, verification of data integrity, data coding, and anonymization/deidentification of excerpts	2
S14 Data analysis	Process by which inferences, themes, etc., were identified and developed, including researchers involved in data analysis; usually references a specific paradigm or approach; rationale[a]	2
S15 Techniques to enhance trustworthiness	Techniques to enhance trustworthiness and credibility of data analysis (e.g., member checking, audit trail, triangulation); rationale[a]	2
**Results/Findings**		
S16 Synthesis and interpretation	Main findings (e.g., interpretations, inferences, and themes); might include development of a theory or model, or integration with prior research or theory	2
S17 Links to empirical data	Evidence (e.g., quotes, field notes, text excerpts, photographs) to substantiate analytic findings	2
**Discussion**		
S18 Integration with prior work, implications, transferability, and contribution(s) to the field	Short summary of main findings; explanation of how findings and conclusions connect to, support, elaborate on, or challenge conclusions of earlier scholarship; discussion of scope of application/generalizability; identification of unique contribution(s) to scholarship in a discipline or field	2
S19 Limitations	Trustworthiness and limitations of findings	0
**Other**		
S20 Conflicts of interest	Potential sources of influence or perceived influence on study conduct and conclusions; how these were managed	2
S21 Funding	Sources of funding and other support; role of funders in data collection, interpretation, and reporting	0
